# Evidences for the augmented Cd(II) biosorption by Cd(II) resistant strain *Candida tropicalis* XTA1874 from contaminated aqueous medium

**DOI:** 10.1038/s41598-023-38485-z

**Published:** 2023-07-25

**Authors:** Kaustav Bhattacharyya, Neelanjan Bhattacharjee, Subhadeep Ganguly

**Affiliations:** 1 Department of Physiology, Vidyasagar College, 39-Sankar Ghosh Lane, Kolkata, West Bengal 700006 India; 2 Department of Mechanical Engineering, University of Alberta, Room 4-31F, 9211 116 Street NW, Edmonton, AB T6G 1H9 Canada

**Keywords:** Microbiology techniques, Biological techniques, Microbiology

## Abstract

Cadmium is one of the most dreadful heavy metals and is becoming a major toxicant in ground water with increasing concentration above the WHO Guidelines in drinking water (0.003 mg/L). The potential sources of cadmium include sewage sludge, phosphate fertilizers and ingredients like Ni–Cd batteries, pigments, plating and plastics. Cadmium levels are increased in water owing to the use and disposal of cadmium containing ingredients. Water draining from a landfill may contain higher cadmium levels. The authors have tried to evaluate the optimized nutritional conditions for the optimal growth and Cd(II) remediation capacity for a developed Cd(II) resistant yeast strain named *Candida tropicalis* XTA 1874 isolated from contaminated water-body in West Bengal. By analyzing the optimization conditions, a synthetic medium was developed and the composition has been given in the main text. The strain showed much better Cd(II) adsorption capacity under the optimized nutritional conditions (Mean removal = 88.077 ± 0.097%).

## Introduction

Heavy metals are originated from the natural environment and are widely distributed in the environment. They are classified as both essential and non essential elements^1^. The term ‘Heavy Metal’ includes the group of metals and metalloids having atomic density greater than 5 g/cm^3^ (Hawkes 1997). The transition metals Chromium (Cr), Copper (Cu), Cobalt (Co), Cadmium (Cd), iron (Fe), Mercury (Hg), Lead (Pb), Molybdenum (Mo), Strontium (Sr), Nickel (Ni), Zinc (Zn), Vanadium (Vi) and Titanium (Ti), the metalloids such as born (Bo), Arsenic (As) are included in this definition^2–4^. The heavy metals tagged as essential act as coenzymes in metabolic reactions whereas the nonessential ones are not and potentially toxic at lower concentrations. They induce various health hazards and spare no organs of our body inducing renal, hematopoietic, cardiovascular, neurological, respiratory, gastro-intestinal and reproductive anomalies^5^. Heavy metals reduce soil fertility by disrupting the balance in soil microbial community and also impose adverse impact on other animals^6^. The worth mentioning of such metals are cadmium, zinc, copper, chromium, mercury, lead and arsenic.

Among these heavy metals, cadmium is widely used in industry and pollutes the environment from unprocessed effluents. The worth mentioning sources are fertilizers and pesticides, nickel–cadmium batteries, smelting, mining, vehicle exhaust, plating and petroleum processing industries. It is non-biodegradable and is stored life-long in our body with increasing body burden. The organs that bear maximum cadmium load are liver and kidney. Elevated cadmium levels are associated with chronic kidney disease^4,7^. The permissible limit of Cd(II) in drinking water is 0.003 mg/L^8^. Drinking water gets contaminated especially from cadmium impurities in zinc-galvanized pipes^9^. Water bodies especially ground water consumed as drinking water in various parts of India, particularly West Bengal, Punjab, Uttar Pradesh, Gujarat and Chennai are bearing Cd(II) concentrations surpassed the above mentioned permissible limit^10–14^. Distribution of cadmium in soil and groundwater and its worldwide status has been explored in detail in the review article^15^.

Various conventional removal methods have been practiced to remove cadmium and other heavy metals from contaminated water since a long time. The worth mentioning are chemical precipitation, reverse osmosis, adsorption, reverse osmosis, ion exchange and other physico-chemical methods^16,17^. With time these methods have become outdated and suffer from severe drawbacks including high operational costs, continual monitoring, inefficiency in toxicant removal from dilute solutions and generation of toxic sludge^18–20^.

Biological remediation techniques such as surface adsorption using biological materials are providing novel strategy for removal of heavy metals like cadmium from contaminated water. Biological adsorption or biosorption provides multiple advantages over the conventional physico-chemical treatment methods such as eco-friendliness, cost effectiveness, high efficiency of metal binding owing to complex surface structure, efficiency of metal removal from dilute aqueous solutions and sorbent regeneration and repeated usage. Biosorbents are quite omnipresent in our environment and can be of microbial origin such as whole cell bacteria, fungi or algae and cell wall saccharide materials derived from them^21–26^. Other biological materials of phytological and zoological origin such as whole plant biomass, stem powder, grape pomace, rose wastes, peat^27–32^. Among all the biological adsorbents used in the remediation process, living microbial cells are quite effective in removing cadmium like toxicants from contaminated water bodies^17^. Living microbial cells have the capacity to remove contaminants both in a metabolism dependent and independent manner^33^. Fungal biosorbents provide multiple *advantages* over other microbial groups. They can be cultivated on large scales using cheap and easy fermentation techniques^34^.

The enhancement of biosorption capacity by empirical optimization is time consuming, costly and laborious. These drawbacks can be overcome by applying response surface methodology using Central-Composite Design. As a result of its application the number of tests decreased and the impact of individual and reciprocal interactions between the factors on the response can be studied.

Hence the present study focuses on the effects of various nutritional factors on aggravating the Cd(II) removal from aqueous solution by using the yeast strain *Candida tropicalis* XTA 1874 by response surface methodology using using Central-Composite Design. The metal biosorption capacity by the strain before and after optimization has also been assessed by using adsorption isotherm and kinetic models. The biomass was further characterized after and before Cd(II) biosorption by using FT-IR, SEM, FESEM and EDAX. Desorption capacity of Cd(II) and the regenerative potential of the biomass has also been explored in the current study.

## Materials and methods

### Preparation of the biomass

The strain *Candida tropicalis* XTA 1874 developed from the obtained yeast strain from the waste water samples from Tollygunge Canal (*2.1. Q4*) was used as the adsorbent in our Cd(II) biosorption study from aqueous suspensions^17^. The strain was proven to be non-pathogenic when tested on swiss albino mice^17^. The growth medium used for the study was the Yeast Extract Peptone Dextrose (YEPD) medium. The composition of the growth medium was Yeast Extract, 0.3%; Peptone, 0.3%; Glucose, 2%^17^. (*2.1. Q1*) The temperature and pH of the sterilized growth medium were ambient (27 ± 2 °C) (*2.1. Q2*) and 6.5 under shaking conditions 180 rpm in rotary shaker (Remi RS 12R). The yeast cells were grown for 24 h (at the end of the exponential phase). Live cell biomass (455 × 10^4^ cells/mL at OD_600_ 0.15) (*2.1. Q3*) was obtained by cell harvesting by centrifugation (11,000×*g*, 15 min)^35^.

### Cd(II) adsorption studies

Cd(II) biosorption experiments were conducted at ambient temperature (27 ± 2 °C) (*2.1. Q2*). Each experiment was carried out in Erlenmeyer flasks containing 100 mL of Cd(II) solution and the flasks were kept shaken 120 rpm for 200 min contact time. Samples were withdrawn at predetermined time intervals (15-500 min) and centrifuged (Remi c24BL) by 8000 rpm for 15 min at 4 °C^36^. The residual Cd(II) concentration in the supernatant was determined by Flame Atomic Absorption Spectroscopy (Shimadzu AA-7000, Japan)^17^ by using the equation:

1 $${\text{q}}_{{\text{e}}} = {\text{V}}\left( {{\text{C}}_{{\text{i}}} - {\text{C}}_{{\text{e}}} } \right)/{\text{m}}$$

where q_e_ is defined as the amount of adsorbed on the biomass in mg/g at equilibrium, V represents the volume of metal containing solution in mL, C_i_ and C_e_ are initial and equilibrium Cd(II) concentration in solution in mg/L and m is the biomass dosage in dry cell mass in g. Percentage removal (%) was determined from the Cd(II) concentration in the supernatant by using the following formula:

2 $${\text{Removal }}\left( \% \right) \, = {\text{ C}}_{{\text{i}}} - {\text{C}}_{{\text{e}}} /{\text{ C}}_{{\text{i}}} \times {1}00$$

In order to determine the amount adsorbed and intracellular accumulation the obtained pellet was undergone some treatments. The pellet thus obtained was washed three times with deionized water and the pellet was treated with 0.1 M EDTA solution for 10 min. The adsorbed Cd(II) over the biomass surface was recovered as EDTA washable fraction and was measured again by Flame Atomic Absorption Spectroscopy (Shimadzu AA-7000, Japan)^37^. The amount that was mobilized intracellularly was enumerated by acid digestion (0.2 N H_2_SO_4_ and HNO_3_) of the biomass and Cd(II) was measured in the lysate by Flame Atomic Absorption Spectroscopy (Shimadzu AA-7000, Japan) at 228.8 nm^38^ (*2.2. Q2*).

### Modeling of biosorption isotherms and kinetics

Langmuir and Freundlich isotherm models have been used to describe the experimental data. Langmuir model describes the formation of monolayer over the adsorbent surface and assumes continuous adsorption energy regardless of the degree of coverage^39–41^.

The Langmuir model is described by the following equation:

3 $${\text{q}}_{{\text{e}}} = {\text{ q}}_{{{\text{max}}}} {\text{K}}_{{\text{L}}} {\text{C}}_{{\text{e}}} /{1} + {\text{ K}}_{{\text{L}}} {\text{C}}_{{\text{e}}}$$

where q_max_ signifies maximum adsorption capacity (mg/g_d.w.),_ K_L_ is the Langmuir constant (L/mg) and C_e_ is the equilibrium Cd(II) concentration in the solution in mg/L.

The reciprocal form of the equation is

4 $${1}/{\text{q}}_{{\text{e}}} = { 1}/{\text{q}}_{{{\text{max}}}} + {1}/{\text{q}}_{{{\text{max}}}} {\text{K}}_{{\text{L}}} \times \left( {{1}/{\text{C}}_{{\text{e}}} } \right)$$

The dimensionless constant R_L_ calculated from the Langmuir isotherm model describing the favorability of the model in describing the adsorption process. The values of R_L_ also known as the separation factor, were calculated from the following equation

5 $${\text{R}}_{{\text{L}}} = { 1}/{1} + {\text{ K}}_{{\text{L}}} {\text{C}}_{{\text{o}}}$$

The Freundlich model is described by the following equation:

6 $${\text{q}}_{{\text{e}}} = {\text{ K}}_{{\text{f}}} \times \, \left( {{\text{C}}_{{\text{e}}} } \right)^{{{1}/{\text{n}}}}$$

K_f_ and 1/n are Freundlich Isotherm constants.

The linearized logarithmic version of the equation is

7 $${\text{Log q}}_{{\text{e}}} = {\text{ Log K}}_{{\text{f}}} + { 1}/{\text{n Log C}}_{{\text{e}}}$$

### Biosorption kinetics

The pseudo-first and second order models have been applied to describe the kinetics of biosorption. The pseudo first order kinetic equation is represented by the equation:

8 $${\text{Ln }}\left( {{\text{q}}_{{\text{e}}} - {\text{q}}_{{\text{t}}} } \right) \, = {\text{ Ln q}}_{{\text{e}}} - {\text{ k}}_{{1}} {\text{t}}$$

where k1 represents pseudo first order rate constant (min^−1^) of Cd(II) adsorption, q_e_ and q_t_ are the amounts of Cd(II) adsorbed at equilibrium and time t (min) respectively. The value of k_1_ was calculated from the slope of the linear plot of Ln (q_e_ − q_t_) verses t. The linearized version of the pseudo second order is represented as:

9 $${\text{t}}/{\text{q}}_{{\text{t}}} = {\text{ t}}/{\text{q}}_{{\text{e}}} + { 1}/{\text{k}}_{{2}} {\text{q}}_{{\text{e}}}^{{2}}$$

where k_2_ represents the pseudo second order rate constant (g/mg/min). The values of k_2_ and q_e_ was obtained from the plot of t/q_t_ verses t.

### Experimentation and optimization of Cd(II) biosorption

The Design Expert Software (DOE, version 13, Stat-Ease Inc, Minneapolis, MN, USA) has been used to fit quadratic model to the experimental data and to determine the best combination of parameters that resulted in the optimum response value. Optimization of Cd(II) biosorption by *Candida tropicalis* XTA 1874 was determined by Central Composite Design (CCD) under Response Surface Methodology (RSM). RSM constitutes a group of empirical techniques evaluating the relationship between clusters of independent variables and the measured responses. Since empirically determining the effects of single factors at a time is time consuming, RSM boosts up the operational conditions as well as save the economy of the process by reducing experimental runs^42^. The modern study depicts the impact of various nutrients influencing the growth and metal bioremediation capability of the resistant strain *Candida tropicalis* XTA 1874. Sixteen independent variables for the current study were: Glucose concentration (%), Urea Concentration (%), K_2_HPO_4_ concentration (%), KH_2_PO_4_ concentration (%), MgSO_4_⋅7H_2_O concentration (%), KCl concentration (%), CoCl_2_⋅5H_2_O concentration (%), NH_4_VO_2_ concentration (%), CaCO_3_ concentration (%), FeSO_4_.7H_2_O concentration (%), ZnSO_4_⋅7H_2_O concentration (%), MnSO_4_⋅4H_2_O concentration (%), NiSO_4_⋅7H_2_O concentration (%), Na_2_B_4_O_7_⋅10H_2_O (%)and dry cell weight (mg/mL). The experimental design with names, symbol codes and actual variable levels has been shown in Table 1 and Supplementary Table 1. The independent variables have been coded by following the equation:

10 $${\text{Z}} = {\text{Z}}_{0} - {\text{Z}}_{{\text{c}}} /\Delta {\text{Z}}$$

where Z and Z_0_ represents coded and actual (uncoded) levels of the independent variables involving seventeen variables [glucose concentration (%), urea concentration (%), K_2_HPO_4_ concentration (%), KH_2_PO_4_ concentration (%), MgSO_4_⋅7H_2_O concentration (%), KCl concentration (%), CoCl_2_⋅5H_2_O concentration (%), NH_4_VO_2_ concentration (%), CaCO_3_ concentration (%), FeSO_4_⋅7H_2_O concentration (%), ZnSO_4_⋅7H_2_O concentration (%), MnSO_4_⋅4H_2_O concentration (%), NiSO_4_⋅7H_2_O concentration (%), Na_2_B_4_O_7_⋅10H_2_O (%) and dry cell weight (mg/ml)]. The step change is indicated by ΔZ and the actual center point value is represented by Z_c_. The interaction among the independent variables and the response was determined by the quadratic equation mentioned below:

11 $$\mathrm{Y}={\sum }_{i=0}^{n}\mathrm{\beta ixi}+{\sum }_{i=0}^{n}\mathrm{\beta i}{\mathrm{xi}}^{2}+{\sum }_{i\ne i=1}^{n}\mathrm{\beta ij xixj}$$

where xi, x^2^_i_, x^2^_j_,….,x^2^_k_, x_i_x_j_, x_i_x_k_ and x_j_x_k_ denote the linear, quadratic and the interaction effects of the variables respectively. The terms β0, βi, βii, and βij are the regression coefficients for the constant, linear, quadratic and interaction terms respectively, the random error is ε and the response variables are indicated by Y. The experimental design along with the alteration in the trend in the variables is shown in Table 2 and Supplementary Table 2. The initial Cd(II) concentration used in the study was 500 ppm Cd(II).

**Table 1:** Independent variables and their corresponding levels for Cd(II) biosorption.

Independent variables	Symbol	Coded levels				
		− α	− 1	0	+ 1	+ α
Glucose concentration (%)	A	6	8	10	12	14
Urea concentration (%)	B	0.6	0.8	1	1.2	1.4
K_2_HPO_4_ concentration (%)	C	0.02	0.06	0.1	0.14	0.18
KH_2_PO_4_ concentration (%)	D	0.02	0.06	0.1	0.14	0.18
MgSO_4_⋅7H_2_O concentration (%)	E	0.005	0.03	0.055	0.08	0.105
KCl concentration (%)	F	0.1	0.4	0.7	1	1.3
CoCl_2_⋅5H_2_O concentration (%)	G	0.01	0.03	0.05	0.07	0.09
NH_4_VO_2_ concentration (%)	H	0.25	0.5	0.75	1	1.25
Na_2_MoO_4_⋅2H_2_O concentration (%)	J	0.02	0.06	0.1	0.14	0.18
CaCO_3_ concentration (%)	K	0.01	0.03	0.05	0.07	0.09
FeSO_4_⋅7H_2_O concentration (%)	L	0.01	0.03	0.05	0.07	0.09
ZnSO_4_⋅7H_2_O concentration (%)	M	0.005	0.03	0.055	0.08	0.105
MnSO_4_⋅4H_2_O concentration (%)	M	0.03	0.04	0.05	0.06	0.07
NiSO_4_⋅7H_2_O concentration (%)	O	0.05	0.06	0.07	0.08	0.09
Na_2_B_4_O_7_⋅10H_2_O concentration (%)	P	0.25	0.5	0.75	1	1.25
Dry cell weight (mg/mL)	Q	0.35	1	1.5	3	4.9

**Table 2:** Experimental design based on central composite design (CCD).

Runs	Independent values																Response		
	A: Glucose conc. (%)	B: Urea conc. (%)	C: K_2_HPO_4_ conc. (%)	D: KH_2_PO_4_ conc. (%)	E: MgSO_4_⋅7H_2_O conc. (%)	F: KCl conc. (%)	G: CoCl_2_⋅6H_2_O conc. (%)	H: NH_4_VO_2_ conc. (%)	I: Na_2_MoO_4_⋅2H_2_O conc. (%)	J: CaCO_3_ conc. (%)	K: FeSO_4_⋅7H_2_O conc. (%)	L: ZnSO_4_⋅7H_2_O conc. (%)	M: MnSO_4_⋅4H_2_O conc. (%)	N: NiSO_4_⋅7H_2_O conc. (%)	O: Na_2_B_4_O_7_⋅10H_2_O conc. (%)	Q: Dry cell weight (mg/ml)	Actual value	Predicted value	Residual
1	10	1	0.1	0.1	0.055	0.7	0.05	0.75	0.1	0.01	0.05	0.055	0.05	0.07	0.75	1.6	90.63	88.39	3.24
2	12	0.8	0.06	0.06	0.03	0.4	0.07	0.5	0.14	0.03	0.07	0.03	0.04	0.08	0.5	1.5	65.37	61.88	3.87
3	8	1.2	0.06	0.06	0.03	0.4	0.03	0.5	0.14	0.07	0.03	0.03	0.04	0.08	0.5	1.3	54.26	57.78	− 1.69
4	12	0.8	0.14	0.06	0.03	1	0.07	1	0.06	0.03	0.03	0.08	0.06	0.08	0.5	2.0	87.55	85.31	4.36
5	8	1.2	0.06	0.14	0.03	0.4	0.07	1	0.06	0.03	0.03	0.03	0.04	0.06	0.5	1.8	56.33	55.88	0.4334
6	8	1.2	0.14	0.14	0.03	0.4	0.03	0.5	0.14	0.03	0.07	0.03	0.04	0.08	0.5	1.9	88.46	89.34	− 0.9966
7	12	0.8	0.06	0.14	0.08	1	0.07	1	0.14	0.07	0.03	0.08	0.04	0.06	1	2.3	86.20	89.00	− 4.79
8	8	1.2	0.14	0.14	0.03	1	0.03	0.5	0.14	0.07	0.03	0.08	0.06	0.08	1	1.9	62.44	63.37	0.9957
9	12	1.2	0.14	0.06	0.08	1	0.03	0.5	0.06	0.03	0.07	0.08	0.06	0.06	1	1.7	88.28	88.25	− 3.01
10	12	0.8	0.14	0.14	0.03	1	0.03	0.5	0.14	0.07	0.03	0.08	0.06	0.06	0.5	1.4	87.12	90.08	− 2.00
11	12	1.2	0.14	0.06	0.03	0.4	0.03	0.5	0.14	0.07	0.07	0.03	0.06	0.08	1	1.8	88.55	86.90	− 0.4366
12	12	0.8	0.06	0.14	0.03	0.4	0.07	0.5	0.06	0.03	0.03	0.08	0.06	0.06	0.5	1.9	52.37	58.43	− 1.96
13	12	1.2	0.06	0.06	0.03	0.4	0.07	1	0.06	0.07	0.03	0.03	0.06	0.06	1	2.0	55.74	61.39	− 1.44
14	8	1.2	0.14	0.14	0.08	1	0.07	1	0.06	0.03	0.03	0.03	0.06	0.06	0.5	1.5	87.74	87.43	0.3079
15	8	0.8	0.14	0.14	0.03	0.4	0.03	1	0.06	0.07	0.03	0.03	0.06	0.06	1	1.8	86.21	89.55	− 2.30
16	8	0.8	0.14	0.06	0.03	0.4	0.07	0.5	0.14	0.03	0.03	0.03	0.06	0.08	1	1.6	90.24	88.63	1.41
17	12	0.8	0.14	0.06	0.03	0.4	0.07	1	0.06	0.07	0.07	0.03	0.04	0.08	1	2.0	61.47	64.44	− 1.01
18	12	1.2	0.14	0.14	0.08	1	0.07	1	0.14	0.07	0.07	0.08	0.06	0.08	1	1.7	88.41	85.67	1.41
19	12	0.8	0.14	0.06	0.08	0.4	0.03	1	0.14	0.03	0.07	0.03	0.06	0.08	1	2.1	53.34	55.56	1.3
20	12	0.8	0.06	0.06	0.03	0.4	0.07	1	0.14	0.03	0.07	0.08	0.06	0.06	1	1.6	90.32	89.89	0.2274
21	12	0.8	0.06	0.06	0.08	1	0.07	1	0.06	0.07	0.07	0.03	0.06	0.08	1	1.9	89.66	87.52	2.10
22	8	0.8	0.06	0.06	0.08	1	0.03	0.5	0.14	0.07	0.07	0.03	0.04	0.06	0.5	1.7	92.25	94.33	− 2.09
23	12	1.2	0.06	0.14	0.08	1	0.07	0.5	0.06	0.03	0.07	0.08	0.06	0.08	0.5	1.4	88.47	89.70	− 0.7241
24	12	1.2	0.14	0.14	0.08	0.4	0.03	1	0.14	0.07	0.07	0.03	0.04	0.06	1	1.9	88.39	88.44	− 0.0238
25	12	1.2	0.14	0.14	0.03	1	0.03	1	0.06	0.03	0.07	0.08	0.04	0.08	1	1.6	89.74	88.02	0.7621
26	8	1.2	0.14	0.06	0.03	1	0.03	1	0.06	0.07	0.07	0.08	0.06	0.08	0.5	1.8	88.32	87.98	− 1.61
27	8	1.2	0.14	0.14	0.03	0.4	0.07	0.5	0.14	0.07	0.03	0.03	0.04	0.06	1	2.0	48.49	53.84	− 7.51
28	8	0.8	0.14	0.14	0.03	1	0.07	1	0.06	0.07	0.03	0.08	0.04	0.08	1	1.7	88.52	88.88	1.68
29	8	0.8	0.06	0.14	0.08	1	0.03	0.5	0.06	0.07	0.03	0.08	0.06	0.08	0.5	1.9	88.04	87.78	− 0.7314
30	8	0.8	0.06	0.14	0.03	0.4	0.07	1	0.14	0.07	0.07	0.08	0.04	0.06	0.5	1.6	82.56	88.55	− 3.47
31	12	1.2	0.06	0.14	0.03	1	0.07	1	0.14	0.03	0.03	0.03	0.06	0.08	0.5	1.8	86.74	92.80	− 3.05
32	8	0.8	0.06	0.14	0.08	0.4	0.03	1	0.06	0.03	0.07	0.08	0.06	0.06	0.5	2.0	82.23	85.27	− 1.05
33	8	1.2	0.14	0.06	0.08	0.4	0.03	1	0.14	0.03	0.07	0.03	0.06	0.06	0.5	1.4	88.23	86.48	− 0.3508
34	12	1.2	0.06	0.14	0.08	1	0.03	0.5	0.06	0.07	0.03	0.08	0.06	0.06	1	1.8	62.52	62.03	0.4624
35	8	1.2	0.14	0.06	0.03	0.4	0.07	1	0.06	0.07	0.07	0.03	0.04	0.06	0.5	1.6	90.14	88.33	1.48
36	8	1.2	0.06	0.06	0.03	0.4	0.07	1	0.14	0.03	0.07	0.08	0.06	0.08	0.5	1.4	58.43	58.76	− 0.3238
37	10	1	0.02	0.1	0.055	0.7	0.05	0.75	0.1	0.05	0.05	0.055	0.05	0.07	0.75	2.1	90.67	91.92	− 1.210
38	8	0.8	0.14	0.14	0.08	0.4	0.07	1	0.14	0.03	0.03	0.03	0.04	0.06	1	1.8	86.88	85.51	1.41
39	8	0.8	0.06	0.06	0.03	1	0.03	1	0.06	0.07	0.03	0.08	0.04	0.06	0.5	1.9	84.70	84.52	0.2377
40	8	0.8	0.14	0.14	0.03	0.4	0.07	0.5	0.06	0.03	0.07	0.08	0.04	0.06	1	1.7	87.55	87.54	− 0.1240
41	12	1.2	0.06	0.06	0.08	0.4	0.07	1	0.14	0.07	0.07	0.03	0.04	0.08	0.5	2.1	82.78	92.44	− 7.70
42	12	1.2	0.06	0.06	0.03	1	0.07	1	0.06	0.03	0.07	0.08	0.04	0.06	0.5	2.0	83.64	87.66	− 1.92
43	8	0.8	0.14	0.14	0.03	0.4	0.03	0.5	0.06	0.07	0.03	0.08	0.04	0.08	0.5	2.0	67.45	62.72	6.17
44	10	1	0.1	0.1	0.055	1.3	0.05	0.75	0.1	0.05	0.05	0.055	0.05	0.07	0.75	1.8	84.52	85.46	1.19
45	12	0.8	0.14	0.06	0.03	0.4	0.03	1	0.06	0.03	0.03	0.03	0.04	0.06	0.5	1.9	54.34	76.62	− 18.28
46	8	0.8	0.14	0.14	0.03	1	0.03	1	0.06	0.03	0.07	0.08	0.04	0.06	0.5	1.8	50.34	53.97	− 1.21
47	8	0.8	0.06	0.14	0.03	1	0.07	1	0.14	0.03	0.03	0.03	0.06	0.06	1	1.6	59.67	59.14	− 1.49
48	12	0.8	0.14	0.14	0.03	1	0.07	0.5	0.14	0.03	0.07	0.08	0.06	0.08	1	1.9	63.26	66.38	− 0.1504
49	8	1.2	0.06	0.06	0.08	0.4	0.03	1	0.06	0.07	0.07	0.08	0.04	0.08	0.5	1.5	62.44	67.69	− 1.23
50	12	0.8	0.06	0.14	0.08	0.4	0.07	0.5	0.14	0.03	0.07	0.08	0.04	0.08	1	2.0	91.30	90.64	− 2.37
51	12	0.8	0.06	0.14	0.03	0.4	0.07	1	0.06	0.03	0.03	0.03	0.04	0.08	1	2.0	92.76	87.47	4.90
52	8	1.2	0.14	0.06	0.03	1	0.03	0.5	0.06	0.07	0.07	0.03	0.04	0.06	1	1.4	68.70	64.82	3.94
53	12	0.8	0.14	0.06	0.08	1	0.03	0.5	0.14	0.07	0.03	0.03	0.06	0.06	1	1.6	64.43	58.66	4.41
54	10	1	0.1	0.1	0.055	0.7	0.05	0.75	0.1	0.05	0.05	0.055	0.05	0.09	0.75	1.8	89.99	91.21	− 0.0640
55	12	1.2	0.06	0.06	0.08	1	0.03	1	0.14	0.07	0.07	0.08	0.06	0.06	0.5	1.8	88.24	84.54	1.82
56	8	0.8	0.14	0.14	0.08	1	0.07	0.5	0.14	0.07	0.07	0.03	0.04	0.08	1	1.9	91.37	90.02	2.525
57	8	0.8	0.14	0.14	0.03	1	0.03	0.5	0.06	0.03	0.07	0.03	0.06	0.08	1	2.0	90.64	91.24	− 1.74
58	8	0.8	0.14	0.06	0.03	0.4	0.07	1	0.14	0.03	0.03	0.08	0.04	0.06	0.5	2.0	59.23	52.35	7.58
59	8	0.8	0.14	0.14	0.08	1	0.03	1	0.14	0.03	0.03	0.08	0.06	0.08	1	2.0	87.45	86.23	0.0217
60	8	1.2	0.14	0.06	0.03	0.4	0.03	0.5	0.06	0.03	0.03	0.08	0.06	0.06	0.5	1.9	89.79	88.87	− 0.0576
61	12	1.2	0.14	0.06	0.03	1	0.07	0.5	0.14	0.07	0.07	0.08	0.04	0.06	1	1.8	91.14	93.61	− 0.8743
62	10	1	0.1	0.1	0.055	0.7	0.05	0.75	0.1	0.05	0.05	0.055	0.05	0.07	0.75	1.9	87.97	91.21	− 2.24
63	10	1	0.1	0.1	0.055	0.7	0.05	0.75	0.1	0.05	0.05	0.055	0.05	0.07	0.75	1.7	85.01	91.12	− 5.20
64	12	0.8	0.14	0.14	0.08	1	0.07	0.5	0.06	0.03	0.03	0.08	0.04	0.06	0.5	1.7	87.14	87.23	0.9455
65	8	0.8	0.06	0.14	0.03	1	0.03	0.5	0.14	0.07	0.07	0.08	0.04	0.06	1	1.2	63.34	57.54	1.81
66	8	1.2	0.06	0.06	0.03	1	0.07	0.5	0.14	0.07	0.03	0.08	0.06	0.06	0.5	1.8	65.47	62.42	5.15
67	12	0.8	0.06	0.14	0.08	0.4	0.03	1	0.14	0.07	0.03	0.03	0.06	0.08	1	1.8	89.12	92.16	− 1.04
68	12	0.8	0.06	0.06	0.03	0.4	0.03	1	0.14	0.07	0.03	0.08	0.06	0.08	0.5	1.7	51.37	52.23	0.1115
69	12	1.2	0.14	0.06	0.08	1	0.03	1	0.06	0.03	0.07	0.03	0.04	0.08	0.5	1.8	54.32	56.32	− 0.9645
70	8	0.8	0.06	0.06	0.03	1	0.03	0.5	0.06	0.07	0.03	0.03	0.06	0.08	1	1.7	58.64	61.34	− 2.75
71	12	1.2	0.14	0.06	0.03	0.4	0.07	1	0.14	0.03	0.03	0.08	0.04	0.08	1	1.8	86.48	89.47	− 2.77
72	8	1.2	0.06	0.14	0.03	1	0.07	0.5	0.06	0.07	0.07	0.03	0.04	0.08	0.5	1.7	48.67	54.41	− 5.56
73	8	1.2	0.06	0.06	0.08	1	0.03	1	0.06	0.03	0.03	0.03	0.06	0.08	1	2.0	88.32	89.52	− 0.8556
74	10	0.6	0.1	0.1	0.055	0.7	0.05	0.75	0.1	0.05	0.05	0.055	0.05	0.07	0.75	2.1	86.57	86.64	0.2134
75	10	1	0.1	0.1	0.055	0.7	0.05	0.75	0.1	0.05	0.05	0.055	0.05	0.07	0.75	1.9	94.76	91.21	3.57
76	12	0.8	0.06	0.14	0.03	1	0.07	1	0.06	0.07	0.07	0.08	0.06	0.08	0.5	1.7	54.27	56.58	− 2.24
77	12	1.2	0.06	0.14	0.03	0.4	0.03	0.5	0.14	0.03	0.03	0.03	0.06	0.08	1	2.1	61.34	58.57	2.41
78	8	0.8	0.06	0.14	0.08	0.4	0.07	1	0.06	0.07	0.03	0.08	0.06	0.08	1	1.8	60.33	62.47	− 2.07
79	8	1.2	0.06	0.14	0.03	1	0.03	1	0.06	0.03	0.03	0.08	0.06	0.08	0.5	2.0	58.76	60.15	− 1.36
80	10	1	0.1	0.1	0.055	0.7	0.05	0.75	0.1	0.05	0.05	0.055	0.05	0.07	0.75	1.9	90.47	91.64	− 0.8208
81	8	0.8	0.14	0.06	0.03	1	0.07	0.5	0.14	0.07	0.07	0.08	0.04	0.08	0.5	1.8	64.37	67.74	− 3.05
82	12	0.8	0.14	0.14	0.08	1	0.07	1	0.06	0.03	0.03	0.03	0.06	0.08	1	1.7	62.42	63.47	− 1.40
83	8	1.2	0.06	0.06	0.03	1	0.07	1	0.14	0.07	0.03	0.03	0.04	0.08	1	1.8	87.99	85.54	2.45
84	10	1	0.1	0.1	0.055	0.7	0.05	0.75	0.1	0.05	0.05	0.105	0.05	0.07	0.75	1.6	88.64	88.57	0.0735
85	8	0.8	0.14	0.06	0.08	1	0.07	0.5	0.06	0.07	0.03	0.08	0.06	0.06	1	1.7	86.21	90.44	− 4.23
86	12	0.8	0.06	0.14	0.08	0.4	0.03	0.5	0.14	0.07	0.03	0.08	0.04	0.06	0.5	1.8	50.24	57.07	− 6.83
87	12	0.8	0.06	0.06	0.08	1	0.03	0.5	0.06	0.03	0.03	0.08	0.04	0.08	1	1.8	87.95	86.51	1.44
88	12	1.2	0.06	0.14	0.08	1	0.07	1	0.06	0.03	0.07	0.03	0.04	0.06	1	1.5	56.32	59.02	− 2.70
89	10	1	0.1	0.1	0.055	0.7	0.05	0.75	0.1	0.05	0.05	0.055	0.05	0.05	0.75	1.9	89.74	89.56	0.1765
90	10	1	0.1	0.1	0.055	0.7	0.01	0.75	0.1	0.05	0.05	0.055	0.05	0.07	0.75	1.9	90.12	88.96	1.16
91	12	1.2	0.06	0.06	0.03	0.4	0.03	0.5	0.06	0.03	0.07	0.08	0.04	0.06	1	1.8	62.41	63.82	− 1.41
92	10	1	0.1	0.1	0.055	0.7	0.05	0.75	0.1	0.05	0.05	0.055	0.05	0.07	0.75	1.9	89.45	91.21	− 1.76
93	12	1.2	0.06	0.06	0.08	0.4	0.07	0.5	0.14	0.07	0.07	0.08	0.06	0.06	1	1.8	54.27	56.68	− 2.41
94	10	1	0.1	0.1	0.055	0.7	0.05	0.75	0.1	0.05	0.05	0.055	0.05	0.07	0.75	1.4	92.47	91.21	1.26
95	8	0.8	0.14	0.14	0.08	1	0.03	0.5	0.14	0.03	0.03	0.03	0.04	0.06	0.5	1.7	58.74	62.60	− 3.86
96	12	1.2	0.06	0.14	0.03	1	0.03	1	0.14	0.07	0.07	0.03	0.06	0.06	1	2.0	46.37	54.28	− 7.91
97	10	1	0.1	0.1	0.055	0.7	0.09	0.75	0.1	0.05	0.05	0.055	0.05	0.07	0.75	1.7	89.64	90.63	− 0.9938
98	8	1.2	0.14	0.14	0.03	0.4	0.07	1	0.14	0.07	0.03	0.08	0.06	0.08	0.5	1.8	88.21	92.90	− 4.69
99	12	1.2	0.14	0.14	0.08	1	0.03	0.5	0.14	0.03	0.03	0.03	0.04	0.08	1	1.7	86.33	88.84	− 2.51
100	8	1.2	0.14	0.14	0.03	0.4	0.03	1	0.14	0.03	0.07	0.08	0.06	0.06	1	1.7	52.37	53.07	− 0.7025
101	8	0.8	0.06	0.06	0.03	1	0.07	1	0.06	0.03	0.07	0.08	0.04	0.08	1	1.6	49.65	55.11	− 5.46
102	12	0.8	0.06	0.06	0.08	1	0.03	1	0.06	0.03	0.03	0.03	0.06	0.06	0.5	1.5	64.27	62.74	1.53
103	8	0.8	0.06	0.14	0.08	1	0.07	0.5	0.06	0.03	0.07	0.08	0.06	0.06	1	1.3	64.21	58.35	5.86
104	8	0.8	0.06	0.06	0.03	0.4	0.07	0.5	0.06	0.07	0.03	0.08	0.04	0.06	1	2.0	64.52	64.40	0.1244
105	12	1.2	0.06	0.14	0.08	0.4	0.03	0.5	0.06	0.03	0.07	0.03	0.04	0.06	0.5	1.8	88.47	85.35	3.12
106	8	1.2	0.06	0.14	0.03	1	0.03	0.5	0.06	0.03	0.03	0.03	0.04	0.06	1	1.9	86.54	90.61	− 4.07
107	8	1.2	0.14	0.06	0.03	1	0.07	0.5	0.06	0.03	0.03	0.03	0.04	0.08	0.5	2.3	87.99	84.89	3.10
108	12	1.2	0.14	0.14	0.03	1	0.03	0.5	0.06	0.03	0.07	0.03	0.06	0.06	0.5	1.9	58.74	56.50	2.24
109	10	1	0.18	0.1	0.055	0.7	0.05	0.75	0.1	0.05	0.05	0.055	0.05	0.07	0.75	1.7	92.12	90.75	1.37
110	12	1.2	0.14	0.06	0.03	0.4	0.03	1	0.14	0.07	0.07	0.08	0.04	0.06	0.5	1.4	56.34	58.39	− 2.05
111	8	1.2	0.14	0.14	0.03	1	0.07	1	0.14	0.03	0.07	0.03	0.04	0.08	1	1.8	52.31	53.17	− 0.8634
112	8	1.2	0.14	0.14	0.08	0.4	0.03	1	0.06	0.03	0.03	0.08	0.04	0.08	0.5	1.9	88.52	89.94	− 1.42
113	8	0.8	0.14	0.14	0.08	0.4	0.03	1	0.14	0.07	0.07	0.03	0.04	0.08	0.5	2.0	60.12	62.25	− 2.13
114	8	1.2	0.06	0.14	0.08	0.4	0.03	0.5	0.14	0.07	0.03	0.08	0.04	0.08	1	1.5	88.58	83.56	5.02
115	12	1.2	0.06	0.14	0.03	1	0.03	0.5	0.14	0.07	0.07	0.08	0.04	0.08	0.5	1.8	88.47	89.95	− 1.48
116	8	1.2	0.06	0.06	0.08	0.4	0.03	0.5	0.06	0.07	0.07	0.03	0.06	0.06	1	1.6	89.24	90.75	− 1.51
117	12	1.2	0.14	0.06	0.03	1	0.07	1	0.14	0.07	0.07	0.03	0.06	0.08	0.5	2.0	56.34	56.22	0.1244
118	12	0.8	0.14	0.14	0.03	0.4	0.03	0.5	0.14	0.03	0.07	0.03	0.04	0.06	1	1.7	54.02	52.09	1.93
119	12	0.8	0.06	0.14	0.03	0.4	0.03	1	0.06	0.07	0.07	0.03	0.04	0.06	0.5	2.1	62.34	54.55	7.79
120	12	1.2	0.06	0.06	0.08	1	0.03	0.5	0.14	0.07	0.07	0.03	0.04	0.08	1	1.6	58.74	57.59	1.15
121	6	1	0.1	0.1	0.055	0.7	0.05	0.75	0.1	0.05	0.05	0.055	0.05	0.07	0.75	1.9	92.45	89.56	2.89
122	10	1	0.1	0.1	0.055	0.7	0.05	0.75	0.18	0.05	0.05	0.055	0.05	0.07	0.75	1.7	86.22	89.01	− 2.79
123	8	0.8	0.14	0.06	0.03	0.4	0.03	0.5	0.14	0.07	0.07	0.03	0.06	0.06	0.5	1.4	48.75	53.83	− 5.08
124	8	1.2	0.06	0.14	0.08	0.4	0.07	0.5	0.14	0.03	0.07	0.08	0.04	0.06	0.5	1.9	58.74	56.96	1.78
125	12	0.8	0.06	0.06	0.03	1	0.07	0.5	0.14	0.07	0.03	0.08	0.06	0.08	1	1.6	86.55	89.96	− 3.41
126	8	0.8	0.14	0.06	0.03	0.4	0.03	1	0.14	0.07	0.07	0.08	0.04	0.08	1	1.8	88.12	90.52	− 2.40
127	12	0.8	0.14	0.14	0.03	0.4	0.03	1	0.14	0.03	0.07	0.08	0.06	0.08	0.5	2.0	89.21	86.38	2.83
128	10	1	0.1	0.1	0.055	0.7	0.05	0.75	0.1	0.05	0.05	0.055	0.05	0.07	0.75	1.7	86.56	91.21	− 4.65
129	12	1.2	0.14	0.14	0.08	1	0.07	0.5	0.14	0.07	0.07	0.03	0.04	0.06	0.5	1.9	58.74	54.29	4.45
130	8	0.8	0.14	0.06	0.03	1	0.03	1	0.14	0.03	0.03	0.03	0.06	0.08	0.5	1.6	56.00	55.43	0.5704
131	12	0.8	0.14	0.14	0.08	1	0.03	1	0.06	0.07	0.07	0.03	0.06	0.06	0.5	1.8	88.97	88.48	0.4907
132	12	1.2	0.06	0.06	0.03	1	0.03	0.5	0.06	0.07	0.03	0.03	0.06	0.06	0.5	2.0	84.72	84.87	− 0.1508
133	12	0.8	0.06	0.14	0.03	1	0.03	0.5	0.06	0.03	0.03	0.03	0.04	0.08	0.5	1.4	62.14	61.69	0.4455
134	12	1.2	0.14	0.14	0.03	0.4	0.03	0.5	0.06	0.07	0.03	0.08	0.04	0.06	1	1.8	88.14	86.94	1.20
135	10	1	0.1	0.1	0.055	0.7	0.05	0.75	0.1	0.05	0.05	0.055	0.07	0.07	0.75	1.6	86.54	87.81	− 1.27
136	12	0.8	0.06	0.06	0.08	1	0.07	0.5	0.06	0.07	0.07	0.08	0.04	0.06	0.5	1.4	66.34	64.04	2.30
137	12	1.2	0.14	0.14	0.08	0.4	0.03	0.5	0.14	0.07	0.07	0.08	0.06	0.08	0.5	2.1	58.75	56.97	1.78
138	8	1.2	0.06	0.14	0.08	1	0.07	1	0.14	0.07	0.03	0.08	0.04	0.08	0.5	1.8	67.45	61.41	6.04
139	8	0.8	0.06	0.14	0.08	1	0.03	1	0.06	0.07	0.03	0.03	0.04	0.06	1	1.9	74.12	71.04	3.08
140	12	0.8	0.14	0.14	0.08	0.4	0.03	0.5	0.06	0.03	0.03	0.03	0.06	0.08	0.5	1.7	88.41	90.89	− 2.48
141	12	1.2	0.06	0.06	0.08	0.4	0.03	0.5	0.14	0.03	0.03	0.08	0.06	0.08	0.5	2.1	87.12	86.74	0.3794
142	8	0.8	0.06	0.06	0.03	1	0.07	0.5	0.06	0.03	0.07	0.03	0.06	0.06	0.5	2.0	90.24	88.79	1.45
143	12	0.8	0.06	0.06	0.08	0.4	0.03	1	0.06	0.07	0.07	0.08	0.04	0.06	1	2.0	92.34	91.87	0.4689
144	10	1	0.1	0.1	0.055	0.7	0.05	0.75	0.1	0.05	0.05	0.055	0.03	0.07	0.75	1.8	90.24	88.80	1.44
145	12	0.8	0.14	0.14	0.03	1	0.07	1	0.14	0.03	0.07	0.03	0.04	0.06	0.5	1.9	88.72	84.76	3.96
146	8	0.8	0.06	0.14	0.08	1	0.07	1	0.06	0.03	0.07	0.03	0.04	0.08	0.5	1.8	88.09	85.05	3.04
147	10	1	0.1	0.1	0.055	0.7	0.05	0.75	0.1	0.05	0.05	0.055	0.05	0.07	0.75	1.6	36.45	32.28	4.17
148	12	0.8	0.14	0.06	0.03	1	0.07	0.5	0.06	0.03	0.03	0.03	0.04	0.06	1	1.9	62.14	59.35	2.79
149	8	1.2	0.14	0.14	0.03	1	0.03	1	0.14	0.07	0.03	0.03	0.04	0.06	0.5	1.5	89.45	86.02	3.43
150	12	1.2	0.06	0.06	0.03	0.4	0.03	1	0.06	0.03	0.07	0.03	0.06	0.08	0.5	2.0	89.12	87.70	1.42
151	10	1	0.1	0.02	0.055	0.7	0.05	0.75	0.1	0.05	0.05	0.055	0.05	0.07	0.75	2.0	88.02	87.51	0.5088
152	12	0.8	0.14	0.14	0.03	1	0.03	1	0.14	0.07	0.03	0.03	0.04	0.08	1	1.4	62.21	61.22	0.9950
153	8	0.8	0.06	0.14	0.08	0.4	0.03	0.5	0.06	0.03	0.07	0.03	0.04	0.08	1	1.6	52.47	53.54	− 1.07
154	10	1.4	0.1	0.1	0.055	0.7	0.05	0.75	0.1	0.05	0.05	0.055	0.05	0.07	0.75	1.8	86.02	86.08	− 0.0553
155	8	1.2	0.06	0.14	0.03	1	0.07	1	0.06	0.07	0.07	0.08	0.06	0.06	1	1.8	90.37	85.64	4.73
156	8	0.8	0.14	0.06	0.03	1	0.07	1	0.14	0.07	0.07	0.03	0.06	0.06	1	1.9	88.52	87.87	0.6464
157	8	1.2	0.06	0.06	0.03	1	0.03	0.5	0.14	0.03	0.07	0.08	0.06	0.08	1	2.0	92.14	90.02	2.12
158	12	0.8	0.14	0.06	0.03	1	0.03	1	0.06	0.07	0.07	0.08	0.06	0.06	1	2.0	54.27	54.88	− 0.6080
159	12	1.2	0.06	0.06	0.08	1	0.07	1	0.14	0.03	0.03	0.08	0.06	0.08	1	2.0	62.47	59.19	3.28
160	8	1.2	0.14	0.14	0.08	1	0.03	1	0.06	0.07	0.07	0.03	0.06	0.08	1	1.9	58.01	59.85	− 1.84
161	12	0.8	0.14	0.06	0.08	0.4	0.03	0.5	0.14	0.03	0.07	0.08	0.04	0.06	0.5	1.8	90.99	85.10	5.89
162	12	0.8	0.14	0.06	0.08	0.4	0.07	0.5	0.14	0.07	0.03	0.08	0.04	0.08	1	1.9	58.47	58.50	− 0.0257
163	12	1.2	0.06	0.14	0.08	0.4	0.07	0.5	0.06	0.07	0.03	0.03	0.04	0.08	1	1.7	54.23	57.31	− 3.08
164	12	1.2	0.06	0.14	0.03	0.4	0.03	1	0.14	0.03	0.03	0.08	0.04	0.06	0.5	1.7	86.07	83.65	2.42
165	8	1.2	0.06	0.14	0.03	0.4	0.03	0.5	0.06	0.07	0.07	0.08	0.06	0.06	0.5	1.2	52.47	56.74	− 4.27
166	12	1.2	0.14	0.06	0.08	0.4	0.07	0.5	0.06	0.03	0.07	0.03	0.04	0.08	1	1.8	91.88	88.51	3.37
167	12	1.2	0.14	0.14	0.08	0.4	0.07	0.5	0.14	0.03	0.03	0.08	0.06	0.06	1	1.8	86.45	86.09	0.3581
168	12	0.8	0.14	0.06	0.03	1	0.03	0.5	0.06	0.07	0.07	0.03	0.04	0.08	0.5	1.7	92.12	89.69	2.43
169	12	1.2	0.14	0.14	0.03	0.4	0.07	0.5	0.06	0.03	0.07	0.08	0.04	0.08	0.5	1.8	56.74	61.57	− 4.83
170	8	0.8	0.06	0.06	0.08	0.4	0.07	0.5	0.14	0.07	0.07	0.08	0.06	0.08	0.5	1.7	86.25	88.63	− 2.38
171	8	1.2	0.06	0.06	0.08	0.4	0.07	1	0.06	0.03	0.03	0.08	0.04	0.06	1	1.8	88.52	90.45	− 1.93
172	12	0.8	0.06	0.06	0.03	1	0.03	1	0.14	0.03	0.07	0.03	0.04	0.08	1	1.7	88.45	90.34	− 1.89
173	8	1.2	0.14	0.06	0.08	0.4	0.03	0.5	0.14	0.03	0.07	0.08	0.04	0.08	1	2.0	55.45	57.95	− 2.50
174	8	0.8	0.06	0.06	0.08	1	0.07	0.5	0.14	0.03	0.03	0.03	0.04	0.08	1	2.1	59.67	61.91	− 2.24
175	12	1.2	0.14	0.06	0.08	0.4	0.03	1	0.06	0.07	0.03	0.08	0.06	0.08	1	1.9	88.03	88.74	− 0.7101
176	8	1.2	0.14	0.14	0.08	1	0.03	0.5	0.06	0.07	0.07	0.08	0.04	0.06	0.5	1.7	88.01	90.66	− 2.65
177	8	0.8	0.14	0.14	0.08	0.4	0.03	0.5	0.14	0.07	0.07	0.08	0.06	0.06	1	2.1	88.12	86.02	2.10
178	12	1.2	0.14	0.06	0.03	1	0.03	0.5	0.14	0.03	0.03	0.08	0.04	0.08	0.5	1.8	58.99	61.46	− 2.47
179	12	0.8	0.14	0.14	0.03	0.4	0.07	0.5	0.14	0.07	0.03	0.03	0.04	0.08	0.5	2.0	88.25	89.94	− 1.69
180	12	0.8	0.06	0.14	0.03	1	0.03	1	0.06	0.03	0.03	0.08	0.06	0.06	1	1.982	91.45	85.13	6.32
181	12	0.8	0.14	0.06	0.08	1	0.03	1	0.14	0.07	0.03	0.08	0.04	0.08	0.5	1.813	86.22	82.79	3.43
182	8	1.2	0.06	0.06	0.08	1	0.07	1	0.06	0.07	0.07	0.03	0.06	0.06	0.5	1.701	58.27	58.16	0.1088
183	8	1.2	0.14	0.14	0.08	0.4	0.07	0.5	0.06	0.07	0.07	0.03	0.06	0.08	0.5	1.850	90.07	86.42	3.65
184	10	1	0.1	0.1	0.055	0.7	0.05	0.75	0.1	0.05	0.05	0.055	0.05	0.07	0.75	1.628	94.64	91.21	3.43
185	8	0.8	0.14	0.06	0.08	0.4	0.07	1	0.06	0.03	0.07	0.08	0.06	0.08	1	1.795	88.12	88.85	− 0.7288
186	12	0.8	0.14	0.06	0.08	0.4	0.07	1	0.14	0.07	0.03	0.03	0.06	0.06	0.5	1.826	88.97	83.51	5.46
187	10	1	0.1	0.1	0.005	0.7	0.05	0.75	0.1	0.05	0.05	0.055	0.05	0.07	0.75	1.823	89.97	87.55	2.42
188	8	0.8	0.06	0.06	0.03	0.4	0.03	1	0.06	0.03	0.07	0.03	0.06	0.06	1	1.515	52.47	53.76	− 1.29
189	10	1	0.1	0.1	0.055	0.7	0.05	0.75	0.1	0.05	0.05	0.055	0.05	0.07	1.25	1.9	92.78	93.08	− 0.2997
190	12	0.8	0.14	0.06	0.08	1	0.07	1	0.14	0.03	0.07	0.08	0.04	0.06	1	1.9	58.97	60.23	− 1.26
191	10	1	0.1	0.1	0.055	0.7	0.05	0.75	0.1	0.09	0.05	0.055	0.05	0.07	0.75	1.8	88.12	91.24	− 3.12
192	8	1.2	0.14	0.06	0.08	1	0.03	1	0.14	0.07	0.03	0.08	0.04	0.06	1	1.9	52.14	58.76	− 6.62
193	12	1.2	0.06	0.06	0.08	1	0.07	0.5	0.14	0.03	0.03	0.03	0.04	0.06	0.5	1.8	88.21	88.03	0.1813
194	8	0.8	0.06	0.14	0.03	1	0.03	1	0.14	0.07	0.07	0.03	0.06	0.08	0.5	1.4	87.68	86.40	1.28
195	8	1.2	0.14	0.14	0.08	1	0.07	0.5	0.06	0.03	0.03	0.08	0.04	0.08	1	1.7	56.98	56.15	0.8325
196	10	1	0.1	0.1	0.055	0.7	0.05	0.75	0.02	0.05	0.05	0.055	0.05	0.07	0.75	2.0	92.45	89.50	2.95
197	12	0.8	0.14	0.06	0.03	0.4	0.07	0.5	0.06	0.07	0.07	0.08	0.06	0.06	0.5	1.7	88.12	87.09	1.03
198	10	1	0.1	0.1	0.055	0.7	0.05	0.75	0.1	0.05	0.05	0.055	0.05	0.07	0.75	1.8	94.52	91.21	3.31
199	12	1.2	0.14	0.06	0.03	0.4	0.07	0.5	0.14	0.03	0.03	0.03	0.06	0.06	0.5	1.7	49.74	51.80	− 2.06
200	8	1.2	0.14	0.06	0.08	0.4	0.07	1	0.14	0.07	0.03	0.03	0.06	0.08	1	1.7	56.07	58.93	− 2.86
201	10	1	0.1	0.1	0.105	0.7	0.05	0.75	0.1	0.05	0.05	0.055	0.05	0.07	0.75	1.6	86.45	88.71	− 2.26
202	8	1.2	0.06	0.06	0.03	0.4	0.03	1	0.14	0.07	0.03	0.08	0.06	0.06	1	1.5	86.07	84.23	1.84
203	12	0.8	0.06	0.06	0.08	0.4	0.07	0.5	0.06	0.03	0.03	0.03	0.06	0.06	1	1.3	92.99	88.98	4.01
204	8	1.2	0.06	0.14	0.08	1	0.03	0.5	0.14	0.03	0.07	0.03	0.06	0.08	0.5	2.0	55.24	55.81	− 0.5670
205	12	0.8	0.06	0.06	0.08	0.4	0.07	1	0.06	0.03	0.03	0.08	0.04	0.08	0.5	1.8	62.34	59.91	2.43
206	8	0.8	0.06	0.14	0.03	0.4	0.07	0.5	0.14	0.07	0.07	0.03	0.06	0.08	1	1.9	60.12	56.06	4.06
207	8	0.8	0.06	0.06	0.08	0.4	0.03	0.5	0.14	0.03	0.03	0.08	0.06	0.06	1	2.3	52.14	53.60	− 1.46
208	12	0.8	0.06	0.06	0.03	1	0.07	1	0.14	0.07	0.03	0.03	0.04	0.06	0.5	1.9	58.45	64.08	− 5.63
209	12	1.2	0.14	0.14	0.08	0.4	0.07	1	0.14	0.03	0.03	0.03	0.04	0.08	0.5	1.7	62.45	61.49	0.9636
210	10	1	0.1	0.1	0.055	0.7	0.05	0.75	0.1	0.05	0.05	0.055	0.05	0.07	0.25	1.4	92.74	92.28	0.4612
211	12	1.2	0.14	0.14	0.03	1	0.07	0.5	0.06	0.07	0.03	0.03	0.06	0.08	1	1.8	88.45	89.68	− 1.23
212	8	0.8	0.06	0.06	0.08	0.4	0.03	1	0.14	0.03	0.03	0.03	0.04	0.08	0.5	1.9	90.64	86.92	3.72
213	8	0.8	0.06	0.06	0.03	0.4	0.03	0.5	0.06	0.03	0.07	0.08	0.04	0.08	0.5	2.0	88.24	86.26	1.98
214	12	0.8	0.14	0.06	0.03	0.4	0.03	0.5	0.06	0.03	0.03	0.08	0.06	0.08	1	1.5	50.41	55.92	− 5.51
215	12	0.8	0.14	0.14	0.08	0.4	0.07	0.5	0.06	0.07	0.07	0.03	0.06	0.06	1	1.8	48.75	54.84	− 6.09
216	8	1.2	0.06	0.14	0.03	0.4	0.03	1	0.06	0.07	0.07	0.03	0.04	0.08	1	1.6	88.34	91.35	− 3.01
217	12	0.8	0.06	0.06	0.03	0.4	0.03	0.5	0.14	0.07	0.03	0.03	0.04	0.06	1	2.0	88.78	85.22	3.56
218	8	0.8	0.06	0.14	0.03	0.4	0.03	1	0.14	0.03	0.03	0.08	0.04	0.08	1	1.7	42.12	50.82	− 8.70
219	12	1.2	0.14	0.14	0.03	1	0.07	1	0.06	0.07	0.03	0.08	0.04	0.06	0.5	2.1	58.74	59.96	− 1.22
220	10	1	0.1	0.1	0.055	0.7	0.05	0.75	0.1	0.05	0.09	0.055	0.05	0.07	0.75	1.6	90.99	85.05	5.94
221	12	1.2	0.06	0.14	0.03	0.4	0.07	1	0.14	0.07	0.07	0.08	0.04	0.08	1	1.9	70.24	63.59	6.65
222	8	0.8	0.14	0.06	0.08	0.4	0.07	0.5	0.06	0.03	0.07	0.03	0.04	0.06	0.5	1.7	49.87	54.05	− 4.18
223	8	1.2	0.14	0.06	0.08	1	0.07	0.5	0.14	0.03	0.07	0.03	0.06	0.06	1	1.4	52.41	53.03	− 0.6163
224	8	1.2	0.06	0.06	0.08	0.4	0.07	0.5	0.06	0.03	0.03	0.03	0.06	0.08	0.5	1.9	54.12	56.55	− 2.43
225	8	0.8	0.14	0.06	0.08	0.4	0.03	1	0.06	0.07	0.03	0.08	0.06	0.06	0.5	1.6	66.54	61.67	4.87
226	8	0.8	0.14	0.06	0.03	1	0.03	0.5	0.14	0.03	0.03	0.08	0.04	0.06	1	1.8	90.64	86.61	4.03
227	8	0.8	0.14	0.06	0.08	1	0.03	1	0.06	0.03	0.07	0.03	0.04	0.06	1	2.0	87.99	90.02	− 2.03
228	8	0.8	0.06	0.06	0.08	0.4	0.07	1	0.14	0.07	0.07	0.03	0.04	0.06	1	1.7	76.85	69.24	7.61
229	10	1	0.1	0.1	0.055	0.7	0.05	0.75	0.1	0.05	0.01	0.055	0.05	0.07	0.75	1.9	79.89	85.67	− 5.78
230	12	1.2	0.06	0.06	0.03	0.4	0.07	0.5	0.06	0.07	0.03	0.08	0.04	0.08	0.5	1.6	91.32	87.42	3.90
231	12	1.2	0.06	0.14	0.08	0.4	0.03	1	0.06	0.03	0.07	0.08	0.06	0.08	1	1.8	59.32	59.35	− 0.0338
232	12	0.8	0.06	0.06	0.08	0.4	0.03	0.5	0.06	0.07	0.07	0.03	0.06	0.08	0.5	2.0	52.37	53.63	− 1.26
233	12	1.2	0.14	0.06	0.08	1	0.07	0.5	0.06	0.07	0.03	0.08	0.06	0.08	0.5	1.4	57.23	59.45	− 2.22
234	12	1.2	0.06	0.14	0.08	1	0.03	1	0.06	0.07	0.03	0.03	0.04	0.08	0.5	1.8	88.24	89.14	− 0.9002
235	10	1	0.1	0.1	0.055	0.7	0.05	1.25	0.1	0.05	0.05	0.055	0.05	0.07	0.75	1.6	86.56	84.86	1.70
236	8	0.8	0.06	0.14	0.03	1	0.07	0.5	0.14	0.03	0.03	0.08	0.04	0.08	0.5	1.4	92.34	92.50	− 0.1649
237	12	0.8	0.14	0.14	0.08	1	0.03	0.5	0.06	0.07	0.07	0.08	0.04	0.08	1	2.1	58.74	58.69	0.0516
238	12	1.2	0.14	0.06	0.08	1	0.07	1	0.06	0.07	0.03	0.03	0.04	0.06	1	1.8	86.40	82.27	4.13
239	12	0.8	0.06	0.14	0.08	1	0.03	1	0.14	0.03	0.07	0.08	0.04	0.08	0.5	1.9	58.74	59.24	− 0.4975
240	8	1.2	0.14	0.14	0.08	0.4	0.03	0.5	0.06	0.03	0.03	0.03	0.06	0.06	1	1.7	67.10	60.56	6.54
241	12	1.2	0.14	0.06	0.08	0.4	0.03	0.5	0.06	0.07	0.03	0.03	0.04	0.06	0.5	2.1	56.24	55.06	1.18
242	8	1.2	0.06	0.06	0.03	1	0.03	1	0.14	0.03	0.07	0.03	0.04	0.06	0.5	2.0	62.23	56.88	5.35
243	8	1.2	0.06	0.06	0.08	1	0.03	0.5	0.06	0.03	0.03	0.08	0.04	0.06	0.5	2.0	60.47	62.58	− 2.11
244	12	1.2	0.06	0.06	0.03	1	0.07	0.5	0.06	0.03	0.07	0.03	0.06	0.08	1	1.8	58.24	57.35	1.05
245	12	0.8	0.14	0.06	0.08	1	0.07	0.5	0.14	0.03	0.07	0.03	0.06	0.08	0.5	1.9	88.78	92.50	− 3.72
246	12	0.8	0.06	0.14	0.03	0.4	0.03	0.5	0.06	0.07	0.07	0.08	0.06	0.08	1	1.8	84.52	84.83	− 0.5249
247	12	0.8	0.06	0.14	0.08	1	0.07	0.5	0.14	0.07	0.03	0.03	0.06	0.08	0.5	1.6	76.85	68.99	7.86
248	8	1.2	0.14	0.06	0.08	1	0.07	1	0.14	0.03	0.07	0.08	0.04	0.08	0.5	1.9	88.52	87.21	1.31
249	8	1.2	0.06	0.14	0.08	0.4	0.07	1	0.14	0.03	0.07	0.03	0.06	0.08	1	1.5	91.32	89.94	1.38
250	8	0.8	0.14	0.14	0.03	1	0.07	0.5	0.06	0.07	0.03	0.03	0.06	0.06	0.5	2.0	68.74	67.70	1.04
251	10	1	0.1	0.1	0.055	0.7	0.05	0.25	0.1	0.05	0.05	0.055	0.05	0.07	0.75	2.0	84.41	85.85	− 1.54
252	12	1.2	0.14	0.14	0.03	0.4	0.03	1	0.06	0.07	0.03	0.03	0.06	0.08	0.5	1.4	68.74	65.73	3.01
253	10	1	0.1	0.1	0.055	0.7	0.05	0.75	0.1	0.05	0.05	0.055	0.05	0.07	0.75	1.6	92.45	91.71	1.24
254	8	1.2	0.14	0.06	0.03	0.4	0.03	1	0.06	0.03	0.03	0.03	0.04	0.08	1	1.8	67.45	59.89	7.56
255	8	1.2	0.14	0.06	0.03	0.4	0.07	0.5	0.06	0.07	0.07	0.08	0.06	0.08	1	1.8	70.32	64.53	5.59
256	10	1	0.1	0.1	0.055	0.7	0.05	0.75	0.1	0.05	0.05	0.055	0.05	0.07	0.75	1.9	86.45	90.45	− 4.00
257	10	1	0.1	0.1	0.055	0.7	0.05	0.75	0.1	0.05	0.05	0.005	0.05	0.07	0.75	2.0	88.52	88.43	0.0867
258	12	1.2	0.14	0.14	0.08	1	0.03	1	0.14	0.03	0.03	0.08	0.06	0.06	0.5	2.0	60.47	61.42	− 0.9468
259	8	1.2	0.14	0.14	0.03	1	0.07	0.5	0.14	0.03	0.07	0.08	0.06	0.06	0.5	2.0	88.78	91.69	− 2.91
260	12	1.2	0.14	0.06	0.08	0.4	0.07	1	0.06	0.03	0.07	0.08	0.06	0.06	0.5	1.9	59.99	57.68	2.31
261	8	0.8	0.14	0.06	0.08	0.4	0.03	0.5	0.06	0.07	0.03	0.03	0.04	0.08	1	1.8	88.78	79.04	− 0.2637
262	8	0.8	0.06	0.06	0.03	0.4	0.07	1	0.06	0.07	0.03	0.03	0.06	0.08	0.5	1.9	88.12	88.09	− 0.0189
263	12	0.8	0.06	0.14	0.08	0.4	0.07	1	0.14	0.03	0.07	0.03	0.06	0.06	0.5	1.7	54.00	62.13	− 6.23
264	12	0.8	0.06	0.06	0.03	1	0.03	0.5	0.14	0.03	0.07	0.08	0.06	0.06	0.5	1.7	56.37	57.75	− 1.38
265	8	0.8	0.14	0.06	0.08	1	0.03	0.5	0.06	0.03	0.07	0.08	0.06	0.08	0.5	1.2	60.70	57.42	3.28
266	8	0.8	0.14	0.14	0.08	1	0.07	1	0.14	0.07	0.07	0.08	0.06	0.06	0.5	1.8	58.74	61.91	− 2.27
267	14	1	0.1	0.1	0.055	0.7	0.05	0.75	0.1	0.05	0.05	0.055	0.05	0.07	0.75	1.8	86.37	89.10	− 2.73
268	12	1.2	0.06	0.14	0.08	0.4	0.07	1	0.06	0.07	0.03	0.08	0.06	0.06	0.5	1.7	88.24	86.56	− 0.3431
269	8	1.2	0.06	0.14	0.08	1	0.07	0.5	0.14	0.07	0.03	0.03	0.06	0.06	1	1.8	87.25	87.15	0.2031
270	10	1	0.1	0.1	0.055	0.1	0.05	0.75	0.1	0.05	0.05	0.055	0.05	0.07	0.75	1.7	82.45	83.48	− 1.03
271	12	1.2	0.06	0.14	0.03	1	0.07	0.5	0.14	0.03	0.03	0.08	0.04	0.06	1	1.8	68.45	64.17	4.10
272	8	1.2	0.06	0.06	0.03	0.4	0.07	0.5	0.14	0.03	0.07	0.03	0.04	0.06	1	1.7	87.45	93.49	− 6.04
273	12	0.8	0.14	0.14	0.08	0.4	0.03	1	0.06	0.03	0.03	0.08	0.04	0.06	1	2.0	56.34	55.30	1.04
274	10	1	0.1	0.18	0.055	0.7	0.05	0.75	0.1	0.05	0.05	0.055	0.05	0.07	0.75	2.1	88.07	88.42	− 0.3473
275	8	0.8	0.14	0.06	0.08	1	0.07	1	0.06	0.07	0.03	0.03	0.04	0.08	0.5	1.9	58.75	63.70	− 4.95
276	8	1.2	0.14	0.06	0.03	1	0.07	1	0.06	0.03	0.03	0.08	0.06	0.06	1	1.7	64.24	56.74	− 3.50
277	8	0.8	0.06	0.14	0.08	0.4	0.07	0.5	0.06	0.07	0.03	0.03	0.04	0.06	0.5	2.1	88.66	86.65	1.01
278	8	0.8	0.06	0.14	0.03	0.4	0.03	0.5	0.14	0.03	0.03	0.03	0.06	0.06	0.5	1.8	84.22	85.14	3.00
279	12	0.8	0.06	0.14	0.08	1	0.03	0.5	0.14	0.03	0.07	0.03	0.06	0.06	1	2.0	90.16	92.20	− 2.98
280	8	0.8	0.06	0.06	0.08	1	0.03	1	0.14	0.07	0.07	0.08	0.06	0.08	1	1.9	43.47	58.66	2.61
281	8	1.2	0.14	0.06	0.08	0.4	0.07	0.5	0.14	0.07	0.03	0.08	0.04	0.06	0.5	1.8	83.28	84.91	1.40
282	8	1.2	0.06	0.14	0.03	0.4	0.07	0.5	0.06	0.03	0.03	0.08	0.06	0.08	1	1.7	84.44	90.52	− 1.84
283	12	0.8	0.14	0.14	0.03	0.4	0.07	1	0.14	0.07	0.03	0.08	0.06	0.06	1	1.8	61.23	64.76	3.47
284	12	1.2	0.14	0.14	0.03	0.4	0.07	1	0.06	0.03	0.07	0.03	0.06	0.06	1	1.6	84.58	85.26	− 0.8240
285	8	1.2	0.14	0.06	0.08	1	0.03	0.5	0.14	0.07	0.03	0.03	0.06	0.08	0.5	1.7	86.59	85.57	− 0.3333
286	8	1.2	0.06	0.14	0.08	0.4	0.03	1	0.14	0.07	0.03	0.03	0.06	0.06	0.5	1.8	63.87	62.06	− 0.6414
287	8	1.2	0.14	0.14	0.08	0.4	0.07	1	0.06	0.07	0.07	0.08	0.04	0.06	1	1.8	63.52	63.49	− 0.5547
288	12	1.2	0.06	0.14	0.03	0.4	0.07	0.5	0.14	0.07	0.07	0.03	0.06	0.06	0.5	1.5	84.78	82.70	6.08
289	12	0.8	0.14	0.14	0.08	0.4	0.07	1	0.06	0.07	0.07	0.08	0.04	0.08	0.5	1.9	78.11	87.45	− 1.34
290	12	0.8	0.06	0.14	0.03	1	0.07	0.5	0.06	0.07	0.07	0.03	0.04	0.06	1	1.9	86.19	92.40	− 4.08
291	8	0.8	0.06	0.06	0.08	1	0.07	1	0.14	0.03	0.03	0.08	0.06	0.06	0.5	1.8	84.16	88.18	− 4.92
292	12	1.2	0.06	0.06	0.03	1	0.03	1	0.06	0.07	0.03	0.08	0.04	0.08	1	1.9	68.64	56.80	− 0.1395
293	8	0.8	0.14	0.14	0.03	0.4	0.07	1	0.06	0.03	0.07	0.03	0.06	0.08	0.5	1.8	61.55	52.32	0.0366
294	12	1.2	0.14	0.06	0.03	1	0.03	1	0.14	0.03	0.03	0.03	0.06	0.06	1	1.4	78.54	81.57	2.34
295	8	1.2	0.06	0.14	0.08	1	0.03	1	0.14	0.03	0.07	0.08	0.04	0.06	1	1.7	91.71	89.54	1.66
296	12	1.2	0.06	0.06	0.08	0.4	0.03	1	0.14	0.03	0.03	0.03	0.04	0.06	1	2.0	62.56	61.42	0.3541
297	8	1.2	0.06	0.06	0.08	1	0.07	0.5	0.06	0.07	0.07	0.08	0.04	0.08	1	1.7	86.41	84.74	0.5312
298	8	0.8	0.14	0.14	0.08	0.4	0.07	0.5	0.14	0.03	0.03	0.08	0.06	0.08	0.5	1.8	62.42	62.32	1.27

### Instrumental analysis

#### FT-IR analysis

FT-IR spectroscopy analyses the electromagnetic radiation absorbed by the sample. The yeast biomass of both before and after optimization was centrifuged (10000 rpm, 10 min, + 4 °C, Cooling Centrifuge, Remi c24BL) and separated from the broth culture. The cell pellets of the biomass of both biosorbents were washed thrice with deionized water to remove the growth media residuals. The biomass was lyophilized and both were then subjected to FT-IR spectroscopy at the wavelength range of 400–4000 cm^−1^ using FT-IR spectrophotometer (Perkin Elmer, Spectrum 100) equipped with beam splitter (KBr) and DTGS (deuterated triglycine sulphate) detector.

#### Scanning electron microscopic and EDAX analyses

The Cd(II) treated strains before and after optimization were subjected for another scanning electron microscopic study after treating the resistant strain with Cd(II). The dried biomass of the strains were treated with gluteraldehyde (Sigma-Aldrich) and dehydrated by ethanol treatment (30–100%)^43^. The samples were then observed under the Scanning Electron Microscope (QUANTA FEG 250) after being sputter coated by platinum. Energy dispersive X-ray (EDAX) analyses were carried out for the biosorbents to elucidate the elemental composition of the samples. The analysis was carried out by using EDAX microanalyzer (ELEMENT EDAX) conjugated with the Scanning Electron Microscope (SEM) (QUANTA FEG 250).

### Desorption experiment

After carrying Cd(II) adsorption studies it was necessary to investigate the desorption capacity and the reusability of the biosorbent. The biomass (0.15 g) was separated from the adsorbing solution and washed three times with deionized water. It was then re-suspended in the eluent solution and agitated for 2.5 h. Cd(II) concentration in the liquid phase was measured by Atomic Absorption Spectroscopy (Shimadzu AA-7000, Japan)^17^. The eluent used in the assay was 0.2(M) HCl^44^. The desorption efficiency (η %) was calculated from the following equation:

12 $${\text{M}}_{{{\text{desorbed}}}} /{\text{M}}_{{{\text{sorbed}}}} \times {1}00\%$$

13 $${\text{C}}_{{\text{r}}} \times {\text{V}}_{{\text{r}}} /\left( {{\text{C}}_{{\text{i}}} - {\text{C}}_{{\text{e}}} } \right){\text{V}} \times {1}00$$

M_desorbed_ represents amount of Cd(II) desorbed (mg/g) and M_sorbed_ (mg/g) as amount adsorbed with the biomass. The terms V_r_ and C_r_ represents desorption volume (L) and concentration of Cd(II) in the desorption solution.

## Results and discussion

### Optimization studies and selection of synthetic media

#### Central composite design (CCD) and statistical analysis

Response surface methodology (RSM) was successfully applied to identify the significant parameters influenced Cd(II) removal and to demonstrate the optimum conditions favoring maximal biosorption capacity by the strain *Candida tropicalis* XTA1874. The quadratic regression model as a function of Glucose concentration (A), Urea concentration (B), K_2_HPO_4_ concentration (C), KH_2_PO_4_ concentration (D), MgSO_4_⋅7H_2_O concentration (E), KCl concentration (F), CoCl_2_⋅6H_2_O (G), NH_4_VO_2_ (H), Na_2_MoO_4_⋅2H_2_O (J), CaCO_3_ (K), FeSO_4_⋅7H_2_O (L), ZnSO_4_⋅7H_2_O (M), MnSO_4_⋅4H_2_O (N), NiSO_4_.7H_2_O (O), Na_2_B_4_O_7_⋅10H_2_O (P) and Dry Cell Weight (Q) are presented in Tables 1–3 and Supplementary Tables 1–3). The F and *p* value are considered to be important in determining the significance of each of the variables. The Model F-value of 21.68 implies the model is significant. There is only a 0.01% chance that an F-value this large could occur due to noise. It has been confirmed by the regression analysis the linear model term (Q), the interactive model terms (AK), (BF), (BG), (CO), (DL), (EH), (FH), (LN), (LQ) and the quadratic terms (F^2^) and (Q^2^) were significant (*p* < 0.05) (Table 3, Supplementary Table 3). The estimation of the quadratic model design matrix was done by using *p*-values. The values of the lack of fit for Cd(II) biosorption was found to be not significant (*p* > 0.05). The lack of fit F-value 2.10 implies the Lack of Fit is not significant relative to the pure error. The estimation of F value is carried out by dividing model mean square by residual mean square comparing the model variance and residual^45^. The coefficient of variance (CV) of 6.17% ascertains the reliability and precision of experimental data. Moreover, the insignificant lack of fit and high determination coefficient (R^2^ = 0.9329) which agrees well with the adjusted R^2^ (*Adj*R^2^ = 0.9134) imply the validity and fitness of the model. The adequate precision of 17.5463 (> 4) shows the signal to noise ratio comparing the predicted values at the design points to the average prediction error (Table 4, Supplementary Table 4)^35,45^.

**Table 3:** Regression analysis using central composite design (CCD).

Source	Sum of squares	df	Mean square	F-value	*p*-value	
Model	65,475.61	141	439.20	21.68	< 0.0001	Significant
A-Glucose concentration	3.45	1	3.45	0.1458	0.6244	
B-Urea concentration	1.01	1	1.01	0.0248	0.8234	
C-K_2_HPO_4_ concentration	24.11	1	24.11	1.11	0.2929	
D-KH_2_PO_4_ concentration	13.45	1	13.45	0.6227	0.4376	
E-MgSO_4_⋅7H_2_O concentration	24.09	1	24.09	1.02	0.3155	
F-KCl concentration	54.14	1	54.14	2.61	0.1074	
G-CoCl_2_⋅6H_2_O concentration	51.97	1	51.97	2.12	0.1524	
H-NH_4_VO_2_ concentration	16.34	1	16.34	0.7578	0.3578	
J-Na_2_MoO_4_⋅2H_2_O concentration	3.72	1	3.72	0.1694	0.6182	
K-CaCO_3_ concentration	52.56	1	52.56	2.34	0.1165	
L-FeSO_4_⋅7H_2_O concentration	6.34	1	6.34	0.2870	0.5764	
M-ZnSO_4_⋅7H_2_O concentration	0.2577	1	0.2577	0.0348	0.9577	
N-MnSO_4_⋅4H_2_O concentration	16.21	1	16.21	0.7662	0.3768	
O-NiSO_4_⋅7H_2_O concentration	34.54	1	34.54	1.58	0.2255	
P-Na_2_B_4_O_7_⋅10H_2_O concentration	10.24	1	10.24	0.4454	0.4178	
Q-Dry cell weight	24,370.69	1	24,370.69	1146.34	< 0.0001	
AB	0.0514	1	0.0514	0.0025	0.9584	
AC	24.30	1	24.30	1.26	0.2621	
AD	10.24	1	10.2	0.4475	0.4902	
AE	0.0000	1	0.0000	6.489E-07	0.9927	
AF	5.27	1	5.27	0.2483	0.6247	
AG	21.24	1	21.24	0.9270	0.3214	
AH	0.0541	1	0.0541	0.0054	0.9623	
AJ	23.34	1	23.34	1.01	0.2578	
AK	84.14	1	84.14	3.71	0.0178	
AL	6.22	1	6.22	0.2548	0.5247	
AM	2.47	1	2.47	0.1528	0.7332	
AN	1.88	1	1.88	0.0125	0.7854	
AO	54.12	1	54.12	2.50	0.1657	
AP	4.14	1	4.14	0.2278	0.6256	
AQ	4.33	1	4.33	0.2809	0.6474	
BC	2.47	1	2.47	0.1474	0.7253	
BD	2.04	1	2.04	0.0644	0.7786	
BE	45.24	1	45. 27	2.12	0.1379	
BF	99.20	1	99.20	4.61	0.0447	
BG	137.65	1	137.65	5.11	0.0477	
BH	34.28	1	34.28	1.84	0.1783	
BJ	24.59	1	24.59	1.13	0.2856	
BK	48.52	1	48.52	2.24	0.1322	
BL	2.64	1	2.64	0.1474	0.7437	
BM	83.12	1	83.12	3.81	0.0547	
BN	0.2843	1	0.2843	0.0248	0.9068	
BO	1.25	1	1.25	0.0545	0.7657	
BP	0.0540	1	0.0540	0.0249	0.9624	
BQ	0.0324	1	0.0324	0.0175	0.9654	
CD	15.11	1	15.11	0.7074	0.4066	
CE	23.26	1	23.26	1.14	0.2247	
CF	0.0523	1	0.0523	0.0878	0.9327	
CG	20.07	1	20.07	0.9270	0.3548	
CH	9.71	1	9.71	0.4746	0.5876	
CJ	29.44	1	29.44	1.30	0.2749	
CK	33.52	1	33.52	1.52	0.2223	
CL	10.74	1	10.74	0.4717	0.4669	
CM	24.23	1	24.23	1.11	0.2540	
CN	31.32	1	31.32	1.47	0.2259	
CO	111.06	1	111.06	4.19	0.0371	
CP	4.25	1	4.25	0.2228	0.6874	
CQ	0.0456	1	0.0456	0.0175	0.9450	
DE	7.54	1	7.54	0.3342	0.5568	
DF	16.22	1	16.22	0.7454	0.3454	
DG	10.26	1	10.26	0.4245	0.4782	
DH	4.16	1	4.16	0.2124	0.6574	
DJ	4.52	1	4.52	0.2041	0.6255	
DK	0.0074	1	0.0074	0.0012	0.9315	
DL	166.52	1	166.52	7.32	0.0063	
DM	2.17	1	2.17	0.1542	0.7524	
DN	66.45	1	66.45	3.05	0.0874	
DO	10.52	1	10.52	0.4744	0.4743	
DP	25.44	1	25.44	1.19	0.2445	
DQ	1.50	1	1.50	0.0241	0.8056	
EF	1.31	1	1.31	0.0120	0.8136	
EG	24.34	1	24.34	1.31	0.2524	
EH	142.35	1	142.35	7.45	0.0067	
EJ	0.6451	1	0.6451	0.0174	0.8665	
EK	10.54	1	10.54	0.4252	0.4559	
EL	6.33	1	6.33	0.3749	0.5885	
EM	22.24	1	22.24	1.05	0.3338	
EN	1.37	1	1.37	0.0644	0.8245	
EO	48.52	1	48.52	2.26	0.1349	
EP	8.47	1	8.47	0.3794	0.5389	
EQ	3.45	1	3.45	0.1487	0.7004	
FG	17.73	1	17.73	0.7550	0.3876	
FH	123.42	1	123.42	6.12	0.0046	
FJ	12.21	1	12.21	0.5163	0.4245	
FK	14.73	1	14.73	0.5173	0.4331	
FL	37.75	1	37.75	1.65	0.1573	
FM	3.64	1	3.64	0.1553	0.6776	
FN	2.25	1	2.25	0.1379	0.7245	
FO	67.79	1	67.79	3.23	0.0538	
FP	24.44	1	24.44	1.13	0.2526	
FQ	11.41	1	11.41	0.5748	0.4656	
GH	0.8454	1	0.8454	0.0385	0.8464	
GJ	75.22	1	75.22	3.47	0.0647	
GK	0.6786	1	0.6786	0.0234	0.8537	
GL	0.0055	1	0.0055	0.0345	0.9822	
GM	48.37	1	48.37	2.22	0.1387	
GN	1.34	1	1.34	0.0576	0.8174	
GO	3.48	1	3.48	0.1442	0.6934	
GP	0.4645	1	0.4645	0.0575	0.8855	
GQ	6.23	1	6.23	0.2550	0.5967	
HJ	1.81	1	1.81	0.0258	0.7547	
HK	43.24	1	43.24	2.00	0.1554	
HL	0.7271	1	0.7271	0.0585	0.8574	
HM	42.87	1	42.87	1.68	0.1632	
HN	0.6534	1	0.6534	0.0212	0.8619	
HO	0.4540	1	0.4540	0.0274	0.8822	
HP	0.4474	1	0.4474	0.0549	0.8818	
HQ	19.32	1	19.32	0.8548	0.3420	
JK	26.74	1	26.74	1.47	0.2616	
JL	28.54	1	28.54	1.37	0.2519	
JM	3.62	1	3.62	0.1555	0.6952	
JN	25.54	1	25.54	1.17	0.2843	
JO	13.31	1	13.31	0.6334	0.4567	
JP	20.74	1	20.74	0.9322	0.3454	
JQ	11.52	1	11.52	0.5877	0.4574	
KL	0.4475	1	0.4475	1.78	0.8339	
KM	3.27	1	3.27	0.1433	0.7551	
KN	31.52	1	31.52	1.48	0.2520	
KO	5.51	1	5.51	0.2547	0.6672	
KP	1.64	1	1.64	0.0555	0.7442	
KQ	63.72	1	63.72	2.12	0.0815	
LM	34.34	1	34.34	1.54	0.2045	
LN	106.74	1	106.74	4.77	0.0027	
LO	2.54	1	2.54	0.1745	0.7228	
LP	14.28	1	14.28	0.6972	0.4117	
LQ	168.41	1	168.41	7.32	0.0022	
MN	1.52	1	1.52	0.0288	0.8342	
MO	3.64	1	3.64	0.1259	0.6573	
MP	11.52	1	11.52	0.5424	0.4664	
MQ	0.4234	1	0.4234	0.0153	0.8859	
NO	4.37	1	4.37	0.2064	0.6542	
NP	18.52	1	18.52	0.8487	0.3657	
NQ	25.21	1	25.21	1.54	0.2782	
OP	43.47	1	43.47	2.33	0.1245	
OQ	10.21	1	10.21	0.4828	0.4372	
PQ	19.47	1	19.47	0.8955	0.3568	
A^2^	7.15	1	7.15	0.3447	0.5589	
B^2^	53.27	1	53.27	2.86	0.1220	
C^2^	0.0414	1	0.0414	0.0023	0.9661	
D^2^	22.15	1	22.15	1.13	0.3152	
E^2^	21.15	1	21.15	0.9317	0.3367	
F^2^	98.32	1	98.32	4.44	0.0378	
G^2^	4.35	1	4.35	0.1541	0.6542	
H^2^	72.64	1	72.64	3.26	0.0369	
J^2^	8.24	1	8.24	0.3448	0.5544	
K^2^	1.34	1	1.34	0.0725	0.7789	
L^2^	72.55	1	72.55	3.18	0.0482	
M^2^	15.64	1	15.64	0.7144	0.3666	
N^2^	17.72	1	17.72	0.8231	0.3794	
O^2^	1.82	1	1.82	0.0541	0.7453	
P^2^	4.32	1	4.32	0.2521	0.6526	
Q^2^	1789.33	1	1789.33	87.19	< 0.0001	
Residual	3134.12	213	21.67			
Lack of fit	3374.32	126	22.40	2.10	0.1107	Not significant
Pure error	94.79	9	10.74			
Cor total	72,477.71	527

**Table 4:** Fit statistics.

Std. dev.	4.45	R^2^	0.9329
Mean	75.46	Adjusted R^2^	0.9134
C.V.%	6.17	Predicted R^2^	0.8277
		Adeq precision	17.5463

**Figure 1:**
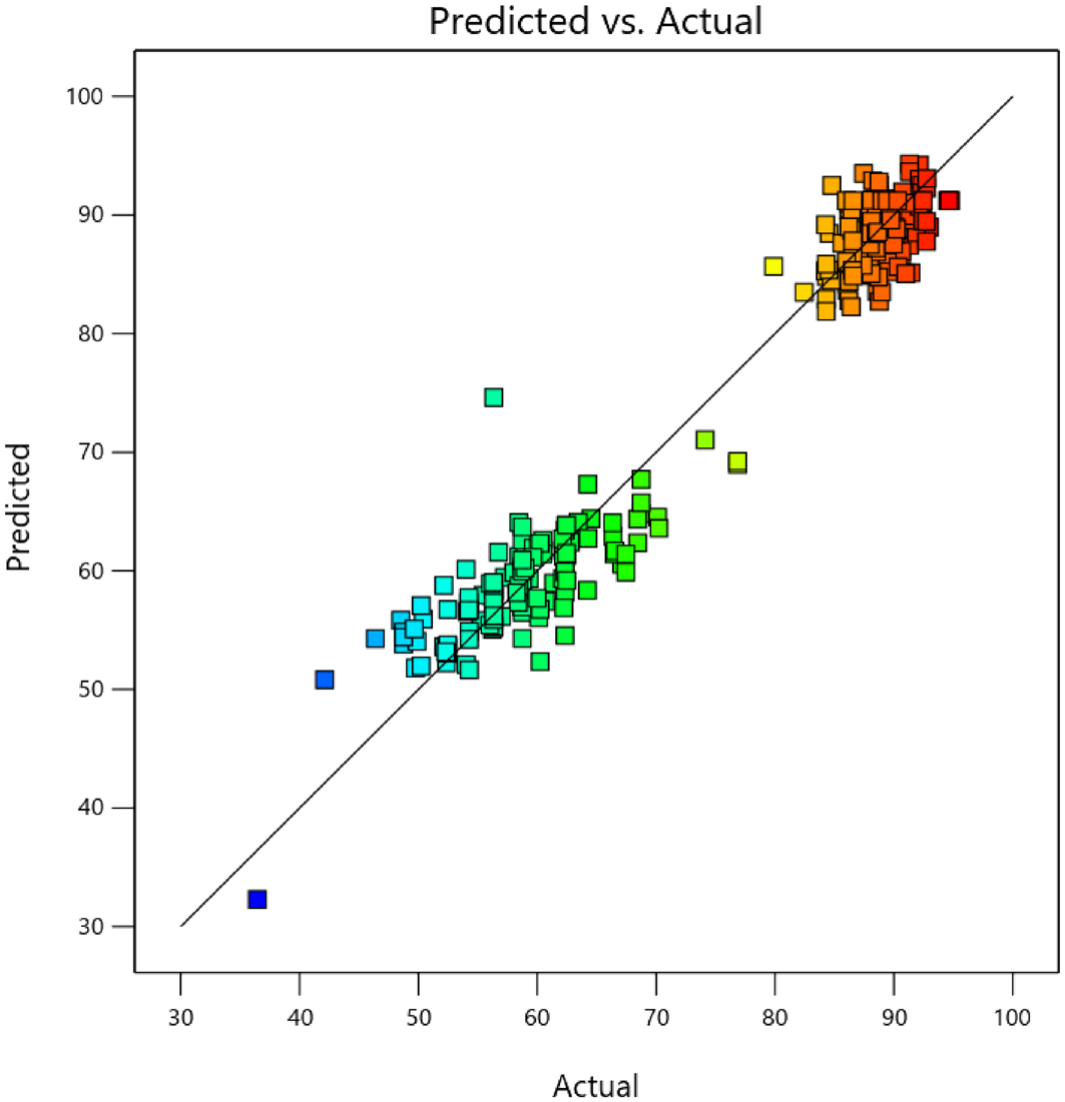
Comparison of predicted vs. actual values for Cd(II) biosorption by *Candida tropicalis* XTA1874.

The significant model terms for Cd(II) removal were (Q), (AK), (BF), (BG), (CO), (DL), (EH), (FH), (LN), (LQ), (F^2^) and (Q^2^) indicating having significant effect on Cd(II) biosorption by the strain. The ANOVA analysis resulted in a standard deviation of 4.45 and a mean of 75.46 (Table 4, Supplementary Table 4). Table 2, and Supplementary Tables 2 and 5 and the plot in Fig. 1 and Supplementary Fig. 1 both shows that the actual and predicted values are very close to each other and distribution of the data is close to the fitted line. This indicated that the experimental model is suitable in describing the experimental data. According to the analysis the small probability value of the model is confirm to reject the null hypothesis and the data followed a normal distribution. The equation obtained from the coefficient terms of the coded factors (Supplementary Table 6) for the response variable has been shown in Eq. (14).

14 $$\begin{aligned} {\text{Y}}_{{{\text{Cd}}({\text{II}})}} = & + {92}.{21} - 0.0{\text{166A}} - 0.0{67}0{\text{B}} - 0.{214}0{\text{C}} + 0.{23}0{\text{5D}} + 0.{\text{2862E}} + 0.{\text{4681F}} + 0.{\text{4171G}} - 0.{\text{2463H}} \\ & - 0.{\text{1254J}} + 0.{\text{2457K}} + 0.0{\text{551L}} + 0.0{\text{341M}} - 0.{\text{2467N}} + 0.{\text{2641O}} + 0.{2}0{\text{31P}} + {6}.{\text{44Q}} + 0.0{\text{174AB}} \\ & - 0.{\text{3247AC}} + 0.{\text{2122AD}} - 0.00{\text{12AE}} + 0.{\text{1475AF}} + 0.{\text{2919AG}} + 0.0{21}0{\text{AH}} + 0.{3}0{\text{37AJ}} - 0.{\text{5146AK}} \\ & + 0.{\text{1582AL}} + 0.0{\text{967AM}} - 0.0{8}0{\text{5AN}} + 0.{\text{3252AO}} + 0.{\text{1463AP}} - 0.{\text{1486AQ}} + 0.0{\text{954BC}} - 0.0{\text{921BD}} \\ & - 0.{\text{4272BE}} - 0.{63}0{\text{8BF}} - 0.{\text{6584BG}} + 0.{\text{3967BH}} - 0.{\text{3579BJ}} - 0.{\text{4425BK}} + 0.0{\text{957BL}} + 0.{\text{5762BM}} \\ & + 0.0{\text{387BN}} + 0.0{\text{862BO}} + 0.0{\text{217BP}} + 0.0{\text{134BQ}} + 0.{\text{2387CD}} - 0.{35}0{\text{3CE}} - 0.0{\text{255CF}} - 0.{\text{2873CG}} \\ & - 0.{\text{1971CH}} - 0.{\text{3352CJ}} + 0.{\text{3631CK}} + 0.{2}0{\text{28CL}} + 0.{\text{3124CM}} + 0.{\text{3513CN}} + 0.{\text{6517CO}} - 0.{\text{1213CP}} \\ & + 0.0{\text{316CQ}} + 0.{\text{1747DE}} + 0.{\text{2174DF}} - 0.{221}0{\text{DG}} + 0.{\text{1741DH}} + 0.{\text{1545DJ}} - 0.0{\text{429DK}} - 0.{\text{8475DL}} \\ & - 0.0{\text{936DM}} + 0.{\text{5272DN}} + 0.{\text{1999DO}} - 0.{\text{3281DP}} + 0.0{\text{724DQ}} - 0.0{\text{688EF}} - 0.{\text{3647EG}} + 0.{\text{7954EH}} \\ & - 0.0{\text{747EJ}} - 0.{\text{2284EK}} - 0.{\text{1657EL}} - 0.{\text{2984EM}} + 0.{\text{1733EN}} - 0.{\text{4574EO}} - 0.{\text{1727EP}} - 0.{\text{1322EQ}} \\ & - 0.{\text{2579FG}} - 0.{\text{7673FH}} + 0.{\text{2711FJ}} - 0.{\text{2251FK}} - 0.{4}0{\text{61FL}} - 0.{\text{1312FM}} + 0.{\text{1126FN}} - 0.{\text{5249FO}} \\ & - 0.{\text{3212FP}} - 0.{\text{2176FQ}} + 0.0{57}0{\text{GH}} + 0.{\text{5424GJ}} - 0.{\text{1676GK}} + 0.{\text{2242GL}} + 0.{\text{4548GM}} + 0.0{\text{687GN}} \\ & + 0.{\text{1227GO}} - 0.0{\text{197GP}} - 0.{\text{1461GQ}} - 0.0{78}0{\text{HJ}} + 0.{437}0{\text{HK}} - 0.0{\text{433HL}} - 0.{\text{4547HM}} - 0.0{\text{215HN}} \\ & + 0.0{\text{438HO}} + 0.0{43}0{\text{HP}} - 0.{\text{2756HQ}} - 0.{\text{3239JK}} + 0.{\text{3332JL}} - 0.{\text{1174JM}} - 0.{\text{3139JN}} + 0.{\text{2352JO}} - 0.{\text{2817JP}} \\ & + 0.{\text{2183JQ}} - 0.0{\text{419KL}} - 0.{11}0{\text{3KM}} - 0.{\text{3523KN}} - 0.{\text{1466KO}} - 0.0{\text{855KP}} - 0.{\text{4974KQ}} + 0.{\text{3666LM}} \\ & - 0.{\text{6487LN}}0.{1}0{\text{43LO}} + 0.{24}0{\text{3LP}} + 0.{812}0{\text{LQ}} - 0.0{\text{681MN}} - 0.{\text{1213MO}} - 0.{\text{2145MP}} - 0.0{4}0{\text{6MQ}} \\ & + 0.{\text{1311NO}} - 0.{\text{2741NP}} + 0.{\text{3216NQ}} - 0.{\text{4523OP}} + 0.{\text{2374OQ}} + 0.{\text{2746PQ}} - 0.{\text{4787 A}}^{2} - {1}.{\text{25 B}}^{2} \\ & + 0.0{\text{356 C}}^{2} - 0.{\text{8196 D}}^{2} - 0.{\text{7174 E}}^{2} - {1}.{7}0{\text{ F}}^{2} - 0.{\text{3159 G}}^{2} - {1}.{\text{37 H}}^{2} - 0.{\text{4721 J}}^{2} - 0.{\text{2244 K}}^{2} \\ & - {1}.{\text{37 L}}^{2} - 0.{\text{6819 M}}^{2} - 0.{\text{7174 N}}^{2} - 0.{\text{2186 O}}^{2} + 0.{\text{3627 P}}^{2} - {7}.{\text{26 Q}}^{2} . \\ \end{aligned}$$

#### Interaction effects of the variables on Cd(II) removal by the strain and selection of synthetic media

**Figure 2:**
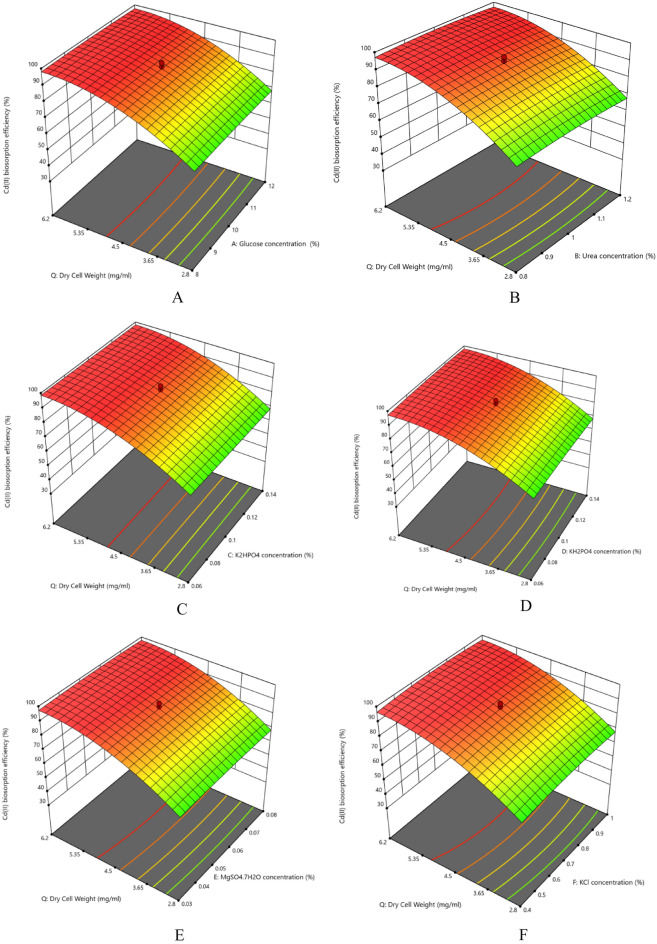

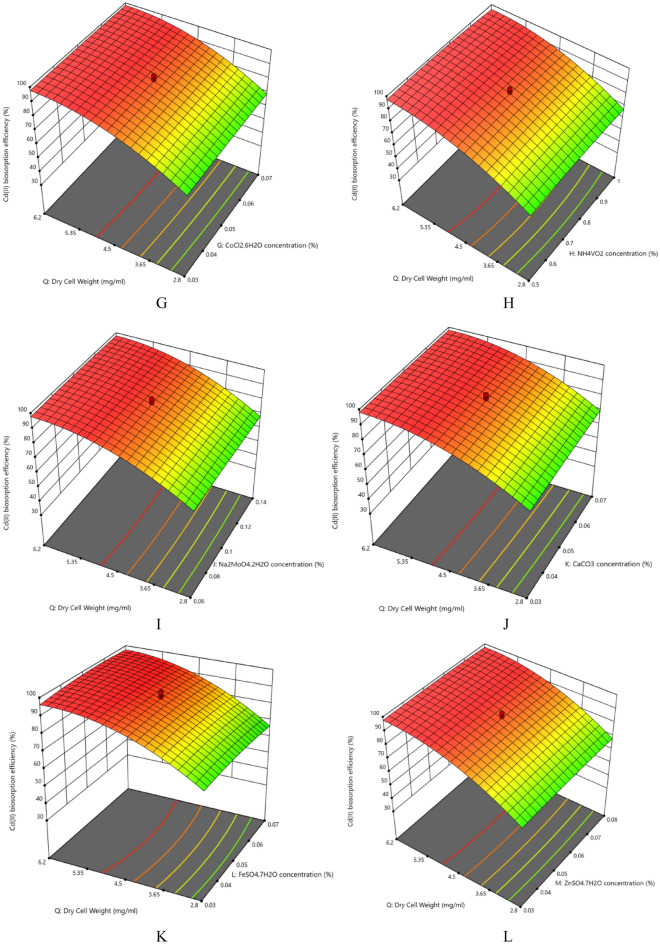

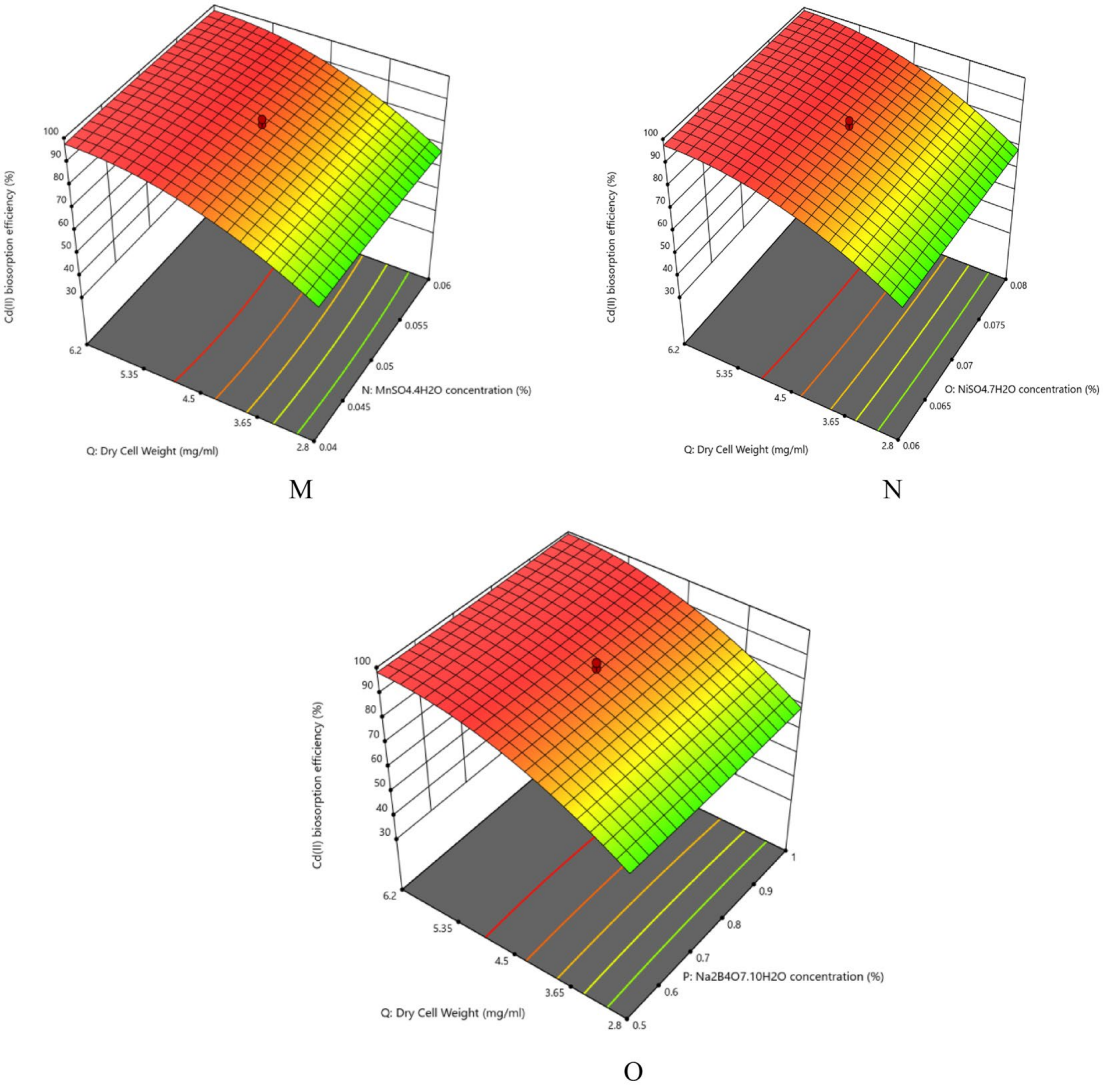
3D response surface plots for surface optimization of (**A**) glucose concentration (%), (**B**) urea concentration (%), (**C**) K_2_HPO_4_ concentration (%), (**D**) KH_2_PO_4_ concentration (%), (**E**) MgSO_4_⋅7H_2_O concentration (%), (**F**) KCl concentration (%), (**G**) CoCl_2_⋅6H_2_O concentration (%), (**H**) NH_4_VO_2_ concentration (%), (**I**) Na_2_MoO_4_ concentration (%), (**J**) CaCO_3_ concentration (%), (**K**) FeSO_4_⋅7H_2_O concentration (%), (**L**) ZnSO_4_⋅7H_2_O concentration (%), (**M**) MnSO_4_⋅7H_2_O concentration (%), (**N**) NiSO_4_⋅7H_2_O concentration (%), (**O**) Na_2_B_4_O_7_ concentration (%).

The nutrient composition plays significant roles in the growth and removal capacities by the organisms apart from the physical parameters. The interaction effects of glucose concentration and dry cell mass on Cd(II) biosorption efficiency (%) has been assessed in Fig. 2 and Supplementary Fig. 2. The contour plots show that the dry cell mass has much significant effect compared to the glucose concentration on Cd(II) biosorption efficiency (%) by the strain. According to the ANOVA results, the *p*-value is more than 0.05 proving the interaction effect on Cd(II) biosorption efficiency (%) to be statistically insignificant. The results showed maximum Cd(II) removal (95.972 ± 0.0001%) was achieved under the optimum nutrient concentrations: Glucose concentration (10.748%), Urea Concentration (1.071%), K_2_HPO_4_ concentration (0.127%), KH_2_PO_4_ concentration (0.122%), MgSO_4_⋅7H_2_O concentration (0.057%), KCl concentration (0.864%), CoCl_2_.5H_2_O concentration (0.027%), NH_4_VO_2_ concentration (0.757%), Na_2_MoO_4_⋅2H_2_O concentration (0.047%), CaCO_3_ concentration (0.054%), FeSO_4_⋅7H_2_O concentration (0.052%), ZnSO_4_⋅7H_2_O concentration (0.057%), MnSO_4_⋅4H_2_O concentration (0.047%), NiSO_4_.7H_2_O concentration (0.037%), Na_2_B_4_O_7_⋅10H_2_O (0.571%) and dry cell weight (1.532 mg/mL) (Table 5, Supplementary Tables 7, 8). The coded values have been calculated using Eq. (10). From the contour and 3D plots (Fig. 2, Supplementary Table 2) it is evident that Cd(II) biosorption efficiency (%) significantly increased with the increase with the increasing amount of both the carbon and nitrogen sources (Glucose and Urea respectively) along with increasing dry cell mass. Similar increase has also been witnessed with the increasing amounts of the trace elements used in the study. But in all cases the effect of dry cell mass is quite prominent. It suggests that the amount of dry cell mass has considerable impact on metal ion biosorption and it too has been found in our recent study. Regarding the ANOVA results, the interaction between Glucose concentration and CaCO_3_ concentration (AK), Urea concentration and KCl concentration (BF), Urea concentration and CoCl_2_.6H_2_O concentration (BG), K_2_HPO_4_ concentration and NiSO_4_.7H_2_O concentration (CO), KH_2_PO_4_ concentration  and FeSO_4_.7H_2_O concentration (DL), MgSO_4_.7H_2_O concentration and NH_4_VO_2_ concentration (EH), KCl concentration and NH_4_VO_2_ concentration (FH), FeSO_4_.7H_2_O concentration and MnSO_4_.4H_2_O concentration (LN), FeSO4.7H2O concentration and Na_2_B4O_7_.10H_2_O concentration (LQ) and the quadratic effects of KCl concentration (F^2^) and dry cell mass (Q^2^) on Cd(II) biosorption efficiency (%) was statistically significant having *p* value less than 0.05. *Candida tropicalis* CBL-1 strain has been reported to remove 70% Cd(II) in lab scale and maximum 60% from industrial wastewater^37^. The data showed that the most important constituents for organisimal growth and optimal biosorption are carbon (Glucose) and nitrogen (Urea) sources. Divalent ions such as Zn^2+^, Fe^2+^ and Ca^2+^ are mimicked by Cd^2+^ and sometimes competitively obstruct its adsorption^46,47^. They also have critical cellular roles and hence were required in trace amount for aggravating the growth of the strain. Potassium has intricate cellular roles in the yeast cells (Mackie and Brodsky 2018). It is concentrated in their cytosol as an electrogenic osmolyte and enzyme cofactor (Mackie and Brodsky 2018). In our experiment the quadratic term (F^2^) indicates the vitality of the potassium ion in the growth and hence removal efficiency of Cd(II) by the strain. Effects of nutrients along with physical parameters affecting biosorption have also been examined. In a biosorption study among eight independent variables including pH, incubation time (min), CuSO_4_⋅7H_2_O (%), Glucose (%), Glycerol (%), Peptone (%), K_2_HPO_4_ (%) and MgSO_4_⋅7H_2_O (%) all the nutrients contributed significantly except MgSO_4_⋅7H_2_O^48^.

**Table 5:** Optimum condition, experimental and predicted values of Cd(II) biosorption (%) at optimized conditions.

Optimum conditions	Coded levels	Actual levels
Glucose concentration (%)	0.239	10.748
Urea concentration (%)	0.355	1.071
K_2_HPO_4_ concentration (%)	0.675	0.127
KH_2_PO_4_ concentration (%)	0.55	0.122
MgSO_4_⋅7H_2_O concentration (%)	0.08	0.057
KCl concentration (%)	0.273	0.864
CoCl_2_⋅5H_2_O concentration (%)	− 0.57	0.027
NH_4_VO_2_ concentration (%)	0.014	0.757
Na_2_MoO_4_⋅2H_2_O concentration (%)	− 0.6	0.047
CaCO_3_ concentration (%)	0.1	0.054
FeSO_4_⋅7H_2_O concentration (%)	0.05	0.052
ZnSO_4_⋅7H_2_O concentration (%)	0.04	0.057
MnSO_4_⋅4H_2_O concentration (%)	0.35	0.047
NiSO_4_⋅7H_2_O concentration (%)	− 1.65	0.037
Na_2_B_4_O_7_⋅10H_2_O concentration (%)	− 0.35	0.571

Response	Predicted values	Experimental values
Cd(II) biosorption (%)	95.028	95.972 ± 0.0001

#### Validation of the model

Optimized conditions were maintained have been maintained for checking the suitability of the model for response value prediction. Optimized Cd(II) biosorption was validated under optimized experimental conditions. The response value at optimized nutritional conditions was 95.028%. On the other hand, experimental value under optimized conditions was 95.972 ± 0.0001% using 500 ppm of initial Cd(II) concentration. Experimental response value was well in agreement with the predicted response value (Table 5, Supplementary Table 8). Based on the above observation the synthetic media selected for optimum growth was shown in (Table 6, Supplementary Table 9).

**Table 6:** Selection of synthetic media.

Ingredients	Amount
Glucose concentration (%)	10.748
Urea concentration (%)	1.071
K_2_HPO_4_ concentration (%)	0.127
KH_2_PO_4_ concentration (%)	0.122
MgSO_4_⋅7H_2_O concentration (%)	0.057
KCl concentration (%)	0.864
CoCl_2_⋅5H_2_O concentration (%)	0.027
NH_4_VO_2_ concentration (%)	0.757
Na_2_MoO_4_⋅2H_2_O concentration (%)	0.047
CaCO_3_ concentration (%)	0.054
FeSO_4_⋅7H_2_O concentration (%)	0.052
ZnSO_4_⋅7H_2_O concentration (%)	0.057
MnSO_4_⋅4H_2_O concentration (%)	0.047
NiSO_4_⋅7H_2_O concentration (%)	0.037
Na_2_B_4_O_7_⋅10H_2_O concentration (%)	0.571

#### Biosorption kinetics

Biosorption kinetics determines the rate of adsorption of dissolved adsorbates on the surface of biological adsorbents. Thus kinetic analysis aids to determine the biosorbent’s ability to use as an effective Cd(II) adsorbent. Among the most profoundly used kinetic models described in the literature, those that uses order of chemical reactions are well considered. These models are the Pseudo First Order (Lagargren) and Pseudo Second Order (Mckay and Ho) kinetic models^41,49,50^.

**Figure 3:**
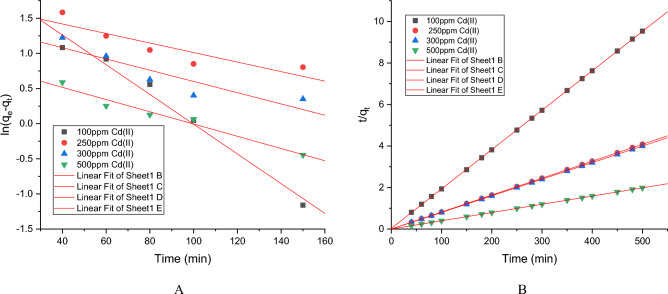
Linear plots for pseudo first (**A**) and second order (**B**) kinetic models for Cd(II) biosorption by the strain *Candida tropicalis* XTA1874 before optimization.

**Figure 4:**
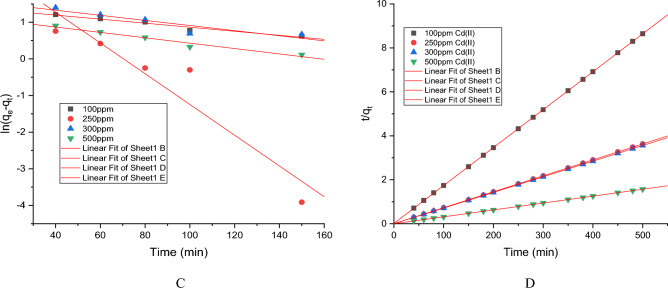
Linear plots for pseudo first (**C**) and second order (**D**) kinetic models for Cd(II) biosorption by the strain *Candida tropicalis* XTA1874 after optimization.

Cd(II) biosorption kinetics by the developed resistant strain *Candida tropicalis* XTA 1874 was performed in the before and after optimized conditions. Usually adsorption kinetics involves two phases: a rapid removal stage (first 60 min) from the aqueous solution followed by a slower removal stage before reaching the equilibrium (150 min) (Supplementary Tables 10–13, 15–18). The detailed kinetic analysis before and after optimization conditions has been described in the supplementary files (Supplementary Tables 10–13, 15–18) respectively. Intracellular accumulation of Cd(II) in the due course of removal has also been estimated during kinetic analysis and shown in the above mentioned tables. The kinetic data for Cd(II) biosorption by the strain before and after optimized conditions has been shown in (Tables 7, 8, Supplementary Tables 14, 19) along with the linear plots (Figs. 3, 4, Supplementary Figs. 3, 4). Considering the correlation coefficient obtained by linear plotting of pseudo first and second order equations it can be concluded that Cd(II) adsorption by the biomass of *Candida tropicalis* XTA1874 according to the pseudo second order model (R^2^ > 0.99) (Figs. 3, 4, Supplementary Figs. 3, 4). From the above observation it can be concluded that the rate limiting step of the Cd(II) biosorption was chemisorption^41,51^. The calculated q_e_ values obtained before and after optimization from the pseudo second order model were closer to that obtained by the experiment (Tables 7, 8, Supplementary Tables 14, 19). The rate constant k_2_ increased with increase in initial Cd(II) concentration indicating the presence of more than one mechanism influencing Cd(II) binding to the yeast cell biomass surface in the culture^41^.

**Table 7:** Values of the parameters of kinetic models for Cd(II) adsorption of *Candida tropicalis* XTA 1874 before optimization.

Metal ion (ppm)	q_e_,exp (mg/g)	Pseudo first order			Pseudo second order		
		k_1_	R^2^	q_e_,cal (mg/g)	k_2_	R^2^	q_e_,cal (mg/g)
100	52.864 ± 0.044	− 0.00014 ± 1.98E−06	0.978	8.227 ± 0.115	0.012 ± 0.001	0.999	53.102 ± 0.202
250	124.616 ± 0.002	− 0.00012 ± 2.4E−05	0.8	5.431 ± 0.306	0.018 ± 0.0004	1	122.784 ± 0.148
300	126. 415 ± 0.001	− 5.4E−05 ± 2.65E−07	0.817	4.074 ± 0.015	0.025 ± 0.002	1	125.157 ± 0.001
500	252.287 ± 0.006	− 5.8E−05 ± 2.77E−07	0.964	2.375 ± 0.002	0.028 ± 0.001	1	251.905 ± 0.010

**Table 8:** Values of the parameters of kinetic models for Cd(II) adsorption of *Candida tropicalis* XTA 1874 after optimization.

Metal ion (ppm)	q_e_,exp (mg/g)	Pseudo first order			Pseudo second order		
		k_1_	R^2^	q_e_,cal (mg/g)	k_2_	R^2^	q_e_,cal (mg/g)
100	59.708 ± 0.005	− 0.0001 ± 6.44E−05	0.952	4.138 ± 0.013	0.020 ± 0.0001	1	57.972 ± 0.038
250	137.684 ± 0.002	− 0.00031 ± 1.59E−05	0.892	19.307 ± 0.145	0.015 ± 3.07E−07	1	137.934 ± 0.003
300	142.415 ± 0.002	− 4.7E−05 ± 6.3E−07	0.842	5.089 ± 0.062	0.018 ± 0.0001	1	140.81 ± 0.120
500	319.622 ± 0.006	− 5E−05 ± 5.64E−07	0.963	3.203 ± 0.002	0.034 ± 0.0001	1	318.472 ± 0.0004

### Equilibrium biosorption isotherm modeling for Cd(II)

Adsorption isotherms define the equilibrium relations between adsorbate concentrations on the solid phase and its concentration in the liquid phase. Information about the maximum adsorption capacity can be obtained from the isotherms. These data provide information on the adsorbent capacity or the amount needed to remove a unit pollutant mass under the experimental conditions. The amount of Cd(II) adsorbed on the cell surface and intracellular accumulation have both been estimated in the before and after optimization conditions in the due course of Cd(II) removal from aqueous medium using 15–500 ppm of Cd(II) in aqueous medium. The values are shown in the supplementary files (Supplementary Tables 20, 22). Langmuir and Freundlich isotherms are most frequently used isotherms describing solid–liquid adsorption^52^.

**Figure 5:**
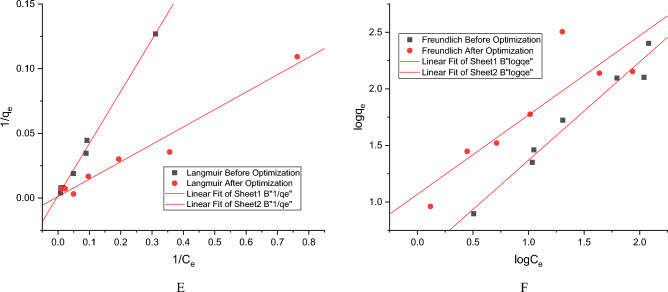
Linear plots for Langmuir (**E**) and Freundlich (**F**) isotherm model for Cd(II) biosorption by the strain *Candida tropicalis* XTA1874 before and after optimization.

The mathematical analysis of Cd(II) sorption at equilibrium by the strain can be best described by the Langmuir equation. According to the Langmuir’s theory adsorption occurs at homogeneous sites on the adsorbent surface by monolayer sorption^53^. After analyzing the data presented in (Tables 9, 10, Supplementary Tables 21, 22) it can be concluded that both Langmuir and Freundlich models can describe the experimental data. The linear plots of Langmuir and Freundlich model both before and after optimization are depicted in Fig. 5 and Supplementary Fig. 5. The higher R^2^ values obtained from the Langmuir model better describes the relationship between the amount of Cd(II) sorption at equilibrium. The values of the separation factor R_L_ lies between 0 and 1 (Tables 9, 10, Supplementary Table 21, 22) describing favorable adsorption both before and after optimization. The maximum Cd(II) adsorption capacity (q_max_) has been increased significantly after optimization which was 885.686 ± 0.26 mg/g (Tables 9, 10, Supplementary Tables 21, 22). The higher value of K_F_ (11.721 ± 0.002) after optimization indicates increase in the affinity of the adsorbent towards the toxicant after optimization. The parameter ‘n’ from Freundlich isotherm indicates the intensity of Cd(II) adsorption. The values (Tables 9, 10, Supplementary Tables 21, 22) lies between in the range 1 < n < 10, confirms the efficiency of the adsorption process. The inverse parameter (1/n), an irrational fraction, informs us about the degree of diversity of the adsorption sites. Its values lies between 0 < 1/n < 1 confirms significant homogeneity of the yeast cell surface^41^. The strain showed mean Cd(II) removal of 88.077 ± 0.097% which is significantly higher than the mean Cd(II) removal before optimized conditions 75.007 ± 0.002% (Tables 9, 10, Supplementary Tables 21, 22). From the statistical analysis using Student’s T-test (paired two tail) [sample size (n) = 6] (Table 11, Supplementary Table 24) it can be seen that there are significant differences in both the maximal surface adsorption capacity (q_max_) and mean removal (%) (*p* < 0.05) before and after optimizing conditions.

**Table 9:** Values of the parameters of isotherm models for Cd(II) biosorption equilibrium of *Candida tropicalis* XTA 1874 before optimization.

	q_max_ (mg/g)	K_L_ (L/mg)	R^2^	R_L_	Mean removal (%)
Langmuir	544.22 ± 0.25	1.155 ± 0.273	0.995	0.308 ± 0.005–0.935 ± 0.002	75.007 ± 0.002

	K_F_ (mg/g)	N	R^2^		
Freundlich	3.182 ± 0.016	1.143 ± 0.007	0.941

**Table 10:** Values of the parameters of isotherm models for Cd(II) biosorption equilibrium of *Candida tropicalis* XTA 1874 after optimization.

Langmuir	q_max_ (mg/g)	K_L_ (L/mg)	R^2^	R_L_	Mean removal (%)
	885.686 ± 0.26	1.612 ± 0.478	0.966	0.205 ± 0.009–0.881 ± 0.028	88.077 ± 0.097

	K_F_ (mg/g)	n	R^2^		
Freundlich	11.721 ± 0.002	1.433 ± 0.004	0.759

**Table 11:** Statistical analysis of the significance of the before and after optimization model by student’s T-test (n = 6).

Before optimization (q_max_)	After optimization (q_max_)	T-test	Significance at (*p* ≤ 0.05)	Before optimization mean removal (%)	After optimization mean removal (%)	T-test	Significance at (*p* ≤ 0.05)
543.478	884.956	4.43E−26	Significant	75.003	87.901	1.28E−17	Significant
544.325	886.247			75.011	87.934		
545.035	886			75	88.466		
543.665	885.583			75.01	88.283		
543.997	886.382			75.014	87.934		
544.821	884.945			75	87.945		

The results were compared with those published by Ref.^24^ where it was showed increase in Cd(II) biosorption capacity after optimization using *Turbinaria ornata* biomass. The immobilized biomass showed an increase in the q_max_ value compared to the free biomass and biosorption better fitted the Langmuir model compared to the Freundlich model. A *Klebsiella* sp. strain named Yangling I2 showed adsorption efficacy towards both Cd(II) and Mn(II)^54^. The equilibrium adsorption also followed Langmuir model. As from the previous study using *Candida tropicalis* CBL-1 strain it can remove 70% Cd(II) has been reported^37^. The developed resistant strain *Candida tropicalis* has the capability to remove 85.55% Cd(II) under optimized conditions using the synthetic media developed from statistical optimization using response surface methodology.

### Instrumental evidences

#### FT-IR analysis

**Figure 6:**
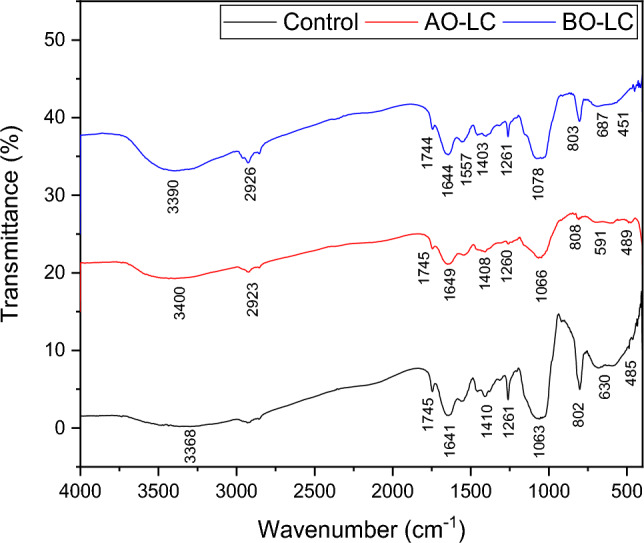
FT-IR analysis for Cd(II) biosorption by the live cells of the Cd(II) resistant strain *Candida tropicalis* XTA1874 (A) control (C), (B) after (AO-LC) and (C) before optimization (BO-LC).

The FT-IR spectra (Fig. 6, Supplementary Fig. 6) between 4000 and 400 cm^−1^ show the number of peaks indicating the presence of several functional groups in the control (C) and the Cd^2+^ resistant strain before (BO-LC), and after (AO-LC) optimisation. The peaks at 3368 cm^−1^ (C), and 3390 cm^−1^ (BO-LC) arose due to the stretching of the N–H bond of the amino groups indicating the presence of bonded –OH group^55^. The change in the peak position from 3368 cm^−1^, and 3390 cm^−1^ to 3400 cm^−1^ (AO-LC) indicates the binding of Cd^2+^ ions with N–H and –OH groups. The broader peaks at 2923 cm^−1^ (BO-LC) and 2926 cm^−1^ (AO-LC) were due to –CH stretching vibrations of –CH_3_ and –CH_2_ functional groups^55^. The peaks between 1750 cm^−1^ and 1740 cm^−1^ were due to the C=O stretching vibration indicating the presence of carboxylic acids or esters^56^. The 1641 cm^−1^ (C) and 1644 cm^−1^ (BO-LC) peaks are due to the C=O group stretching from aldehydes and ketones^57^. The shifting of the peaks from 1641 and 1644 cm^−1^ to 1649 cm^−1^ (AO-LC) were due to the binding capability of these groups with Cd^2+^ ions^55^. The peak at 1570 cm^−1^ was due to CO, C–O, and O–H groups in the BO-LC spectra^55^. The region between 1410 and 1060 cm^−1^ was due to OH, C–H stretching vibrations and C–O bending vibrations^55^. The shifting of the bands from 1063 cm^−1^ (C) and 1066 cm^−1^ (BO-LC) to 1078 cm^−1^ (AO-LC) was due to the binding capability of C–O bonds with Cd^2+^ ions^55^. The region between 810 and 800 cm^−1^ was due to C–C, C–O, and C–O–P stretching vibrations of cellular polysaccharides^58^. The shifting of the bands from 802 cm^−1^ (C) and 803 cm^−1^ (BO-LC) to 808 cm^−1^ (AO-LC) was due to the binding capability of C–C, C–O, and C–O–P bonds with Cd^2+^ ions. Similarly, the shifting of bands from 630 cm^−1^ (C) and 687 cm^−1^ (BO-LC) to 591 cm^−1^ (AO-LC) was due to the binding capability of phosphorous (P) with Cd^2+^ ions^59^. P–Cd^2+^ binding was also in the region between 490 to 450 cm^−1^^59^.

### SEM and EDAX analysis before and after optimized conditions for Cd(II) biosorption

**Figure 7:**
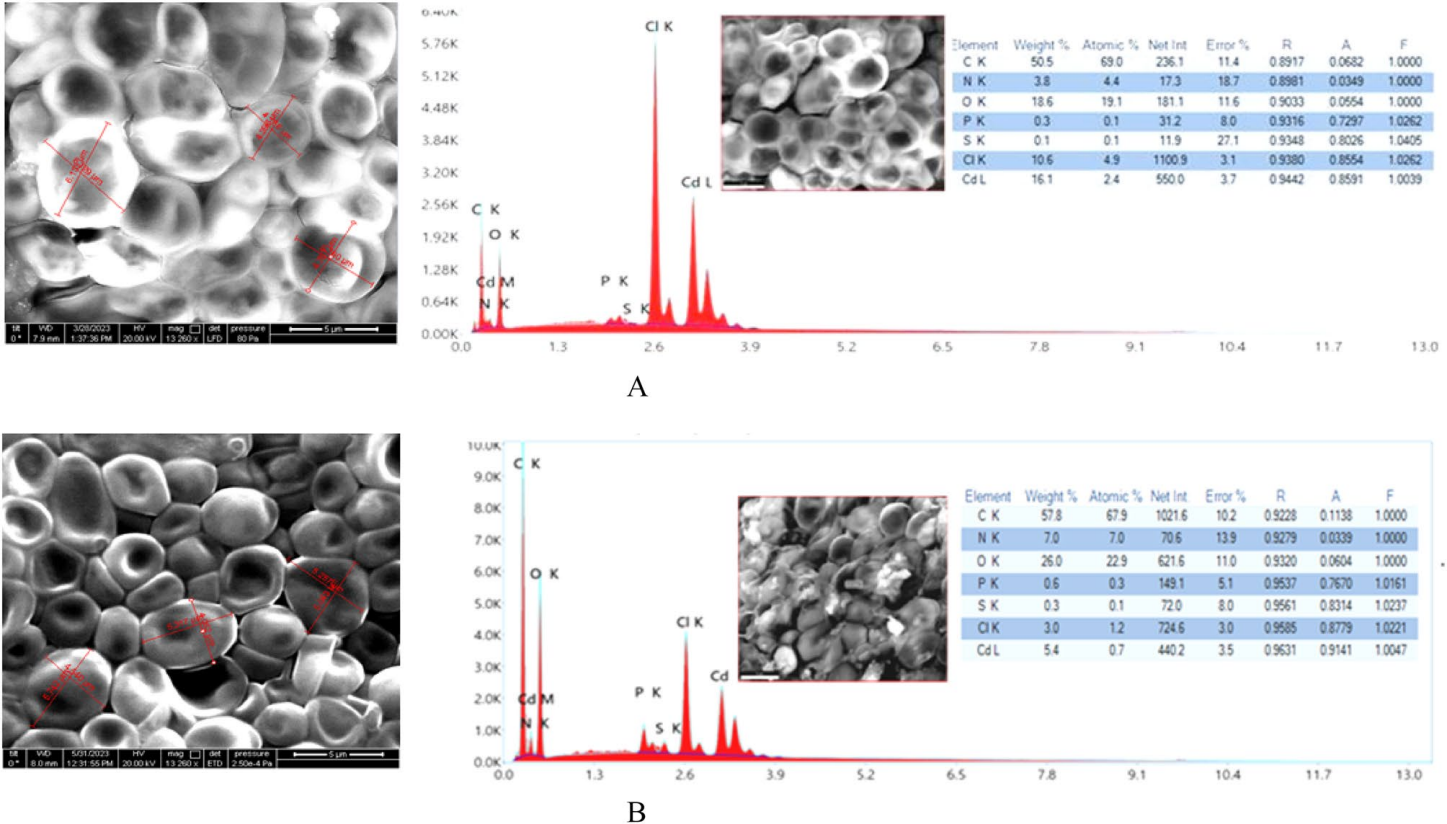
FE-SEM and EDAX analyses For Cd(II) biosorption by the live cells of the Cd(II) resistant strain *Candida tropicalis* XTA1874 before (**A**) and after optimization (**B**).

Morphological changes were detected by Field Emission Scanning Electron Microscopy (FE-SEM) (QUANTA FEG 250) before and after optimization of Cd(II) adsorption. Prominent morphological changes have been documented on the cell surface after Cd(II) treatment under optimized conditions clearly evident from two-dimensional surface measurements before (4.84 ± 0.156 × 5.182 ± 0.025 µm) and after optimization (5.236 ± 0.502 × 5.021 ± 0.581 µm) of Cd(II) biosorption by the developed resistant strain *Candida tropicalis* XTA1874. Elemental analysis by Energy Dispersive X-Ray (EDAX) (ELEMENT EDAX) analysis demonstrates pronounced increase of cell surface accumulation of Cd(II) after optimization (Fig. 7, Supplementary Fig. 7). The EDAX analysis also shows the presence of the peaks for C, N, O, P and S in both samples. Morphological changes in the changes in cells under stressed conditions resulted from the adaption with heavy metal stress^17,60^. Microbial physiological alteration with concomitant morphological changes has also been reported^61^. EDAX analysis also enlighten us about the elemental composition which shows increased microbial sorption of Cd(II) under optimized conditions.

### Analysis of the desorption efficiency and reusability of the biomass

**Figure 8:**
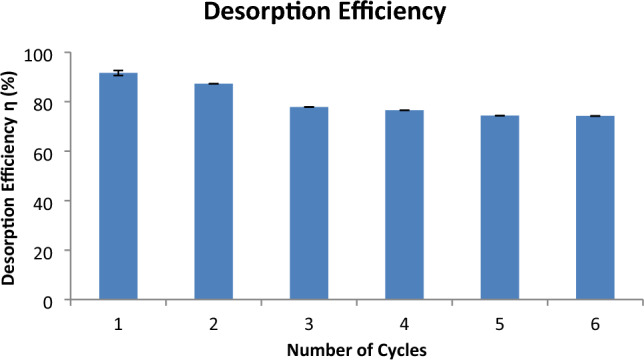
Graphical representation of the desorption efficiencies with the number of cycles.

Desorption efficiency (η%) and reusability of the biomass is regarded as one of the most important properties that make waste water treatment as a cost effective process. As can be seen from (Table 12, Fig. 8, Supplementary Table 25, Supplementary Fig. 8) that biomass from the developed resistant strain showed efficient desorption capacity (Q5: Results) (91.648 ± 0.197%) at the first round of the desorption experiment. The adsorbent was reused with slight decrease in the adsorptive removal and desorption efficiency (η%). Desorption analysis was carried out for five cycles after that no change in desorption efficiency was observed. In each reusage cycle of the biomass the surface and intracellularly accumulated amount (mg/g) has been shown which was determined by EDTA chelation and acid digestion respectively (Supplementary Table 25). Kinetic analysis also showed that equilibrium was reached at 150 min. of contact time with the eluent and a little retention of Cd(II) (q_t_ = 0.005 mg/g) (Supplementary Table 26). The terms q_i_ and q_t_ signifies the initial amount (mg/g) of surface accumulated Cd(II) and the amount of Cd(II) still remained in the biomass at time (t) after contact with the eluent solution respectively (Supplementary Table 26).

15 $${\text{Desorption efficiency }}\left( {\eta \, \% } \right) \, = {\text{ C}}_{{\text{r}}} \times {\text{V}}_{{\text{r}}} /\left( {{\text{C}}_{{\text{i}}} - {\text{C}}_{{\text{e}}} } \right){\text{V}} \times {1}00$$

**Table 12:** Estimation of desorption capacity (η %) and the regeneration capacity of the biomass.

Number of cycles	C_r_	C_i_	C_e_	V	Adsorbed (ppm)	Absorbed (ppm)	Surface accumulation (mg/g)	Intracellular accmulation (mg/g)	Vr	Removal (%)	η (%)
1	422.28 ± 0.0006	500	20.14 ± 0.0002	0.1	479.785 ± 0.073	0.439 ± 0.0002	319.614 ± 0.0003	0.294 ± 0.001	0.104	96.096 ± 0.073	91.648 ± 0.197
2	362.28 ± 0.0003	500	72.281 ± 0.0004	0.1	427.678 ± 0.039	0.239 ± 0.0002	284.987 ± 0.0003	0.161 ± 0.0005	0.103	85.577 ± 0.012	87.251 ± 0.078
								0.107 ± 0.0005			
3	302.28 ± 0.0001	500	100.282 ± 0.0005	0.1	399.689 ± 0.026	0.159 ± 0.0001	266.373 ± 0.0001	0.014 ± 0.0001	0.103	80.008 ± 0.044	77.876 ± 0.018
4	298.28 ± 0.0005	500	102.364 ± 0.0009	0.1	397.632 ± 0.002	0.02 ± 0.00003	258.355 ± 0.0001	0.005 ± 0.0001	0.102	79.727 ± 0.16	76.565 ± 0.019
5	282.28 ± 0.0002	500	112.46 ± 0.0002	0.1	387.532 ± 0.0003	0.008 ± 0.0003	258.355 ± 0.0001	0.005 ± 0.0001	0.102	77.514 ± 0.002	74.394 ± 0.06
6	281.95 ± 0.0005	500	112.46 ± 0.0002	0.1	387.532 ± 0.0002	0.008 ± 0.0001			0.102	77.514 ± 0.002	74.237 ± 0.016

C_r_, Concentration of Cd(II) in the desorbing solution (ppm), V_r_, Volume of the desorbing solution, C_i_, initial Cd(II) concentration at the adsorbing solution (500 ppm), V, volume of the adsorbing solution (0.1L), C_e_, Cd(II) concentration (ppm) in the adsorbing solution at equilibrium.

**Figure 9:**
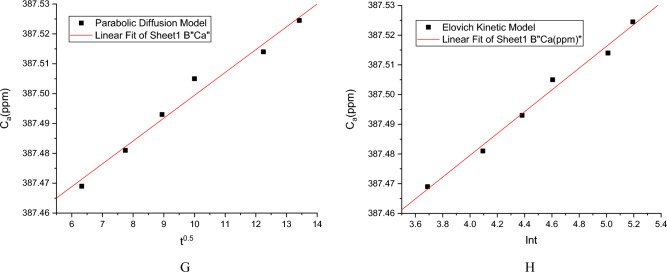
Evaluation of Cd(II) desorption kinetics by parabolic diffusion (G) and Elovich model (H) by Cd(II) resistant strain *Candida tropicalis* XTA1874.

**Figure 10:**
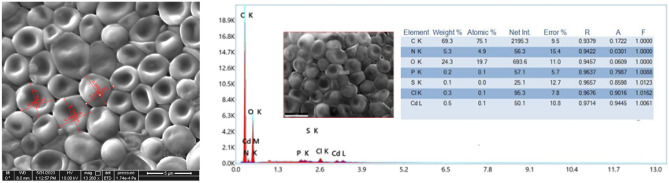
FE-SEM and EDAX analyses of developed Cd(II) resistant strain *Candida tropicalis* XTA1874 after desorption.

Kinetic analysis of desorption was carried out using liner plotting of parabolic diffusion model and Elovich-type model (Table 13, Fig. 9, Supplementary Table 27, Supplementary Fig. 9)^62^. FE SEM image with EDAX analysis of the cells (3.956 ± 1.296 × 3.878 ± 0.097 µm) after desorption have been shown in (Fig. 10, Supplementary Fig. 10). EDAX analysis showed a little retention of Cd(II) (0.5wt%) even after desorption of Cd(II) from the biomass.

**Table 13:** Estimated desorption kinetics parameters.

Model	Equation	Parameters	Ca_0exp_	Ca_0cal_	α	β	R^2^	SE
Parabolic diffusion model	1/C_a_ = 1/C_a0_ − K_a2_t	C_a_, Cd(II) released at time t C_a0_, Cd(II) concentration in solution when all ions released	387.532	387.5346 ± 0.004	–	–	0.86566	0.014
Elovich type model	C_a_ = (1/β)ln(αβ) + (1/β)lnt	α, initial Cd(II) ion desorption rate (mg L^−1^ min^−1^) β, desorption rate constant (mg g^−1^)	387.532	387.526 ± 0.002	1.98E+168	359.712	0.92158	0.009

To analyze the best fitting of the models, the coefficient of determination (R^2^) and standard error of estimate (SE) were calculated by the following formula

16 $$\mathrm{SE}= \sqrt{\sum (\mathrm{Ci}-\mathrm{Ci{^{\prime}}})2/(\mathrm{N}-2)}$$

Ci and Ci′ measured and calculated Cd(II) in solution, N is the sample size (6).

In the Elovich Model it was assumed that αβt >  > 1^63,64^.

Based on the values of R^2^ and SE (Table 13, Supplementary Table 27), it can be demonstrated that desorption kinetics is following the Elovich Kinetic Model where the calculated and experimental values of C_a0_ are very close. The derived parameter data complied with the Elovich model assumption αβt >  > 1^62^. Cd(II) release from soil has been tested by various organic acids where it has been found that parabolic diffusion best fitted Cd(II) desorption kinetics^65^. One the other hand^62^ found Elovich type model to be best fitted for desorption kinetic data.

## Conclusion

Toxicant removal by microbial biosorption represents an efficient and cost-effective means of environmental remediation. The developed resistant strain *Candida tropicalis* XTA1874 exhibited high biosorption capacity after optimizing the culture conditions. In this work the contributions of various nutritional factors have been considered to aggravate microbial growth and biosorption capacity. The obtained results indicate significant Cd(II) binding after optimized conditions. The data follows the Langmuir isotherm model and biosorption plowed pseudo second order kinetics. Each of the nutritional factors plays vital role in accelerating microbial growth and toxicant removal process besides the physical parameters. Based on the optimization study a synthetic media has been developed which aids in accelerated microbial growth and bio-removal capacity. The strain was also undergone efficient desorption and showed significant bio-removal capacity as far as six cycles. Based on the above findings it can be concluded that the strain has tremendous bio-removal capacity and can be assumed to be effectively used in Cd(II) removal from polluted water bodies with an efficient and easily doable technique.

## Supplementary Information

Supplementary Information.

## Data Availability

All data generated or analysed during this study are included in this published article [Supplementary Information files].
