# Supplementary material for: Evidences for the augmented Cd(II) biosorption by Cd(II) resistant strain Candida tropicalis XTA1874 from contaminated aqueous medium
Source: Sci Rep. 2023 Jul 25;13:12034. doi: 10.1038/s41598-023-38485-z (PMC10368703; doi:10.1038/s41598-023-38485-z)
Supplement: Supplementary file 1 — Supplementary Information. [file 41598_2023_38485_MOESM1_ESM.docx]

**S Table 1: Independent variables and their corresponding levels for Cd(II) Biosorption**

| Independent Variables | Symbol |  | Coded Levels |  |  |  |  |
| --- | --- | --- | --- | --- | --- | --- | --- |
|  |  |  | - α | - 1 | 0 | + 1 | + α |
| Glucose concentration (%) | A |  | 6 | 8 | 10 | 12 | 14 |
| Urea concentration (%) | B |  | 0.6 | 0.8 | 1 | 1.2 | 1.4 |
| K_2_HPO_4_ concentration (%) | C |  | 0.02 | 0.06 | 0.1 | 0.14 | 0.18 |
| KH_2_PO_4_ concentration (%) | D |  | 0.02 | 0.06 | 0.1 | 0.14 | 0.18 |
| MgSO_4_.7H_2_O concentration (%) | E |  | 0.005 | 0.03 | 0.055 | 0.08 | 0.105 |
| KCl concentration  (%) | F |  | 0.1 | 0.4 | 0.7 | 1 | 1.3 |
| CoCl_2_.5H_2_O concentration (%) | G |  | 0.01 | 0.03 | 0.05 | 0.07 | 0.09 |
| NH_4_VO_2_ concentration (%) | H |  | 0.25 | 0.5 | 0.75 | 1 | 1.25 |
| Na_2_MoO_4_.2H_2_O concentration (%) | J |  | 0.02 | 0.06 | 0.1 | 0.14 | 0.18 |
| CaCO_3_ concentration (%) | K |  | 0.01 | 0.03 | 0.05 | 0.07 | 0.09 |
| FeSO_4_.7H_2_O concentration (%) | L |  | 0.01 | 0.03 | 0.05 | 0.07 | 0.09 |
| ZnSO_4_.7H_2_O concentration (%) | M |  | 0.005 | 0.03 | 0.055 | 0.08 | 0.105 |
| MnSO_4_.4H_2_O concentration (%) | M |  | 0.03 | 0.04 | 0.05 | 0.06 | 0.07 |
| NiSO_4_.7H_2_O concentration (%) | O |  | 0.05 | 0.06 | 0.07 | 0.08 | 0.09 |
| Na_2_B_4_O_7_.10H_2_O concentration (%) | P |  | 0.25 | 0.5 | 0.75 | 1 | 1.25 |
| Dry cell weight (mg/ml) | Q |  | 0.35 | 1 | 1.5 | 3 | 4.9 |
|  |  |  |  |  |  |  |  |

**S Table 2**

**Experimental Design Based on Central Composite Design (CCD)**

**Independent Values** **Response**

| **Runs** | **A:**  **Glucose conc. (%)** | **B:**  **Urea conc. (%)** | **C:**  **K_2_HPO_4_ conc. (%)** | **D:**  **KH_2_PO_4_ conc. (%)** | **E:**  **MgSO_4_.7H_2_O conc. (%)** | **F:**  **KCl conc. (%)** | **G:**  **CoCl_2_.6H_2_O conc. (%)** | **H:**  **NH_4_VO_2_ conc. (%)** | **I:**  **Na_2_MoO_4_.2H_2_O conc. (%)** | **J:**  **CaCO_3_ conc. (%)** | **K:**  **FeSO_4_.7H_2_O conc. (%)** | **L:**  **ZnSO_4_.7H_2_O conc. (%)** | **M:**  **MnSO_4_.4H_2_O conc. (%)** | **N:**  **NiSO_4_.7H_2_O conc. (%)** | | **O:**  **Na_2_B_4_O_7_.10H_2_O conc. (%)** | **Q:**  **Dry Cell Weight(mg/ml)** | **Actual Value** | **Predicted Value** | **Residual** |
| --- | --- | --- | --- | --- | --- | --- | --- | --- | --- | --- | --- | --- | --- | --- | --- | --- | --- | --- | --- | --- |
| 1 | 10 | 1 | 0.1 | 0.1 | 0.055 | 0.7 | 0.05 | 0.75 | 0.1 | 0.01 | 0.05 | 0.055 | 0.05 | | 0.07 | 0.75 | **1.6** | 90.63 | 88.39 | 3.24 |
| 2 | 12 | 0.8 | 0.06 | 0.06 | 0.03 | 0.4 | 0.07 | 0.5 | 0.14 | 0.03 | 0.07 | 0.03 | 0.04 | | 0.08 | 0.5 | 1.5 | 65.37 | 61.88 | 3.87 |
| 3 | 8 | 1.2 | 0.06 | 0.06 | 0.03 | 0.4 | 0.03 | 0.5 | 0.14 | 0.07 | 0.03 | 0.03 | 0.04 | | 0.08 | 0.5 | 1.3 | 54.26 | 57.78 | -1.69 |
| 4 | 12 | 0.8 | 0.14 | 0.06 | 0.03 | 1 | 0.07 | 1 | 0.06 | 0.03 | 0.03 | 0.08 | 0.06 | | 0.08 | 0.5 | 2.0 | 87.55 | 85.31 | 4.36 |
| 5 | 8 | 1.2 | 0.06 | 0.14 | 0.03 | 0.4 | 0.07 | 1 | 0.06 | 0.03 | 0.03 | 0.03 | 0.04 | | 0.06 | 0.5 | 1.8 | 56.33 | 55.88 | 0.4334 |
| 6 | 8 | 1.2 | 0.14 | 0.14 | 0.03 | 0.4 | 0.03 | 0.5 | 0.14 | 0.03 | 0.07 | 0.03 | 0.04 | | 0.08 | 0.5 | 1.9 | 88.46 | 89.34 | -0.9966 |
| 7 | 12 | 0.8 | 0.06 | 0.14 | 0.08 | 1 | 0.07 | 1 | 0.14 | 0.07 | 0.03 | 0.08 | 0.04 | | 0.06 | 1 | 2.3 | 86.20 | 89.00 | -4.79 |
| 8 | 8 | 1.2 | 0.14 | 0.14 | 0.03 | 1 | 0.03 | 0.5 | 0.14 | 0.07 | 0.03 | 0.08 | 0.06 | | 0.08 | 1 | 1.9 | 62.44 | 63.37 | 0.9957 |
| 9 | 12 | 1.2 | 0.14 | 0.06 | 0.08 | 1 | 0.03 | 0.5 | 0.06 | 0.03 | 0.07 | 0.08 | 0.06 | | 0.06 | 1 | 1.7 | 88.28 | 88.25 | -3.01 |
| 10 | 12 | 0.8 | 0.14 | 0.14 | 0.03 | 1 | 0.03 | 0.5 | 0.14 | 0.07 | 0.03 | 0.08 | 0.06 | | 0.06 | 0.5 | 1.4 | 87.12 | 90.08 | -2.00 |
| 11 | 12 | 1.2 | 0.14 | 0.06 | 0.03 | 0.4 | 0.03 | 0.5 | 0.14 | 0.07 | 0.07 | 0.03 | 0.06 | | 0.08 | 1 | 1.8 | 88.55 | 86.90 | -0.4366 |
| 12 | 12 | 0.8 | 0.06 | 0.14 | 0.03 | 0.4 | 0.07 | 0.5 | 0.06 | 0.03 | 0.03 | 0.08 | 0.06 | | 0.06 | 0.5 | 1.9 | 52.37 | 58.43 | -1.96 |
| 13 | 12 | 1.2 | 0.06 | 0.06 | 0.03 | 0.4 | 0.07 | 1 | 0.06 | 0.07 | 0.03 | 0.03 | 0.06 | | 0.06 | 1 | 2.0 | 55.74 | 61.39 | -1.44 |
| 14 | 8 | 1.2 | 0.14 | 0.14 | 0.08 | 1 | 0.07 | 1 | 0.06 | 0.03 | 0.03 | 0.03 | 0.06 | | 0.06 | 0.5 | 1.5 | 87.74 | 87.43 | 0.3079 |
| 15 | 8 | 0.8 | 0.14 | 0.14 | 0.03 | 0.4 | 0.03 | 1 | 0.06 | 0.07 | 0.03 | 0.03 | 0.06 | | 0.06 | 1 | 1.8 | 86.21 | 89.55 | -2.30 |
| 16 | 8 | 0.8 | 0.14 | 0.06 | 0.03 | 0.4 | 0.07 | 0.5 | 0.14 | 0.03 | 0.03 | 0.03 | 0.06 | | 0.08 | 1 | 1.6 | 90.24 | 88.63 | 1.41 |
| 17 | 12 | 0.8 | 0.14 | 0.06 | 0.03 | 0.4 | 0.07 | 1 | 0.06 | 0.07 | 0.07 | 0.03 | 0.04 | | 0.08 | 1 | 2.0 | 61.47 | 64.44 | -1.01 |
| 18 | 12 | 1.2 | 0.14 | 0.14 | 0.08 | 1 | 0.07 | 1 | 0.14 | 0.07 | 0.07 | 0.08 | 0.06 | | 0.08 | 1 | 1.7 | 88.41 | 85.67 | 1.41 |
| 19 | 12 | 0.8 | 0.14 | 0.06 | 0.08 | 0.4 | 0.03 | 1 | 0.14 | 0.03 | 0.07 | 0.03 | 0.06 | | 0.08 | 1 | 2.1 | 53.34 | 55.56 | 1.3 |
| 20 | 12 | 0.8 | 0.06 | 0.06 | 0.03 | 0.4 | 0.07 | 1 | 0.14 | 0.03 | 0.07 | 0.08 | 0.06 | | 0.06 | 1 | 1.6 | 90.32 | 89.89 | 0.2274 |
| 21 | 12 | 0.8 | 0.06 | 0.06 | 0.08 | 1 | 0.07 | 1 | 0.06 | 0.07 | 0.07 | 0.03 | 0.06 | | 0.08 | 1 | 1.9 | 89.66 | 87.52 | 2.10 |
| 22 | 8 | 0.8 | 0.06 | 0.06 | 0.08 | 1 | 0.03 | 0.5 | 0.14 | 0.07 | 0.07 | 0.03 | 0.04 | | 0.06 | 0.5 | 1.7 | 92.25 | 94.33 | -2.09 |
| 23 | 12 | 1.2 | 0.06 | 0.14 | 0.08 | 1 | 0.07 | 0.5 | 0.06 | 0.03 | 0.07 | 0.08 | 0.06 | | 0.08 | 0.5 | 1.4 | 88.47 | 89.70 | -0.7241 |
| 24 | 12 | 1.2 | 0.14 | 0.14 | 0.08 | 0.4 | 0.03 | 1 | 0.14 | 0.07 | 0.07 | 0.03 | 0.04 | | 0.06 | 1 | 1.9 | 88.39 | 88.44 | -0.0238 |
| 25 | 12 | 1.2 | 0.14 | 0.14 | 0.03 | 1 | 0.03 | 1 | 0.06 | 0.03 | 0.07 | 0.08 | 0.04 | | 0.08 | 1 | 1.6 | 89.74 | 88.02 | 0.7621 |
| 26 | 8 | 1.2 | 0.14 | 0.06 | 0.03 | 1 | 0.03 | 1 | 0.06 | 0.07 | 0.07 | 0.08 | 0.06 | | 0.08 | 0.5 | 1.8 | 88.32 | 87.98 | -1.61 |
| 27 | 8 | 1.2 | 0.14 | 0.14 | 0.03 | 0.4 | 0.07 | 0.5 | 0.14 | 0.07 | 0.03 | 0.03 | 0.04 | | 0.06 | 1 | 2.0 | 48.49 | 53.84 | -7.51 |
| 28 | 8 | 0.8 | 0.14 | 0.14 | 0.03 | 1 | 0.07 | 1 | 0.06 | 0.07 | 0.03 | 0.08 | 0.04 | | 0.08 | 1 | 1.7 | 88.52 | 88.88 | 1.68 |
| 29 | 8 | 0.8 | 0.06 | 0.14 | 0.08 | 1 | 0.03 | 0.5 | 0.06 | 0.07 | 0.03 | 0.08 | 0.06 | | 0.08 | 0.5 | 1.9 | 88.04 | 87.78 | -0.7314 |
| 30 | 8 | 0.8 | 0.06 | 0.14 | 0.03 | 0.4 | 0.07 | 1 | 0.14 | 0.07 | 0.07 | 0.08 | 0.04 | | 0.06 | 0.5 | 1.6 | 82.56 | 88.55 | -3.47 |
| 31 | 12 | 1.2 | 0.06 | 0.14 | 0.03 | 1 | 0.07 | 1 | 0.14 | 0.03 | 0.03 | 0.03 | 0.06 | | 0.08 | 0.5 | 1.8 | 86.74 | 92.80 | -3.05 |
| 32 | 8 | 0.8 | 0.06 | 0.14 | 0.08 | 0.4 | 0.03 | 1 | 0.06 | 0.03 | 0.07 | 0.08 | 0.06 | | 0.06 | 0.5 | 2.0 | 82.23 | 85.27 | -1.05 |
| 33 | 8 | 1.2 | 0.14 | 0.06 | 0.08 | 0.4 | 0.03 | 1 | 0.14 | 0.03 | 0.07 | 0.03 | 0.06 | | 0.06 | 0.5 | 1.4 | 88.23 | 86.48 | -0.3508 |
| 34 | 12 | 1.2 | 0.06 | 0.14 | 0.08 | 1 | 0.03 | 0.5 | 0.06 | 0.07 | 0.03 | 0.08 | 0.06 | | 0.06 | 1 | 1.8 | 62.52 | 62.03 | 0.4624 |
| 35 | 8 | 1.2 | 0.14 | 0.06 | 0.03 | 0.4 | 0.07 | 1 | 0.06 | 0.07 | 0.07 | 0.03 | 0.04 | | 0.06 | 0.5 | 1.6 | 90.14 | 88.33 | 1.48 |
| 36 | 8 | 1.2 | 0.06 | 0.06 | 0.03 | 0.4 | 0.07 | 1 | 0.14 | 0.03 | 0.07 | 0.08 | 0.06 | | 0.08 | 0.5 | 1.4 | 58.43 | 58.76 | -0.3238 |
| 37 | 10 | 1 | 0.02 | 0.1 | 0.055 | 0.7 | 0.05 | 0.75 | 0.1 | 0.05 | 0.05 | 0.055 | 0.05 | | 0.07 | 0.75 | 2.1 | 90.67 | 91.92 | -1.210 |
| 38 | 8 | 0.8 | 0.14 | 0.14 | 0.08 | 0.4 | 0.07 | 1 | 0.14 | 0.03 | 0.03 | 0.03 | 0.04 | | 0.06 | 1 | 1.8 | 86.88 | 85.51 | 1.41 |
| 39 | 8 | 0.8 | 0.06 | 0.06 | 0.03 | 1 | 0.03 | 1 | 0.06 | 0.07 | 0.03 | 0.08 | 0.04 | | 0.06 | 0.5 | 1.9 | 84.70 | 84.52 | 0.2377 |
| 40 | 8 | 0.8 | 0.14 | 0.14 | 0.03 | 0.4 | 0.07 | 0.5 | 0.06 | 0.03 | 0.07 | 0.08 | 0.04 | | 0.06 | 1 | 1.7 | 87.55 | 87.54 | -0.1240 |
| 41 | 12 | 1.2 | 0.06 | 0.06 | 0.08 | 0.4 | 0.07 | 1 | 0.14 | 0.07 | 0.07 | 0.03 | 0.04 | | 0.08 | 0.5 | 2.1 | 82.78 | 92.44 | -7.70 |
| 42 | 12 | 1.2 | 0.06 | 0.06 | 0.03 | 1 | 0.07 | 1 | 0.06 | 0.03 | 0.07 | 0.08 | 0.04 | | 0.06 | 0.5 | 2.0 | 83.64 | 87.66 | -1.92 |
| 43 | 8 | 0.8 | 0.14 | 0.14 | 0.03 | 0.4 | 0.03 | 0.5 | 0.06 | 0.07 | 0.03 | 0.08 | 0.04 | | 0.08 | 0.5 | 2.0 | 67.45 | 62.72 | 6.17 |
| 44 | 10 | 1 | 0.1 | 0.1 | 0.055 | 1.3 | 0.05 | 0.75 | 0.1 | 0.05 | 0.05 | 0.055 | 0.05 | | 0.07 | 0.75 | 1.8 | 84.52 | 85.46 | 1.19 |
| 45 | 12 | 0.8 | 0.14 | 0.06 | 0.03 | 0.4 | 0.03 | 1 | 0.06 | 0.03 | 0.03 | 0.03 | 0.04 | | 0.06 | 0.5 | 1.9 | 54.34 | 76.62 | -18.28 |
| 46 | 8 | 0.8 | 0.14 | 0.14 | 0.03 | 1 | 0.03 | 1 | 0.06 | 0.03 | 0.07 | 0.08 | 0.04 | | 0.06 | 0.5 | 1.8 | 50.34 | 53.97 | -1.21 |
| 47 | 8 | 0.8 | 0.06 | 0.14 | 0.03 | 1 | 0.07 | 1 | 0.14 | 0.03 | 0.03 | 0.03 | 0.06 | | 0.06 | 1 | 1.6 | 59.67 | 59.14 | -1.49 |
| 48 | 12 | 0.8 | 0.14 | 0.14 | 0.03 | 1 | 0.07 | 0.5 | 0.14 | 0.03 | 0.07 | 0.08 | 0.06 | | 0.08 | 1 | 1.9 | 63.26 | 66.38 | -0.1504 |
| 49 | 8 | 1.2 | 0.06 | 0.06 | 0.08 | 0.4 | 0.03 | 1 | 0.06 | 0.07 | 0.07 | 0.08 | 0.04 | | 0.08 | 0.5 | 1.5 | 62.44 | 67.69 | -1.23 |
| 50 | 12 | 0.8 | 0.06 | 0.14 | 0.08 | 0.4 | 0.07 | 0.5 | 0.14 | 0.03 | 0.07 | 0.08 | 0.04 | | 0.08 | 1 | 2.0 | 91.30 | 90.64 | -2.37 |
| 51 | 12 | 0.8 | 0.06 | 0.14 | 0.03 | 0.4 | 0.07 | 1 | 0.06 | 0.03 | 0.03 | 0.03 | 0.04 | | 0.08 | 1 | 2.0 | 92.76 | 87.47 | 4.90 |
| 52 | 8 | 1.2 | 0.14 | 0.06 | 0.03 | 1 | 0.03 | 0.5 | 0.06 | 0.07 | 0.07 | 0.03 | 0.04 | | 0.06 | 1 | 1.4 | 68.70 | 64.82 | 3.94 |
| 53 | 12 | 0.8 | 0.14 | 0.06 | 0.08 | 1 | 0.03 | 0.5 | 0.14 | 0.07 | 0.03 | 0.03 | 0.06 | | 0.06 | 1 | 1.6 | 64.43 | 58.66 | 4.41 |
| 54 | 10 | 1 | 0.1 | 0.1 | 0.055 | 0.7 | 0.05 | 0.75 | 0.1 | 0.05 | 0.05 | 0.055 | 0.05 | | 0.09 | 0.75 | 1.8 | 89.99 | 91.21 | -0.0640 |
| 55 | 12 | 1.2 | 0.06 | 0.06 | 0.08 | 1 | 0.03 | 1 | 0.14 | 0.07 | 0.07 | 0.08 | 0.06 | | 0.06 | 0.5 | 1.8 | 88.24 | 84.54 | 1.82 |
| 56 | 8 | 0.8 | 0.14 | 0.14 | 0.08 | 1 | 0.07 | 0.5 | 0.14 | 0.07 | 0.07 | 0.03 | 0.04 | | 0.08 | 1 | 1.9 | 91.37 | 90.02 | 2.525 |
| 57 | 8 | 0.8 | 0.14 | 0.14 | 0.03 | 1 | 0.03 | 0.5 | 0.06 | 0.03 | 0.07 | 0.03 | 0.06 | | 0.08 | 1 | 2.0 | 90.64 | 91.24 | -1.74 |
| 58 | 8 | 0.8 | 0.14 | 0.06 | 0.03 | 0.4 | 0.07 | 1 | 0.14 | 0.03 | 0.03 | 0.08 | 0.04 | | 0.06 | 0.5 | 2.0 | 59.23 | 52.35 | 7.58 |
| 59 | 8 | 0.8 | 0.14 | 0.14 | 0.08 | 1 | 0.03 | 1 | 0.14 | 0.03 | 0.03 | 0.08 | 0.06 | | 0.08 | 1 | 2.0 | 87.45 | 86.23 | 0.0217 |
| 60 | 8 | 1.2 | 0.14 | 0.06 | 0.03 | 0.4 | 0.03 | 0.5 | 0.06 | 0.03 | 0.03 | 0.08 | 0.06 | | 0.06 | 0.5 | 1.9 | 89.79 | 88.87 | -0.0576 |
| 61 | 12 | 1.2 | 0.14 | 0.06 | 0.03 | 1 | 0.07 | 0.5 | 0.14 | 0.07 | 0.07 | 0.08 | 0.04 | | 0.06 | 1 | 1.8 | 91.14 | 93.61 | -0.8743 |
| 62 | 10 | 1 | 0.1 | 0.1 | 0.055 | 0.7 | 0.05 | 0.75 | 0.1 | 0.05 | 0.05 | 0.055 | 0.05 | | 0.07 | 0.75 | 1.9 | 87.97 | 91.21 | -2.24 |
| 63 | 10 | 1 | 0.1 | 0.1 | 0.055 | 0.7 | 0.05 | 0.75 | 0.1 | 0.05 | 0.05 | 0.055 | 0.05 | | 0.07 | 0.75 | 1.7 | 85.01 | 91.12 | -5.20 |
| 64 | 12 | 0.8 | 0.14 | 0.14 | 0.08 | 1 | 0.07 | 0.5 | 0.06 | 0.03 | 0.03 | 0.08 | 0.04 | | 0.06 | 0.5 | 1.7 | 87.14 | 87.23 | 0.9455 |
| 65 | 8 | 0.8 | 0.06 | 0.14 | 0.03 | 1 | 0.03 | 0.5 | 0.14 | 0.07 | 0.07 | 0.08 | 0.04 | | 0.06 | 1 | 1.2 | 63.34 | 57.54 | 1.81 |
| 66 | 8 | 1.2 | 0.06 | 0.06 | 0.03 | 1 | 0.07 | 0.5 | 0.14 | 0.07 | 0.03 | 0.08 | 0.06 | | 0.06 | 0.5 | 1.8 | 65.47 | 62.42 | 5.15 |
| 67 | 12 | 0.8 | 0.06 | 0.14 | 0.08 | 0.4 | 0.03 | 1 | 0.14 | 0.07 | 0.03 | 0.03 | 0.06 | | 0.08 | 1 | 1.8 | 89.12 | 92.16 | -1.04 |
| 68 | 12 | 0.8 | 0.06 | 0.06 | 0.03 | 0.4 | 0.03 | 1 | 0.14 | 0.07 | 0.03 | 0.08 | 0.06 | | 0.08 | 0.5 | 1.7 | 51.37 | 52.23 | 0.1115 |
| 69 | 12 | 1.2 | 0.14 | 0.06 | 0.08 | 1 | 0.03 | 1 | 0.06 | 0.03 | 0.07 | 0.03 | 0.04 | | 0.08 | 0.5 | 1.8 | 54.32 | 56.32 | -0.9645 |
| 70 | 8 | 0.8 | 0.06 | 0.06 | 0.03 | 1 | 0.03 | 0.5 | 0.06 | 0.07 | 0.03 | 0.03 | 0.06 | | 0.08 | 1 | 1.7 | 58.64 | 61.34 | -2.75 |
| 71 | 12 | 1.2 | 0.14 | 0.06 | 0.03 | 0.4 | 0.07 | 1 | 0.14 | 0.03 | 0.03 | 0.08 | 0.04 | | 0.08 | 1 | 1.8 | 86.48 | 89.47 | -2.77 |
| 72 | 8 | 1.2 | 0.06 | 0.14 | 0.03 | 1 | 0.07 | 0.5 | 0.06 | 0.07 | 0.07 | 0.03 | 0.04 | | 0.08 | 0.5 | 1.7 | 48.67 | 54.41 | -5.56 |
| 73 | 8 | 1.2 | 0.06 | 0.06 | 0.08 | 1 | 0.03 | 1 | 0.06 | 0.03 | 0.03 | 0.03 | 0.06 | | 0.08 | 1 | 2.0 | 88.32 | 89.52 | -0.8556 |
| 74 | 10 | 0.6 | 0.1 | 0.1 | 0.055 | 0.7 | 0.05 | 0.75 | 0.1 | 0.05 | 0.05 | 0.055 | 0.05 | | 0.07 | 0.75 | 2.1 | 86.57 | 86.64 | 0.2134 |
| 75 | 10 | 1 | 0.1 | 0.1 | 0.055 | 0.7 | 0.05 | 0.75 | 0.1 | 0.05 | 0.05 | 0.055 | 0.05 | | 0.07 | 0.75 | 1.9 | 94.76 | 91.21 | 3.57 |
| 76 | 12 | 0.8 | 0.06 | 0.14 | 0.03 | 1 | 0.07 | 1 | 0.06 | 0.07 | 0.07 | 0.08 | 0.06 | | 0.08 | 0.5 | 1.7 | 54.27 | 56.58 | -2.24 |
| 77 | 12 | 1.2 | 0.06 | 0.14 | 0.03 | 0.4 | 0.03 | 0.5 | 0.14 | 0.03 | 0.03 | 0.03 | 0.06 | | 0.08 | 1 | 2.1 | 61.34 | 58.57 | 2.41 |
| 78 | 8 | 0.8 | 0.06 | 0.14 | 0.08 | 0.4 | 0.07 | 1 | 0.06 | 0.07 | 0.03 | 0.08 | 0.06 | | 0.08 | 1 | 1.8 | 60.33 | 62.47 | -2.07 |
| 79 | 8 | 1.2 | 0.06 | 0.14 | 0.03 | 1 | 0.03 | 1 | 0.06 | 0.03 | 0.03 | 0.08 | 0.06 | | 0.08 | 0.5 | 2.0 | 58.76 | 60.15 | -1.36 |
| 80 | 10 | 1 | 0.1 | 0.1 | 0.055 | 0.7 | 0.05 | 0.75 | 0.1 | 0.05 | 0.05 | 0.055 | 0.05 | | 0.07 | 0.75 | 1.9 | 90.47 | 91.64 | -0.8208 |
| 81 | 8 | 0.8 | 0.14 | 0.06 | 0.03 | 1 | 0.07 | 0.5 | 0.14 | 0.07 | 0.07 | 0.08 | 0.04 | | 0.08 | 0.5 | 1.8 | 64.37 | 67.74 | -3.05 |
| 82 | 12 | 0.8 | 0.14 | 0.14 | 0.08 | 1 | 0.07 | 1 | 0.06 | 0.03 | 0.03 | 0.03 | 0.06 | | 0.08 | 1 | 1.7 | 62.42 | 63.47 | -1.40 |
| 83 | 8 | 1.2 | 0.06 | 0.06 | 0.03 | 1 | 0.07 | 1 | 0.14 | 0.07 | 0.03 | 0.03 | 0.04 | | 0.08 | 1 | 1.8 | 87.99 | 85.54 | 2.45 |
| 84 | 10 | 1 | 0.1 | 0.1 | 0.055 | 0.7 | 0.05 | 0.75 | 0.1 | 0.05 | 0.05 | 0.105 | 0.05 | | 0.07 | 0.75 | 1.6 | 88.64 | 88.57 | 0.0735 |
| 85 | 8 | 0.8 | 0.14 | 0.06 | 0.08 | 1 | 0.07 | 0.5 | 0.06 | 0.07 | 0.03 | 0.08 | 0.06 | | 0.06 | 1 | 1.7 | 86.21 | 90.44 | -4.23 |
| 86 | 12 | 0.8 | 0.06 | 0.14 | 0.08 | 0.4 | 0.03 | 0.5 | 0.14 | 0.07 | 0.03 | 0.08 | 0.04 | | 0.06 | 0.5 | 1.8 | 50.24 | 57.07 | -6.83 |
| 87 | 12 | 0.8 | 0.06 | 0.06 | 0.08 | 1 | 0.03 | 0.5 | 0.06 | 0.03 | 0.03 | 0.08 | 0.04 | | 0.08 | 1 | 1.8 | 87.95 | 86.51 | 1.44 |
| 88 | 12 | 1.2 | 0.06 | 0.14 | 0.08 | 1 | 0.07 | 1 | 0.06 | 0.03 | 0.07 | 0.03 | 0.04 | | 0.06 | 1 | 1.5 | 56.32 | 59.02 | -2.70 |
| 89 | 10 | 1 | 0.1 | 0.1 | 0.055 | 0.7 | 0.05 | 0.75 | 0.1 | 0.05 | 0.05 | 0.055 | 0.05 | | 0.05 | 0.75 | 1.9 | 89.74 | 89.56 | 0.1765 |
| 90 | 10 | 1 | 0.1 | 0.1 | 0.055 | 0.7 | 0.01 | 0.75 | 0.1 | 0.05 | 0.05 | 0.055 | 0.05 | | 0.07 | 0.75 | 1.9 | 90.12 | 88.96 | 1.16 |
| 91 | 12 | 1.2 | 0.06 | 0.06 | 0.03 | 0.4 | 0.03 | 0.5 | 0.06 | 0.03 | 0.07 | 0.08 | 0.04 | | 0.06 | 1 | 1.8 | 62.41 | 63.82 | -1.41 |
| 92 | 10 | 1 | 0.1 | 0.1 | 0.055 | 0.7 | 0.05 | 0.75 | 0.1 | 0.05 | 0.05 | 0.055 | 0.05 | | 0.07 | 0.75 | 1.9 | 89.45 | 91.21 | -1.76 |
| 93 | 12 | 1.2 | 0.06 | 0.06 | 0.08 | 0.4 | 0.07 | 0.5 | 0.14 | 0.07 | 0.07 | 0.08 | 0.06 | | 0.06 | 1 | 1.8 | 54.27 | 56.68 | -2.41 |
| 94 | 10 | 1 | 0.1 | 0.1 | 0.055 | 0.7 | 0.05 | 0.75 | 0.1 | 0.05 | 0.05 | 0.055 | 0.05 | | 0.07 | 0.75 | 1.4 | 92.47 | 91.21 | 1.26 |
| 95 | 8 | 0.8 | 0.14 | 0.14 | 0.08 | 1 | 0.03 | 0.5 | 0.14 | 0.03 | 0.03 | 0.03 | 0.04 | | 0.06 | 0.5 | 1.7 | 58.74 | 62.60 | -3.86 |
| 96 | 12 | 1.2 | 0.06 | 0.14 | 0.03 | 1 | 0.03 | 1 | 0.14 | 0.07 | 0.07 | 0.03 | 0.06 | | 0.06 | 1 | 2.0 | 46.37 | 54.28 | -7.91 |
| 97 | 10 | 1 | 0.1 | 0.1 | 0.055 | 0.7 | 0.09 | 0.75 | 0.1 | 0.05 | 0.05 | 0.055 | 0.05 | | 0.07 | 0.75 | 1.7 | 89.64 | 90.63 | -0.9938 |
| 98 | 8 | 1.2 | 0.14 | 0.14 | 0.03 | 0.4 | 0.07 | 1 | 0.14 | 0.07 | 0.03 | 0.08 | 0.06 | | 0.08 | 0.5 | 1.8 | 88.21 | 92.90 | -4.69 |
| 99 | 12 | 1.2 | 0.14 | 0.14 | 0.08 | 1 | 0.03 | 0.5 | 0.14 | 0.03 | 0.03 | 0.03 | 0.04 | | 0.08 | 1 | 1.7 | 86.33 | 88.84 | -2.51 |
| 100 | 8 | 1.2 | 0.14 | 0.14 | 0.03 | 0.4 | 0.03 | 1 | 0.14 | 0.03 | 0.07 | 0.08 | 0.06 | | 0.06 | 1 | 1.7 | 52.37 | 53.07 | -0.7025 |
| 101 | 8 | 0.8 | 0.06 | 0.06 | 0.03 | 1 | 0.07 | 1 | 0.06 | 0.03 | 0.07 | 0.08 | 0.04 | | 0.08 | 1 | 1.6 | 49.65 | 55.11 | -5.46 |
| 102 | 12 | 0.8 | 0.06 | 0.06 | 0.08 | 1 | 0.03 | 1 | 0.06 | 0.03 | 0.03 | 0.03 | 0.06 | | 0.06 | 0.5 | 1.5 | 64.27 | 62.74 | 1.53 |
| 103 | 8 | 0.8 | 0.06 | 0.14 | 0.08 | 1 | 0.07 | 0.5 | 0.06 | 0.03 | 0.07 | 0.08 | 0.06 | | 0.06 | 1 | 1.3 | 64.21 | 58.35 | 5.86 |
| 104 | 8 | 0.8 | 0.06 | 0.06 | 0.03 | 0.4 | 0.07 | 0.5 | 0.06 | 0.07 | 0.03 | 0.08 | 0.04 | | 0.06 | 1 | 2.0 | 64.52 | 64.40 | 0.1244 |
| 105 | 12 | 1.2 | 0.06 | 0.14 | 0.08 | 0.4 | 0.03 | 0.5 | 0.06 | 0.03 | 0.07 | 0.03 | 0.04 | | 0.06 | 0.5 | 1.8 | 88.47 | 85.35 | 3.12 |
| 106 | 8 | 1.2 | 0.06 | 0.14 | 0.03 | 1 | 0.03 | 0.5 | 0.06 | 0.03 | 0.03 | 0.03 | 0.04 | | 0.06 | 1 | 1.9 | 86.54 | 90.61 | -4.07 |
| 107 | 8 | 1.2 | 0.14 | 0.06 | 0.03 | 1 | 0.07 | 0.5 | 0.06 | 0.03 | 0.03 | 0.03 | 0.04 | | 0.08 | 0.5 | 2.3 | 87.99 | 84.89 | 3.10 |
| 108 | 12 | 1.2 | 0.14 | 0.14 | 0.03 | 1 | 0.03 | 0.5 | 0.06 | 0.03 | 0.07 | 0.03 | 0.06 | | 0.06 | 0.5 | 1.9 | 58.74 | 56.50 | 2.24 |
| 109 | 10 | 1 | 0.18 | 0.1 | 0.055 | 0.7 | 0.05 | 0.75 | 0.1 | 0.05 | 0.05 | 0.055 | 0.05 | | 0.07 | 0.75 | 1.7 | 92.12 | 90.75 | 1.37 |
| 110 | 12 | 1.2 | 0.14 | 0.06 | 0.03 | 0.4 | 0.03 | 1 | 0.14 | 0.07 | 0.07 | 0.08 | 0.04 | | 0.06 | 0.5 | 1.4 | 56.34 | 58.39 | -2.05 |
| 111 | 8 | 1.2 | 0.14 | 0.14 | 0.03 | 1 | 0.07 | 1 | 0.14 | 0.03 | 0.07 | 0.03 | 0.04 | | 0.08 | 1 | 1.8 | 52.31 | 53.17 | -0.8634 |
| 112 | 8 | 1.2 | 0.14 | 0.14 | 0.08 | 0.4 | 0.03 | 1 | 0.06 | 0.03 | 0.03 | 0.08 | 0.04 | | 0.08 | 0.5 | 1.9 | 88.52 | 89.94 | -1.42 |
| 113 | 8 | 0.8 | 0.14 | 0.14 | 0.08 | 0.4 | 0.03 | 1 | 0.14 | 0.07 | 0.07 | 0.03 | 0.04 | | 0.08 | 0.5 | 2.0 | 60.12 | 62.25 | -2.13 |
| 114 | 8 | 1.2 | 0.06 | 0.14 | 0.08 | 0.4 | 0.03 | 0.5 | 0.14 | 0.07 | 0.03 | 0.08 | 0.04 | | 0.08 | 1 | 1.5 | 88.58 | 83.56 | 5.02 |
| 115 | 12 | 1.2 | 0.06 | 0.14 | 0.03 | 1 | 0.03 | 0.5 | 0.14 | 0.07 | 0.07 | 0.08 | 0.04 | | 0.08 | 0.5 | 1.8 | 88.47 | 89.95 | -1.48 |
| 116 | 8 | 1.2 | 0.06 | 0.06 | 0.08 | 0.4 | 0.03 | 0.5 | 0.06 | 0.07 | 0.07 | 0.03 | 0.06 | | 0.06 | 1 | 1.6 | 89.24 | 90.75 | -1.51 |
| 117 | 12 | 1.2 | 0.14 | 0.06 | 0.03 | 1 | 0.07 | 1 | 0.14 | 0.07 | 0.07 | 0.03 | 0.06 | | 0.08 | 0.5 | 2.0 | 56.34 | 56.22 | 0.1244 |
| 118 | 12 | 0.8 | 0.14 | 0.14 | 0.03 | 0.4 | 0.03 | 0.5 | 0.14 | 0.03 | 0.07 | 0.03 | 0.04 | | 0.06 | 1 | 1.7 | 54.02 | 52.09 | 1.93 |
| 119 | 12 | 0.8 | 0.06 | 0.14 | 0.03 | 0.4 | 0.03 | 1 | 0.06 | 0.07 | 0.07 | 0.03 | 0.04 | | 0.06 | 0.5 | 2.1 | 62.34 | 54.55 | 7.79 |
| 120 | 12 | 1.2 | 0.06 | 0.06 | 0.08 | 1 | 0.03 | 0.5 | 0.14 | 0.07 | 0.07 | 0.03 | 0.04 | | 0.08 | 1 | 1.6 | 58.74 | 57.59 | 1.15 |
| 121 | 6 | 1 | 0.1 | 0.1 | 0.055 | 0.7 | 0.05 | 0.75 | 0.1 | 0.05 | 0.05 | 0.055 | 0.05 | | 0.07 | 0.75 | 1.9 | 92.45 | 89.56 | 2.89 |
| 122 | 10 | 1 | 0.1 | 0.1 | 0.055 | 0.7 | 0.05 | 0.75 | 0.18 | 0.05 | 0.05 | 0.055 | 0.05 | | 0.07 | 0.75 | 1.7 | 86.22 | 89.01 | -2.79 |
| 123 | 8 | 0.8 | 0.14 | 0.06 | 0.03 | 0.4 | 0.03 | 0.5 | 0.14 | 0.07 | 0.07 | 0.03 | 0.06 | | 0.06 | 0.5 | 1.4 | 48.75 | 53.83 | -5.08 |
| 124 | 8 | 1.2 | 0.06 | 0.14 | 0.08 | 0.4 | 0.07 | 0.5 | 0.14 | 0.03 | 0.07 | 0.08 | 0.04 | | 0.06 | 0.5 | 1.9 | 58.74 | 56.96 | 1.78 |
| 125 | 12 | 0.8 | 0.06 | 0.06 | 0.03 | 1 | 0.07 | 0.5 | 0.14 | 0.07 | 0.03 | 0.08 | 0.06 | | 0.08 | 1 | 1.6 | 86.55 | 89.96 | -3.41 |
| 126 | 8 | 0.8 | 0.14 | 0.06 | 0.03 | 0.4 | 0.03 | 1 | 0.14 | 0.07 | 0.07 | 0.08 | 0.04 | | 0.08 | 1 | 1.8 | 88.12 | 90.52 | -2.40 |
| 127 | 12 | 0.8 | 0.14 | 0.14 | 0.03 | 0.4 | 0.03 | 1 | 0.14 | 0.03 | 0.07 | 0.08 | 0.06 | | 0.08 | 0.5 | 2.0 | 89.21 | 86.38 | 2.83 |
| 128 | 10 | 1 | 0.1 | 0.1 | 0.055 | 0.7 | 0.05 | 0.75 | 0.1 | 0.05 | 0.05 | 0.055 | 0.05 | | 0.07 | 0.75 | 1.7 | 86.56 | 91.21 | -4.65 |
| 129 | 12 | 1.2 | 0.14 | 0.14 | 0.08 | 1 | 0.07 | 0.5 | 0.14 | 0.07 | 0.07 | 0.03 | 0.04 | | 0.06 | 0.5 | 1.9 | 58.74 | 54.29 | 4.45 |
| 130 | 8 | 0.8 | 0.14 | 0.06 | 0.03 | 1 | 0.03 | 1 | 0.14 | 0.03 | 0.03 | 0.03 | 0.06 | | 0.08 | 0.5 | 1.6 | 56.00 | 55.43 | 0.5704 |
| 131 | 12 | 0.8 | 0.14 | 0.14 | 0.08 | 1 | 0.03 | 1 | 0.06 | 0.07 | 0.07 | 0.03 | 0.06 | | 0.06 | 0.5 | 1.8 | 88.97 | 88.48 | 0.4907 |
| 132 | 12 | 1.2 | 0.06 | 0.06 | 0.03 | 1 | 0.03 | 0.5 | 0.06 | 0.07 | 0.03 | 0.03 | 0.06 | | 0.06 | 0.5 | 2.0 | 84.72 | 84.87 | -0.1508 |
| 133 | 12 | 0.8 | 0.06 | 0.14 | 0.03 | 1 | 0.03 | 0.5 | 0.06 | 0.03 | 0.03 | 0.03 | 0.04 | | 0.08 | 0.5 | 1.4 | 62.14 | 61.69 | 0.4455 |
| 134 | 12 | 1.2 | 0.14 | 0.14 | 0.03 | 0.4 | 0.03 | 0.5 | 0.06 | 0.07 | 0.03 | 0.08 | 0.04 | | 0.06 | 1 | 1.8 | 88.14 | 86.94 | 1.20 |
| 135 | 10 | 1 | 0.1 | 0.1 | 0.055 | 0.7 | 0.05 | 0.75 | 0.1 | 0.05 | 0.05 | 0.055 | 0.07 | | 0.07 | 0.75 | 1.6 | 86.54 | 87.81 | -1.27 |
| 136 | 12 | 0.8 | 0.06 | 0.06 | 0.08 | 1 | 0.07 | 0.5 | 0.06 | 0.07 | 0.07 | 0.08 | 0.04 | | 0.06 | 0.5 | 1.4 | 66.34 | 64.04 | 2.30 |
| 137 | 12 | 1.2 | 0.14 | 0.14 | 0.08 | 0.4 | 0.03 | 0.5 | 0.14 | 0.07 | 0.07 | 0.08 | 0.06 | | 0.08 | 0.5 | 2.1 | 58.75 | 56.97 | 1.78 |
| 138 | 8 | 1.2 | 0.06 | 0.14 | 0.08 | 1 | 0.07 | 1 | 0.14 | 0.07 | 0.03 | 0.08 | 0.04 | | 0.08 | 0.5 | 1.8 | 67.45 | 61.41 | 6.04 |
| 139 | 8 | 0.8 | 0.06 | 0.14 | 0.08 | 1 | 0.03 | 1 | 0.06 | 0.07 | 0.03 | 0.03 | 0.04 | | 0.06 | 1 | 1.9 | 74.12 | 71.04 | 3.08 |
| 140 | 12 | 0.8 | 0.14 | 0.14 | 0.08 | 0.4 | 0.03 | 0.5 | 0.06 | 0.03 | 0.03 | 0.03 | 0.06 | | 0.08 | 0.5 | 1.7 | 88.41 | 90.89 | -2.48 |
| 141 | 12 | 1.2 | 0.06 | 0.06 | 0.08 | 0.4 | 0.03 | 0.5 | 0.14 | 0.03 | 0.03 | 0.08 | 0.06 | | 0.08 | 0.5 | 2.1 | 87.12 | 86.74 | 0.3794 |
| 142 | 8 | 0.8 | 0.06 | 0.06 | 0.03 | 1 | 0.07 | 0.5 | 0.06 | 0.03 | 0.07 | 0.03 | 0.06 | | 0.06 | 0.5 | 2.0 | 90.24 | 88.79 | 1.45 |
| 143 | 12 | 0.8 | 0.06 | 0.06 | 0.08 | 0.4 | 0.03 | 1 | 0.06 | 0.07 | 0.07 | 0.08 | 0.04 | | 0.06 | 1 | 2.0 | 92.34 | 91.87 | 0.4689 |
| 144 | 10 | 1 | 0.1 | 0.1 | 0.055 | 0.7 | 0.05 | 0.75 | 0.1 | 0.05 | 0.05 | 0.055 | 0.03 | | 0.07 | 0.75 | 1.8 | 90.24 | 88.80 | 1.44 |
| 145 | 12 | 0.8 | 0.14 | 0.14 | 0.03 | 1 | 0.07 | 1 | 0.14 | 0.03 | 0.07 | 0.03 | 0.04 | | 0.06 | 0.5 | 1.9 | 88.72 | 84.76 | 3.96 |
| 146 | 8 | 0.8 | 0.06 | 0.14 | 0.08 | 1 | 0.07 | 1 | 0.06 | 0.03 | 0.07 | 0.03 | 0.04 | | 0.08 | 0.5 | 1.8 | 88.09 | 85.05 | 3.04 |
| 147 | 10 | 1 | 0.1 | 0.1 | 0.055 | 0.7 | 0.05 | 0.75 | 0.1 | 0.05 | 0.05 | 0.055 | 0.05 | | 0.07 | 0.75 | 1.6 | 36.45 | 32.28 | 4.17 |
| 148 | 12 | 0.8 | 0.14 | 0.06 | 0.03 | 1 | 0.07 | 0.5 | 0.06 | 0.03 | 0.03 | 0.03 | 0.04 | | 0.06 | 1 | 1.9 | 62.14 | 59.35 | 2.79 |
| 149 | 8 | 1.2 | 0.14 | 0.14 | 0.03 | 1 | 0.03 | 1 | 0.14 | 0.07 | 0.03 | 0.03 | 0.04 | | 0.06 | 0.5 | 1.5 | 89.45 | 86.02 | 3.43 |
| 150 | 12 | 1.2 | 0.06 | 0.06 | 0.03 | 0.4 | 0.03 | 1 | 0.06 | 0.03 | 0.07 | 0.03 | 0.06 | | 0.08 | 0.5 | 2.0 | 89.12 | 87.70 | 1.42 |
| 151 | 10 | 1 | 0.1 | 0.02 | 0.055 | 0.7 | 0.05 | 0.75 | 0.1 | 0.05 | 0.05 | 0.055 | 0.05 | | 0.07 | 0.75 | 2.0 | 88.02 | 87.51 | 0.5088 |
| 152 | 12 | 0.8 | 0.14 | 0.14 | 0.03 | 1 | 0.03 | 1 | 0.14 | 0.07 | 0.03 | 0.03 | 0.04 | | 0.08 | 1 | 1.4 | 62.21 | 61.22 | 0.9950 |
| 153 | 8 | 0.8 | 0.06 | 0.14 | 0.08 | 0.4 | 0.03 | 0.5 | 0.06 | 0.03 | 0.07 | 0.03 | 0.04 | | 0.08 | 1 | 1.6 | 52.47 | 53.54 | -1.07 |
| 154 | 10 | 1.4 | 0.1 | 0.1 | 0.055 | 0.7 | 0.05 | 0.75 | 0.1 | 0.05 | 0.05 | 0.055 | 0.05 | | 0.07 | 0.75 | 1.8 | 86.02 | 86.08 | -0.0553 |
| 155 | 8 | 1.2 | 0.06 | 0.14 | 0.03 | 1 | 0.07 | 1 | 0.06 | 0.07 | 0.07 | 0.08 | 0.06 | | 0.06 | 1 | 1.8 | 90.37 | 85.64 | 4.73 |
| 156 | 8 | 0.8 | 0.14 | 0.06 | 0.03 | 1 | 0.07 | 1 | 0.14 | 0.07 | 0.07 | 0.03 | 0.06 | | 0.06 | 1 | 1.9 | 88.52 | 87.87 | 0.6464 |
| 157 | 8 | 1.2 | 0.06 | 0.06 | 0.03 | 1 | 0.03 | 0.5 | 0.14 | 0.03 | 0.07 | 0.08 | 0.06 | | 0.08 | 1 | 2.0 | 92.14 | 90.02 | 2.12 |
| 158 | 12 | 0.8 | 0.14 | 0.06 | 0.03 | 1 | 0.03 | 1 | 0.06 | 0.07 | 0.07 | 0.08 | 0.06 | | 0.06 | 1 | 2.0 | 54.27 | 54.88 | -0.6080 |
| 159 | 12 | 1.2 | 0.06 | 0.06 | 0.08 | 1 | 0.07 | 1 | 0.14 | 0.03 | 0.03 | 0.08 | 0.06 | | 0.08 | 1 | 2.0 | 62.47 | 59.19 | 3.28 |
| 160 | 8 | 1.2 | 0.14 | 0.14 | 0.08 | 1 | 0.03 | 1 | 0.06 | 0.07 | 0.07 | 0.03 | 0.06 | | 0.08 | 1 | 1.9 | 58.01 | 59.85 | -1.84 |
| 161 | 12 | 0.8 | 0.14 | 0.06 | 0.08 | 0.4 | 0.03 | 0.5 | 0.14 | 0.03 | 0.07 | 0.08 | 0.04 | | 0.06 | 0.5 | 1.8 | 90.99 | 85.10 | 5.89 |
| 162 | 12 | 0.8 | 0.14 | 0.06 | 0.08 | 0.4 | 0.07 | 0.5 | 0.14 | 0.07 | 0.03 | 0.08 | 0.04 | | 0.08 | 1 | 1.9 | 58.47 | 58.50 | -0.0257 |
| 163 | 12 | 1.2 | 0.06 | 0.14 | 0.08 | 0.4 | 0.07 | 0.5 | 0.06 | 0.07 | 0.03 | 0.03 | 0.04 | | 0.08 | 1 | 1.7 | 54.23 | 57.31 | -3.08 |
| 164 | 12 | 1.2 | 0.06 | 0.14 | 0.03 | 0.4 | 0.03 | 1 | 0.14 | 0.03 | 0.03 | 0.08 | 0.04 | | 0.06 | 0.5 | 1.7 | 86.07 | 83.65 | 2.42 |
| 165 | 8 | 1.2 | 0.06 | 0.14 | 0.03 | 0.4 | 0.03 | 0.5 | 0.06 | 0.07 | 0.07 | 0.08 | 0.06 | | 0.06 | 0.5 | 1.2 | 52.47 | 56.74 | -4.27 |
| 166 | 12 | 1.2 | 0.14 | 0.06 | 0.08 | 0.4 | 0.07 | 0.5 | 0.06 | 0.03 | 0.07 | 0.03 | 0.04 | | 0.08 | 1 | 1.8 | 91.88 | 88.51 | 3.37 |
| 167 | 12 | 1.2 | 0.14 | 0.14 | 0.08 | 0.4 | 0.07 | 0.5 | 0.14 | 0.03 | 0.03 | 0.08 | 0.06 | | 0.06 | 1 | 1.8 | 86.45 | 86.09 | 0.3581 |
| 168 | 12 | 0.8 | 0.14 | 0.06 | 0.03 | 1 | 0.03 | 0.5 | 0.06 | 0.07 | 0.07 | 0.03 | 0.04 | | 0.08 | 0.5 | 1.7 | 92.12 | 89.69 | 2.43 |
| 169 | 12 | 1.2 | 0.14 | 0.14 | 0.03 | 0.4 | 0.07 | 0.5 | 0.06 | 0.03 | 0.07 | 0.08 | 0.04 | | 0.08 | 0.5 | 1.8 | 56.74 | 61.57 | -4.83 |
| 170 | 8 | 0.8 | 0.06 | 0.06 | 0.08 | 0.4 | 0.07 | 0.5 | 0.14 | 0.07 | 0.07 | 0.08 | 0.06 | | 0.08 | 0.5 | 1.7 | 86.25 | 88.63 | -2.38 |
| 171 | 8 | 1.2 | 0.06 | 0.06 | 0.08 | 0.4 | 0.07 | 1 | 0.06 | 0.03 | 0.03 | 0.08 | 0.04 | | 0.06 | 1 | 1.8 | 88.52 | 90.45 | -1.93 |
| 172 | 12 | 0.8 | 0.06 | 0.06 | 0.03 | 1 | 0.03 | 1 | 0.14 | 0.03 | 0.07 | 0.03 | 0.04 | | 0.08 | 1 | 1.7 | 88.45 | 90.34 | -1.89 |
| 173 | 8 | 1.2 | 0.14 | 0.06 | 0.08 | 0.4 | 0.03 | 0.5 | 0.14 | 0.03 | 0.07 | 0.08 | 0.04 | | 0.08 | 1 | 2.0 | 55.45 | 57.95 | -2.50 |
| 174 | 8 | 0.8 | 0.06 | 0.06 | 0.08 | 1 | 0.07 | 0.5 | 0.14 | 0.03 | 0.03 | 0.03 | 0.04 | | 0.08 | 1 | 2.1 | 59.67 | 61.91 | -2.24 |
| 175 | 12 | 1.2 | 0.14 | 0.06 | 0.08 | 0.4 | 0.03 | 1 | 0.06 | 0.07 | 0.03 | 0.08 | 0.06 | | 0.08 | 1 | 1.9 | 88.03 | 88.74 | -0.7101 |
| 176 | 8 | 1.2 | 0.14 | 0.14 | 0.08 | 1 | 0.03 | 0.5 | 0.06 | 0.07 | 0.07 | 0.08 | 0.04 | | 0.06 | 0.5 | 1.7 | 88.01 | 90.66 | -2.65 |
| 177 | 8 | 0.8 | 0.14 | 0.14 | 0.08 | 0.4 | 0.03 | 0.5 | 0.14 | 0.07 | 0.07 | 0.08 | 0.06 | | 0.06 | 1 | 2.1 | 88.12 | 86.02 | 2.10 |
| 178 | 12 | 1.2 | 0.14 | 0.06 | 0.03 | 1 | 0.03 | 0.5 | 0.14 | 0.03 | 0.03 | 0.08 | 0.04 | | 0.08 | 0.5 | 1.8 | 58.99 | 61.46 | -2.47 |
| 179 | 12 | 0.8 | 0.14 | 0.14 | 0.03 | 0.4 | 0.07 | 0.5 | 0.14 | 0.07 | 0.03 | 0.03 | 0.04 | | 0.08 | 0.5 | 2.0 | 88.25 | 89.94 | -1.69 |
| 180 | 12 | 0.8 | 0.06 | 0.14 | 0.03 | 1 | 0.03 | 1 | 0.06 | 0.03 | 0.03 | 0.08 | 0.06 | | 0.06 | 1 | 1.982 | 91.45 | 85.13 | 6.32 |
| 181 | 12 | 0.8 | 0.14 | 0.06 | 0.08 | 1 | 0.03 | 1 | 0.14 | 0.07 | 0.03 | 0.08 | 0.04 | | 0.08 | 0.5 | 1.813 | 86.22 | 82.79 | 3.43 |
| 182 | 8 | 1.2 | 0.06 | 0.06 | 0.08 | 1 | 0.07 | 1 | 0.06 | 0.07 | 0.07 | 0.03 | 0.06 | | 0.06 | 0.5 | 1.701 | 58.27 | 58.16 | 0.1088 |
| 183 | 8 | 1.2 | 0.14 | 0.14 | 0.08 | 0.4 | 0.07 | 0.5 | 0.06 | 0.07 | 0.07 | 0.03 | 0.06 | | 0.08 | 0.5 | 1.850 | 90.07 | 86.42 | 3.65 |
| 184 | 10 | 1 | 0.1 | 0.1 | 0.055 | 0.7 | 0.05 | 0.75 | 0.1 | 0.05 | 0.05 | 0.055 | 0.05 | | 0.07 | 0.75 | 1.628 | 94.64 | 91.21 | 3.43 |
| 185 | 8 | 0.8 | 0.14 | 0.06 | 0.08 | 0.4 | 0.07 | 1 | 0.06 | 0.03 | 0.07 | 0.08 | 0.06 | | 0.08 | 1 | 1.795 | 88.12 | 88.85 | -0.7288 |
| 186 | 12 | 0.8 | 0.14 | 0.06 | 0.08 | 0.4 | 0.07 | 1 | 0.14 | 0.07 | 0.03 | 0.03 | 0.06 | | 0.06 | 0.5 | 1.826 | 88.97 | 83.51 | 5.46 |
| 187 | 10 | 1 | 0.1 | 0.1 | 0.005 | 0.7 | 0.05 | 0.75 | 0.1 | 0.05 | 0.05 | 0.055 | 0.05 | | 0.07 | 0.75 | 1.823 | 89.97 | 87.55 | 2.42 |
| 188 | 8 | 0.8 | 0.06 | 0.06 | 0.03 | 0.4 | 0.03 | 1 | 0.06 | 0.03 | 0.07 | 0.03 | 0.06 | | 0.06 | 1 | 1.515 | 52.47 | 53.76 | -1.29 |
| 189 | 10 | 1 | 0.1 | 0.1 | 0.055 | 0.7 | 0.05 | 0.75 | 0.1 | 0.05 | 0.05 | 0.055 | 0.05 | | 0.07 | 1.25 | 1.9 | 92.78 | 93.08 | -0.2997 |
| 190 | 12 | 0.8 | 0.14 | 0.06 | 0.08 | 1 | 0.07 | 1 | 0.14 | 0.03 | 0.07 | 0.08 | 0.04 | | 0.06 | 1 | 1.9 | 58.97 | 60.23 | -1.26 |
| 191 | 10 | 1 | 0.1 | 0.1 | 0.055 | 0.7 | 0.05 | 0.75 | 0.1 | 0.09 | 0.05 | 0.055 | 0.05 | | 0.07 | 0.75 | 1.8 | 88.12 | 91.24 | -3.12 |
| 192 | 8 | 1.2 | 0.14 | 0.06 | 0.08 | 1 | 0.03 | 1 | 0.14 | 0.07 | 0.03 | 0.08 | 0.04 | | 0.06 | 1 | 1.9 | 52.14 | 58.76 | -6.62 |
| 193 | 12 | 1.2 | 0.06 | 0.06 | 0.08 | 1 | 0.07 | 0.5 | 0.14 | 0.03 | 0.03 | 0.03 | 0.04 | | 0.06 | 0.5 | 1.8 | 88.21 | 88.03 | 0.1813 |
| 194 | 8 | 0.8 | 0.06 | 0.14 | 0.03 | 1 | 0.03 | 1 | 0.14 | 0.07 | 0.07 | 0.03 | 0.06 | | 0.08 | 0.5 | 1.4 | 87.68 | 86.40 | 1.28 |
| 195 | 8 | 1.2 | 0.14 | 0.14 | 0.08 | 1 | 0.07 | 0.5 | 0.06 | 0.03 | 0.03 | 0.08 | 0.04 | | 0.08 | 1 | 1.7 | 56.98 | 56.15 | 0.8325 |
| 196 | 10 | 1 | 0.1 | 0.1 | 0.055 | 0.7 | 0.05 | 0.75 | 0.02 | 0.05 | 0.05 | 0.055 | 0.05 | | 0.07 | 0.75 | 2.0 | 92.45 | 89.50 | 2.95 |
| 197 | 12 | 0.8 | 0.14 | 0.06 | 0.03 | 0.4 | 0.07 | 0.5 | 0.06 | 0.07 | 0.07 | 0.08 | 0.06 | | 0.06 | 0.5 | 1.7 | 88.12 | 87.09 | 1.03 |
| 198 | 10 | 1 | 0.1 | 0.1 | 0.055 | 0.7 | 0.05 | 0.75 | 0.1 | 0.05 | 0.05 | 0.055 | 0.05 | | 0.07 | 0.75 | 1.8 | 94.52 | 91.21 | 3.31 |
| 199 | 12 | 1.2 | 0.14 | 0.06 | 0.03 | 0.4 | 0.07 | 0.5 | 0.14 | 0.03 | 0.03 | 0.03 | 0.06 | | 0.06 | 0.5 | 1.7 | 49.74 | 51.80 | -2.06 |
| 200 | 8 | 1.2 | 0.14 | 0.06 | 0.08 | 0.4 | 0.07 | 1 | 0.14 | 0.07 | 0.03 | 0.03 | 0.06 | | 0.08 | 1 | 1.7 | 56.07 | 58.93 | -2.86 |
| 201 | 10 | 1 | 0.1 | 0.1 | 0.105 | 0.7 | 0.05 | 0.75 | 0.1 | 0.05 | 0.05 | 0.055 | 0.05 | | 0.07 | 0.75 | 1.6 | 86.45 | 88.71 | -2.26 |
| 202 | 8 | 1.2 | 0.06 | 0.06 | 0.03 | 0.4 | 0.03 | 1 | 0.14 | 0.07 | 0.03 | 0.08 | 0.06 | | 0.06 | 1 | 1.5 | 86.07 | 84.23 | 1.84 |
| 203 | 12 | 0.8 | 0.06 | 0.06 | 0.08 | 0.4 | 0.07 | 0.5 | 0.06 | 0.03 | 0.03 | 0.03 | 0.06 | | 0.06 | 1 | 1.3 | 92.99 | 88.98 | 4.01 |
| 204 | 8 | 1.2 | 0.06 | 0.14 | 0.08 | 1 | 0.03 | 0.5 | 0.14 | 0.03 | 0.07 | 0.03 | 0.06 | | 0.08 | 0.5 | 2.0 | 55.24 | 55.81 | -0.5670 |
| 205 | 12 | 0.8 | 0.06 | 0.06 | 0.08 | 0.4 | 0.07 | 1 | 0.06 | 0.03 | 0.03 | 0.08 | 0.04 | | 0.08 | 0.5 | 1.8 | 62.34 | 59.91 | 2.43 |
| 206 | 8 | 0.8 | 0.06 | 0.14 | 0.03 | 0.4 | 0.07 | 0.5 | 0.14 | 0.07 | 0.07 | 0.03 | 0.06 | | 0.08 | 1 | 1.9 | 60.12 | 56.06 | 4.06 |
| 207 | 8 | 0.8 | 0.06 | 0.06 | 0.08 | 0.4 | 0.03 | 0.5 | 0.14 | 0.03 | 0.03 | 0.08 | 0.06 | | 0.06 | 1 | 2.3 | 52.14 | 53.60 | -1.46 |
| 208 | 12 | 0.8 | 0.06 | 0.06 | 0.03 | 1 | 0.07 | 1 | 0.14 | 0.07 | 0.03 | 0.03 | 0.04 | | 0.06 | 0.5 | 1.9 | 58.45 | 64.08 | -5.63 |
| 209 | 12 | 1.2 | 0.14 | 0.14 | 0.08 | 0.4 | 0.07 | 1 | 0.14 | 0.03 | 0.03 | 0.03 | 0.04 | | 0.08 | 0.5 | 1.7 | 62.45 | 61.49 | 0.9636 |
| 210 | 10 | 1 | 0.1 | 0.1 | 0.055 | 0.7 | 0.05 | 0.75 | 0.1 | 0.05 | 0.05 | 0.055 | 0.05 | | 0.07 | 0.25 | 1.4 | 92.74 | 92.28 | 0.4612 |
| 211 | 12 | 1.2 | 0.14 | 0.14 | 0.03 | 1 | 0.07 | 0.5 | 0.06 | 0.07 | 0.03 | 0.03 | 0.06 | | 0.08 | 1 | 1.8 | 88.45 | 89.68 | -1.23 |
| 212 | 8 | 0.8 | 0.06 | 0.06 | 0.08 | 0.4 | 0.03 | 1 | 0.14 | 0.03 | 0.03 | 0.03 | 0.04 | | 0.08 | 0.5 | 1.9 | 90.64 | 86.92 | 3.72 |
| 213 | 8 | 0.8 | 0.06 | 0.06 | 0.03 | 0.4 | 0.03 | 0.5 | 0.06 | 0.03 | 0.07 | 0.08 | 0.04 | | 0.08 | 0.5 | 2.0 | 88.24 | 86.26 | 1.98 |
| 214 | 12 | 0.8 | 0.14 | 0.06 | 0.03 | 0.4 | 0.03 | 0.5 | 0.06 | 0.03 | 0.03 | 0.08 | 0.06 | | 0.08 | 1 | 1.5 | 50.41 | 55.92 | -5.51 |
| 215 | 12 | 0.8 | 0.14 | 0.14 | 0.08 | 0.4 | 0.07 | 0.5 | 0.06 | 0.07 | 0.07 | 0.03 | 0.06 | | 0.06 | 1 | 1.8 | 48.75 | 54.84 | -6.09 |
| 216 | 8 | 1.2 | 0.06 | 0.14 | 0.03 | 0.4 | 0.03 | 1 | 0.06 | 0.07 | 0.07 | 0.03 | 0.04 | | 0.08 | 1 | 1.6 | 88.34 | 91.35 | -3.01 |
| 217 | 12 | 0.8 | 0.06 | 0.06 | 0.03 | 0.4 | 0.03 | 0.5 | 0.14 | 0.07 | 0.03 | 0.03 | 0.04 | | 0.06 | 1 | 2.0 | 88.78 | 85.22 | 3.56 |
| 218 | 8 | 0.8 | 0.06 | 0.14 | 0.03 | 0.4 | 0.03 | 1 | 0.14 | 0.03 | 0.03 | 0.08 | 0.04 | | 0.08 | 1 | 1.7 | 42.12 | 50.82 | -8.70 |
| 219 | 12 | 1.2 | 0.14 | 0.14 | 0.03 | 1 | 0.07 | 1 | 0.06 | 0.07 | 0.03 | 0.08 | 0.04 | | 0.06 | 0.5 | 2.1 | 58.74 | 59.96 | -1.22 |
| 220 | 10 | 1 | 0.1 | 0.1 | 0.055 | 0.7 | 0.05 | 0.75 | 0.1 | 0.05 | 0.09 | 0.055 | 0.05 | | 0.07 | 0.75 | 1.6 | 90.99 | 85.05 | 5.94 |
| 221 | 12 | 1.2 | 0.06 | 0.14 | 0.03 | 0.4 | 0.07 | 1 | 0.14 | 0.07 | 0.07 | 0.08 | 0.04 | | 0.08 | 1 | 1.9 | 70.24 | 63.59 | 6.65 |
| 222 | 8 | 0.8 | 0.14 | 0.06 | 0.08 | 0.4 | 0.07 | 0.5 | 0.06 | 0.03 | 0.07 | 0.03 | 0.04 | | 0.06 | 0.5 | 1.7 | 49.87 | 54.05 | -4.18 |
| 223 | 8 | 1.2 | 0.14 | 0.06 | 0.08 | 1 | 0.07 | 0.5 | 0.14 | 0.03 | 0.07 | 0.03 | 0.06 | | 0.06 | 1 | 1.4 | 52.41 | 53.03 | -0.6163 |
| 224 | 8 | 1.2 | 0.06 | 0.06 | 0.08 | 0.4 | 0.07 | 0.5 | 0.06 | 0.03 | 0.03 | 0.03 | 0.06 | | 0.08 | 0.5 | 1.9 | 54.12 | 56.55 | -2.43 |
| 225 | 8 | 0.8 | 0.14 | 0.06 | 0.08 | 0.4 | 0.03 | 1 | 0.06 | 0.07 | 0.03 | 0.08 | 0.06 | | 0.06 | 0.5 | 1.6 | 66.54 | 61.67 | 4.87 |
| 226 | 8 | 0.8 | 0.14 | 0.06 | 0.03 | 1 | 0.03 | 0.5 | 0.14 | 0.03 | 0.03 | 0.08 | 0.04 | | 0.06 | 1 | 1.8 | 90.64 | 86.61 | 4.03 |
| 227 | 8 | 0.8 | 0.14 | 0.06 | 0.08 | 1 | 0.03 | 1 | 0.06 | 0.03 | 0.07 | 0.03 | 0.04 | | 0.06 | 1 | 2.0 | 87.99 | 90.02 | -2.03 |
| 228 | 8 | 0.8 | 0.06 | 0.06 | 0.08 | 0.4 | 0.07 | 1 | 0.14 | 0.07 | 0.07 | 0.03 | 0.04 | | 0.06 | 1 | 1.7 | 76.85 | 69.24 | 7.61 |
| 229 | 10 | 1 | 0.1 | 0.1 | 0.055 | 0.7 | 0.05 | 0.75 | 0.1 | 0.05 | 0.01 | 0.055 | 0.05 | | 0.07 | 0.75 | 1.9 | 79.89 | 85.67 | -5.78 |
| 230 | 12 | 1.2 | 0.06 | 0.06 | 0.03 | 0.4 | 0.07 | 0.5 | 0.06 | 0.07 | 0.03 | 0.08 | 0.04 | | 0.08 | 0.5 | 1.6 | 91.32 | 87.42 | 3.90 |
| 231 | 12 | 1.2 | 0.06 | 0.14 | 0.08 | 0.4 | 0.03 | 1 | 0.06 | 0.03 | 0.07 | 0.08 | 0.06 | | 0.08 | 1 | 1.8 | 59.32 | 59.35 | -0.0338 |
| 232 | 12 | 0.8 | 0.06 | 0.06 | 0.08 | 0.4 | 0.03 | 0.5 | 0.06 | 0.07 | 0.07 | 0.03 | 0.06 | | 0.08 | 0.5 | 2.0 | 52.37 | 53.63 | -1.26 |
| 233 | 12 | 1.2 | 0.14 | 0.06 | 0.08 | 1 | 0.07 | 0.5 | 0.06 | 0.07 | 0.03 | 0.08 | 0.06 | | 0.08 | 0.5 | 1.4 | 57.23 | 59.45 | -2.22 |
| 234 | 12 | 1.2 | 0.06 | 0.14 | 0.08 | 1 | 0.03 | 1 | 0.06 | 0.07 | 0.03 | 0.03 | 0.04 | | 0.08 | 0.5 | 1.8 | 88.24 | 89.14 | -0.9002 |
| 235 | 10 | 1 | 0.1 | 0.1 | 0.055 | 0.7 | 0.05 | 1.25 | 0.1 | 0.05 | 0.05 | 0.055 | 0.05 | | 0.07 | 0.75 | 1.6 | 86.56 | 84.86 | 1.70 |
| 236 | 8 | 0.8 | 0.06 | 0.14 | 0.03 | 1 | 0.07 | 0.5 | 0.14 | 0.03 | 0.03 | 0.08 | 0.04 | | 0.08 | 0.5 | 1.4 | 92.34 | 92.50 | -0.1649 |
| 237 | 12 | 0.8 | 0.14 | 0.14 | 0.08 | 1 | 0.03 | 0.5 | 0.06 | 0.07 | 0.07 | 0.08 | 0.04 | | 0.08 | 1 | 2.1 | 58.74 | 58.69 | 0.0516 |
| 238 | 12 | 1.2 | 0.14 | 0.06 | 0.08 | 1 | 0.07 | 1 | 0.06 | 0.07 | 0.03 | 0.03 | 0.04 | | 0.06 | 1 | 1.8 | 86.40 | 82.27 | 4.13 |
| 239 | 12 | 0.8 | 0.06 | 0.14 | 0.08 | 1 | 0.03 | 1 | 0.14 | 0.03 | 0.07 | 0.08 | 0.04 | | 0.08 | 0.5 | 1.9 | 58.74 | 59.24 | -0.4975 |
| 240 | 8 | 1.2 | 0.14 | 0.14 | 0.08 | 0.4 | 0.03 | 0.5 | 0.06 | 0.03 | 0.03 | 0.03 | 0.06 | | 0.06 | 1 | 1.7 | 67.10 | 60.56 | 6.54 |
| 241 | 12 | 1.2 | 0.14 | 0.06 | 0.08 | 0.4 | 0.03 | 0.5 | 0.06 | 0.07 | 0.03 | 0.03 | 0.04 | | 0.06 | 0.5 | 2.1 | 56.24 | 55.06 | 1.18 |
| 242 | 8 | 1.2 | 0.06 | 0.06 | 0.03 | 1 | 0.03 | 1 | 0.14 | 0.03 | 0.07 | 0.03 | 0.04 | | 0.06 | 0.5 | 2.0 | 62.23 | 56.88 | 5.35 |
| 243 | 8 | 1.2 | 0.06 | 0.06 | 0.08 | 1 | 0.03 | 0.5 | 0.06 | 0.03 | 0.03 | 0.08 | 0.04 | | 0.06 | 0.5 | 2.0 | 60.47 | 62.58 | -2.11 |
| 244 | 12 | 1.2 | 0.06 | 0.06 | 0.03 | 1 | 0.07 | 0.5 | 0.06 | 0.03 | 0.07 | 0.03 | 0.06 | | 0.08 | 1 | 1.8 | 58.24 | 57.35 | 1.05 |
| 245 | 12 | 0.8 | 0.14 | 0.06 | 0.08 | 1 | 0.07 | 0.5 | 0.14 | 0.03 | 0.07 | 0.03 | 0.06 | | 0.08 | 0.5 | 1.9 | 88.78 | 92.50 | -3.72 |
| 246 | 12 | 0.8 | 0.06 | 0.14 | 0.03 | 0.4 | 0.03 | 0.5 | 0.06 | 0.07 | 0.07 | 0.08 | 0.06 | | 0.08 | 1 | 1.8 | 84.52 | 84.83 | -0.5249 |
| 247 | 12 | 0.8 | 0.06 | 0.14 | 0.08 | 1 | 0.07 | 0.5 | 0.14 | 0.07 | 0.03 | 0.03 | 0.06 | | 0.08 | 0.5 | 1.6 | 76.85 | 68.99 | 7.86 |
| 248 | 8 | 1.2 | 0.14 | 0.06 | 0.08 | 1 | 0.07 | 1 | 0.14 | 0.03 | 0.07 | 0.08 | 0.04 | | 0.08 | 0.5 | 1.9 | 88.52 | 87.21 | 1.31 |
| 249 | 8 | 1.2 | 0.06 | 0.14 | 0.08 | 0.4 | 0.07 | 1 | 0.14 | 0.03 | 0.07 | 0.03 | 0.06 | | 0.08 | 1 | 1.5 | 91.32 | 89.94 | 1.38 |
| 250 | 8 | 0.8 | 0.14 | 0.14 | 0.03 | 1 | 0.07 | 0.5 | 0.06 | 0.07 | 0.03 | 0.03 | 0.06 | | 0.06 | 0.5 | 2.0 | 68.74 | 67.70 | 1.04 |
| 251 | 10 | 1 | 0.1 | 0.1 | 0.055 | 0.7 | 0.05 | 0.25 | 0.1 | 0.05 | 0.05 | 0.055 | 0.05 | | 0.07 | 0.75 | 2.0 | 84.41 | 85.85 | -1.54 |
| 252 | 12 | 1.2 | 0.14 | 0.14 | 0.03 | 0.4 | 0.03 | 1 | 0.06 | 0.07 | 0.03 | 0.03 | 0.06 | | 0.08 | 0.5 | 1.4 | 68.74 | 65.73 | 3.01 |
| 253 | 10 | 1 | 0.1 | 0.1 | 0.055 | 0.7 | 0.05 | 0.75 | 0.1 | 0.05 | 0.05 | 0.055 | 0.05 | | 0.07 | 0.75 | 1.6 | 92.45 | 91.71 | 1.24 |
| 254 | 8 | 1.2 | 0.14 | 0.06 | 0.03 | 0.4 | 0.03 | 1 | 0.06 | 0.03 | 0.03 | 0.03 | 0.04 | | 0.08 | 1 | 1.8 | 67.45 | 59.89 | 7.56 |
| 255 | 8 | 1.2 | 0.14 | 0.06 | 0.03 | 0.4 | 0.07 | 0.5 | 0.06 | 0.07 | 0.07 | 0.08 | 0.06 | | 0.08 | 1 | 1.8 | 70.32 | 64.53 | 5.59 |
| 256 | 10 | 1 | 0.1 | 0.1 | 0.055 | 0.7 | 0.05 | 0.75 | 0.1 | 0.05 | 0.05 | 0.055 | 0.05 | | 0.07 | 0.75 | 1.9 | 86.45 | 90.45 | -4.00 |
| 257 | 10 | 1 | 0.1 | 0.1 | 0.055 | 0.7 | 0.05 | 0.75 | 0.1 | 0.05 | 0.05 | 0.005 | 0.05 | | 0.07 | 0.75 | 2.0 | 88.52 | 88.43 | 0.0867 |
| 258 | 12 | 1.2 | 0.14 | 0.14 | 0.08 | 1 | 0.03 | 1 | 0.14 | 0.03 | 0.03 | 0.08 | 0.06 | | 0.06 | 0.5 | 2.0 | 60.47 | 61.42 | -0.9468 |
| 259 | 8 | 1.2 | 0.14 | 0.14 | 0.03 | 1 | 0.07 | 0.5 | 0.14 | 0.03 | 0.07 | 0.08 | 0.06 | | 0.06 | 0.5 | 2.0 | 88.78 | 91.69 | -2.91 |
| 260 | 12 | 1.2 | 0.14 | 0.06 | 0.08 | 0.4 | 0.07 | 1 | 0.06 | 0.03 | 0.07 | 0.08 | 0.06 | | 0.06 | 0.5 | 1.9 | 59.99 | 57.68 | 2.31 |
| 261 | 8 | 0.8 | 0.14 | 0.06 | 0.08 | 0.4 | 0.03 | 0.5 | 0.06 | 0.07 | 0.03 | 0.03 | 0.04 | | 0.08 | 1 | 1.8 | 88.78 | 79.04 | -0.2637 |
| 262 | 8 | 0.8 | 0.06 | 0.06 | 0.03 | 0.4 | 0.07 | 1 | 0.06 | 0.07 | 0.03 | 0.03 | 0.06 | | 0.08 | 0.5 | 1.9 | 88.12 | 88.09 | -0.0189 |
| 263 | 12 | 0.8 | 0.06 | 0.14 | 0.08 | 0.4 | 0.07 | 1 | 0.14 | 0.03 | 0.07 | 0.03 | 0.06 | | 0.06 | 0.5 | 1.7 | 54.00 | 62.13 | -6.23 |
| 264 | 12 | 0.8 | 0.06 | 0.06 | 0.03 | 1 | 0.03 | 0.5 | 0.14 | 0.03 | 0.07 | 0.08 | 0.06 | | 0.06 | 0.5 | 1.7 | 56.37 | 57.75 | -1.38 |
| 265 | 8 | 0.8 | 0.14 | 0.06 | 0.08 | 1 | 0.03 | 0.5 | 0.06 | 0.03 | 0.07 | 0.08 | 0.06 | | 0.08 | 0.5 | 1.2 | 60.70 | 57.42 | 3.28 |
| 266 | 8 | 0.8 | 0.14 | 0.14 | 0.08 | 1 | 0.07 | 1 | 0.14 | 0.07 | 0.07 | 0.08 | 0.06 | | 0.06 | 0.5 | 1.8 | 58.74 | 61.91 | -2.27 |
| 267 | 14 | 1 | 0.1 | 0.1 | 0.055 | 0.7 | 0.05 | 0.75 | 0.1 | 0.05 | 0.05 | 0.055 | 0.05 | | 0.07 | 0.75 | 1.8 | 86.37 | 89.10 | -2.73 |
| 268 | 12 | 1.2 | 0.06 | 0.14 | 0.08 | 0.4 | 0.07 | 1 | 0.06 | 0.07 | 0.03 | 0.08 | 0.06 | | 0.06 | 0.5 | 1.7 | 88.24 | 86.56 | -0.3431 |
| 269 | 8 | 1.2 | 0.06 | 0.14 | 0.08 | 1 | 0.07 | 0.5 | 0.14 | 0.07 | 0.03 | 0.03 | 0.06 | | 0.06 | 1 | 1.8 | 87.25 | 87.15 | 0.2031 |
| 270 | 10 | 1 | 0.1 | 0.1 | 0.055 | 0.1 | 0.05 | 0.75 | 0.1 | 0.05 | 0.05 | 0.055 | 0.05 | | 0.07 | 0.75 | 1.7 | 82.45 | 83.48 | -1.03 |
| 271 | 12 | 1.2 | 0.06 | 0.14 | 0.03 | 1 | 0.07 | 0.5 | 0.14 | 0.03 | 0.03 | 0.08 | 0.04 | | 0.06 | 1 | 1.8 | 68.45 | 64.17 | 4.10 |
| 272 | 8 | 1.2 | 0.06 | 0.06 | 0.03 | 0.4 | 0.07 | 0.5 | 0.14 | 0.03 | 0.07 | 0.03 | 0.04 | | 0.06 | 1 | 1.7 | 87.45 | 93.49 | -6.04 |
| 273 | 12 | 0.8 | 0.14 | 0.14 | 0.08 | 0.4 | 0.03 | 1 | 0.06 | 0.03 | 0.03 | 0.08 | 0.04 | | 0.06 | 1 | 2.0 | 56.34 | 55.30 | 1.04 |
| 274 | 10 | 1 | 0.1 | 0.18 | 0.055 | 0.7 | 0.05 | 0.75 | 0.1 | 0.05 | 0.05 | 0.055 | 0.05 | | 0.07 | 0.75 | 2.1 | 88.07 | 88.42 | -0.3473 |
| 275 | 8 | 0.8 | 0.14 | 0.06 | 0.08 | 1 | 0.07 | 1 | 0.06 | 0.07 | 0.03 | 0.03 | 0.04 | | 0.08 | 0.5 | 1.9 | 58.75 | 63.70 | -4.95 |
| 276 | 8 | 1.2 | 0.14 | 0.06 | 0.03 | 1 | 0.07 | 1 | 0.06 | 0.03 | 0.03 | 0.08 | 0.06 | | 0.06 | 1 | 1.7 | 64.24 | 56.74 | -3.50 |
| 277 | 8 | 0.8 | 0.06 | 0.14 | 0.08 | 0.4 | 0.07 | 0.5 | 0.06 | 0.07 | 0.03 | 0.03 | 0.04 | | 0.06 | 0.5 | 2.1 | 88.66 | 86.65 | 1.01 |
| 278 | 8 | 0.8 | 0.06 | 0.14 | 0.03 | 0.4 | 0.03 | 0.5 | 0.14 | 0.03 | 0.03 | 0.03 | 0.06 | | 0.06 | 0.5 | 1.8 | 84.22 | 85.14 | 3.00 |
| 279 | 12 | 0.8 | 0.06 | 0.14 | 0.08 | 1 | 0.03 | 0.5 | 0.14 | 0.03 | 0.07 | 0.03 | 0.06 | | 0.06 | 1 | 2.0 | 90.16 | 92.20 | -2.98 |
| 280 | 8 | 0.8 | 0.06 | 0.06 | 0.08 | 1 | 0.03 | 1 | 0.14 | 0.07 | 0.07 | 0.08 | 0.06 | | 0.08 | 1 | 1.9 | 43.47 | 58.66 | 2.61 |
| 281 | 8 | 1.2 | 0.14 | 0.06 | 0.08 | 0.4 | 0.07 | 0.5 | 0.14 | 0.07 | 0.03 | 0.08 | 0.04 | | 0.06 | 0.5 | 1.8 | 83.28 | 84.91 | 1.40 |
| 282 | 8 | 1.2 | 0.06 | 0.14 | 0.03 | 0.4 | 0.07 | 0.5 | 0.06 | 0.03 | 0.03 | 0.08 | 0.06 | | 0.08 | 1 | 1.7 | 84.44 | 90.52 | -1.84 |
| 283 | 12 | 0.8 | 0.14 | 0.14 | 0.03 | 0.4 | 0.07 | 1 | 0.14 | 0.07 | 0.03 | 0.08 | 0.06 | | 0.06 | 1 | 1.8 | 61.23 | 64.76 | 3.47 |
| 284 | 12 | 1.2 | 0.14 | 0.14 | 0.03 | 0.4 | 0.07 | 1 | 0.06 | 0.03 | 0.07 | 0.03 | 0.06 | | 0.06 | 1 | 1.6 | 84.58 | 85.26 | -0.8240 |
| 285 | 8 | 1.2 | 0.14 | 0.06 | 0.08 | 1 | 0.03 | 0.5 | 0.14 | 0.07 | 0.03 | 0.03 | 0.06 | | 0.08 | 0.5 | 1.7 | 86.59 | 85.57 | -0.3333 |
| 286 | 8 | 1.2 | 0.06 | 0.14 | 0.08 | 0.4 | 0.03 | 1 | 0.14 | 0.07 | 0.03 | 0.03 | 0.06 | | 0.06 | 0.5 | 1.8 | 63.87 | 62.06 | -0.6414 |
| 287 | 8 | 1.2 | 0.14 | 0.14 | 0.08 | 0.4 | 0.07 | 1 | 0.06 | 0.07 | 0.07 | 0.08 | 0.04 | | 0.06 | 1 | 1.8 | 63.52 | 63.49 | -0.5547 |
| 288 | 12 | 1.2 | 0.06 | 0.14 | 0.03 | 0.4 | 0.07 | 0.5 | 0.14 | 0.07 | 0.07 | 0.03 | 0.06 | | 0.06 | 0.5 | 1.5 | 84.78 | 82.70 | 6.08 |
| 289 | 12 | 0.8 | 0.14 | 0.14 | 0.08 | 0.4 | 0.07 | 1 | 0.06 | 0.07 | 0.07 | 0.08 | 0.04 | | 0.08 | 0.5 | 1.9 | 78.11 | 87.45 | -1.34 |
| 290 | 12 | 0.8 | 0.06 | 0.14 | 0.03 | 1 | 0.07 | 0.5 | 0.06 | 0.07 | 0.07 | 0.03 | 0.04 | | 0.06 | 1 | 1.9 | 86.19 | 92.40 | -4.08 |
| 291 | 8 | 0.8 | 0.06 | 0.06 | 0.08 | 1 | 0.07 | 1 | 0.14 | 0.03 | 0.03 | 0.08 | 0.06 | | 0.06 | 0.5 | 1.8 | 84.16 | 88.18 | -4.92 |
| 292 | 12 | 1.2 | 0.06 | 0.06 | 0.03 | 1 | 0.03 | 1 | 0.06 | 0.07 | 0.03 | 0.08 | 0.04 | | 0.08 | 1 | 1.9 | 68.64 | 56.80 | -0.1395 |
| 293 | 8 | 0.8 | 0.14 | 0.14 | 0.03 | 0.4 | 0.07 | 1 | 0.06 | 0.03 | 0.07 | 0.03 | 0.06 | | 0.08 | 0.5 | 1.8 | 61.55 | 52.32 | 0.0366 |
| 294 | 12 | 1.2 | 0.14 | 0.06 | 0.03 | 1 | 0.03 | 1 | 0.14 | 0.03 | 0.03 | 0.03 | 0.06 | | 0.06 | 1 | 1.4 | 78.54 | 81.57 | 2.34 |
| 295 | 8 | 1.2 | 0.06 | 0.14 | 0.08 | 1 | 0.03 | 1 | 0.14 | 0.03 | 0.07 | 0.08 | 0.04 | | 0.06 | 1 | 1.7 | 91.71 | 89.54 | 1.66 |
| 296 | 12 | 1.2 | 0.06 | 0.06 | 0.08 | 0.4 | 0.03 | 1 | 0.14 | 0.03 | 0.03 | 0.03 | 0.04 | | 0.06 | 1 | 2.0 | 62.56 | 61.42 | 0.3541 |
| 297 | 8 | 1.2 | 0.06 | 0.06 | 0.08 | 1 | 0.07 | 0.5 | 0.06 | 0.07 | 0.07 | 0.08 | 0.04 | | 0.08 | 1 | 1.7 | 86.41 | 84.74 | 0.5312 |
| 298 | 8 | 0.8 | 0.14 | 0.14 | 0.08 | 0.4 | 0.07 | 0.5 | 0.14 | 0.03 | 0.03 | 0.08 | 0.06 | | 0.08 | 0.5 | 1.8 | 62.42 | 62.32 | 1.27 |

**S Table 3.**

**Regression analysis using central composite design (CCD)**

| **Source** | **Sum of Squares** | **df** | **Mean Square** | **F-value** | **p-value** |  |
| --- | --- | --- | --- | --- | --- | --- |
| **Model** | 65475.61 | 141 | 439.20 | 21.68 | < 0.0001 | significant |
| A-Glucose concentration | 3.45 | 1 | 3.45 | 0.1458 | 0.6244 |  |
| B-Urea concentration | 1.01 | 1 | 1.01 | 0.0248 | 0.8234 |  |
| C-K_2_HPO_4_ concentration | 24.11 | 1 | 24.11 | 1.11 | 0.2929 |  |
| D-KH_2_PO_4_ concentration | 13.45 | 1 | 13.45 | 0.6227 | 0.4376 |  |
| E-MgSO_4_.7H_2_O concentration | 24.09 | 1 | 24.09 | 1.02 | 0.3155 |  |
| F-KCl concentration | 54.14 | 1 | 54.14 | 2.61 | 0.1074 |  |
| G-CoCl_2_.6H_2_O concentration | 51.97 | 1 | 51.97 | 2.12 | 0.1524 |  |
| H-NH_4_VO_2_ concentration | 16.34 | 1 | 16.34 | 0.7578 | 0.3578 |  |
| J-Na_2_MoO_4_.2H_2_O concentration | 3.72 | 1 | 3.72 | 0.1694 | 0.6182 |  |
| K-CaCO_3_ concentration | 52.56 | 1 | 52.56 | 2.34 | 0.1165 |  |
| L-FeSO_4_.7H_2_O concentration | 6.34 | 1 | 6.34 | 0.2870 | 0.5764 |  |
| M-ZnSO_4_.7H_2_O concentration | 0.2577 | 1 | 0.2577 | 0.0348 | 0.9577 |  |
| N-MnSO_4_.4H_2_O concentration | 16.21 | 1 | 16.21 | 0.7662 | 0.3768 |  |
| O-NiSO_4_.7H_2_O concentration | 34.54 | 1 | 34.54 | 1.58 | 0.2255 |  |
| P-Na_2_B_4_O_7_.10H_2_O concentration | 10.24 | 1 | 10.24 | 0.4454 | 0.4178 |  |
| Q-Dry Cell Weight | 24370.69 | 1 | 24370.69 | 1146.34 | < 0.0001 |  |
| AB | 0.0514 | 1 | 0.0514 | 0.0025 | 0.9584 |  |
| AC | 24.30 | 1 | 24.30 | 1.26 | 0.2621 |  |
| AD | 10.24 | 1 | 10.2 | 0.4475 | 0.4902 |  |
| AE | 0.0000 | 1 | 0.0000 | 6.489E-07 | 0.9927 |  |
| AF | 5.27 | 1 | 5.27 | 0.2483 | 0.6247 |  |
| AG | 21.24 | 1 | 21.24 | 0.9270 | 0.3214 |  |
| AH | 0.0541 | 1 | 0.0541 | 0.0054 | 0.9623 |  |
| AJ | 23.34 | 1 | 23.34 | 1.01 | 0.2578 |  |
| AK | 84.14 | 1 | 84.14 | 3.71 | 0.0178 |  |
| AL | 6.22 | 1 | 6.22 | 0.2548 | 0.5247 |  |
| AM | 2.47 | 1 | 2.47 | 0.1528 | 0.7332 |  |
| AN | 1.88 | 1 | 1.88 | 0.0125 | 0.7854 |  |
| AO | 54.12 | 1 | 54.12 | 2.50 | 0.1657 |  |
| AP | 4.14 | 1 | 4.14 | 0.2278 | 0.6256 |  |
| AQ | 4.33 | 1 | 4.33 | 0.2809 | 0.6474 |  |
| BC | 2.47 | 1 | 2.47 | 0.1474 | 0.7253 |  |
| BD | 2.04 | 1 | 2.04 | 0.0644 | 0.7786 |  |
| BE | 45.24 | 1 | 45. 27 | 2.12 | 0.1379 |  |
| BF | 99.20 | 1 | 99.20 | 4.61 | 0.0547 |  |
| BG | 137.65 | 1 | 137.65 | 5.11 | 0.0577 |  |
| BH | 34.28 | 1 | 34.28 | 1.84 | 0.1783 |  |
| BJ | 24.59 | 1 | 24.59 | 1.13 | 0.2856 |  |
| BK | 48.52 | 1 | 48.52 | 2.24 | 0.1322 |  |
| BL | 2.64 | 1 | 2.64 | 0.1474 | 0.7437 |  |
| BM | 83.12 | 1 | 83.12 | 3.81 | 0.0547 |  |
| BN | 0.2843 | 1 | 0.2843 | 0.0248 | 0.9068 |  |
| BO | 1.25 | 1 | 1.25 | 0.0545 | 0.7657 |  |
| BP | 0.0540 | 1 | 0.0540 | 0.0249 | 0.9624 |  |
| BQ | 0.0324 | 1 | 0.0324 | 0.0175 | 0.9654 |  |
| CD | 15.11 | 1 | 15.11 | 0.7074 | 0.4066 |  |
| CE | 23.26 | 1 | 23.26 | 1.14 | 0.2247 |  |
| CF | 0.0523 | 1 | 0.0523 | 0.0878 | 0.9327 |  |
| CG | 20.07 | 1 | 20.07 | 0.9270 | 0.3548 |  |
| CH | 9.71 | 1 | 9.71 | 0.4746 | 0.5876 |  |
| CJ | 29.44 | 1 | 29.44 | 1.30 | 0.2749 |  |
| CK | 33.52 | 1 | 33.52 | 1.52 | 0.2223 |  |
| CL | 10.74 | 1 | 10.74 | 0.4717 | 0.4669 |  |
| CM | 24.23 | 1 | 24.23 | 1.11 | 0.2540 |  |
| CN | 31.32 | 1 | 31.32 | 1.47 | 0.2259 |  |
| CO | 111.06 | 1 | 111.06 | 4.19 | 0.0671 |  |
| CP | 4.25 | 1 | 4.25 | 0.2228 | 0.6874 |  |
| CQ | 0.0456 | 1 | 0.0456 | 0.0175 | 0.9450 |  |
| DE | 7.54 | 1 | 7.54 | 0.3342 | 0.5568 |  |
| DF | 16.22 | 1 | 16.22 | 0.7454 | 0.3454 |  |
| DG | 10.26 | 1 | 10.26 | 0.4245 | 0.4782 |  |
| DH | 4.16 | 1 | 4.16 | 0.2124 | 0.6574 |  |
| DJ | 4.52 | 1 | 4.52 | 0.2041 | 0.6255 |  |
| DK | 0.0074 | 1 | 0.0074 | 0.0012 | 0.9315 |  |
| DL | 166.52 | 1 | 166.52 | 7.32 | 0.0634 |  |
| DM | 2.17 | 1 | 2.17 | 0.1542 | 0.7524 |  |
| DN | 66.45 | 1 | 66.45 | 3.05 | 0.0874 |  |
| DO | 10.52 | 1 | 10.52 | 0.4744 | 0.4743 |  |
| DP | 25.44 | 1 | 25.44 | 1.19 | 0.2445 |  |
| DQ | 1.50 | 1 | 1.50 | 0.0241 | 0.8056 |  |
| EF | 1.31 | 1 | 1.31 | 0.0120 | 0.8136 |  |
| EG | 24.34 | 1 | 24.34 | 1.31 | 0.2524 |  |
| EH | 142.35 | 1 | 142.35 | 7.45 | 0.0067 |  |
| EJ | 0.6451 | 1 | 0.6451 | 0.0174 | 0.8665 |  |
| EK | 10.54 | 1 | 10.54 | 0.4252 | 0.4559 |  |
| EL | 6.33 | 1 | 6.33 | 0.3749 | 0.5885 |  |
| EM | 22.24 | 1 | 22.24 | 1.05 | 0.3338 |  |
| EN | 1.37 | 1 | 1.37 | 0.0644 | 0.8245 |  |
| EO | 48.52 | 1 | 48.52 | 2.26 | 0.1349 |  |
| EP | 8.47 | 1 | 8.47 | 0.3794 | 0.5389 |  |
| EQ | 3.45 | 1 | 3.45 | 0.1487 | 0.7004 |  |
| FG | 17.73 | 1 | 17.73 | 0.7550 | 0.3876 |  |
| FH | 123.42 | 1 | 123.42 | 6.12 | 0.0246 |  |
| FJ | 12.21 | 1 | 12.21 | 0.5163 | 0.4245 |  |
| FK | 14.73 | 1 | 14.73 | 0.5173 | 0.4331 |  |
| FL | 37.75 | 1 | 37.75 | 1.65 | 0.1573 |  |
| FM | 3.64 | 1 | 3.64 | 0.1553 | 0.6776 |  |
| FN | 2.25 | 1 | 2.25 | 0.1379 | 0.7245 |  |
| FO | 67.79 | 1 | 67.79 | 3.23 | 0.0538 |  |
| FP | 24.44 | 1 | 24.44 | 1.13 | 0.2526 |  |
| FQ | 11.41 | 1 | 11.41 | 0.5748 | 0.4656 |  |
| GH | 0.8454 | 1 | 0.8454 | 0.0385 | 0.8464 |  |
| GJ | 75.22 | 1 | 75.22 | 3.47 | 0.0647 |  |
| GK | 0.6786 | 1 | 0.6786 | 0.0234 | 0.8537 |  |
| GL | 0.0055 | 1 | 0.0055 | 0.0345 | 0.9822 |  |
| GM | 48.37 | 1 | 48.37 | 2.22 | 0.1387 |  |
| GN | 1.34 | 1 | 1.34 | 0.0576 | 0.8174 |  |
| GO | 3.48 | 1 | 3.48 | 0.1442 | 0.6934 |  |
| GP | 0.4645 | 1 | 0.4645 | 0.0575 | 0.8855 |  |
| GQ | 6.23 | 1 | 6.23 | 0.2550 | 0.5967 |  |
| HJ | 1.81 | 1 | 1.81 | 0.0258 | 0.7547 |  |
| HK | 43.24 | 1 | 43.24 | 2.00 | 0.1554 |  |
| HL | 0.7271 | 1 | 0.7271 | 0.0585 | 0.8574 |  |
| HM | 42.87 | 1 | 42.87 | 1.68 | 0.1632 |  |
| HN | 0.6534 | 1 | 0.6534 | 0.0212 | 0.8619 |  |
| HO | 0.4540 | 1 | 0.4540 | 0.0274 | 0.8822 |  |
| HP | 0.4474 | 1 | 0.4474 | 0.0549 | 0.8818 |  |
| HQ | 19.32 | 1 | 19.32 | 0.8548 | 0.3420 |  |
| JK | 26.74 | 1 | 26.74 | 1.47 | 0.2616 |  |
| JL | 28.54 | 1 | 28.54 | 1.37 | 0.2519 |  |
| JM | 3.62 | 1 | 3.62 | 0.1555 | 0.6952 |  |
| JN | 25.54 | 1 | 25.54 | 1.17 | 0.2843 |  |
| JO | 13.31 | 1 | 13.31 | 0.6334 | 0.4567 |  |
| JP | 20.74 | 1 | 20.74 | 0.9322 | 0.3454 |  |
| JQ | 11.52 | 1 | 11.52 | 0.5877 | 0.4574 |  |
| KL | 0.4475 | 1 | 0.4475 | 1.78 | 0.8339 |  |
| KM | 3.27 | 1 | 3.27 | 0.1433 | 0.7551 |  |
| KN | 31.52 | 1 | 31.52 | 1.48 | 0.2520 |  |
| KO | 5.51 | 1 | 5.51 | 0.2547 | 0.6672 |  |
| KP | 1.64 | 1 | 1.64 | 0.0555 | 0.7442 |  |
| KQ | 63.72 | 1 | 63.72 | 2.12 | 0.0815 |  |
| LM | 34.34 | 1 | 34.34 | 1.54 | 0.2045 |  |
| LN | 106.74 | 1 | 106.74 | 4.77 | 0.0237 |  |
| LO | 2.54 | 1 | 2.54 | 0.1745 | 0.7228 |  |
| LP | 14.28 | 1 | 14.28 | 0.6972 | 0.4117 |  |
| LQ | 168.41 | 1 | 168.41 | 7.32 | 0.0022 |  |
| MN | 1.52 | 1 | 1.52 | 0.0288 | 0.8342 |  |
| MO | 3.64 | 1 | 3.64 | 0.1259 | 0.6573 |  |
| MP | 11.52 | 1 | 11.52 | 0.5424 | 0.4664 |  |
| MQ | 0.4234 | 1 | 0.4234 | 0.0153 | 0.8859 |  |
| NO | 4.37 | 1 | 4.37 | 0.2064 | 0.6542 |  |
| NP | 18.52 | 1 | 18.52 | 0.8487 | 0.3657 |  |
| NQ | 25.21 | 1 | 25.21 | 1.54 | 0.2782 |  |
| OP | 43.47 | 1 | 43.47 | 2.33 | 0.1245 |  |
| OQ | 10.21 | 1 | 10.21 | 0.4828 | 0.4372 |  |
| PQ | 19.47 | 1 | 19.47 | 0.8955 | 0.3568 |  |
| A² | 7.15 | 1 | 7.15 | 0.3447 | 0.5589 |  |
| B² | 53.27 | 1 | 53.27 | 2.86 | 0.1220 |  |
| C² | 0.0414 | 1 | 0.0414 | 0.0023 | 0.9661 |  |
| D² | 22.15 | 1 | 22.15 | 1.13 | 0.3152 |  |
| E² | 21.15 | 1 | 21.15 | 0.9317 | 0.3367 |  |
| F² | 98.32 | 1 | 98.32 | 4.44 | 0.0378 |  |
| G² | 4.35 | 1 | 4.35 | 0.1541 | 0.6542 |  |
| H² | 72.64 | 1 | 72.64 | 3.26 | 0.0369 |  |
| J² | 8.24 | 1 | 8.24 | 0.3448 | 0.5544 |  |
| K² | 1.34 | 1 | 1.34 | 0.0725 | 0.7789 |  |
| L² | 72.55 | 1 | 72.55 | 3.18 | 0.0482 |  |
| M² | 15.64 | 1 | 15.64 | 0.7144 | 0.3666 |  |
| N² | 17.72 | 1 | 17.72 | 0.8231 | 0.3794 |  |
| O² | 1.82 | 1 | 1.82 | 0.0541 | 0.7453 |  |
| P² | 4.32 | 1 | 4.32 | 0.2521 | 0.6526 |  |
| Q² | 1789.33 | 1 | 1789.33 | 87.19 | < 0.0001 |  |
| **Residual** | 3134.12 | 213 | 21.67 |  |  |  |
| Lack of Fit | 3374.32 | 126 | 22.40 | 2.10 | 0.1107 | not significant |
| Pure Error | 94.79 | 9 | 10.74 |  |  |  |
| **Cor Total** | 72477.71 | 527 |  |  |  |  |

Factor coding is **Coded**.
Sum of squares is **Type III - Partial**

The **Model F-value** of 20.68 implies the model is significant. There is only a 0.01% chance that an F-value this large could occur due to noise.

**P-values** less than 0.0500 indicate model terms are significant. In this case Q, AK, BF, BG, CO, DL, EH, FH, LN, LQ, F², Q² are significant model terms. Values greater than 0.1000 indicate the model terms are not significant. If there are many insignificant model terms (not counting those required to support hierarchy), model reduction may improve your model.

The **Lack of Fit F-value** of 2.10 implies the Lack of Fit is not significant relative to the pure error. There is a 11.07% chance that a Lack of Fit F-value this large could occur due to noise. Non-significant lack of fit is good -- we want the model to fit.

**S Table 4:**

**Fit Statistics**

| **Std. Dev.** | 4.45 |  | **R²** | 0.9329 |
| --- | --- | --- | --- | --- |
| **Mean** | 75.46 |  | **Adjusted R²** | 0.9134 |
| **C.V. %** | 6.17 |  | **Predicted R²** | 0.8277 |
|  |  |  | **Adeq Precision** | 17.5463 |

**S Table 5:**

Report

| **Run Order** | **Actual Value** | **Predicted Value** | **Residual** | **Leverage** | **Internally Studentized Residuals** | **Externally Studentized Residuals** | **Cook's Distance** | **Influence on Fitted Value DFFITS** | **Standard Order** |
| --- | --- | --- | --- | --- | --- | --- | --- | --- | --- |
| 1 | 90.63 | 88.39 | 3.24 | 0.533 | 1.002 | 1.002 | 0.008 | 1.013 | 287 |
| 2 | 65.37 | 61.88 | 3.87 | 0.533 | 1.084 | 1.087 | 0.004 | 1.145 | 67 |
| 3 | 54.26 | 57.78 | -1.69 | 0.533 | -0.531 | -0.528 | 0.002 | -0.566 | 3 |
| 4 | 87.55 | 85.31 | 4.36 | 0.533 | 1.360 | 1.361 | 0.014 | 1.452 | 230 |
| 5 | 56.33 | 55.88 | 0.4334 | 0.533 | 0.174 | 0.144 | 0.000 | 0.147 | 201 |
| 6 | 88.46 | 89.34 | -0.9966 | 0.533 | -0.313 | -0.312 | 0.002 | -0.334 | 21 |
| 7 | 86.20 | 89.00 | -4.79 | 0.533 | -1.508 | -1.514 | 0.014 | -1.619 | 250 |
| 8 | 62.44 | 63.37 | 0.9957 | 0.533 | 0.312 | 0.315 | 0.001 | 0.328 | 47 |
| 9 | 88.28 | 88.25 | -3.01 | 0.533 | -0.945 | -0.945 | 0.005 | -1.010 | 56 |
| 10 | 87.12 | 90.08 | -2.00 | 0.533 | -0.629 | -0.624 | 0.003 | -0.649 | 44 |
| 11 | 88.55 | 86.90 | -0.4366 | 0.533 | -0.137 | -0.136 | 0.000 | -0.168 | 8 |
| 12 | 52.37 | 58.43 | -1.96 | 0.533 | -0.615 | -0.613 | 0.003 | -0.647 | 76 |
| 13 | 55.74 | 61.39 | -1.44 | 0.533 | -0.519 | -0.522 | 0.002 | -0.559 | 196 |
| 14 | 87.74 | 87.43 | 0.3079 | 0.533 | 0.063 | 0.096 | 0.000 | 0.154 | 264 |
| 15 | 86.21 | 89.55 | -2.30 | 0.533 | -0.747 | -0.734 | 0.003 | -0.771 | 147 |
| 16 | 90.24 | 88.63 | 1.41 | 0.533 | 0.500 | 0.499 | 0.002 | 0.536 | 69 |
| 17 | 61.47 | 64.44 | -1.01 | 0.533 | -0.361 | -0.332 | 0.001 | -0.347 | 198 |
| 18 | 88.41 | 85.67 | 1.41 | 0.533 | 0.520 | 0.518 | 0.002 | 0.554 | 252 |
| 19 | 53.34 | 55.56 | 1.3 | 0.533 | 0.341 | 0.340 | 0.001 | 0.352 | 150 |
| 20 | 90.32 | 89.89 | 0.2274 | 0.533 | 0.055 | 0.077 | 0.000 | 0.041 | 192 |
| 21 | 89.66 | 87.52 | 2.10 | 0.533 | 0.659 | 0.658 | 0.003 | 0.704 | 242 |
| 22 | 92.25 | 94.33 | -2.09 | 0.533 | -0.656 | -0.655 | 0.003 | -0.700 | 49 |
| 23 | 88.47 | 89.70 | -0.7241 | 0.533 | -0.207 | -0.224 | 0.000 | -0.252 | 121 |
| 24 | 88.39 | 88.44 | -0.0238 | 0.533 | -0.023 | -0.012 | 0.000 | -0.032 | 150 |
| 25 | 89.74 | 88.02 | 0.7621 | 0.533 | 0.247 | 0.234 | 0.000 | 0.255 | 126 |
| 26 | 88.32 | 87.98 | -1.61 | 0.533 | -0.531 | -0.508 | 0.002 | -0.543 | 157 |
| 27 | 48.49 | 53.84 | -7.51 | 0.533 | -2.324 | -2.330 | 0.040 | -2.499⁽¹⁾ | 72 |
| 28 | 88.52 | 88.88 | 1.68 | 0.533 | 0.537 | 0.522 | 0.002 | 0.562 | 233 |
| 29 | 88.04 | 87.78 | -0.7314 | 0.533 | -0.247 | -0.226 | 0.000 | -0.240 | 51 |
| 30 | 82.56 | 88.55 | -3.47 | 0.533 | -1.227 | -1.211 | 0.011 | -1.307 | 222 |
| 31 | 86.74 | 92.80 | -3.05 | 0.533 | -1.237 | -1.231 | 0.012 | -1.362 | 212 |
| 32 | 82.23 | 85.27 | -1.05 | 0.533 | -0.392 | -0.317 | 0.001 | -0.351 | 153 |
| 33 | 88.23 | 86.48 | -0.3508 | 0.533 | -0.127 | -0.107 | 0.000 | -0.118 | 151 |
| 34 | 62.52 | 62.03 | 0.4624 | 0.533 | 0.164 | 0.150 | 0.000 | 0.143 | 60 |
| 35 | 90.14 | 88.33 | 1.48 | 0.533 | 0.548 | 0.542 | 0.002 | 0.587 | 127 |
| 36 | 58.43 | 58.76 | -0.3238 | 0.533 | -0.059 | -0.053 | 0.000 | -0.101 | 195 |
| 37 | 90.67 | 91.92 | -1.210 | 0.504 | -0.307 | -0.357 | 0.001 | -0.372 | 231 |
| 38 | 86.88 | 85.51 | 1.41 | 0.533 | 0.486 | 0.462 | 0.002 | 0.491 | 221 |
| 39 | 84.70 | 84.52 | 0.2377 | 0.533 | 0.087 | 0.078 | 0.000 | 0.083 | 142 |
| 40 | 87.55 | 87.54 | -0.1240 | 0.533 | -0.024 | -0.024 | 0.000 | -0.047 | 77 |
| 41 | 82.78 | 92.44 | -7.70 | 0.533 | -2.442 | -2.467 | 0.044 | -2.636⁽¹⁾ | 231 |
| 42 | 83.64 | 87.66 | -1.92 | 0.533 | -0.634 | -0.634 | 0.003 | -0.655 | 228 |
| 43 | 67.45 | 62.72 | 6.17 | 0.533 | 1.961 | 1.744 | 0.027 | 2.068 | 13 |
| 44 | 84.52 | 85.46 | 1.19 | 0.504 | 0.346 | 0.313 | 0.001 | 0.326 | 214 |
| 45 | 54.34 | 76.62 | -18.28 | 0.533 | -5.784 | -6.519⁽²⁾ | 0.247 | -6.967⁽¹⁾ | 130 |
| 46 | 50.34 | 53.97 | -1.21 | 0.533 | -0.531 | -0.552 | 0.002 | -0.570 | 137 |
| 47 | 59.67 | 59.14 | -1.49 | 0.533 | -0.407 | -0.463 | 0.002 | -0.501 | 233 |
| 48 | 63.26 | 66.38 | -0.1504 | 0.533 | -0.044 | -0.021 | 0.000 | -0.047 | 114 |
| 49 | 62.44 | 67.69 | -1.23 | 0.533 | -0.319 | -0.388 | 0.001 | -0.416 | 137 |
| 50 | 91.30 | 90.64 | -2.37 | 0.533 | -0.713 | -0.703 | 0.004 | -0.778 | 90 |
| 51 | 92.76 | 87.47 | 4.90 | 0.533 | 1.540 | 1.554 | 0.018 | 1.608 | 203 |
| 52 | 68.70 | 64.82 | 3.94 | 0.533 | 1.221 | 1.228 | 0.011 | 1.331 | 31 |
| 53 | 64.43 | 58.66 | 4.41 | 0.533 | 1.296 | 1.320 | 0.013 | 1.389 | 54 |
| 54 | 89.99 | 91.21 | -0.0640 | 0.504 | -0.005 | -0.005 | 0.000 | -0.005 | 284 |
| 55 | 88.24 | 84.54 | 1.82 | 0.533 | 0.522 | 0.511 | 0.002 | 0.610 | 178 |
| 56 | 91.37 | 90.02 | 2.525 | 0.533 | 0.719 | 0.708 | 0.004 | 0.748 | 137 |
| 57 | 90.64 | 91.24 | -1.74 | 0.533 | -0.549 | -0.541 | 0.002 | -0.585 | 45 |
| 58 | 59.23 | 52.35 | 7.58 | 0.533 | 2.477 | 2.520 | 0.046 | 2.696⁽¹⁾ | 190 |
| 59 | 87.45 | 86.23 | 0.0217 | 0.533 | 0.001 | 0.001 | 0.000 | 0.001 | 189 |
| 60 | 89.79 | 88.87 | -0.0576 | 0.533 | -0.026 | -0.034 | 0.000 | -0.068 | 7 |
| 61 | 91.14 | 93.61 | -0.8743 | 0.533 | -0.271 | -0.254 | 0.001 | -0.233 | 107 |
| 62 | 87.97 | 91.21 | -2.24 | 0.036 | -0.490 | -0.494 | 0.000 | -0.094 | 296 |
| 63 | 85.01 | 91.12 | -5.20 | 0.036 | -1.131 | -1.109 | 0.000 | -0.224 | 281 |
| 64 | 87.14 | 87.23 | 0.9455 | 0.533 | 0.288 | 0.287 | 0.001 | 0.306 | 127 |
| 65 | 63.34 | 57.54 | 1.81 | 0.533 | 0.546 | 0.564 | 0.002 | 0.631 | 41 |
| 66 | 65.47 | 62.42 | 5.15 | 0.533 | 1.589 | 1.562 | 0.019 | 1.708 | 99 |
| 67 | 89.12 | 92.16 | -1.04 | 0.533 | -0.328 | -0.371 | 0.001 | -0.354 | 137 |
| 68 | 51.37 | 52.23 | 0.1115 | 0.533 | 0.043 | 0.024 | 0.000 | 0.045 | 130 |
| 69 | 54.32 | 56.32 | -0.9645 | 0.533 | -0.315 | -0.312 | 0.001 | -0.345 | 175 |
| 70 | 58.64 | 61.34 | -2.75 | 0.533 | -0.865 | -0.846 | 0.006 | -0.924 | 33 |
| 71 | 86.48 | 89.47 | -2.77 | 0.533 | -0.817 | -0.807 | 0.006 | -0.930 | 200 |
| 72 | 48.67 | 54.41 | -5.56 | 0.533 | -1.779 | -1.793 | 0.024 | -1.916 | 176 |
| 73 | 88.32 | 89.52 | -0.8556 | 0.533 | -0.261 | -0.255 | 0.000 | -0.272 | 192 |
| 74 | 86.57 | 86.64 | 0.2134 | 0.504 | 0.051 | 0.058 | 0.000 | 0.049 | 257 |
| 75 | 94.76 | 91.21 | 3.57 | 0.036 | 0.737 | 0.764 | 0.000 | 0.127 | 268 |
| 76 | 54.27 | 56.58 | -2.24 | 0.533 | -0.766 | -0.756 | 0.004 | -0.818 | 267 |
| 77 | 61.34 | 58.57 | 2.41 | 0.533 | 0.751 | 0.753 | 0.004 | 0.876 | 12 |
| 78 | 60.33 | 62.47 | -2.07 | 0.533 | -0.652 | -0.617 | 0.003 | -0.696 | 287 |
| 79 | 58.76 | 60.15 | -1.36 | 0.533 | -0.474 | -0.428 | 0.001 | -0.447 | 171 |
| 80 | 90.47 | 91.64 | -0.8208 | 0.036 | -0.195 | -0.194 | 0.000 | -0.037 | 224 |
| 81 | 64.37 | 67.74 | -3.05 | 0.533 | -0.924 | -0.944 | 0.007 | -1.031 | 101 |
| 82 | 62.42 | 63.47 | -1.40 | 0.533 | -0.452 | -0.440 | 0.001 | -0.418 | 204 |
| 83 | 87.99 | 85.54 | 2.45 | 0.533 | 0.770 | 0.71 | 0.004 | 0.837 | 241 |
| 84 | 88.64 | 88.57 | 0.0735 | 0.504 | 0.022 | 0.022 | 0.000 | 0.023 | 76 |
| 85 | 86.21 | 90.44 | -4.23 | 0.533 | -1.329 | -1.332 | 0.013 | -1.424 | 117 |
| 86 | 50.24 | 57.07 | -6.83 | 0.533 | -2.147 | -2.175 | 0.034 | -2.325⁽¹⁾ | 26 |
| 87 | 87.95 | 86.51 | 1.44 | 0.533 | 0.452 | 0.451 | 0.002 | 0.482 | 50 |
| 88 | 56.32 | 59.02 | -2.70 | 0.533 | -0.848 | -0.847 | 0.005 | -0.906 | 252 |
| 89 | 89.74 | 89.56 | 0.1765 | 0.504 | 0.054 | 0.054 | 0.000 | 0.054 | 283 |
| 90 | 90.12 | 88.96 | 1.16 | 0.504 | 0.352 | 0.351 | 0.001 | 0.354 | 269 |
| 91 | 62.41 | 63.82 | -1.41 | 0.533 | -0.445 | -0.444 | 0.001 | -0.474 | 4 |
| 92 | 89.45 | 91.21 | -1.76 | 0.036 | -0.385 | -0.384 | 0.000 | -0.074 | 293 |
| 93 | 54.27 | 56.68 | -2.41 | 0.533 | -0.757 | -0.756 | 0.004 | -0.808 | 84 |
| 94 | 92.47 | 91.21 | 1.26 | 0.036 | 0.275 | 0.275 | 0.000 | 0.053 | 290 |
| 95 | 58.74 | 62.60 | -3.86 | 0.533 | -1.215 | -1.217 | 0.011 | -1.301 | 61 |
| 96 | 46.37 | 54.28 | -7.91 | 0.533 | -2.488 | -2.534 | 0.046 | -2.708⁽¹⁾ | 172 |
| 97 | 89.64 | 90.63 | -0.9938 | 0.504 | -0.303 | -0.302 | 0.001 | -0.305 | 270 |
| 98 | 88.21 | 92.90 | -4.69 | 0.533 | -1.474 | -1.480 | 0.016 | -1.582 | 207 |
| 99 | 86.33 | 88.84 | -2.51 | 0.533 | -0.789 | -0.788 | 0.005 | -0.842 | 64 |
| 100 | 52.37 | 53.07 | -0.7025 | 0.533 | -0.221 | -0.220 | 0.000 | -0.235 | 143 |
| 101 | 49.65 | 55.11 | -5.46 | 0.533 | -1.716 | -1.728 | 0.022 | -1.847 | 225 |
| 102 | 64.27 | 62.74 | 1.53 | 0.533 | 0.480 | 0.479 | 0.002 | 0.512 | 178 |
| 103 | 64.21 | 58.35 | 5.86 | 0.533 | 1.842 | 1.857 | 0.025 | 1.985 | 121 |
| 104 | 64.52 | 64.40 | 0.1244 | 0.533 | 0.039 | 0.039 | 0.000 | 0.042 | 65 |
| 105 | 88.47 | 85.35 | 3.12 | 0.533 | 0.981 | 0.980 | 0.007 | 1.048 | 28 |
| 106 | 86.54 | 90.61 | -4.07 | 0.533 | -1.278 | -1.281 | 0.012 | -1.369 | 43 |
| 107 | 87.99 | 84.89 | 3.10 | 0.533 | 0.976 | 0.975 | 0.007 | 1.043 | 103 |
| 108 | 58.74 | 56.50 | 2.24 | 0.533 | 0.704 | 0.703 | 0.004 | 0.751 | 48 |
| 109 | 92.12 | 90.75 | 1.37 | 0.504 | 0.419 | 0.418 | 0.001 | 0.422 | 262 |
| 110 | 56.34 | 58.39 | -2.05 | 0.533 | -0.645 | -0.644 | 0.003 | -0.688 | 136 |
| 111 | 52.31 | 53.17 | -0.8634 | 0.533 | -0.271 | -0.271 | 0.001 | -0.289 | 239 |
| 112 | 88.52 | 89.94 | -1.42 | 0.533 | -0.446 | -0.445 | 0.001 | -0.475 | 159 |
| 113 | 60.12 | 62.25 | -2.13 | 0.533 | -0.671 | -0.669 | 0.003 | -0.716 | 157 |
| 114 | 88.58 | 83.56 | 5.02 | 0.533 | 1.578 | 1.586 | 0.019 | 1.695 | 27 |
| 115 | 88.47 | 89.95 | -1.48 | 0.533 | -0.466 | -0.464 | 0.002 | -0.496 | 44 |
| 116 | 89.24 | 90.75 | -1.51 | 0.533 | -0.475 | -0.474 | 0.002 | -0.507 | 19 |
| 117 | 56.34 | 56.22 | 0.1244 | 0.533 | 0.039 | 0.039 | 0.000 | 0.042 | 232 |
| 118 | 54.02 | 52.09 | 1.93 | 0.533 | 0.608 | 0.606 | 0.003 | 0.648 | 14 |
| 119 | 62.34 | 54.55 | 7.79 | 0.533 | 2.451 | 2.494 | 0.045 | 2.666⁽¹⁾ | 138 |
| 120 | 58.74 | 57.59 | 1.15 | 0.533 | 0.361 | 0.360 | 0.001 | 0.385 | 52 |
| 121 | 92.45 | 89.56 | 2.89 | 0.504 | 0.881 | 0.881 | 0.005 | 0.888 | 257 |
| 122 | 86.22 | 89.01 | -2.79 | 0.504 | -0.852 | -0.851 | 0.005 | -0.858 | 274 |
| 123 | 48.75 | 53.83 | -5.08 | 0.533 | -1.596 | -1.604 | 0.019 | -1.715 | 5 |
| 124 | 58.74 | 56.96 | 1.78 | 0.533 | 0.558 | 0.557 | 0.002 | 0.596 | 91 |
| 125 | 86.55 | 89.96 | -3.41 | 0.533 | -1.071 | -1.071 | 0.009 | -1.145 | 98 |
| 126 | 88.12 | 90.52 | -2.40 | 0.533 | -0.756 | -0.755 | 0.004 | -0.807 | 133 |
| 127 | 89.21 | 86.38 | 2.83 | 0.533 | 0.889 | 0.888 | 0.006 | 0.950 | 142 |
| 128 | 86.56 | 91.21 | -4.65 | 0.036 | -1.018 | -1.018 | 0.000 | -0.196 | 298 |
| 129 | 58.74 | 54.29 | 4.45 | 0.533 | 1.399 | 1.403 | 0.015 | 1.500 | 128 |
| 130 | 56.00 | 55.43 | 0.5704 | 0.533 | 0.179 | 0.179 | 0.000 | 0.191 | 165 |
| 131 | 88.97 | 88.48 | 0.4907 | 0.533 | 0.154 | 0.154 | 0.000 | 0.164 | 190 |
| 132 | 84.72 | 84.87 | -0.1508 | 0.533 | -0.047 | -0.047 | 0.000 | -0.051 | 36 |
| 133 | 62.14 | 61.69 | 0.4455 | 0.533 | 0.140 | 0.140 | 0.000 | 0.149 | 42 |
| 134 | 88.14 | 86.94 | 1.20 | 0.533 | 0.377 | 0.376 | 0.001 | 0.402 | 16 |
| 135 | 86.54 | 87.81 | -1.27 | 0.504 | -0.389 | -0.387 | 0.001 | -0.391 | 282 |
| 136 | 66.34 | 64.04 | 2.30 | 0.533 | 0.723 | 0.721 | 0.004 | 0.771 | 114 |
| 137 | 58.75 | 56.97 | 1.78 | 0.533 | 0.558 | 0.557 | 0.002 | 0.596 | 32 |
| 138 | 67.45 | 61.41 | 6.04 | 0.533 | 1.898 | 1.915 | 0.027 | 2.047 | 251 |
| 139 | 74.12 | 71.04 | 3.08 | 0.533 | 0.968 | 0.968 | 0.007 | 1.034 | 185 |
| 140 | 88.41 | 90.89 | -2.48 | 0.533 | -0.779 | -0.778 | 0.005 | -0.832 | 30 |
| 141 | 87.12 | 86.74 | 0.3794 | 0.533 | 0.119 | 0.119 | 0.000 | 0.127 | 20 |
| 142 | 90.24 | 88.79 | 1.45 | 0.533 | 0.456 | 0.455 | 0.002 | 0.486 | 97 |
| 143 | 92.34 | 91.87 | 0.4689 | 0.533 | 0.147 | 0.147 | 0.000 | 0.157 | 146 |
| 144 | 90.24 | 88.80 | 1.44 | 0.504 | 0.438 | 0.437 | 0.001 | 0.440 | 281 |
| 145 | 88.72 | 84.76 | 3.96 | 0.533 | 1.245 | 1.247 | 0.012 | 1.333 | 238 |
| 146 | 88.09 | 85.05 | 3.04 | 0.533 | 0.956 | 0.956 | 0.007 | 1.021 | 249 |
| 147 | 36.45 | 32.28 | 4.17 | 0.504 | 1.271 | 1.273 | 0.011 | 1.284 | 287 |
| 148 | 62.14 | 59.35 | 2.79 | 0.533 | 0.878 | 0.877 | 0.006 | 0.937 | 102 |
| 149 | 89.45 | 86.02 | 3.43 | 0.533 | 1.080 | 1.080 | 0.009 | 1.155 | 175 |
| 150 | 89.12 | 87.70 | 1.42 | 0.533 | 0.446 | 0.445 | 0.001 | 0.475 | 132 |
| 151 | 88.02 | 87.51 | 0.5088 | 0.504 | 0.155 | 0.155 | 0.000 | 0.156 | 263 |
| 152 | 62.21 | 61.22 | 0.9950 | 0.533 | 0.313 | 0.312 | 0.001 | 0.333 | 174 |
| 153 | 52.47 | 53.54 | -1.07 | 0.533 | -0.336 | -0.335 | 0.001 | -0.358 | 25 |
| 154 | 86.02 | 86.08 | -0.0553 | 0.504 | -0.017 | -0.017 | 0.000 | -0.017 | 260 |
| 155 | 90.37 | 85.64 | 4.73 | 0.533 | 1.488 | 1.495 | 0.017 | 1.598 | 235 |
| 156 | 88.52 | 87.87 | 0.6464 | 0.533 | 0.203 | 0.203 | 0.000 | 0.217 | 229 |
| 157 | 92.14 | 90.02 | 2.12 | 0.533 | 0.667 | 0.666 | 0.003 | 0.711 | 35 |
| 158 | 54.27 | 54.88 | -0.6080 | 0.533 | -0.191 | -0.191 | 0.000 | -0.204 | 166 |
| 159 | 62.47 | 59.19 | 3.28 | 0.533 | 1.032 | 1.032 | 0.008 | 1.103 | 244 |
| 160 | 58.01 | 59.85 | -1.84 | 0.533 | -0.580 | -0.578 | 0.003 | -0.618 | 191 |
| 161 | 90.99 | 85.10 | 5.89 | 0.533 | 1.852 | 1.868 | 0.026 | 1.996 | 22 |
| 162 | 58.47 | 58.50 | -0.0257 | 0.533 | -0.008 | -0.008 | 0.000 | -0.009 | 86 |
| 163 | 54.23 | 57.31 | -3.08 | 0.533 | -0.969 | -0.969 | 0.007 | -1.036 | 92 |
| 164 | 86.07 | 83.65 | 2.42 | 0.533 | 0.761 | 0.760 | 0.004 | 0.812 | 140 |
| 165 | 52.47 | 56.74 | -4.27 | 0.533 | -1.343 | -1.347 | 0.013 | -1.440 | 11 |
| 166 | 91.88 | 88.51 | 3.37 | 0.533 | 1.060 | 1.060 | 0.008 | 1.133 | 88 |
| 167 | 86.45 | 86.09 | 0.3581 | 0.533 | 0.113 | 0.112 | 0.000 | 0.120 | 96 |
| 168 | 92.12 | 89.69 | 2.43 | 0.533 | 0.763 | 0.762 | 0.004 | 0.814 | 38 |
| 169 | 56.74 | 61.57 | -4.83 | 0.533 | -1.520 | -1.526 | 0.017 | -1.632 | 80 |
| 170 | 86.25 | 88.63 | -2.38 | 0.533 | -0.750 | -0.749 | 0.004 | -0.800 | 81 |
| 171 | 88.52 | 90.45 | -1.93 | 0.533 | -0.607 | -0.606 | 0.003 | -0.647 | 211 |
| 172 | 88.45 | 90.34 | -1.89 | 0.533 | -0.593 | -0.592 | 0.003 | -0.632 | 162 |
| 173 | 55.45 | 57.95 | -2.50 | 0.533 | -0.785 | -0.784 | 0.005 | -0.838 | 23 |
| 174 | 59.67 | 61.91 | -2.24 | 0.533 | -0.705 | -0.704 | 0.004 | -0.753 | 113 |
| 175 | 88.03 | 88.74 | -0.7101 | 0.533 | -0.223 | -0.223 | 0.000 | -0.238 | 152 |
| 176 | 88.01 | 90.66 | -2.65 | 0.533 | -0.833 | -0.832 | 0.005 | -0.890 | 63 |
| 177 | 88.12 | 86.02 | 2.10 | 0.533 | 0.661 | 0.660 | 0.003 | 0.705 | 29 |
| 178 | 58.99 | 61.46 | -2.47 | 0.533 | -0.776 | -0.775 | 0.004 | -0.828 | 40 |
| 179 | 88.25 | 89.94 | -1.69 | 0.533 | -0.533 | -0.531 | 0.002 | -0.568 | 78 |
| 180 | 91.45 | 85.13 | 6.32 | 0.533 | 1.988 | 2.008 | 0.030 | 2.147 | 170 |
| 181 | 86.22 | 82.79 | 3.43 | 0.533 | 1.079 | 1.079 | 0.009 | 1.154 | 182 |
| 182 | 58.27 | 58.16 | 0.1088 | 0.533 | 0.034 | 0.034 | 0.000 | 0.036 | 243 |
| 183 | 90.07 | 86.42 | 3.65 | 0.533 | 1.147 | 1.148 | 0.010 | 1.227 | 95 |
| 184 | 94.64 | 91.21 | 3.43 | 0.036 | 0.750 | 0.749 | 0.000 | 0.145 | 297 |
| 185 | 88.12 | 88.85 | -0.7288 | 0.533 | -0.229 | -0.228 | 0.000 | -0.244 | 213 |
| 186 | 88.97 | 83.51 | 5.46 | 0.533 | 1.718 | 1.729 | 0.022 | 1.849 | 214 |
| 187 | 89.97 | 87.55 | 2.42 | 0.504 | 0.738 | 0.737 | 0.004 | 0.743 | 265 |
| 188 | 52.47 | 53.76 | -1.29 | 0.533 | -0.404 | -0.403 | 0.001 | -0.431 | 129 |
| 189 | 92.78 | 93.08 | -0.2997 | 0.504 | -0.091 | -0.091 | 0.000 | -0.092 | 286 |
| 190 | 58.97 | 60.23 | -1.26 | 0.533 | -0.397 | -0.396 | 0.001 | -0.423 | 246 |
| 191 | 88.12 | 91.24 | -3.12 | 0.504 | -0.953 | -0.953 | 0.006 | -0.960 | 276 |
| 192 | 52.14 | 58.76 | -6.62 | 0.533 | -2.083 | -2.107 | 0.032 | -2.253⁽¹⁾ | 183 |
| 193 | 88.21 | 88.03 | 0.1813 | 0.533 | 0.057 | 0.057 | 0.000 | 0.061 | 116 |
| 194 | 87.68 | 86.40 | 1.28 | 0.533 | 0.402 | 0.401 | 0.001 | 0.428 | 169 |
| 195 | 56.98 | 56.15 | 0.8325 | 0.533 | 0.262 | 0.261 | 0.001 | 0.279 | 127 |
| 196 | 92.45 | 89.50 | 2.95 | 0.504 | 0.901 | 0.901 | 0.005 | 0.908 | 273 |
| 197 | 88.12 | 87.09 | 1.03 | 0.533 | 0.322 | 0.321 | 0.001 | 0.344 | 70 |
| 198 | 94.52 | 91.21 | 3.31 | 0.036 | 0.724 | 0.723 | 0.000 | 0.139 | 295 |
| 199 | 49.74 | 51.80 | -2.06 | 0.533 | -0.647 | -0.646 | 0.003 | -0.690 | 72 |
| 200 | 56.07 | 58.93 | -2.86 | 0.533 | -0.900 | -0.900 | 0.006 | -0.962 | 215 |
| 201 | 86.45 | 88.71 | -2.26 | 0.504 | -0.689 | -0.687 | 0.003 | -0.693 | 266 |
| 202 | 86.07 | 84.23 | 1.84 | 0.533 | 0.580 | 0.578 | 0.003 | 0.618 | 131 |
| 203 | 92.99 | 88.98 | 4.01 | 0.533 | 1.262 | 1.265 | 0.012 | 1.352 | 82 |
| 204 | 55.24 | 55.81 | -0.5670 | 0.533 | -0.178 | -0.178 | 0.000 | -0.190 | 59 |
| 205 | 62.34 | 59.91 | 2.43 | 0.533 | 0.763 | 0.762 | 0.004 | 0.814 | 210 |
| 206 | 60.12 | 56.06 | 4.06 | 0.533 | 1.278 | 1.281 | 0.012 | 1.369 | 73 |
| 207 | 52.14 | 53.60 | -1.46 | 0.533 | -0.458 | -0.457 | 0.002 | -0.488 | 17 |
| 208 | 58.45 | 64.08 | -5.63 | 0.533 | -1.770 | -1.783 | 0.023 | -1.906 | 226 |
| 209 | 62.45 | 61.49 | 0.9636 | 0.533 | 0.303 | 0.302 | 0.001 | 0.323 | 224 |
| 210 | 92.74 | 92.28 | 0.4612 | 0.504 | 0.141 | 0.140 | 0.000 | 0.141 | 285 |
| 211 | 88.45 | 89.68 | -1.23 | 0.533 | -0.386 | -0.385 | 0.001 | -0.411 | 112 |
| 212 | 90.64 | 86.92 | 3.72 | 0.533 | 1.170 | 1.171 | 0.010 | 1.252 | 145 |
| 213 | 88.24 | 86.26 | 1.98 | 0.533 | 0.621 | 0.620 | 0.003 | 0.663 | 1 |
| 214 | 50.41 | 55.92 | -5.51 | 0.533 | -1.734 | -1.746 | 0.022 | -1.867 | 6 |
| 215 | 48.75 | 54.84 | -6.09 | 0.533 | -1.915 | -1.933 | 0.027 | -2.067 | 94 |
| 216 | 88.34 | 91.35 | -3.01 | 0.533 | -0.945 | -0.945 | 0.007 | -1.010 | 139 |
| 217 | 88.78 | 85.22 | 3.56 | 0.533 | 1.118 | 1.119 | 0.009 | 1.196 | 2 |
| 218 | 42.12 | 50.82 | -8.70 | 0.533 | -2.736 | -2.799 | 0.056 | -2.992⁽¹⁾ | 137 |
| 219 | 58.74 | 59.96 | -1.22 | 0.533 | -0.383 | -0.381 | 0.001 | -0.408 | 240 |
| 220 | 90.99 | 85.05 | 5.94 | 0.504 | 1.812 | 1.826 | 0.022 | 1.841 | 278 |
| 221 | 70.24 | 63.59 | 6.65 | 0.533 | 2.090 | 2.115 | 0.033 | 2.261⁽¹⁾ | 204 |
| 222 | 49.87 | 54.05 | -4.18 | 0.533 | -1.315 | -1.318 | 0.013 | -1.409 | 85 |
| 223 | 52.41 | 53.03 | -0.6163 | 0.533 | -0.194 | -0.193 | 0.000 | -0.206 | 119 |
| 224 | 54.12 | 56.55 | -2.43 | 0.533 | -0.763 | -0.762 | 0.004 | -0.814 | 83 |
| 225 | 66.54 | 61.67 | 4.87 | 0.533 | 1.530 | 1.537 | 0.017 | 1.643 | 149 |
| 226 | 90.64 | 86.61 | 4.03 | 0.533 | 1.268 | 1.271 | 0.012 | 1.358 | 37 |
| 227 | 87.99 | 90.02 | -2.03 | 0.533 | -0.640 | -0.638 | 0.003 | -0.682 | 181 |
| 228 | 76.85 | 69.24 | 7.61 | 0.533 | 2.394 | 2.435 | 0.043 | 2.602⁽¹⁾ | 209 |
| 229 | 79.89 | 85.67 | -5.78 | 0.504 | -1.763 | -1.776 | 0.021 | -1.790 | 277 |
| 230 | 91.32 | 87.42 | 3.90 | 0.533 | 1.226 | 1.228 | 0.011 | 1.312 | 68 |
| 231 | 59.32 | 59.35 | -0.0338 | 0.533 | -0.011 | -0.011 | 0.000 | -0.011 | 156 |
| 232 | 52.37 | 53.63 | -1.26 | 0.533 | -0.397 | -0.395 | 0.001 | -0.423 | 18 |
| 233 | 57.23 | 59.45 | -2.22 | 0.533 | -0.697 | -0.696 | 0.004 | -0.744 | 120 |
| 234 | 88.24 | 89.14 | -0.9002 | 0.533 | -0.283 | -0.282 | 0.001 | -0.302 | 188 |
| 235 | 86.56 | 84.86 | 1.70 | 0.504 | 0.520 | 0.519 | 0.002 | 0.523 | 272 |
| 236 | 92.34 | 92.50 | -0.1649 | 0.533 | -0.052 | -0.052 | 0.000 | -0.055 | 105 |
| 237 | 58.74 | 58.69 | 0.0516 | 0.533 | 0.016 | 0.016 | 0.000 | 0.017 | 62 |
| 238 | 86.40 | 82.27 | 4.13 | 0.533 | 1.300 | 1.303 | 0.013 | 1.393 | 248 |
| 239 | 58.74 | 59.24 | -0.4975 | 0.533 | -0.156 | -0.156 | 0.000 | -0.167 | 186 |
| 240 | 67.10 | 60.56 | 6.54 | 0.533 | 2.055 | 2.079 | 0.032 | 2.222⁽¹⁾ | 31 |
| 241 | 56.24 | 55.06 | 1.18 | 0.533 | 0.371 | 0.370 | 0.001 | 0.395 | 24 |
| 242 | 62.23 | 56.88 | 5.35 | 0.533 | 1.682 | 1.652 | 0.021 | 1.801 | 163 |
| 243 | 60.47 | 62.58 | -2.11 | 0.533 | -0.663 | -0.662 | 0.003 | -0.717 | 51 |
| 244 | 58.24 | 57.35 | 1.05 | 0.533 | 0.330 | 0.329 | 0.001 | 0.451 | 100 |
| 245 | 88.78 | 92.50 | -3.72 | 0.533 | -1.170 | -1.172 | 0.010 | -1.252 | 118 |
| 246 | 84.52 | 84.83 | -0.5249 | 0.533 | -0.165 | -0.164 | 0.000 | -0.176 | 10 |
| 247 | 76.85 | 68.99 | 7.86 | 0.533 | 2.471 | 2.516 | 0.046 | 2.690⁽¹⁾ | 122 |
| 248 | 88.52 | 87.21 | 1.31 | 0.533 | 0.412 | 0.431 | 0.001 | 0.469 | 247 |
| 249 | 91.32 | 89.94 | 1.38 | 0.533 | 0.433 | 0.443 | 0.001 | 0.462 | 219 |
| 250 | 68.74 | 67.70 | 1.04 | 0.533 | 0.327 | 0.326 | 0.001 | 0.349 | 109 |
| 251 | 84.41 | 85.85 | -1.54 | 0.504 | -0.471 | -0.469 | 0.001 | -0.473 | 271 |
| 252 | 68.74 | 65.73 | 3.01 | 0.533 | 0.946 | 0.945 | 0.007 | 1.010 | 144 |
| 253 | 92.45 | 91.71 | 1.24 | 0.036 | 0.271 | 0.270 | 0.000 | 0.052 | 291 |
| 254 | 67.45 | 59.89 | 7.56 | 0.533 | 2.377 | 2.417 | 0.042 | 2.583⁽¹⁾ | 135 |
| 255 | 70.32 | 64.53 | 5.59 | 0.533 | 1.758 | 1.771 | 0.023 | 1.893 | 71 |
| 256 | 86.45 | 90.45 | -4.00 | 0.504 | -1.221 | -1.223 | 0.010 | -1.233 | 288 |
| 257 | 88.52 | 88.43 | 0.0867 | 0.504 | 0.027 | 0.027 | 0.000 | 0.027 | 279 |
| 258 | 60.47 | 61.42 | -0.9468 | 0.533 | -0.298 | -0.297 | 0.001 | -0.318 | 192 |
| 259 | 88.78 | 91.69 | -2.91 | 0.533 | -0.914 | -0.914 | 0.006 | -0.976 | 121 |
| 260 | 59.99 | 57.68 | 2.31 | 0.533 | 0.746 | 0.724 | 0.004 | 0.774 | 216 |
| 261 | 88.78 | 79.04 | -0.2637 | 0.533 | -0.078 | -0.083 | 0.000 | -0.084 | 21 |
| 262 | 88.12 | 88.09 | -0.0189 | 0.533 | -0.016 | -0.006 | 0.000 | -0.002 | 193 |
| 263 | 54.00 | 62.13 | -6.23 | 0.533 | -1.937 | -1.945 | 0.028 | -2.089 | 219 |
| 264 | 56.37 | 57.75 | -1.38 | 0.533 | -0.434 | -0.433 | 0.001 | -0.472 | 34 |
| 265 | 60.70 | 57.42 | 3.28 | 0.533 | 1.047 | 1.047 | 0.008 | 1.117 | 54 |
| 266 | 58.74 | 61.91 | -2.27 | 0.533 | -0.682 | -0.681 | 0.003 | -0.728 | 253 |
| 267 | 86.37 | 89.10 | -2.73 | 0.504 | -0.841 | -0.852 | 0.005 | -0.823 | 258 |
| 268 | 88.24 | 86.56 | -0.3431 | 0.533 | -0.112 | -0.101 | 0.000 | -0.128 | 210 |
| 269 | 87.25 | 87.15 | 0.2031 | 0.533 | 0.064 | 0.068 | 0.000 | 0.068 | 123 |
| 270 | 82.45 | 83.48 | -1.03 | 0.504 | -0.315 | -0.314 | 0.001 | -0.312 | 257 |
| 271 | 68.45 | 64.17 | 4.10 | 0.533 | 1.288 | 1.391 | 0.012 | 1.380 | 108 |
| 272 | 87.45 | 93.49 | -6.04 | 0.533 | -1.900 | -1.918 | 0.027 | -2.050 | 67 |
| 273 | 56.34 | 55.30 | 1.04 | 0.533 | 0.326 | 0.325 | 0.001 | 0.348 | 158 |
| 274 | 88.07 | 88.42 | -0.3473 | 0.504 | -0.106 | -0.106 | 0.000 | -0.106 | 264 |
| 275 | 58.75 | 63.70 | -4.95 | 0.533 | -1.558 | -1.565 | 0.018 | -1.673 | 245 |
| 276 | 64.24 | 56.74 | -3.50 | 0.533 | -1.102 | -1.102 | 0.009 | -1.178 | 231 |
| 277 | 88.66 | 86.65 | 1.01 | 0.533 | 0.317 | 0.316 | 0.001 | 0.337 | 89 |
| 278 | 84.22 | 85.14 | 3.00 | 0.533 | 0.943 | 0.942 | 0.007 | 1.007 | 9 |
| 279 | 90.16 | 92.20 | -2.98 | 0.533 | -0.938 | -0.938 | 0.007 | -1.002 | 62 |
| 280 | 43.47 | 58.66 | 2.61 | 0.533 | 0.822 | 0.821 | 0.005 | 0.878 | 169 |
| 281 | 83.28 | 84.91 | 1.40 | 0.533 | 0.441 | 0.440 | 0.001 | 0.470 | 84 |
| 282 | 84.44 | 90.52 | -1.84 | 0.533 | -0.578 | -0.576 | 0.002 | -0.616 | 77 |
| 283 | 61.23 | 64.76 | 3.47 | 0.533 | 1.092 | 1.092 | 0.009 | 1.168 | 204 |
| 284 | 84.58 | 85.26 | -0.8240 | 0.533 | -0.259 | -0.258 | 0.001 | -0.276 | 206 |
| 285 | 86.59 | 85.57 | -0.3333 | 0.533 | -0.105 | -0.104 | 0.000 | -0.112 | 49 |
| 286 | 63.87 | 62.06 | -0.6414 | 0.533 | -0.202 | -0.201 | 0.000 | -0.215 | 146 |
| 287 | 63.52 | 63.49 | -0.5547 | 0.533 | -0.174 | -0.174 | 0.000 | -0.186 | 227 |
| 288 | 84.78 | 82.70 | 6.08 | 0.533 | 1.910 | 1.928 | 0.027 | 2.061 | 74 |
| 289 | 78.11 | 87.45 | -1.34 | 0.533 | -0.423 | -0.422 | 0.001 | -0.451 | 223 |
| 290 | 86.19 | 92.40 | -4.08 | 0.533 | -1.282 | -1.285 | 0.012 | -1.374 | 104 |
| 291 | 84.16 | 88.18 | -4.92 | 0.533 | -1.548 | -1.556 | 0.018 | -1.663 | 241 |
| 292 | 68.64 | 56.80 | -0.1395 | 0.533 | -0.040 | -0.050 | 0.000 | -0.052 | 174 |
| 293 | 61.55 | 52.32 | 0.0366 | 0.533 | 0.014 | 0.004 | 0.000 | 0.013 | 195 |
| 294 | 78.54 | 81.57 | 2.34 | 0.523 | 0.786 | 0.735 | 0.004 | 0.817 | 164 |
| 295 | 91.71 | 89.54 | 1.66 | 0.533 | 0.520 | 0.519 | 0.002 | 0.555 | 187 |
| 296 | 62.56 | 61.42 | 0.3541 | 0.523 | 0.123 | 0.114 | 0.000 | 0.140 | 178 |
| 297 | 86.41 | 84.74 | 0.5312 | 0.523 | 0.158 | 0.147 | 0.000 | 0.149 | 113 |
| 298 | 62.42 | 62.32 | 1.27 | 0.533 | 0.358 | 0.364 | 0.001 | 0.373 | 91 |

⁽¹⁾ Exceeds limits.

⁽²⁾ Observation with |External Stud. Residuals| > 3.86

**S Table 6:**

Coefficients in Terms of Coded Factors

| **Factor** | **Coefficient Estimate** | **df** | **Standard Error** | **95% CI Low** | **95% CI High** | **VIF** |
| --- | --- | --- | --- | --- | --- | --- |
| Intercept | 92.21 | 1 | 0.8819 | 89.47 | 92.95 |  |
| A-Glucose concentration | -0.0166 | 1 | 0.2865 | -0.6819 | 0.4506 | 1.0000 |
| B-Urea concentration | -0.0670 | 1 | 0.2865 | -0.6282 | 0.5043 | 1.0000 |
| C-K_2_HPO_4_ concentration | -0.2140 | 1 | 0.2865 | -0.8683 | 0.2642 | 1.0000 |
| D-KH_2_PO_4_ concentration | 0.2305 | 1 | 0.2865 | -0.3397 | 0.7928 | 1.0000 |
| E-MgSO_4_.7H_2_O concentration | 0.2862 | 1 | 0.2865 | -0.2770 | 0.8555 | 1.0000 |
| F-KCl concentration | 0.4681 | 1 | 0.2865 | -0.1051 | 1.03 | 1.0000 |
| G-CoCl_2_.6H_2_O concentration | 0.4171 | 1 | 0.2865 | -0.1490 | 0.9835 | 1.0000 |
| H-NH_4_VO_2_ concentration | -0.2463 | 1 | 0.2865 | -0.8155 | 0.3170 | 1.0000 |
| J-Na_2_MoO_4_.2H_2_O concentration | -0.1254 | 1 | 0.2865 | -0.6869 | 0.4456 | 1.0000 |
| K-CaCO_3_ concentration | 0.2457 | 1 | 0.2865 | -0.1188 | 1.01 | 1.0000 |
| L-FeSO_4_.7H_2_O concentration | 0.0551 | 1 | 0.2865 | -0.7208 | 0.4117 | 1.0000 |
| M-ZnSO_4_.7H_2_O concentration | 0.0341 | 1 | 0.2865 | -0.5326 | 0.5999 | 1.0000 |
| N-MnSO_4_.4H_2_O concentration | -0.2467 | 1 | 0.2865 | -0.8139 | 0.3186 | 1.0000 |
| O-NiSO_4_.7H_2_O concentration | 0.2641 | 1 | 0.2865 | -0.2059 | 0.9266 | 1.0000 |
| P-Na_2_B_4_O_7_.10H_2_O concentration | 0.2031 | 1 | 0.2865 | -0.3660 | 0.7665 | 1.0000 |
| Q-Dry Cell Weight | 6.44 | 1 | 0.2865 | 8.98 | 11.11 | 1.0000 |
| AB | 0.0174 | 1 | 0.2909 | -0.5606 | 0.5895 | 1.0000 |
| AC | -0.3247 | 1 | 0.2909 | -0.9016 | 0.2485 | 1.0000 |
| AD | 0.2122 | 1 | 0.2909 | -0.3738 | 0.7763 | 1.0000 |
| AE | -0.0012 | 1 | 0.2909 | -0.5753 | 0.5748 | 1.0000 |
| AF | 0.1475 | 1 | 0.2909 | -0.4315 | 0.7186 | 1.0000 |
| AG | 0.2919 | 1 | 0.2909 | -0.2851 | 0.8650 | 1.0000 |
| AH | 0.0210 | 1 | 0.2909 | -0.5630 | 0.5871 | 1.0000 |
| AJ | 0.3037 | 1 | 0.2909 | -0.2723 | 0.8778 | 1.0000 |
| AK | -0.5146 | 1 | 0.2909 | -1.15 | -0.0006 | 1.0000 |
| AL | 0.1582 | 1 | 0.2909 | -0.4165 | 0.7336 | 1.0000 |
| AM | 0.0967 | 1 | 0.2909 | -0.4773 | 0.6728 | 1.0000 |
| AN | -0.0805 | 1 | 0.2909 | -0.6555 | 0.4946 | 1.0000 |
| AO | 0.3252 | 1 | 0.2909 | -0.1146 | 1.04 | 1.0000 |
| AP | 0.1463 | 1 | 0.2909 | -0.4377 | 0.7124 | 1.0000 |
| AQ | -0.1486 | 1 | 0.2909 | -0.7136 | 0.4364 | 1.0000 |
| BC | 0.0954 | 1 | 0.2909 | -0.4788 | 0.6713 | 1.0000 |
| BD | -0.0921 | 1 | 0.2909 | -0.6669 | 0.4832 | 1.0000 |
| BE | -0.4272 | 1 | 0.2909 | -0.9982 | 0.1518 | 1.0000 |
| BF | -0.6308 | 1 | 0.2909 | -1.20 | -0.0488 | 1.0000 |
| BG | -0.6584 | 1 | 0.2909 | -1.24 | -0.0854 | 1.0000 |
| BH | 0.3967 | 1 | 0.2909 | -0.1833 | 0.9668 | 1.0000 |
| BJ | -0.3579 | 1 | 0.2909 | -0.8850 | 0.2651 | 1.0000 |
| BK | -0.4425 | 1 | 0.2909 | -1.01 | 0.1405 | 1.0000 |
| BL | 0.0957 | 1 | 0.2909 | -0.4806 | 0.6695 | 1.0000 |
| BM | 0.5762 | 1 | 0.2909 | -0.0034 | 1.15 | 1.0000 |
| BN | 0.0387 | 1 | 0.2909 | -0.5421 | 0.6080 | 1.0000 |
| BO | 0.0862 | 1 | 0.2909 | -0.4895 | 0.6606 | 1.0000 |
| BP | 0.0217 | 1 | 0.2909 | -0.5624 | 0.5877 | 1.0000 |
| BQ | 0.0134 | 1 | 0.2909 | -0.5639 | 0.5861 | 1.0000 |
| CD | 0.2387 | 1 | 0.2909 | -0.3314 | 0.8187 | 1.0000 |
| CE | -0.3503 | 1 | 0.2909 | -0.8803 | 0.2697 | 1.0000 |
| CF | -0.0255 | 1 | 0.2909 | -0.5905 | 0.5596 | 1.0000 |
| CG | -0.2873 | 1 | 0.2909 | -0.8613 | 0.2888 | 1.0000 |
| CH | -0.1971 | 1 | 0.2909 | -0.7701 | 0.3800 | 1.0000 |
| CJ | -0.3352 | 1 | 0.2909 | -0.9125 | 0.2375 | 1.0000 |
| CK | 0.3631 | 1 | 0.2909 | -0.2137 | 0.9364 | 1.0000 |
| CL | 0.2028 | 1 | 0.2909 | -0.3746 | 0.7755 | 1.0000 |
| CM | 0.3124 | 1 | 0.2909 | -0.2686 | 0.8814 | 1.0000 |
| CN | 0.3513 | 1 | 0.2909 | -0.2257 | 0.9243 | 1.0000 |
| CO | 0.6517 | 1 | 0.2909 | 0.0747 | 1.22 | 1.0000 |
| CP | -0.1213 | 1 | 0.2909 | -0.7064 | 0.4437 | 1.0000 |
| CQ | 0.0316 | 1 | 0.2909 | -0.5604 | 0.5896 | 1.0000 |
| DE | 0.1747 | 1 | 0.2909 | -0.4024 | 0.7477 | 1.0000 |
| DF | 0.2174 | 1 | 0.2909 | -0.3207 | 0.8294 | 1.0000 |
| DG | -0.2210 | 1 | 0.2909 | -0.7800 | 0.3700 | 1.0000 |
| DH | 0.1741 | 1 | 0.2909 | -0.4409 | 0.7092 | 1.0000 |
| DJ | 0.1545 | 1 | 0.2909 | -0.4446 | 0.7055 | 1.0000 |
| DK | -0.0429 | 1 | 0.2909 | -0.5800 | 0.5701 | 1.0000 |
| DL | -0.8475 | 1 | 0.2909 | -1.38 | -0.2304 | 1.0000 |
| DM | -0.0936 | 1 | 0.2909 | -0.6716 | 0.4785 | 1.0000 |
| DN | 0.5272 | 1 | 0.2909 | -0.0668 | 1.08 | 1.0000 |
| DO | 0.1999 | 1 | 0.2909 | -0.3761 | 0.7739 | 1.0000 |
| DP | -0.3281 | 1 | 0.2909 | -0.8921 | 0.2579 | 1.0000 |
| DQ | 0.0724 | 1 | 0.2909 | -0.5037 | 0.6464 | 1.0000 |
| EF | -0.0688 | 1 | 0.2909 | -0.6439 | 0.5062 | 1.0000 |
| EG | -0.3647 | 1 | 0.2909 | -0.9078 | 0.2423 | 1.0000 |
| EH | 0.7954 | 1 | 0.2909 | 0.2189 | 1.37 | 1.0000 |
| EJ | -0.0747 | 1 | 0.2909 | -0.6257 | 0.5243 | 1.0000 |
| EK | -0.2284 | 1 | 0.2909 | -0.7764 | 0.3736 | 1.0000 |
| EL | -0.1657 | 1 | 0.2909 | -0.7362 | 0.4139 | 1.0000 |
| EM | -0.2984 | 1 | 0.2909 | -0.8734 | 0.2767 | 1.0000 |
| EN | 0.1733 | 1 | 0.2909 | -0.5018 | 0.6483 | 1.0000 |
| EO | -0.4574 | 1 | 0.2909 | -1.01 | 0.1376 | 1.0000 |
| EP | -0.1727 | 1 | 0.2909 | -0.7543 | 0.3958 | 1.0000 |
| EQ | -0.1322 | 1 | 0.2909 | -0.6872 | 0.4628 | 1.0000 |
| FG | -0.2579 | 1 | 0.2909 | -0.8229 | 0.3271 | 1.0000 |
| FH | -0.7673 | 1 | 0.2909 | -1.31 | -0.1603 | 1.0000 |
| FJ | 0.2711 | 1 | 0.2909 | -0.3559 | 0.7942 | 1.0000 |
| FK | -0.2251 | 1 | 0.2909 | -0.7891 | 0.3610 | 1.0000 |
| FL | -0.4061 | 1 | 0.2909 | -0.9789 | 0.1712 | 1.0000 |
| FM | -0.1312 | 1 | 0.2909 | -0.6982 | 0.4518 | 1.0000 |
| FN | 0.1126 | 1 | 0.2909 | -0.4735 | 0.6766 | 1.0000 |
| FO | -0.5249 | 1 | 0.2909 | -1.10 | 0.0492 | 1.0000 |
| FP | -0.3212 | 1 | 0.2909 | -0.8843 | 0.2658 | 1.0000 |
| FQ | -0.2176 | 1 | 0.2909 | -0.7866 | 0.3635 | 1.0000 |
| GH | 0.0570 | 1 | 0.2909 | -0.5175 | 0.6325 | 1.0000 |
| GJ | 0.5424 | 1 | 0.2909 | -0.0332 | 1.12 | 1.0000 |
| GK | -0.1676 | 1 | 0.2909 | -0.6266 | 0.5235 | 1.0000 |
| GL | 0.2242 | 1 | 0.2909 | -0.5689 | 0.5812 | 1.0000 |
| GM | 0.4548 | 1 | 0.2909 | -0.1412 | 1.01 | 1.0000 |
| GN | 0.0687 | 1 | 0.2909 | -0.5064 | 0.6436 | 1.0000 |
| GO | 0.1227 | 1 | 0.2909 | -0.4593 | 0.6907 | 1.0000 |
| GP | -0.0197 | 1 | 0.2909 | -0.6177 | 0.5324 | 1.0000 |
| GQ | -0.1461 | 1 | 0.2909 | -0.7282 | 0.4219 | 1.0000 |
| HJ | -0.0780 | 1 | 0.2909 | -0.6530 | 0.4971 | 1.0000 |
| HK | 0.4370 | 1 | 0.2909 | -0.1640 | 0.9861 | 1.0000 |
| HL | -0.0433 | 1 | 0.2909 | -0.6283 | 0.5218 | 1.0000 |
| HM | -0.4547 | 1 | 0.2909 | -0.9847 | 0.1653 | 1.0000 |
| HN | -0.0215 | 1 | 0.2909 | -0.6256 | 0.5245 | 1.0000 |
| HO | 0.0438 | 1 | 0.2909 | -0.5313 | 0.6188 | 1.0000 |
| HP | 0.0430 | 1 | 0.2909 | -0.5320 | 0.6181 | 1.0000 |
| HQ | -0.2756 | 1 | 0.2909 | -0.8492 | 0.3009 | 1.0000 |
| JK | -0.3239 | 1 | 0.2909 | -0.8989 | 0.2511 | 1.0000 |
| JL | 0.3332 | 1 | 0.2909 | -0.2436 | 0.9065 | 1.0000 |
| JM | -0.1174 | 1 | 0.2909 | -0.6907 | 0.4593 | 1.0000 |
| JN | -0.3139 | 1 | 0.2909 | -0.8889 | 0.2611 | 1.0000 |
| JO | 0.2352 | 1 | 0.2909 | -0.3423 | 0.8078 | 1.0000 |
| JP | -0.2817 | 1 | 0.2909 | -0.8564 | 0.2936 | 1.0000 |
| JQ | 0.2183 | 1 | 0.2909 | -0.3597 | 0.7903 | 1.0000 |
| KL | -0.0419 | 1 | 0.2909 | -0.6169 | 0.5332 | 1.0000 |
| KM | -0.1103 | 1 | 0.2909 | -0.6853 | 0.4647 | 1.0000 |
| KN | -0.3523 | 1 | 0.2909 | -0.9273 | 0.2228 | 1.0000 |
| KO | -0.1466 | 1 | 0.2909 | -0.7216 | 0.4285 | 1.0000 |
| KP | -0.0855 | 1 | 0.2909 | -0.6606 | 0.4895 | 1.0000 |
| KQ | -0.4974 | 1 | 0.2909 | -1.07 | 0.0776 | 1.0000 |
| LM | 0.3666 | 1 | 0.2909 | -0.2084 | 0.9417 | 1.0000 |
| LN | -0.6487 | 1 | 0.2909 | -1.22 | -0.0737 | 1.0000 |
| LO | -0.1043 | 1 | 0.2909 | -0.6793 | 0.4707 | 1.0000 |
| LP | 0.2403 | 1 | 0.2909 | -0.3347 | 0.8153 | 1.0000 |
| LQ | 0.8120 | 1 | 0.2909 | 0.2370 | 1.39 | 1.0000 |
| MN | -0.0681 | 1 | 0.2909 | -0.6432 | 0.5069 | 1.0000 |
| MO | -0.1213 | 1 | 0.2909 | -0.6964 | 0.4537 | 1.0000 |
| MP | -0.2145 | 1 | 0.2909 | -0.7896 | 0.3605 | 1.0000 |
| MQ | -0.0406 | 1 | 0.2909 | -0.6157 | 0.5344 | 1.0000 |
| NO | 0.1311 | 1 | 0.2909 | -0.4439 | 0.7061 | 1.0000 |
| NP | -0.2741 | 1 | 0.2909 | -0.8421 | 0.3079 | 1.0000 |
| NQ | 0.3216 | 1 | 0.2909 | -0.2584 | 0.8917 | 1.0000 |
| OP | -0.4523 | 1 | 0.2909 | -0.9874 | 0.1627 | 1.0000 |
| OQ | 0.2374 | 1 | 0.2909 | -0.3724 | 0.7777 | 1.0000 |
| PQ | 0.2746 | 1 | 0.2909 | -0.3004 | 0.8496 | 1.0000 |
| A² | -0.4787 | 1 | 0.7990 | -2.05 | 1.11 | 1.59 |
| B² | -1.25 | 1 | 0.7990 | -2.83 | 0.3262 | 1.59 |
| C² | 0.0356 | 1 | 0.7990 | -1.54 | 1.61 | 1.59 |
| D² | -0.8196 | 1 | 0.7990 | -2.39 | 0.7675 | 1.59 |
| E² | -0.7174 | 1 | 0.7990 | -2.35 | 0.8087 | 1.59 |
| F² | -1.70 | 1 | 0.7990 | -3.28 | -0.1225 | 1.59 |
| G² | -0.3159 | 1 | 0.7990 | -1.93 | 1.23 | 1.59 |
| H² | -1.37 | 1 | 0.7990 | -3.04 | 0.1150 | 1.59 |
| J² | -0.4721 | 1 | 0.7990 | -2.07 | 1.09 | 1.59 |
| K² | -0.2244 | 1 | 0.7990 | -1.79 | 1.36 | 1.59 |
| L² | -1.37 | 1 | 0.7990 | -3.04 | 0.1162 | 1.59 |
| M² | -0.6819 | 1 | 0.7990 | -2.26 | 0.9012 | 1.59 |
| N² | -0.7174 | 1 | 0.7990 | -2.30 | 0.8537 | 1.59 |
| O² | -0.2186 | 1 | 0.7990 | -1.81 | 1.35 | 1.59 |
| P² | 0.3627 | 1 | 0.7990 | -1.21 | 1.95 | 1.59 |
| Q² | -7.26 | 1 | 0.7990 | -9.04 | -5.88 | 1.59 |

**S Table 7:**

The Solutions (100 Solutions)

| **Number** | **Glucose concentration** | **Urea concentration** | **K_2_HPO_4_ concentration** | **KH_2_PO_4_ concentration** | **MgSO_4_.7H_2_O concentration** | **KCl concentration** | **CoCl_2_.6H_2_O concentration** | **NH_4_VO_2_ concentration** | **Na_2_MoO_4_.2H_2_O concentration** | **CaCO_3_ concentration** | **FeSO_4_.7H_2_O concentration** | **ZnSO_4_.7H_2_O concentration** | **MnSO_4_.4H_2_O concentration** | **NiSO_4_.7H_2_O concentration** | **Na_2_B_4_O_7_.10H_2_O concentration** | **Dry Cell Weight** | **Cd(II) biosorption efficiency** | **Desirability** |  |
| --- | --- | --- | --- | --- | --- | --- | --- | --- | --- | --- | --- | --- | --- | --- | --- | --- | --- | --- | --- |
| **1** | **10.748** | **1.071** | **0.127** | **0.122** | **0.057** | **0.864** | **0.027** | **0.757** | **0.047** | **0.054** | **0.052** | **0.057** | **0.047** | **0.037** | **0.571** | **1.532** | **95.018** | **1.000** | **Selected** |
| 2 | 8.211 | 1.047 | 0.164 | 0.114 | 0.038 | 0.484 | 0.070 | 0.539 | 0.070 | 0.061 | 0.043 | 0.048 | 0.058 | 0.078 | 0.510 | 1.482 | 94.208 | 1.000 |  |
| 3 | 9.404 | 0.904 | 0.087 | 0.102 | 0.056 | 0.685 | 0.051 | 0.712 | 0.095 | 0.051 | 0.048 | 0.053 | 0.059 | 0.060 | 0.748 | 1.309 | 96.369 | 1.000 |  |
| 4 | 10.111 | 0.814 | 0.131 | 0.081 | 0.067 | 0.716 | 0.034 | 0.564 | 0.082 | 0.064 | 0.046 | 0.037 | 0.052 | 0.065 | 0.812 | 1.089 | 95.121 | 1.000 |  |
| 5 | 8.219 | 0.848 | 0.063 | 0.074 | 0.050 | 0.406 | 0.064 | 0.534 | 0.118 | 0.063 | 0.062 | 0.063 | 0.041 | 0.075 | 0.587 | 1.427 | 95.189 | 1.000 |  |
| 6 | 10.614 | 0.826 | 0.146 | 0.130 | 0.076 | 0.900 | 0.030 | 0.570 | 0.062 | 0.070 | 0.052 | 0.032 | 0.059 | 0.060 | 0.518 | 1.992 | 94.940 | 1.000 |  |
| 7 | 8.122 | 0.817 | 0.133 | 0.131 | 0.074 | 0.862 | 0.066 | 0.907 | 0.126 | 0.068 | 0.055 | 0.072 | 0.052 | 0.065 | 0.514 | 1.392 | 95.114 | 1.000 |  |
| 8 | 10.448 | 1.121 | 0.142 | 0.130 | 0.025 | 0.825 | 0.031 | 0.563 | 0.079 | 0.038 | 0.039 | 0.042 | 0.057 | 0.061 | 0.518 | 1.983 | 94.868 | 1.000 |  |
| 9 | 9.401 | 0.828 | 0.123 | 0.082 | 0.076 | 0.787 | 0.068 | 0.849 | 0.058 | 0.064 | 0.037 | 0.037 | 0.053 | 0.078 | 0.537 | 1.797 | 95.063 | 1.000 |  |
| 10 | 11.224 | 1.013 | 0.072 | 0.133 | 0.076 | 0.824 | 0.036 | 0.947 | 0.067 | 0.065 | 0.037 | 0.046 | 0.045 | 0.074 | 0.522 | 1.498 | 95.116 | 1.000 |  |
| 11 | 10.106 | 1.200 | 0.137 | 0.134 | 0.033 | 0.687 | 0.036 | 0.522 | 0.062 | 0.070 | 0.044 | 0.074 | 0.041 | 0.061 | 0.929 | 1.824 | 94.988 | 1.000 |  |
| 12 | 11.117 | 0.942 | 0.134 | 0.124 | 0.045 | 0.911 | 0.044 | 0.734 | 0.084 | 0.048 | 0.055 | 0.066 | 0.042 | 0.068 | 0.546 | 1.627 | 95.569 | 1.000 |  |
| 13 | 8.752 | 1.041 | 0.121 | 0.166 | 0.057 | 0.671 | 0.051 | 0.682 | 0.086 | 0.027 | 0.045 | 0.048 | 0.044 | 0.076 | 0.848 | 1.007 | 96.341 | 1.000 |  |
| 14 | 8.6574 | 0.834 | 0.137 | 0.066 | 0.033 | 0.903 | 0.058 | 0.534 | 0.132 | 0.069 | 0.066 | 0.076 | 0.031 | 0.079 | 0.525 | 1.515 | 95.227 | 1.000 |  |
| 15 | 9.455 | 0.860 | 0.075 | 0.075 | 0.037 | 0.585 | 0.056 | 0.680 | 0.135 | 0.044 | 0.051 | 0.049 | 0.051 | 0.078 | 0.727 | 1.871 | 94.789 | 1.000 |  |
| 16 | 8.291 | 0.982 | 0.115 | 0.073 | 0.071 | 0.940 | 0.036 | 0.586 | 0.140 | 0.056 | 0.039 | 0.026 | 0.056 | 0.073 | 0.582 | 1.634 | 94.689 | 1.000 |  |
| 17 | 10.795 | 0.889 | 0.086 | 0.137 | 0.041 | 0.819 | 0.035 | 0.564 | 0.072 | 0.035 | 0.033 | 0.032 | 0.047 | 0.078 | 0.564 | 2.029 | 94.520 | 1.000 |  |
| 18 | 10.486 | 1.131 | 0.062 | 0.090 | 0.072 | 0.829 | 0.042 | 0.561 | 0.127 | 0.066 | 0.065 | 0.034 | 0.041 | 0.072 | 0.949 | 1.786 | 94.264 | 1.000 |  |
| 19 | 11.425 | 1.143 | 0.063 | 0.134 | 0.034 | 0.937 | 0.068 | 0.548 | 0.131 | 0.032 | 0.035 | 0.077 | 0.042 | 0.061 | 0.975 | 2.124 | 95.235 | 1.000 |  |
| 20 | 9.445 | 0.920 | 0.081 | 0.071 | 0.065 | 0.863 | 0.047 | 0.764 | 0.097 | 0.042 | 0.042 | 0.040 | 0.046 | 0.078 | 0.833 | 1.696 | 94.719 | 1.000 |  |
| 21 | 10.064 | 0.902 | 0.120 | 0.086 | 0.056 | 0.799 | 0.041 | 0.811 | 0.119 | 0.065 | 0.056 | 0.064 | 0.053 | 0.074 | 0.732 | 1.933 | 96.279 | 1.000 |  |
| 22 | 8.555 | 0.931 | 0.132 | 0.073 | 0.076 | 0.505 | 0.058 | 0.625 | 0.080 | 0.036 | 0.067 | 0.074 | 0.058 | 0.076 | 0.912 | 1.751 | 94.327 | 1.000 |  |
| 23 | 10.753 | 0.984 | 0.123 | 0.114 | 0.064 | 0.501 | 0.052 | 0.941 | 0.080 | 0.061 | 0.064 | 0.062 | 0.046 | 0.077 | 0.615 | 1.485 | 94.288 | 1.000 |  |
| 24 | 9.014 | 1.082 | 0.072 | 0.139 | 0.052 | 0.542 | 0.067 | 0.780 | 0.136 | 0.069 | 0.066 | 0.079 | 0.046 | 0.064 | 0.633 | 1.998 | 94.272 | 1.000 |  |
| 25 | 9.380 | 0.931 | 0.098 | 0.071 | 0.063 | 0.942 | 0.057 | 0.779 | 0.124 | 0.052 | 0.054 | 0.051 | 0.052 | 0.064 | 0.799 | 1.678 | 95.621 | 1.000 |  |
| 26 | 11.807 | 1.172 | 0.060 | 0.071 | 0.033 | 0.473 | 0.030 | 0.914 | 0.062 | 0.069 | 0.051 | 0.070 | 0.043 | 0.077 | 0.984 | 1.830 | 94.702 | 1.000 |  |
| 27 | 11.125 | 0.871 | 0.126 | 0.130 | 0.041 | 0.902 | 0.032 | 0.849 | 0.145 | 0.067 | 0.033 | 0.037 | 0.053 | 0.079 | 0.828 | 2.051 | 94.378 | 1.000 |  |
| 28 | 11.987 | 0.975 | 0.122 | 0.085 | 0.070 | 0.766 | 0.036 | 0.653 | 0.087 | 0.034 | 0.053 | 0.072 | 0.046 | 0.068 | 0.612 | 1.761 | 94.624 | 1.000 |  |
| 29 | 11.813 | 1.171 | 0.067 | 0.126 | 0.033 | 0.451 | 0.069 | 0.949 | 0.136 | 0.067 | 0.067 | 0.077 | 0.041 | 0.079 | 0.983 | 1.973 | 95.316 | 1.000 |  |
| 30 | 8.372 | 0.808 | 0.167 | 0.127 | 0.040 | 0.662 | 0.042 | 0.680 | 0.124 | 0.056 | 0.041 | 0.056 | 0.046 | 0.063 | 0.987 | 1.600 | 95.152 | 1.000 |  |
| 31 | 10.740 | 1.117 | 0.073 | 0.090 | 0.065 | 0.720 | 0.065 | 0.646 | 0.124 | 0.061 | 0.063 | 0.052 | 0.057 | 0.068 | 0.952 | 1.802 | 95.463 | 1.000 |  |
| 32 | 10.638 | 0.917 | 0.074 | 0.079 | 0.064 | 0.756 | 0.033 | 0.827 | 0.086 | 0.059 | 0.065 | 0.033 | 0.055 | 0.068 | 0.523 | 2.056 | 94.465 | 1.000 |  |
| 33 | 11.152 | 0.905 | 0.075 | 0.113 | 0.053 | 0.714 | 0.042 | 0.894 | 0.097 | 0.058 | 0.048 | 0.045 | 0.049 | 0.069 | 0.776 | 1.423 | 95.680 | 1.000 |  |
| 34 | 10.581 | 0.861 | 0.123 | 0.126 | 0.037 | 0.777 | 0.032 | 0.575 | 0.142 | 0.041 | 0.063 | 0.045 | 0.047 | 0.063 | 0.567 | 1.896 | 94.794 | 1.000 |  |
| 35 | 10.579 | 0.839 | 0.143 | 0.080 | 0.049 | 0.871 | 0.044 | 0.673 | 0.163 | 0.061 | 0.061 | 0.049 | 0.046 | 0.076 | 0.642 | 1.623 | 95.389 | 1.000 |  |
| 36 | 8.352 | 0.892 | 0.120 | 0.065 | 0.049 | 0.965 | 0.070 | 0.753 | 0.141 | 0.030 | 0.065 | 0.073 | 0.041 | 0.074 | 0.587 | 1.494 | 94.564 | 1.000 |  |
| 37 | 11.359 | 1.061 | 0.071 | 0.130 | 0.071 | 0.948 | 0.043 | 0.707 | 0.076 | 0.064 | 0.035 | 0.070 | 0.057 | 0.061 | 0.853 | 2.121 | 94.782 | 1.000 |  |
| 38 | 10.732 | 1.061 | 0.100 | 0.123 | 0.051 | 0.770 | 0.059 | 0.788 | 0.086 | 0.065 | 0.041 | 0.055 | 0.043 | 0.066 | 0.548 | 1.803 | 95.823 | 1.000 |  |
| 39 | 11.139 | 1.067 | 0.064 | 0.072 | 0.075 | 0.924 | 0.049 | 0.689 | 0.149 | 0.038 | 0.048 | 0.035 | 0.043 | 0.062 | 0.676 | 1.924 | 95.748 | 1.000 |  |
| 40 | 10.677 | 0.9817 | 0.065 | 0.075 | 0.061 | 0.712 | 0.061 | 0.904 | 0.020 | 0.065 | 0.044 | 0.063 | 0.062 | 0.064 | 0.504 | 1.708 | 94.989 | 1.000 |  |
| 41 | 11.086 | 0.818 | 0.061 | 0.060 | 0.080 | 0.403 | 0.032 | 0.958 | 0.062 | 0.069 | 0.065 | 0.051 | 0.040 | 0.060 | 0.986 | 2.140 | 94.286 | 1.000 |  |
| 42 | 11.206 | 0.822 | 0.062 | 0.068 | 0.048 | 0.431 | 0.069 | 0.872 | 0.137 | 0.031 | 0.066 | 0.079 | 0.055 | 0.071 | 1.000 | 2.098 | 95.644 | 1.000 |  |
| 43 | 11.860 | 1.168 | 0.063 | 0.130 | 0.078 | 0.451 | 0.031 | 0.978 | 0.069 | 0.035 | 0.055 | 0.075 | 0.058 | 0.079 | 0.927 | 2.091 | 94.584 | 1.000 |  |
| 44 | 11.083 | 1.160 | 0.129 | 0.119 | 0.063 | 0.560 | 0.035 | 0.529 | 0.130 | 0.064 | 0.056 | 0.064 | 0.057 | 0.078 | 0.537 | 1.827 | 95.498 | 1.000 |  |
| 45 | 8.212 | 1.139 | 0.065 | 0.133 | 0.076 | 0.578 | 0.036 | 0.966 | 0.130 | 0.068 | 0.033 | 0.039 | 0.058 | 0.063 | 0.512 | 1.958 | 94.612 | 1.000 |  |
| 46 | 8.165 | 1.124 | 0.051 | 0.081 | 0.072 | 0.744 | 0.031 | 0.820 | 0.061 | 0.034 | 0.031 | 0.030 | 0.059 | 0.078 | 0.929 | 1.870 | 95.525 | 1.000 |  |
| 47 | 8.240 | 1.147 | 0.123 | 0.146 | 0.075 | 0.653 | 0.031 | 0.605 | 0.064 | 0.034 | 0.035 | 0.036 | 0.055 | 0.063 | 0.971 | 1.690 | 96.720 | 1.000 |  |
| 48 | 11.812 | 1.051 | 0.066 | 0.172 | 0.077 | 0.817 | 0.032 | 0.640 | 0.020 | 0.031 | 0.066 | 0.035 | 0.044 | 0.069 | 0.627 | 1.996 | 94.210 | 1.000 |  |
| 49 | 8.467 | 0.729 | 0.076 | 0.121 | 0.047 | 0.549 | 0.065 | 0.754 | 0.067 | 0.065 | 0.037 | 0.034 | 0.056 | 0.077 | 0.627 | 1.531 | 95.349 | 1.000 |  |
| 50 | 11.029 | 1.160 | 0.061 | 0.053 | 0.079 | 0.412 | 0.076 | 0.944 | 0.139 | 0.068 | 0.069 | 0.068 | 0.042 | 0.078 | 0.549 | 2.098 | 94.278 | 1.000 |  |
| 51 | 8.535 | 1.163 | 0.372 | 0.086 | 0.075 | 0.481 | 0.068 | 0.492 | 0.135 | 0.032 | 0.063 | 0.070 | 0.042 | 0.065 | 0.521 | 2.003 | 94.489 | 1.000 |  |
| 52 | 10.420 | 0.829 | 0.081 | 0.134 | 0.078 | 0.812 | 0.052 | 0.765 | 0.099 | 0.032 | 0.057 | 0.037 | 0.058 | 0.078 | 0.950 | 1.481 | 95.374 | 1.000 |  |
| 53 | 8.260 | 1.092 | 0.140 | 0.120 | 0.084 | 0.901 | 0.061 | 0.892 | 0.096 | 0.067 | 0.061 | 0.074 | 0.053 | 0.077 | 0.542 | 1.654 | 95.480 | 1.000 |  |
| 54 | 8.607 | 1.154 | 0.139 | 0.069 | 0.039 | 0.766 | 0.044 | 0.905 | 0.061 | 0.067 | 0.059 | 0.078 | 0.058 | 0.060 | 0.518 | 1.863 | 94.819 | 1.000 |  |
| 55 | 8.228 | 1.143 | 0.075 | 0.096 | 0.053 | 0.840 | 0.064 | 0.711 | 0.065 | 0.059 | 0.043 | 0.079 | 0.059 | 0.074 | 0.946 | 1.823 | 94.569 | 1.000 |  |
| 56 | 8.414 | 1.000 | 0.106 | 0.110 | 0.075 | 0.612 | 0.049 | 0.823 | 0.067 | 0.055 | 0.034 | 0.072 | 0.056 | 0.078 | 0.927 | 1.924 | 95.768 | 1.000 |  |
| 57 | 11.021 | 1.113 | 0.139 | 0.112 | 0.038 | 0.521 | 0.045 | 0.813 | 0.084 | 0.034 | 0.065 | 0.076 | 0.059 | 0.064 | 0.749 | 2.011 | 94.581 | 1.000 |  |
| 58 | 11.291 | 1.078 | 0.132 | 0.124 | 0.040 | 0.833 | 0.033 | 0.841 | 0.101 | 0.034 | 0.044 | 0.051 | 0.042 | 0.072 | 0.961 | 2.031 | 96.466 | 1.000 |  |
| 59 | 10.699 | 0.897 | 0.129 | 0.116 | 0.070 | 0.865 | 0.060 | 0.561 | 0.122 | 0.054 | 0.045 | 0.047 | 0.047 | 0.067 | 0.635 | 2.010 | 95.181 | 1.000 |  |
| 60 | 11.291 | 0.849 | 0.079 | 0.072 | 0.055 | 0.838 | 0.049 | 0.892 | 0.139 | 0.033 | 0.062 | 0.038 | 0.044 | 0.064 | 0.606 | 1.931 | 95.635 | 1.000 |  |
| 61 | 10.348 | 1.116 | 0.052 | 0.076 | 0.031 | 0.985 | 0.064 | 0.584 | 0.135 | 0.032 | 0.036 | 0.040 | 0.050 | 0.067 | 0.603 | 1.851 | 94.808 | 1.000 |  |
| 62 | 10.665 | 1.023 | 0.083 | 0.130 | 0.076 | 0.845 | 0.047 | 0.907 | 0.135 | 0.055 | 0.037 | 0.069 | 0.048 | 0.072 | 0.837 | 1.994 | 94.619 | 1.000 |  |
| 63 | 10.863 | 0.820 | 0.084 | 0.088 | 0.076 | 0.687 | 0.033 | 0.731 | 0.098 | 0.043 | 0.063 | 0.064 | 0.052 | 0.070 | 0.822 | 1.771 | 95.432 | 1.000 |  |
| 64 | 10.520 | 0.944 | 0.136 | 0.080 | 0.065 | 0.602 | 0.036 | 0.879 | 0.060 | 0.064 | 0.044 | 0.067 | 0.046 | 0.063 | 0.819 | 1.772 | 94.860 | 1.000 |  |
| 65 | 10.072 | 0.982 | 0.099 | 0.128 | 0.052 | 0.832 | 0.033 | 0.727 | 0.124 | 0.057 | 0.059 | 0.064 | 0.049 | 0.069 | 0.563 | 1.277 | 95.023 | 1.000 |  |
| 66 | 10.001 | 0.967 | 0.096 | 0.095 | 0.037 | 0.531 | 0.040 | 0.852 | 0.131 | 0.055 | 0.066 | 0.073 | 0.055 | 0.077 | 0.775 | 1.850 | 94.498 | 1.000 |  |
| 67 | 9.983 | 0.801 | 0.120 | 0.102 | 0.037 | 0.514 | 0.048 | 0.921 | 0.080 | 0.066 | 0.063 | 0.053 | 0.042 | 0.079 | 0.978 | 1.883 | 94.789 | 1.000 |  |
| 68 | 11.038 | 1.118 | 0.067 | 0.070 | 0.068 | 0.541 | 0.043 | 0.945 | 0.124 | 0.053 | 0.054 | 0.051 | 0.058 | 0.067 | 0.917 | 1.742 | 95.278 | 1.000 |  |
| 69 | 8.908 | 0.941 | 0.134 | 0.134 | 0.047 | 0.502 | 0.036 | 0.849 | 0.123 | 0.036 | 0.061 | 0.034 | 0.056 | 0.079 | 0.518 | 1.808 | 94.631 | 1.000 |  |
| 70 | 9.642 | 1.127 | 0.104 | 0.090 | 0.035 | 0.722 | 0.031 | 0.765 | 0.073 | 0.039 | 0.034 | 0.048 | 0.055 | 0.078 | 0.527 | 1.729 | 95.327 | 1.000 |  |
| 71 | 10.848 | 1.116 | 0.079 | 0.071 | 0.041 | 0.626 | 0.036 | 0.574 | 0.126 | 0.039 | 0.065 | 0.064 | 0.052 | 0.076 | 0.974 | 1.816 | 97.227 | 1.000 |  |
| 72 | 9.740 | 1.108 | 0.123 | 0.100 | 0.071 | 0.654 | 0.034 | 0.702 | 0.130 | 0.034 | 0.069 | 0.074 | 0.043 | 0.079 | 0.545 | 1.799 | 96.615 | 1.000 |  |
| 73 | 8.845 | 1.146 | 0.121 | 0.128 | 0.071 | 0.486 | 0.057 | 0.662 | 0.081 | 0.062 | 0.058 | 0.055 | 0.051 | 0.072 | 0.586 | 2.045 | 95.811 | 1.000 |  |
| 74 | 11.272 | 0.873 | 0.064 | 0.071 | 0.074 | 0.684 | 0.052 | 0.618 | 0.131 | 0.038 | 0.046 | 0.059 | 0.053 | 0.066 | 0.800 | 2.111 | 95.866 | 1.000 |  |
| 75 | 8.681 | 1.071 | 0.061 | 0.071 | 0.049 | 0.865 | 0.067 | 0.771 | 0.130 | 0.051 | 0.043 | 0.054 | 0.040 | 0.072 | 0.678 | 1.917 | 95.806 | 1.000 |  |
| 76 | 11.182 | 0.847 | 0.091 | 0.130 | 0.053 | 0.631 | 0.045 | 0.709 | 0.089 | 0.039 | 0.062 | 0.033 | 0.045 | 0.086 | 0.659 | 1.775 | 94.816 | 1.000 |  |
| 77 | 11.611 | 1.110 | 0.122 | 0.083 | 0.045 | 0.724 | 0.034 | 0.798 | 0.130 | 0.056 | 0.045 | 0.033 | 0.042 | 0.080 | 0.548 | 2.124 | 95.572 | 1.000 |  |
| 78 | 10.925 | 1.098 | 0.094 | 0.130 | 0.075 | 0.542 | 0.034 | 0.845 | 0.078 | 0.035 | 0.061 | 0.039 | 0.050 | 0.065 | 0.552 | 1.891 | 94.874 | 1.000 |  |
| 79 | 10.397 | 0.870 | 0.234 | 0.135 | 0.068 | 0.763 | 0.065 | 0.974 | 0.130 | 0.066 | 0.048 | 0.046 | 0.045 | 0.063 | 0.923 | 2.064 | 95.054 | 1.000 |  |
| 80 | 11.028 | 0.872 | 0.089 | 0.062 | 0.077 | 0.726 | 0.069 | 0.652 | 0.104 | 0.031 | 0.068 | 0.074 | 0.059 | 0.062 | 0.564 | 1.982 | 95.475 | 1.000 |  |
| 81 | 9.699 | 0.917 | 0.057 | 0.132 | 0.036 | 0.880 | 0.042 | 0.845 | 0.086 | 0.065 | 0.040 | 0.031 | 0.044 | 0.062 | 0.891 | 1.813 | 95.617 | 1.000 |  |
| 82 | 8.920 | 0.828 | 0.144 | 0.070 | 0.046 | 0.474 | 0.068 | 0.843 | 0.120 | 0.051 | 0.059 | 0.046 | 0.040 | 0.065 | 0.762 | 1.701 | 94.828 | 1.000 |  |
| 83 | 11.095 | 0.910 | 0.095 | 0.065 | 0.034 | 0.545 | 0.066 | 0.691 | 0.065 | 0.065 | 0.069 | 0.077 | 0.056 | 0.065 | 0.921 | 1.850 | 95.721 | 1.000 |  |
| 84 | 9.729 | 1.044 | 0.116 | 0.091 | 0.068 | 0.572 | 0.034 | 0.727 | 0.095 | 0.056 | 0.039 | 0.046 | 0.058 | 0.078 | 0.941 | 1.628 | 95.232 | 1.000 |  |
| 85 | 9.688 | 0.977 | 0.094 | 0.082 | 0.069 | 0.892 | 0.051 | 0.928 | 0.076 | 0.054 | 0.036 | 0.037 | 0.053 | 0.077 | 0.792 | 1.795 | 94.426 | 1.000 |  |
| 86 | 11.298 | 0.969 | 0.140 | 0.137 | 0.064 | 0.787 | 0.070 | 0.886 | 0.140 | 0.070 | 0.048 | 0.064 | 0.041 | 0.072 | 0.968 | 1.826 | 94.522 | 1.000 |  |
| 87 | 9.765 | 0.954 | 0.128 | 0.095 | 0.053 | 0.873 | 0.044 | 0.564 | 0.113 | 0.034 | 0.045 | 0.041 | 0.049 | 0.077 | 0.923 | 1.823 | 96.315 | 1.000 |  |
| 88 | 10.844 | 0.954 | 0.091 | 0.104 | 0.068 | 0.710 | 0.041 | 0.730 | 0.084 | 0.065 | 0.051 | 0.053 | 0.056 | 0.074 | 0.876 | 1.515 | 96.166 | 1.000 |  |
| 89 | 9.637 | 1.182 | 0.133 | 0.138 | 0.075 | 0.519 | 0.061 | 0.795 | 0.127 | 0.037 | 0.047 | 0.042 | 0.068 | 0.064 | 0.568 | 1.937 | 94.232 | 1.000 |  |
| 90 | 10.675 | 1.137 | 0.196 | 0.144 | 0.047 | 0.899 | 0.056 | 0.768 | 0.070 | 0.048 | 0.048 | 0.074 | 0.051 | 0.070 | 0.661 | 1.905 | 96.120 | 1.000 |  |
| 91 | 8.970 | 1.042 | 0.074 | 0.097 | 0.074 | 0.728 | 0.057 | 0.631 | 0.085 | 0.036 | 0.064 | 0.073 | 0.054 | 0.072 | 0.652 | 1.865 | 95.424 | 1.000 |  |
| 92 | 8.934 | 1.125 | 0.137 | 0.078 | 0.051 | 0.765 | 0.044 | 0.831 | 0.064 | 0.039 | 0.035 | 0.078 | 0.048 | 0.079 | 0.530 | 1.961 | 95.485 | 1.000 |  |
| 93 | 9.608 | 0.838 | 0.081 | 0.114 | 0.071 | 0.823 | 0.031 | 0.571 | 0.068 | 0.038 | 0.055 | 0.058 | 0.043 | 0.066 | 0.959 | 1.841 | 94.280 | 1.000 |  |
| 94 | 10.680 | 1.021 | 0.073 | 0.078 | 0.033 | 0.869 | 0.052 | 0.745 | 0.101 | 0.057 | 0.059 | 0.046 | 0.043 | 0.080 | 0.942 | 1.499 | 95.630 | 1.000 |  |
| 95 | 10.278 | 1.041 | 0.139 | 0.138 | 0.041 | 0.682 | 0.050 | 0.661 | 0.169 | 0.067 | 0.046 | 0.044 | 0.057 | 0.067 | 0.572 | 1.769 | 95.631 | 1.000 |  |
| 96 | 10.629 | 0.843 | 0.056 | 0.114 | 0.039 | 0.586 | 0.060 | 0.741 | 0.121 | 0.066 | 0.065 | 0.057 | 0.047 | 0.061 | 0.966 | 2.094 | 95.743 | 1.000 |  |
| 97 | 9.347 | 1.002 | 0.062 | 0.100 | 0.054 | 0.945 | 0.065 | 0.653 | 0.065 | 0.057 | 0.070 | 0.074 | 0.041 | 0.061 | 0.566 | 1.722 | 95.512 | 1.000 |  |
| 98 | 11.218 | 1.093 | 0.098 | 0.097 | 0.073 | 0.834 | 0.039 | 0.911 | 0.092 | 0.035 | 0.038 | 0.040 | 0.047 | 0.066 | 0.544 | 1.813 | 94.926 | 1.000 |  |
| 99 | 8.929 | 1.056 | 0.077 | 0.112 | 0.036 | 0.888 | 0.034 | 0.793 | 0.115 | 0.056 | 0.059 | 0.073 | 0.052 | 0.065 | 0.956 | 1.793 | 95.286 | 1.000 |  |
| 100 | 11.073 | 0.881 | 0.067 | 0.066 | 0.073 | 0.699 | 0.064 | 0.823 | 0.073 | 0.035 | 0.068 | 0.069 | 0.042 | 0.063 | 0.925 | 1.701 | 96.618 | 1.000 |  |

**S Table 8:**

**Optimum condition, Experimental and Predicted Values of Cd(II) biosorption (%) at Optimized Conditions**

| **Optimum Conditions** | | **Coded Levels** |  | **Actual Levels** | |
| --- | --- | --- | --- | --- | --- |
| Glucose concentration (%) | | 0.239 |  | 10.748 |  |
| Urea concentration (%) | | 0.355 |  | 1.071 |  |
| K_2_HPO_4_ concentration (%) | | 0.675 |  | 0.127 |  |
| KH_2_PO_4_ concentration (%) | | 0.55 |  | 0.122 |  |
| MgSO_4_.7H_2_O concentration (%) | | 0.08 |  | 0.057 |  |
| KCl concentration(%) | | 0.273 |  | 0.864 |  |
| CoCl_2_. 5H_2_O concentration (%) | | -0.57 |  | 0.027 |  |
| NH_4_VO_2_ concentration (%) | | 0.014 |  | 0.757 |  |
| Na_2_MoO_4_.2H_2_O concentration (%) | | -0.6 |  | 0.047 |  |
| CaCO_3_ concentration (%) | | 0.1 |  | 0.054 |  |
| FeSO_4_.7H_2_O concentration (%) | | 0.05 |  | 0.052 |  |
| ZnSO_4_.7H_2_O concentration (%) | | 0.04 |  | 0.057 |  |
| MnSO_4_.4H_2_O concentration (%) | | 0.35 |  | 0.047 |  |
| NiSO_4_.7H_2_O concentration (%) | | -1.65 |  | 0.037 |  |
| Na_2_B_4_O_7_.10H_2_O concentration (%) | | -0.35 |  | 0.571 |  |
|  |  |  |  |  |  |
| **Response** | | Predicted Values | | Experimental Values | |
| Cd(II) biosorption (%) | | 95.028 |  | 95.972±0.0001 | |

**S Table 9:**

**Selection of Synthetic Media**

| **Ingredients** | **Amount** |
| --- | --- |
| Glucose concentration (%) | 10.748 |
| Urea concentration (%) | 1.071 |
| K_2_HPO_4_ concentration (%) | 0.127 |
| KH_2_PO_4_ concentration (%) | 0.122 |
| MgSO_4_.7H_2_O concentration (%) | 0.057 |
| KCl concentration(%) | 0.864 |
| CoCl_2._ 5H_2_O concentration (%) | 0.027 |
| NH_4_VO_2_ concentration (%) | 0.757 |
| Na_2_MoO_4._ 2H_2_O concentration (%) | 0.047 |
| CaCO_3_ concentration (%) | 0.054 |
| FeSO_4_.7H_2_O concentration (%) | 0.052 |
| ZnSO_4_.7H_2_O concentration (%) | 0.057 |
| MnSO_4_. 4H_2_O concentration (%) | 0.047 |
| NiSO_4_.7H_2_O concentration (%) | 0.037 |
| Na_2_B_4_O_7_.10H_2_O concentration (%) | 0.571 |

**S Table 10:**

**Kinetic analysis of Cd(II) biosorption using 100ppm Cd(II) Before Optimization**

| **Experiment No.** | **Time (min)** | **C_i_ (ppm)** | **C_e_ (ppm)** | **C_i_-C_e_** | **Adsorbed** | **Absorbed** | **Intracellular Accumulation (mg/g)** | **q_t_ (mg/g)/Adsorbed** | **t/q_t_** | **ln (q_e_-q_t_)** |
| --- | --- | --- | --- | --- | --- | --- | --- | --- | --- | --- |
| 1 | 0 | 0 | 0 | 0 | 0 | 0 | 0 | 0 | 0 | 0 |
| 2 | 40 | 100 | 25.26 | 74.74 | 74.72 | 0.02 | 0.01333 | 49.813333 | 0.803 | 1.082934 |
| 3 | 60 | 100 | 24.56 | 75.44 | 75.39 | 0.05 | 0.03333 | 50.26 | 1.19379 | 0.918954 |
| 4 | 80 | 100 | 23.43 | 76.57 | 76.53 | 0.04 | 0.02667 | 51.02 | 1.56801 | 0.557709 |
| 5 | 100 | 100 | 22.37 | 77.63 | 77.58 | 0.05 | 0.03333 | 51.72 | 1.93349 | 0.045611 |
| 6 | 150 | 100 | 21.28 | 78.72 | 78.68 | 0.04 | 0.02667 | 52.453333 | 2.85968 | -1.16049 |
| 7 | 180 | 100 | 21.28 | 78.72 | 78.68 | 0.04 | 0.02667 | 52.453333 | 3.43162 | -1.16049 |
| 8 | 200 | 100 | 21.28 | 78.72 | 78.68 | 0.04 | 0.02667 | 52.453333 | 3.81291 | -1.16049 |
| 9 | 250 | 100 | 21.28 | 78.72 | 78.68 | 0.04 | 0.02667 | 52.453333 | 4.76614 | -1.16049 |
| 10 | 280 | 100 | 21.28 | 78.72 | 78.68 | 0.04 | 0.02667 | 52.453333 | 5.33808 | -1.16049 |
| 11 | 300 | 100 | 21.28 | 78.72 | 78.68 | 0.04 | 0.02667 | 52.453333 | 5.71937 | -1.16049 |
| 12 | 350 | 100 | 21.28 | 78.72 | 78.68 | 0.04 | 0.02667 | 52.453333 | 6.6726 | -1.16049 |
| 13 | 380 | 100 | 21.28 | 78.72 | 78.68 | 0.04 | 0.02667 | 52.453333 | 7.24453 | -1.16049 |
| 14 | 400 | 100 | 21.28 | 78.72 | 78.68 | 0.04 | 0.02667 | 52.453333 | 7.62583 | -1.16049 |
| 15 | 450 | 100 | 21.28 | 78.72 | 78.68 | 0.04 | 0.02667 | 52.453333 | 8.57905 | -1.16049 |
| 16 | 480 | 100 | 21.28 | 78.72 | 78.68 | 0.04 | 0.02667 | 52.453333 | 9.15099 | -1.16049 |
| 17 | 500 | 100 | 21.28 | 78.72 | 78.68 | 0.04 | 0.02667 | 52.453333 | 9.53228 | -1.16049 |

**S Table 11:**

**Kinetic analysis of Cd(II) biosorption using 250ppm Cd(II) Before Optimization**

| **Experiment No.** | **Time (min)** | **C_i_ (ppm)** | **C_e_ (ppm)** | **C_i_-C_e_** | **Adsorbed** | **Absorbed** | **Intracellular Accumulation (mg/g)** | **q_t_ (mg/g)/Adsorbed** | **t/q_t_** | **ln (q_e_-q_t_)** |
| --- | --- | --- | --- | --- | --- | --- | --- | --- | --- | --- |
| 1 | 0 | 0 | 0 | 0 | 0 | 0 | 0 | 0 | 0 | 0 |
| 2 | 40 | 250 | 70.35 | 179.65 | 179.61 | 0.04 | 0.0267 | 119.74 | 0.33406 | 1.583778 |
| 3 | 60 | 250 | 68.28 | 181.72 | 181.69 | 0.03 | 0.02 | 121.126667 | 0.49535 | 1.248946 |
| 4 | 80 | 250 | 67.34 | 182.66 | 182.64 | 0.02 | 0.0133 | 121.76 | 0.65703 | 1.048488 |
| 5 | 100 | 250 | 66.57 | 183.43 | 183.41 | 0.02 | 0.0133 | 122.273333 | 0.81784 | 0.850151 |
| 6 | 150 | 250 | 66.42 | 183.58 | 183.57 | 0.01 | 0.0067 | 122.38 | 1.22569 | 0.803495 |
| 7 | 180 | 250 | 66.42 | 183.58 | 183.57 | 0.01 | 0.0067 | 122.38 | 1.47083 | 0.803495 |
| 8 | 200 | 250 | 66.42 | 183.58 | 183.57 | 0.01 | 0.0067 | 122.38 | 1.63425 | 0.803495 |
| 9 | 250 | 250 | 66.42 | 183.58 | 183.57 | 0.01 | 0.0067 | 122.38 | 2.04282 | 0.803495 |
| 10 | 280 | 250 | 66.42 | 183.58 | 183.57 | 0.01 | 0.0067 | 122.38 | 2.28796 | 0.803495 |
| 11 | 300 | 250 | 66.42 | 183.58 | 183.57 | 0.01 | 0.0067 | 122.38 | 2.45138 | 0.803495 |
| 12 | 350 | 250 | 66.42 | 183.58 | 183.57 | 0.01 | 0.0067 | 122.38 | 2.85994 | 0.803495 |
| 13 | 380 | 250 | 66.42 | 183.58 | 183.57 | 0.01 | 0.0067 | 122.38 | 3.10508 | 0.803495 |
| 14 | 400 | 250 | 66.42 | 183.58 | 183.57 | 0.01 | 0.0067 | 122.38 | 3.26851 | 0.803495 |
| 15 | 450 | 250 | 66.42 | 183.58 | 183.57 | 0.01 | 0.0067 | 122.38 | 3.67707 | 0.803495 |
| 16 | 480 | 250 | 66.42 | 183.58 | 183.57 | 0.01 | 0.0067 | 122.38 | 3.92221 | 0.803495 |
| 17 | 500 | 250 | 66.42 | 183.58 | 183.57 | 0.01 | 0.0067 | 122.38 | 4.08563 | 0.803495 |

**S Table 12:**

**Kinetic analysis of Cd(II) biosorption using 300ppm Cd(II) Before Optimization**

| **Experiment No.** | **Time (min)** | **C_i_ (ppm)** | **C_e_ (ppm)** | **C_i_-C_e_** | **Adsorbed** | **Absorbed** | **Intracellular Accumulation (mg/g)** | **q_t_ (mg/g)/Adsorbed** | **t/q_t_** | **ln (q_e_-q_t_)** |
| --- | --- | --- | --- | --- | --- | --- | --- | --- | --- | --- |
| 1 | 0 | 0 | 0 | 0 | 0 | 0 | 0 | 0 | 0 | 0 |
| 2 | 40 | 300 | 115.46 | 184.54 | 184.52 | 0.02 | 0.0133 | 123.0133333 | 0.325168 | 1.2237754 |
| 3 | 60 | 300 | 114.27 | 185.73 | 185.7 | 0.03 | 0.02 | 123.8 | 0.484653 | 0.9606265 |
| 4 | 80 | 300 | 113.15 | 186.85 | 186.81 | 0.04 | 0.0267 | 124.54 | 0.642364 | 0.6277194 |
| 5 | 100 | 300 | 112.57 | 187.43 | 187.38 | 0.05 | 0.0333 | 124.92 | 0.800512 | 0.4010108 |
| 6 | 150 | 300 | 112.45 | 187.55 | 187.49 | 0.06 | 0.04 | 124.9933333 | 1.200064 | 0.3506569 |
| 7 | 180 | 300 | 112.45 | 187.55 | 187.49 | 0.06 | 0.04 | 124.9933333 | 1.440077 | 0.3506569 |
| 8 | 200 | 300 | 112.45 | 187.55 | 187.49 | 0.06 | 0.04 | 124.9933333 | 1.600085 | 0.3506569 |
| 9 | 250 | 300 | 112.45 | 187.55 | 187.49 | 0.06 | 0.04 | 124.9933333 | 2.000107 | 0.3506569 |
| 10 | 280 | 300 | 112.45 | 187.55 | 187.49 | 0.06 | 0.04 | 124.9933333 | 2.240119 | 0.3506569 |
| 11 | 300 | 300 | 112.45 | 187.55 | 187.49 | 0.06 | 0.04 | 124.9933333 | 2.400128 | 0.3506569 |
| 12 | 350 | 300 | 112.45 | 187.55 | 187.49 | 0.06 | 0.04 | 124.9933333 | 2.800149 | 0.3506569 |
| 13 | 380 | 300 | 112.45 | 187.55 | 187.49 | 0.06 | 0.04 | 124.9933333 | 3.040162 | 0.3506569 |
| 14 | 400 | 300 | 112.45 | 187.55 | 187.49 | 0.06 | 0.04 | 124.9933333 | 3.200171 | 0.3506569 |
| 15 | 450 | 300 | 112.45 | 187.55 | 187.49 | 0.06 | 0.04 | 124.9933333 | 3.600192 | 0.3506569 |
| 16 | 480 | 300 | 112.45 | 187.55 | 187.49 | 0.06 | 0.04 | 124.9933333 | 3.840205 | 0.3506569 |
| 17 | 500 | 300 | 112.45 | 187.55 | 187.49 | 0.06 | 0.04 | 124.9933333 | 4.000213 | 0.3506569 |

**S Table 13:**

**Kinetic analysis of Cd(II) biosorption using 500ppm Cd(II) Before Optimization**

| **Experiment No.** | **Time (min)** | **C_i_ (ppm)** | **C_e_ (ppm)** | **C_i_-C_e_** | **Adsorbed** | **Absorbed** | **Intracellular Accumulation (mg/g)** | **q_t_ (mg/g)/Adsorbed** | **t/q_t_** | **ln (q_e_-q_t_)** |
| --- | --- | --- | --- | --- | --- | --- | --- | --- | --- | --- |
| 1 | 0 | 0 | 0 | 0 | 0 | 0 | 0 | 0 | 0 | 0 |
| 2 | 40 | 500 | 124.26 | 375.74 | 375.72 | 0.02 | 0.013333333 | 250.48 | 0.159693 | 0.587786665 |
| 3 | 60 | 500 | 123.48 | 376.52 | 376.49 | 0.03 | 0.02 | 250.9933333 | 0.23905 | 0.252054895 |
| 4 | 80 | 500 | 123.25 | 376.75 | 376.72 | 0.03 | 0.02 | 251.1466667 | 0.318539 | 0.125163143 |
| 5 | 100 | 500 | 123.13 | 376.87 | 376.82 | 0.05 | 0.033333333 | 251.2133333 | 0.398068 | 0.064538521 |
| 6 | 150 | 500 | 122.48 | 377.52 | 377.46 | 0.06 | 0.04 | 251.64 | 0.59609 | -0.446287103 |
| 7 | 180 | 500 | 122.48 | 377.52 | 377.46 | 0.06 | 0.04 | 251.64 | 0.715308 | -0.446287103 |
| 8 | 200 | 500 | 122.48 | 377.52 | 377.46 | 0.06 | 0.04 | 251.64 | 0.794786 | -0.446287103 |
| 9 | 250 | 500 | 122.48 | 377.52 | 377.46 | 0.06 | 0.04 | 251.64 | 0.993483 | -0.446287103 |
| 10 | 280 | 500 | 122.48 | 377.52 | 377.46 | 0.06 | 0.04 | 251.64 | 1.112701 | -0.446287103 |
| 11 | 300 | 500 | 122.48 | 377.52 | 377.46 | 0.06 | 0.04 | 251.64 | 1.192179 | -0.446287103 |
| 12 | 350 | 500 | 122.48 | 377.52 | 377.46 | 0.06 | 0.04 | 251.64 | 1.390876 | -0.446287103 |
| 13 | 380 | 500 | 122.48 | 377.52 | 377.46 | 0.06 | 0.04 | 251.64 | 1.510094 | -0.446287103 |
| 14 | 400 | 500 | 122.48 | 377.52 | 377.46 | 0.06 | 0.04 | 251.64 | 1.589572 | -0.446287103 |
| 15 | 450 | 500 | 122.48 | 377.52 | 377.46 | 0.06 | 0.04 | 251.64 | 1.788269 | -0.446287103 |
| 16 | 480 | 500 | 122.48 | 377.52 | 377.46 | 0.06 | 0.04 | 251.64 | 1.907487 | -0.446287103 |
| 17 | 500 | 500 | 122.48 | 377.52 | 377.46 | 0.06 | 0.04 | 251.64 | 1.986966 | -0.446287103 |

**Table 14:**

**Values of the parameters of kinetic models for Cd(II) adsorption of *Candida tropicalis* XTA 1874 Before Optimization**

| **Pseudo First Order** | | | | | | **Pseudo Second Order** | | | |
| --- | --- | --- | --- | --- | --- | --- | --- | --- | --- |
| **Metal ion (ppm)** | **q_e_,exp (mg/g)** | **k_1_** | **R^2^** | **q_e_,cal (mg/g)** |  | **k_2_** | **R^2^** | **q_e_, cal (mg/g)** |  |
| 100 | 52.864± 0.044 | -0.00014±1.98E^-06^ | 0.978 | 8.227±0.115 |  | 0.012±0.001 | 0.999 | 53.102±0.202 |  |
| 250 | 124.616± 0.002 | -0.00012±2.4E^-05^ | 0.8 | 5.431±0.306 |  | 0.018±0.0004 | 1 | 122.784±0.148 |  |
| 300 | 126. 415± 0.001 | -5.4E^-05^±2.65E^-07^ | 0.817 | 4.074±0.015 |  | 0.025±0.002 | 1 | 125.157± 0.001 |  |
| 500 | 252.287±0.006 | -5.8E^-05^±2.77E^-07^ | 0.964 | 2.375±0.002 |  | 0.028±0.001 | 1 | 251.905±0.010 |  |

**S Table 15:**

**Kinetic analysis of Cd(II) biosorption using 100ppm Cd(II) After Optimization**

| **Experiment No.** | **Time (min)** | **C_i_ (ppm)** | **C_e_ (ppm)** | **C_i_-C_e_** | **Adsorbed** | **Absorbed** | **Intracellular Accumulation (mg/g)** | **q_t_ (mg/g)/Adsorbed** | **t/q_t_** | **ln (q_e_-q_t_)** |
| --- | --- | --- | --- | --- | --- | --- | --- | --- | --- | --- |
| 1 | 0 | 0 | 0 | 0 | 0 | 0 | 0 | 0 | 0 | 0 |
| 2 | 40 | 100 | 15.46 | 84.54 | 84.53 | 0.01 | 0.006666667 | 56.35333333 | 0.709807169 | 1.207964826 |
| 3 | 60 | 100 | 14.93 | 85.07 | 85.06 | 0.01 | 0.006666667 | 56.70666667 | 1.058076652 | 1.096387594 |
| 4 | 80 | 100 | 14.53 | 85.47 | 85.45 | 0.02 | 0.013333333 | 56.96666667 | 1.404330018 | 1.005521866 |
| 5 | 100 | 100 | 13.68 | 86.32 | 86.3 | 0.02 | 0.013333333 | 57.53333333 | 1.738122827 | 0.773189888 |
| 6 | 150 | 100 | 13.22 | 86.78 | 86.75 | 0.03 | 0.02 | 57.83333333 | 2.593659942 | 0.624154309 |
| 7 | 180 | 100 | 13.22 | 86.78 | 86.75 | 0.03 | 0.02 | 57.83333333 | 3.112391931 | 0.624154309 |
| 8 | 200 | 100 | 13.22 | 86.78 | 86.75 | 0.03 | 0.02 | 57.83333333 | 3.458213256 | 0.624154309 |
| 9 | 250 | 100 | 13.22 | 86.78 | 86.75 | 0.03 | 0.02 | 57.83333333 | 4.322766571 | 0.624154309 |
| 10 | 280 | 100 | 13.22 | 86.78 | 86.75 | 0.03 | 0.02 | 57.83333333 | 4.841498559 | 0.624154309 |
| 11 | 300 | 100 | 13.22 | 86.78 | 86.75 | 0.03 | 0.02 | 57.83333333 | 5.187319885 | 0.624154309 |
| 12 | 350 | 100 | 13.22 | 86.78 | 86.75 | 0.03 | 0.02 | 57.83333333 | 6.051873199 | 0.624154309 |
| 13 | 380 | 100 | 13.22 | 86.78 | 86.75 | 0.03 | 0.02 | 57.83333333 | 6.570605187 | 0.624154309 |
| 14 | 400 | 100 | 13.22 | 86.78 | 86.75 | 0.03 | 0.02 | 57.83333333 | 6.916426513 | 0.624154309 |
| 15 | 450 | 100 | 13.22 | 86.78 | 86.75 | 0.03 | 0.02 | 57.83333333 | 7.780979827 | 0.624154309 |
| 16 | 480 | 100 | 13.22 | 86.78 | 86.75 | 0.03 | 0.02 | 57.83333333 | 8.299711816 | 0.624154309 |
| 17 | 500 | 100 | 13.22 | 86.78 | 86.75 | 0.03 | 0.02 | 57.83333333 | 8.645533141 | 0.624154309 |

**S Table 16:**

**Kinetic analysis of Cd(II) biosorption using 250ppm Cd(II) After Optimization**

| **Experiment No.** | **Time (min)** | **C_i_ (ppm)** | **C_e_ (ppm)** | **C_i_-C_e_** | **Adsorbed** | **Absorbed** | **Intracellular Accumulation (mg/g)** | **q_t_ (mg/g)/Adsorbed** | **t/q_t_** | **ln (q_e_-q_t_)** |
| --- | --- | --- | --- | --- | --- | --- | --- | --- | --- | --- |
| 1 | 0 | 0 | 0 | 0 | 0 | 0 | 0 | 0 | 0 | 0 |
| 2 | 40 | 250 | 46.67 | 203.33 | 203.32 | 0.01 | 0.006667 | 135.546667 | 0.295101 | 0.757686 |
| 3 | 60 | 250 | 45.75 | 204.25 | 204.24 | 0.01 | 0.006667 | 136.16 | 0.440658 | 0.41871 |
| 4 | 80 | 250 | 44.64 | 205.36 | 205.35 | 0.01 | 0.006667 | 136.9 | 0.584368 | -0.24846 |
| 5 | 100 | 250 | 44.57 | 205.43 | 205.41 | 0.02 | 0.013333 | 136.94 | 0.730247 | -0.30111 |
| 6 | 150 | 250 | 43.48 | 206.52 | 206.49 | 0.03 | 0.02 | 137.66 | 1.089641 | -3.91202 |
| 7 | 180 | 250 | 43.48 | 206.52 | 206.49 | 0.03 | 0.02 | 137.66 | 1.307569 | -3.91202 |
| 8 | 200 | 250 | 43.48 | 206.52 | 206.49 | 0.03 | 0.02 | 137.66 | 1.452855 | -3.91202 |
| 9 | 250 | 250 | 43.48 | 206.52 | 206.49 | 0.03 | 0.02 | 137.66 | 1.816069 | -3.91202 |
| 10 | 280 | 250 | 43.48 | 206.52 | 206.49 | 0.03 | 0.02 | 137.66 | 2.033997 | -3.91202 |
| 11 | 300 | 250 | 43.48 | 206.52 | 206.49 | 0.03 | 0.02 | 137.66 | 2.179282 | -3.91202 |
| 12 | 350 | 250 | 43.48 | 206.52 | 206.49 | 0.03 | 0.02 | 137.66 | 2.542496 | -3.91202 |
| 13 | 380 | 250 | 43.48 | 206.52 | 206.49 | 0.03 | 0.02 | 137.66 | 2.760424 | -3.91202 |
| 14 | 400 | 250 | 43.48 | 206.52 | 206.49 | 0.03 | 0.02 | 137.66 | 2.90571 | -3.91202 |
| 15 | 450 | 250 | 43.48 | 206.52 | 206.49 | 0.03 | 0.02 | 137.66 | 3.268923 | -3.91202 |
| 16 | 480 | 250 | 43.48 | 206.52 | 206.49 | 0.03 | 0.02 | 137.66 | 3.486852 | -3.91202 |
| 17 | 500 | 250 | 43.48 | 206.52 | 206.49 | 0.03 | 0.02 | 137.66 | 3.632137 | -3.91202 |

**S Table 17:**

**Kinetic analysis of Cd(II) biosorption using 300ppm Cd(II) After Optimization**

| **Experiment No.** | **Time (min)** | **C_i_ (ppm)** | **C_e_ (ppm)** | **C_i_-C_e_** | **Adsorbed** | **Absorbed** | **Intracellular Accumulation (mg/g)** | **q_t_ (mg/g)/Adsorbed** | **t/q_t_** | **ln (q_e_-q_t_)** |
| --- | --- | --- | --- | --- | --- | --- | --- | --- | --- | --- |
| 1 | 0 | 0 | 0 | 0 | 0 | 0 | 0 | 0 | 0 | 0 |
| 2 | 40 | 300 | 92.45 | 207.55 | 207.54 | 0.01 | 0.006666667 | 138.36 | 0.289101 | 1.39954 |
| 3 | 60 | 300 | 91.38 | 208.62 | 208.6 | 0.02 | 0.013333333 | 139.0666667 | 0.431448 | 1.207965 |
| 4 | 80 | 300 | 90.74 | 209.26 | 209.24 | 0.02 | 0.013333333 | 139.4933333 | 0.573504 | 1.071584 |
| 5 | 100 | 300 | 89.35 | 210.65 | 210.62 | 0.03 | 0.02 | 140.4133333 | 0.712183 | 0.693147 |
| 6 | 150 | 300 | 89.28 | 210.72 | 210.69 | 0.03 | 0.02 | 140.46 | 1.06792 | 0.669537 |
| 7 | 180 | 300 | 89.28 | 210.72 | 210.69 | 0.03 | 0.02 | 140.46 | 1.281504 | 0.669537 |
| 8 | 200 | 300 | 89.28 | 210.72 | 210.69 | 0.03 | 0.02 | 140.46 | 1.423893 | 0.669537 |
| 9 | 250 | 300 | 89.28 | 210.72 | 210.69 | 0.03 | 0.02 | 140.46 | 1.779866 | 0.669537 |
| 10 | 280 | 300 | 89.28 | 210.72 | 210.69 | 0.03 | 0.02 | 140.46 | 1.99345 | 0.669537 |
| 11 | 300 | 300 | 89.28 | 210.72 | 210.69 | 0.03 | 0.02 | 140.46 | 2.135839 | 0.669537 |
| 12 | 350 | 300 | 89.28 | 210.72 | 210.69 | 0.03 | 0.02 | 140.46 | 2.491813 | 0.669537 |
| 13 | 380 | 300 | 89.28 | 210.72 | 210.69 | 0.03 | 0.02 | 140.46 | 2.705397 | 0.669537 |
| 14 | 400 | 300 | 89.28 | 210.72 | 210.69 | 0.03 | 0.02 | 140.46 | 2.847786 | 0.669537 |
| 15 | 450 | 300 | 89.28 | 210.72 | 210.69 | 0.03 | 0.02 | 140.46 | 3.203759 | 0.669537 |
| 16 | 480 | 300 | 89.28 | 210.72 | 210.69 | 0.03 | 0.02 | 140.46 | 3.417343 | 0.669537 |
| 17 | 500 | 300 | 89.28 | 210.72 | 210.69 | 0.03 | 0.02 | 140.46 | 3.559732 | 0.669537 |

**S Table 18:**

**Kinetic analysis of Cd(II) biosorption using 500ppm Cd(II) After Optimization**

| **Experiment No.** | **Time (min)** | **C_i_ (ppm)** | **C_e_ (ppm)** | **C_i_-C_e_** | **Adsorbed** | **Absorbed** | **Intracellular Accumulation (mg/g)** | **q_t_ (mg/g)/Adsorbed** | **t/q_t_** | **ln (q_e_-q_t_)** |
| --- | --- | --- | --- | --- | --- | --- | --- | --- | --- | --- |
| 1 | 0 | 0 | 0 | 0 | 0 | 0 | 0 | 0 | 0 | 0 |
| 2 | 40 | 500 | 24.28 | 475.72 | 475.71 | 0.01 | 0.006667 | 317.14 | 0.126127 | 0.905567 |
| 3 | 60 | 500 | 23.68 | 476.32 | 476.31 | 0.01 | 0.006667 | 317.54 | 0.188953 | 0.729158 |
| 4 | 80 | 500 | 23.25 | 476.75 | 476.73 | 0.02 | 0.013333 | 317.82 | 0.251715 | 0.584076 |
| 5 | 100 | 500 | 22.64 | 477.36 | 477.34 | 0.02 | 0.013333 | 318.2267 | 0.314241 | 0.326903 |
| 6 | 150 | 500 | 22.24 | 477.76 | 477.74 | 0.02 | 0.013333 | 318.4933 | 0.470967 | 0.113329 |
| 7 | 180 | 500 | 22.24 | 477.76 | 477.74 | 0.02 | 0.013333 | 318.4933 | 0.565161 | 0.113329 |
| 8 | 200 | 500 | 22.24 | 477.76 | 477.74 | 0.02 | 0.013333 | 318.4933 | 0.627957 | 0.113329 |
| 9 | 250 | 500 | 22.24 | 477.76 | 477.74 | 0.02 | 0.013333 | 318.4933 | 0.784946 | 0.113329 |
| 10 | 280 | 500 | 22.24 | 477.76 | 477.74 | 0.02 | 0.013333 | 318.4933 | 0.879139 | 0.113329 |
| 11 | 300 | 500 | 22.24 | 477.76 | 477.74 | 0.02 | 0.013333 | 318.4933 | 0.941935 | 0.113329 |
| 12 | 350 | 500 | 22.24 | 477.76 | 477.74 | 0.02 | 0.013333 | 318.4933 | 1.098924 | 0.113329 |
| 13 | 380 | 500 | 22.24 | 477.76 | 477.74 | 0.02 | 0.013333 | 318.4933 | 1.193118 | 0.113329 |
| 14 | 400 | 500 | 22.24 | 477.76 | 477.74 | 0.02 | 0.013333 | 318.4933 | 1.255913 | 0.113329 |
| 15 | 450 | 500 | 22.24 | 477.76 | 477.74 | 0.02 | 0.013333 | 318.4933 | 1.412902 | 0.113329 |
| 16 | 480 | 500 | 22.24 | 477.76 | 477.74 | 0.02 | 0.013333 | 318.4933 | 1.507096 | 0.113329 |
| 17 | 500 | 500 | 22.24 | 477.76 | 477.74 | 0.02 | 0.013333 | 318.4933 | 1.569892 | 0.113329 |

**Table 19:**

**Values of the parameters of kinetic models for Cd(II) adsorption of *Candida tropicalis* XTA 1874 After Optimization**

| **Pseudo First Order** | | | | | | | **Pseudo Second Order** | | | |
| --- | --- | --- | --- | --- | --- | --- | --- | --- | --- | --- |
| **Metal ion (ppm)** | **q_e_,exp (mg/g)** | **k_1_** | | **R^2^** | **q_e_,cal (mg/g)** |  | **k_2_** | **R^2^** | **q_e_, cal (mg/g)** |  |
| 100 | 59.708±0.005 | | -0.0001±6.44E^-05^ | 0.952 | 4.138±0.013 |  | 0.020±0.0001 | 1 | 57.972±0.038 |  |
| 250 | 137.684±0.002 | | -0.00031±1.59E^-05^ | 0.892 | 19.307±0.145 |  | 0.015±3.07E^-07^ | 1 | 137.934±0.003 |  |
| 300 | 142.415±0.002 | | -4.7E^-05^±6.3E^-07^ | 0.842 | 5.089±0.062 |  | 0.018±0.0001 | 1 | 140.81 ±0.120 |  |
| 500 | 319.622±0.006 | | -5E^-05^±5.64E^-07^ | 0.963 | 3.203±0.002 |  | 0.034±0.0001 | 1 | 318.472±0.0004 |  |

**S Table 20:**

**Analysis of equilibrium Cd (II) biosorption capacity of *Candida tropicalis* XTA1874 by Langmuir Isotherm Before Optimization**

| **Experiment No.** | **C_i_ (ppm)** | **C_e_ (ppm)** | **1/C_e_** | **logC_e_** | **Adsorbed**  **(ppm)** | **Absorbed**  **(ppm)** | **Intracellular accumulation (mg/g)** | **q_e_(mg/g)** | **1/q_e_** | **logq_e_** |  | **Equilibrium Biosorption capacity(%)(C_i_-C_e_)*100/C_i_** | **Mean Equilibrium Biosorption capacity(%)** |
| --- | --- | --- | --- | --- | --- | --- | --- | --- | --- | --- | --- | --- | --- |
| 1 | 15 | 3.214±0.008 | 0.3111 | 0.507 | 11.76±0.0003 | 0.01±0.0003 | 0.022±0.014 | 7.882±0.009 | 0.127 | 0.8966 |  | 78.652±0.018 |  |
| 2 | 45 | 10.804±0.003 | 0.092 | 1.034 | 34.15±0.0007 | 0.05±0.0002 | 0.039±0.003 | 22.427±0.0009 | 0.045 | 1.3507 |  | 74.755±0.0002 |  |
| 3 | 55 | 11.16±0.0004 | 0.089 | 1.048 | 43.77±0.0008 | 0.07±0.0001 | 0.047±0.0007 | 28.912±0.024 | 0.035 | 1.4611 |  | 78.783±0.001 | 75.007±0.001 |
| 4 | 100 | 20.32±0.0003 | 0.049 | 1.308 | 79.62±0.006 | 0.08±0.0003 | 0.054±0.002 | 52.864±0.044 | 0.012 | 1.7232 |  | 79.151±0.0004 |  |
| 5 | 250 | 62.34±0.0005 | 0.016 | 1.795 | 187.56±0.0006 | 0.99±0.0004 | 0.065±0.0007 | 124.616±0.002 | 0.008 | 2.0956 |  | 74.769±0.006 |  |
| 6 | 300 | 109.231±0.0008 | 0.009 | 2.038 | 190.64±0.0004 | 0.13±0.0004 | 0.088±0.001 | 126.414±0.001 | 0.00791 | 2.1018 |  | 63.207±0.0004 |  |
| 7 | 500 | 120.14±0.003 | 0.008 | 2.079 | 379.53±0.0007 | 0.33±0.0004 | 0.221±0.0005 | 252.287±0.005 | 0.00396 | 2.4019 |  | 75.684±0.0002 |  |

**S Table 21:**

**Values of the parameters of isotherm models for Cd(II) biosorption equilibrium of *Candida tropicalis* XTA 1874 Before Optimization**

| **Langmuir** | q_max_(mg/g) | K_L_ (L/mg) | R^2^ | R_L_ | Mean Removal (%) |
| --- | --- | --- | --- | --- | --- |
|  | 544.22±0.25 | 1.155±0.273 | 0.995 | 0.308±0.005- 0.935±0.002 | 75.007±0.002 |
| **Freundlich** | K_F_ (mg/g) | n | R^2^ |  |  |
|  | 3.182±0.016 | 1.143±0.007 | 0.941 |  |  |

**S Table 22:**

**Analysis of equilibrium Cd (II) biosorption capacity of *Candida tropicalis* XTA1874 by Langmuir Isotherm After Optimization**

| **Experiment No.** | **C_i_ (ppm)** | **C_e_ (ppm)** | **1/C_e_** | **logC_e_** | **Adsorbed**  **(ppm)** | **Absorbed**  **(ppm)** | **Intracellular accumulation (mg/g)** | **q_e_(mg/g)** | **1/q_e_** | **logq_e_** |  | **Equilibrium Biosorption capacity(%)(C_i_-C_e_)*100/C_i_** | **Mean Equilibrium Biosorption capacity(%)** |
| --- | --- | --- | --- | --- | --- | --- | --- | --- | --- | --- | --- | --- | --- |
| 1 | 15 | 1.31±0.002 | 0.763 | 0.117 | 13.68±0.008 | 0.017±0.002 | 0.014± 0.0003 | 9.153±0.018 | 0.109 | 0.962 |  | 91.328±0.012 |  |
| 2 | 45 | 2.81±0.003 | 0.356 | 0.448 | 42.14±0.001 | 0.06±0.0003 | 0.041± 0.0005 | 28.104±0.011 | 0.035 | 1.448 |  | 93.778±0.002 |  |
| 3 | 55 | 5.16±0.0001 | 0.194 | 0.713 | 49.73±0.0009 | 0.11±0.0004 | 0.112± 0.0006 | 33.225±0.005 | 0.030 | 1.521 |  | 90.623±0.003 | 88.077±0.097 |
| 4 | 100 | 10.32±0.0002 | 0.097 | 1.014 | 89.58±0.013 | 0.13±0.0005 | 0.094± 0.005 | 59.708±0.005 | 0.017 | 1.776 |  | 89.681±0.0007 |  |
| 5 | 250 | 43.34±0.0001 | 0.023 | 1.637 | 206.53±0.004 | 0.14±0.0002 | 0.095±0.01 | 137.684±0.002 | 0.0072 | 2.138 |  | 82.665±0.001 |  |
| 6 | 300 | 86.23±0.0001 | 0.011 | 1.936 | 213.62±0.001 | 0.15±0.0002 | 0.085±0.023 | 142.415±0.002 | 0.007 | 2.154 |  | 71.256±0.0004 |  |
| 7 | 500 | 20.14±0.0001 | 0.049 | 1.304 | 479.44±0.016 | 0.44±0.0004 | 0.306±0.006 | 319.621±0.006 | 0.003 | 2.505 |  | 95.972±0.0001 |  |

**S Table 23:**

**Values of the parameters of isotherm models for Cd(II) biosorption equilibrium of *Candida tropicalis* XTA 1874 After Optimization**

| **Langmuir** | q_max_(mg/g) | K_L_ (L/mg) | R^2^ | R_L_ | Mean Removal (%) |
| --- | --- | --- | --- | --- | --- |
|  | 885.686±0.26 | 1.612±0.478 | 0.966 | 0.205±0.009-0.881±0.028 | 88.077±0.097 |
| **Freundlich** | K_F_ (mg/g) | n | R^2^ |  |  |
|  | 11.721±0.002 | 1.433±0.004 | 0.759 |  |  |

**S Table 24:**

**Statistical Analysis of the significance of the before and after optimization model by Student’s T-test (n=6)**

| **Before Optimization (q_max_)** | **After Optimization**  **(q_max_)** | **T-test** | **Significance at**  **(*p*≤0.05)** | **Before Optimization Mean Removal (%)** | **After Optimization**  **Mean Removal (%)** | **T-test** | **Significance at**  **(*p*≤0.05)** |
| --- | --- | --- | --- | --- | --- | --- | --- |
| 543.478 | 884.956 | 4.43E^-26^ | significant | 75.003 | 87.901 | 1.28E^-17^ | significant |
| 544.325 | 886.247 |  |  | 75.011 | 87.934 |  |  |
| 545.035 | 886 |  |  | 75 | 88.466 |  |  |
| 543.665 | 885.583 |  |  | 75.01 | 88.283 |  |  |
| 543.997 | 886.382 |  |  | 75.014 | 87.934 |  |  |
| 544.821 | 884.945 |  |  | 75 | 87.945 |  |  |

**S Table 25:**

**Estimation of desorption capacity( η %) and the regeneration capacity of the biomass**

| **Number of Cycles** | **C_r_** | **C_i_** | **C_e_** | **V** |  | **Adsorbed (ppm)** | **Absorbed (ppm)** | **Surface accumulation (mg/g)** |  | **Intracellular accmulation**  **(mg/g)** | **Vr** | **Removal (%)** | **η (%)** |
| --- | --- | --- | --- | --- | --- | --- | --- | --- | --- | --- | --- | --- | --- |
| 1 | 422.28±0.0006 | 500 | 20.14±0.0002 | 0.1 |  | 479.785±0.073 | 0.439±0.0002 | 319.614±0.0003 |  | 0.294±0.001 | 0.104 | 96.096±0.073 | 91.648±0.197 |
| 2 | 362.28±0.0003 | 500 | 72.281±0.0004 | 0.1 |  | 427.678±0.039 | 0.239±0.0002 | 284.987±0.0003 |  | 0.161±0.0005  0.107±0.0005 | 0.103 | 85.577±0.012 | 87.251±0.078 |
| 3 | 302.28±0.0001 | 500 | 100.282±0.0005 | 0.1 |  | 399.689±0.026 | 0.159±0.0001 | 266.373±0.0001 |  | 0.014±0.0001 | 0.103 | 80.008±0.044 | 77.876±0.018 |
| 4 | 298.28±0.0005 | 500 | 102.364±0.0009 | 0.1 |  | 397.632±0.002 | 0.02±0.00003 | 258.355±0.0001 |  | 0.005±0.0001 | 0.102 | 79.727±0.16 | 76.565±0.019 |
| 5 | 282.28±0.0002 | 500 | 112.46±0.0002 | 0.1 |  | 387.532±0.0003 | 0.008±0.0003 | 258.355±0.0001 |  | 0.005±0.0001 | 0.102 | 77.514±0.002 | 74.394±0.06 |
| 6 | 281.95±0.0005 | 500 | 112.46±0.0002 | 0.1 |  | 387.532±0.0002 | 0.008±0.0001 |  |  |  | 0.102 | 77.514±0.002 | 74.237±0.016 |

**S Table 26:**

**Kinetic analysis of Cd(II) Desorption using 387.532ppm Cd(II)**

| **Experiment No.** | **Time (min)** | **q_i_ (mg/g)** | **q_t_ (mg/g)** | **C_a_(ppm)** | **1/C_a_** | **lnt** | **t^0.5^** |
| --- | --- | --- | --- | --- | --- | --- | --- |
| 1 | 0 | 0 | 0 | 0 | 0 | 0 | 0 |
| 2 | 40 | 258.3547 | 0.003 | 387.5275 | 0.00258 | 3.688879 | 6.324555 |
| 3 | 60 | 258.3547 | 0.0022 | 387.5287 | 0.00258 | 4.094345 | 7.745967 |
| 4 | 80 | 258.3547 | 0.0014 | 387.5299 | 0.00258 | 4.382027 | 8.944272 |
| 5 | 100 | 258.3547 | 0.00082 | 387.5308 | 0.00258 | 4.60517 | 10 |
| 6 | 150 | 258.3547 | 0.00075 | 387.5309 | 0.00258 | 5.010635 | 12.24745 |
| 7 | 180 | 258.3547 | 0.00075 | 387.5309 | 0.00258 | 5.192957 | 13.41641 |
| 8 | 200 | 258.3547 | 0.00075 | 387.5309 | 0.00258 | 5.298317 | 14.14214 |
| 9 | 250 | 258.3547 | 0.00075 | 387.5309 | 0.00258 | 5.521461 | 15.81139 |
| 10 | 280 | 258.3547 | 0.00075 | 387.5309 | 0.00258 | 5.63479 | 16.7332 |
| 11 | 300 | 258.3547 | 0.00075 | 387.5309 | 0.00258 | 5.703782 | 17.32051 |
| 12 | 350 | 258.3547 | 0.00075 | 387.5309 | 0.00258 | 5.857933 | 18.70829 |
| 13 | 380 | 258.3547 | 0.00075 | 387.5309 | 0.00258 | 5.940171 | 19.49359 |
| 14 | 400 | 258.3547 | 0.00075 | 387.5309 | 0.00258 | 5.991465 | 20 |
| 15 | 450 | 258.3547 | 0.00075 | 387.5309 | 0.00258 | 6.109248 | 21.2132 |
| 16 | 480 | 258.3547 | 0.00075 | 387.5309 | 0.00258 | 6.173786 | 21.9089 |
| 17 | 500 | 258.3547 | 0.00075 | 387.5309 | 0.00258 | 6.214608 | 22.36068 |

**S Table 27:**

**Estimated Desorption kinetics parameters**

| **Model** | **Equation** | **Parameters** | **Ca_0exp_** | **Ca_0cal_** | **α** | **β** | **R^2^** | **SE** |
| --- | --- | --- | --- | --- | --- | --- | --- | --- |
| Parabolic Diffusion Model | 1/C_a_=1/C_a0_-K_a2_t | C_a_, Cd(II) released at Time t  C_a0_, Cd(II) concentration in solution when all ions released | 387.532 | 387.5346±0.004 | - | - | 0.86566 | 0.014 |
| Elovich Type Model | C_a_= (1/β)ln(αβ)+(1/β)lnt | α, initial Cd(II) ion desorption rate (mgL^-1^min^-1^)  β, desorption rate constant (mgg^-1^) | 387.532 | 387.526±0.002 | 1.98E^+168^ | 359.712 | 0.92158 | 0.009 |


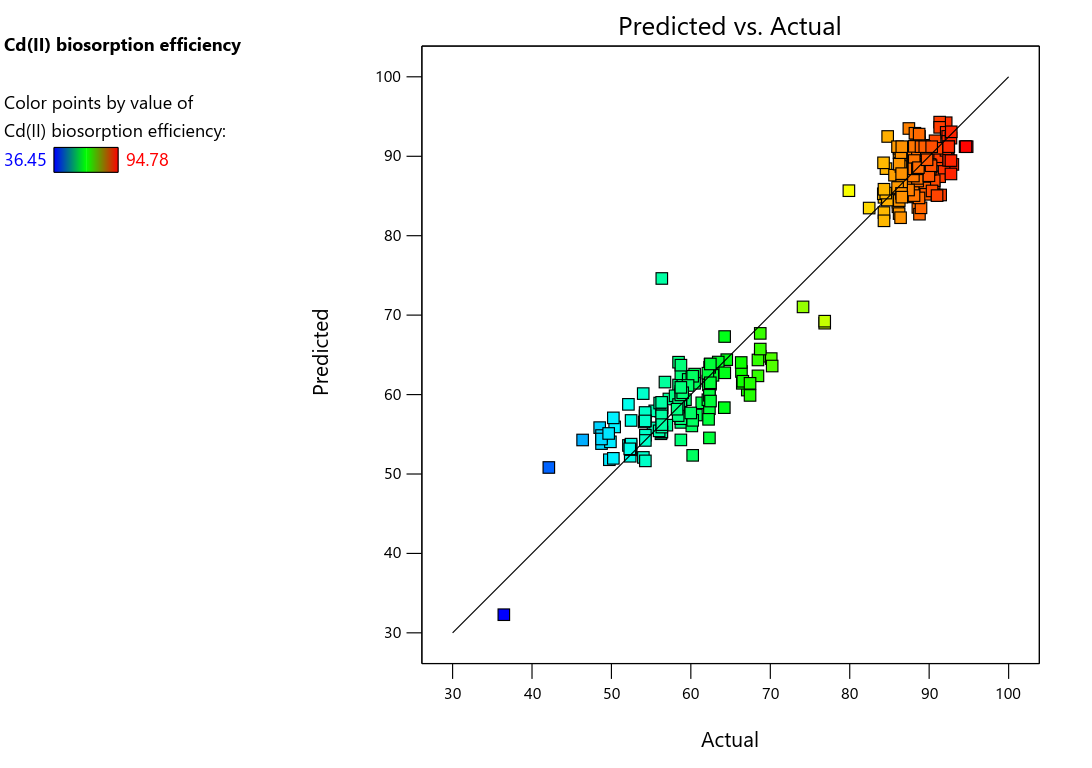


**S Fig. 1.** Comparison of predicted vs. actual values for Cd(II) biosorption by *Candida tropicalis* XTA1874


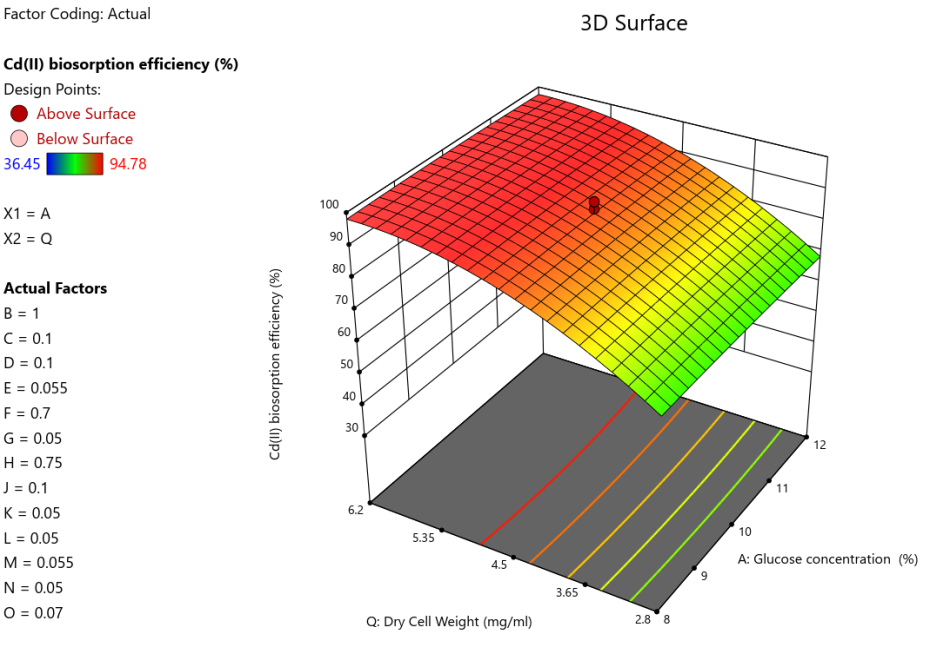

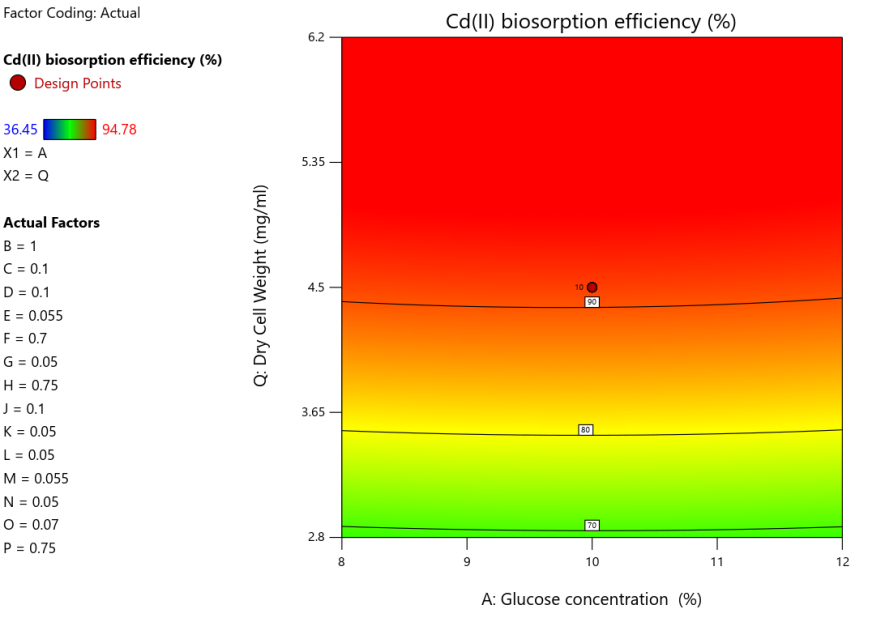


A1

A2


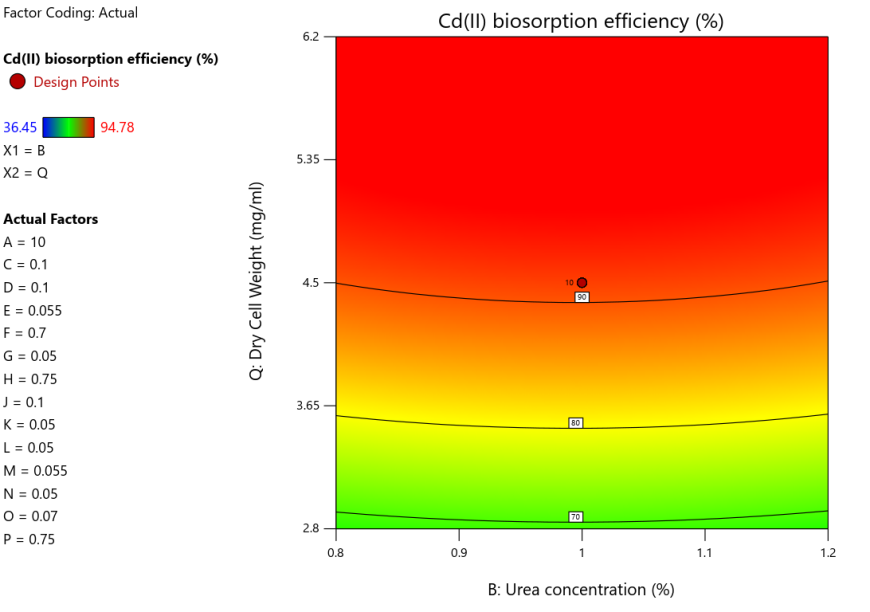

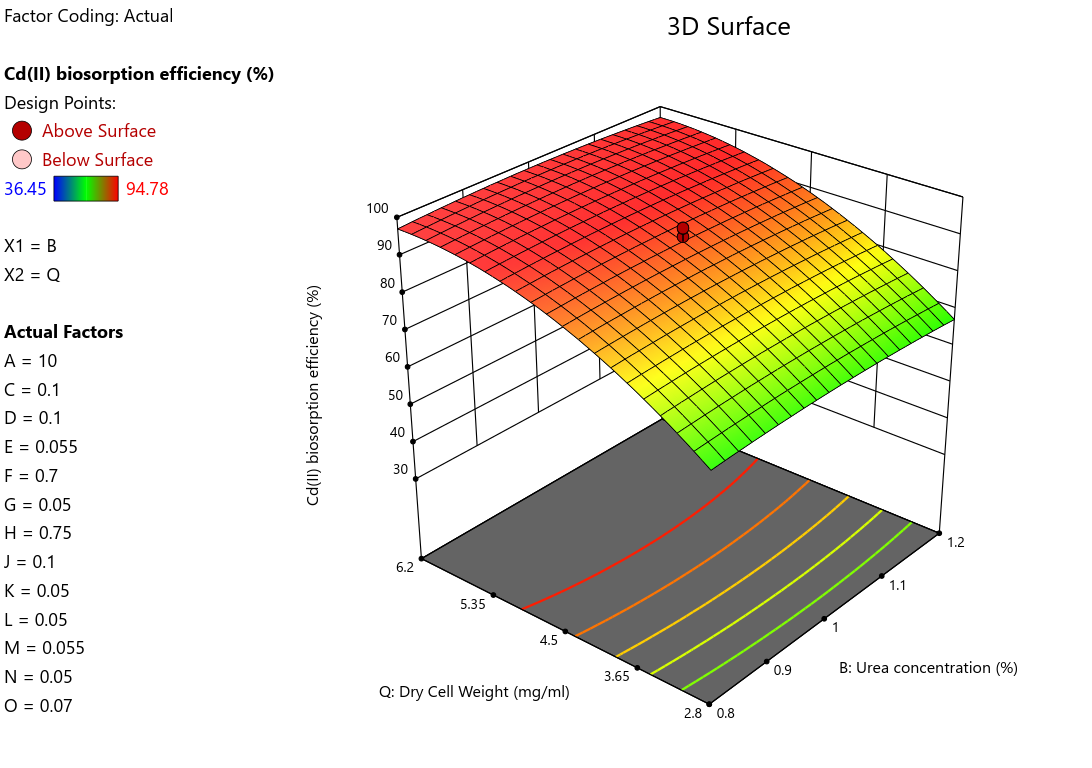


B1

B2


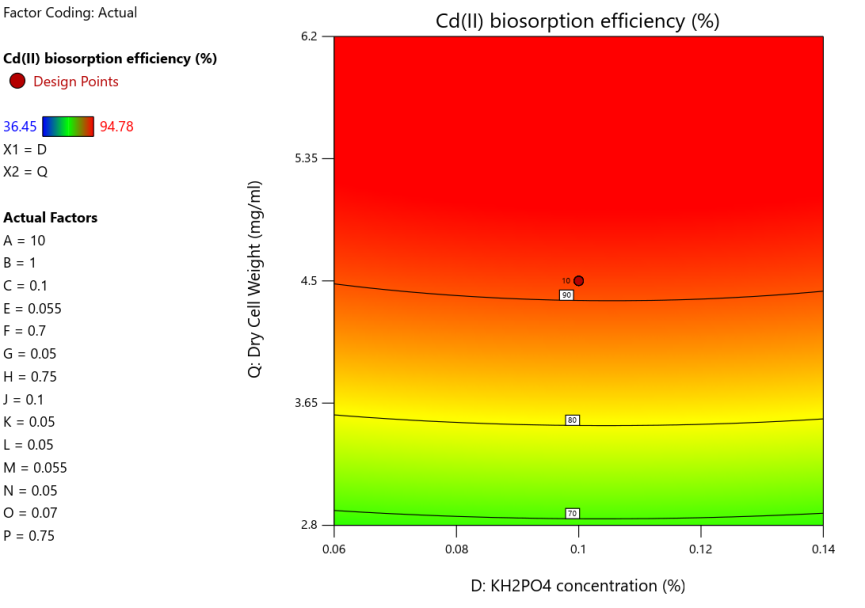

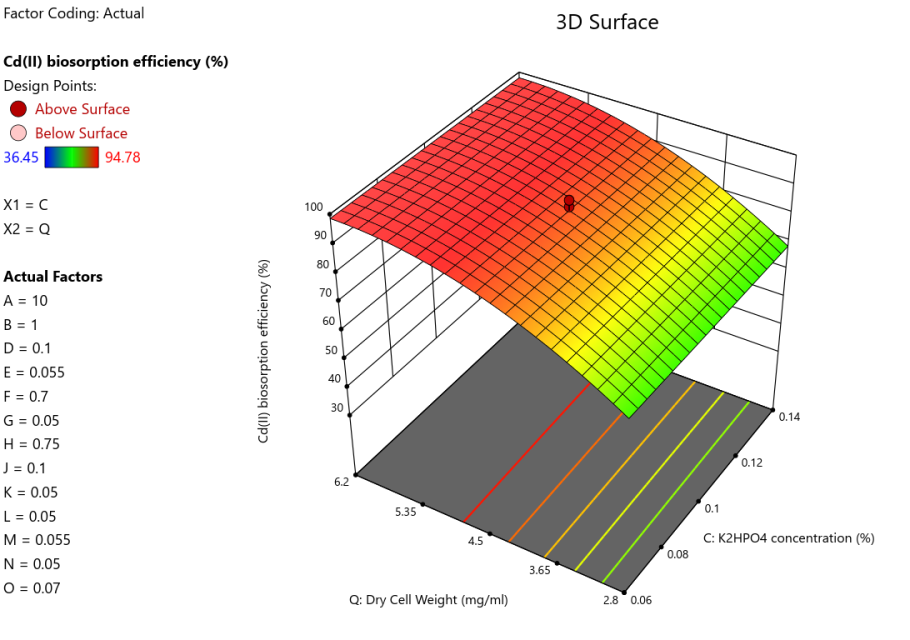


C2

C1


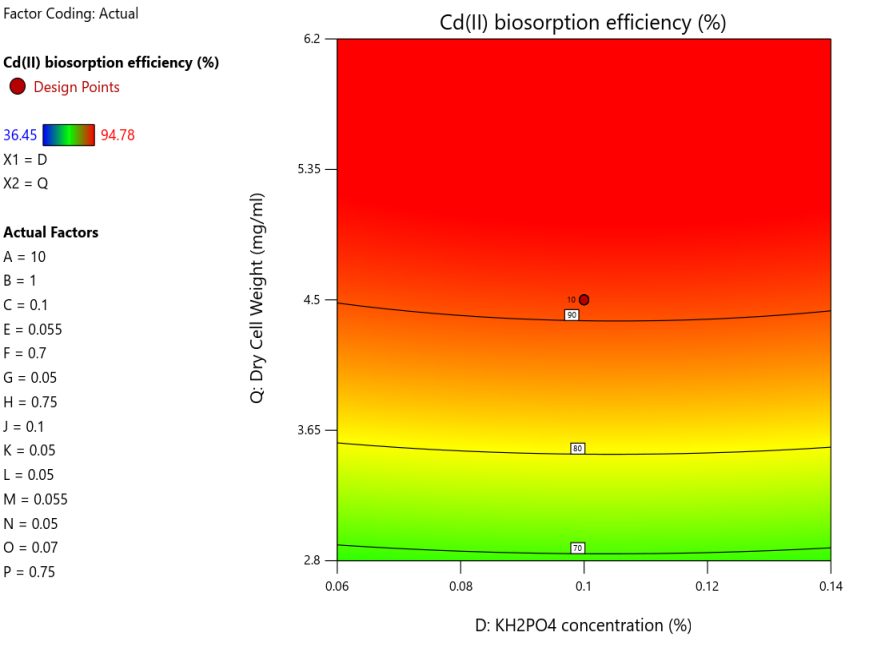

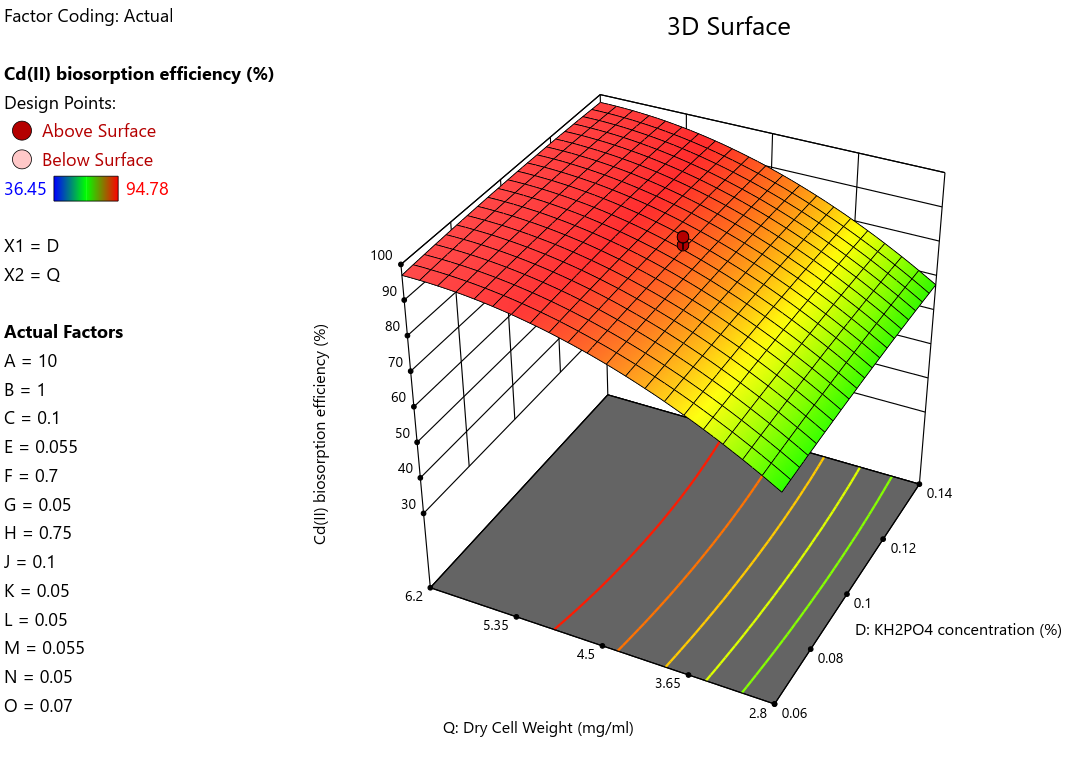


D2

D1


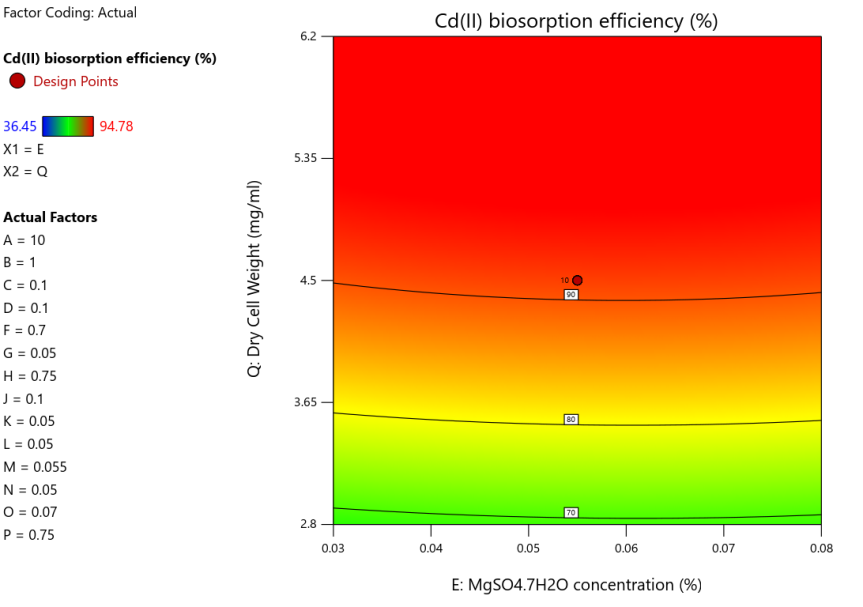

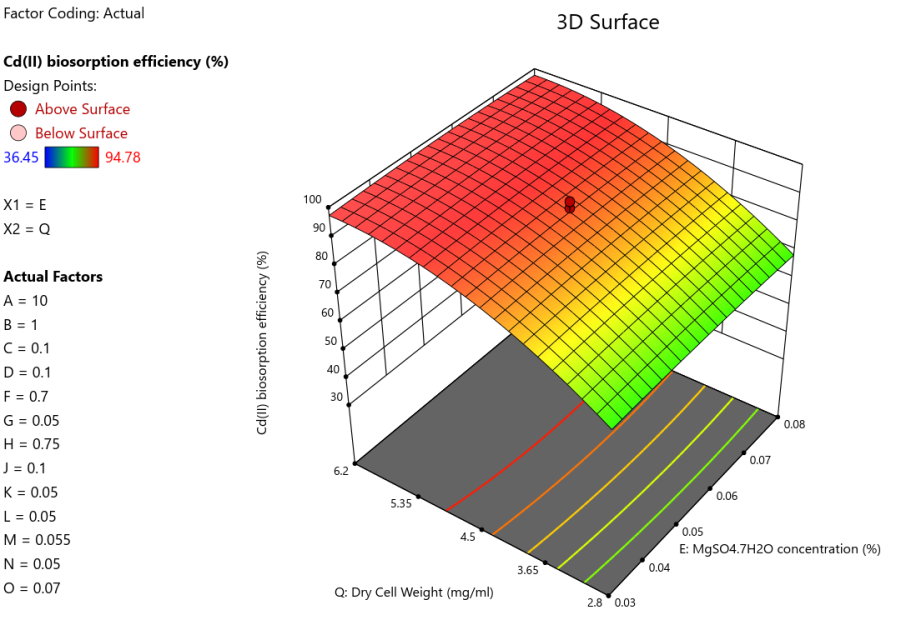


E2

E1


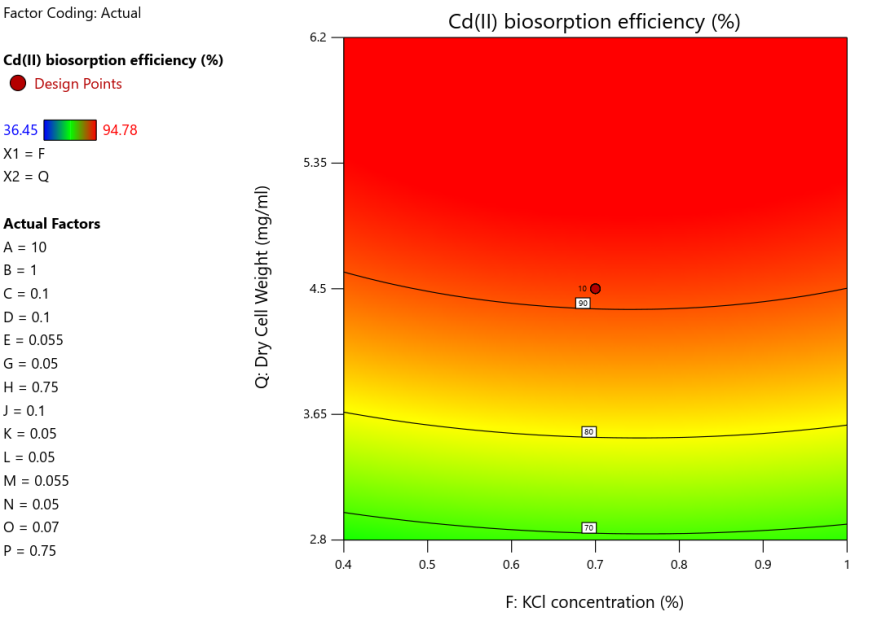

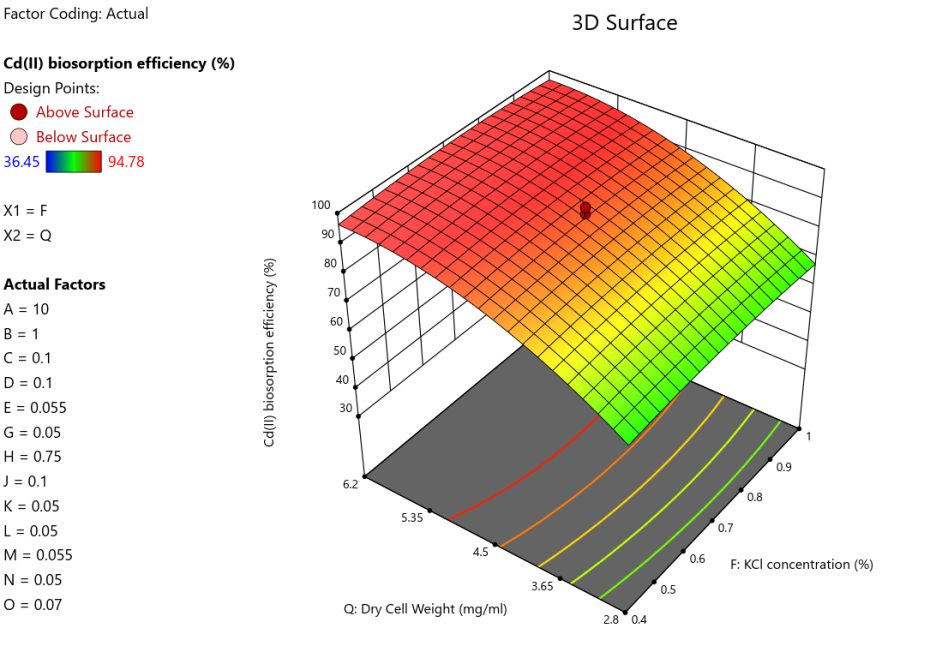


F1

F2


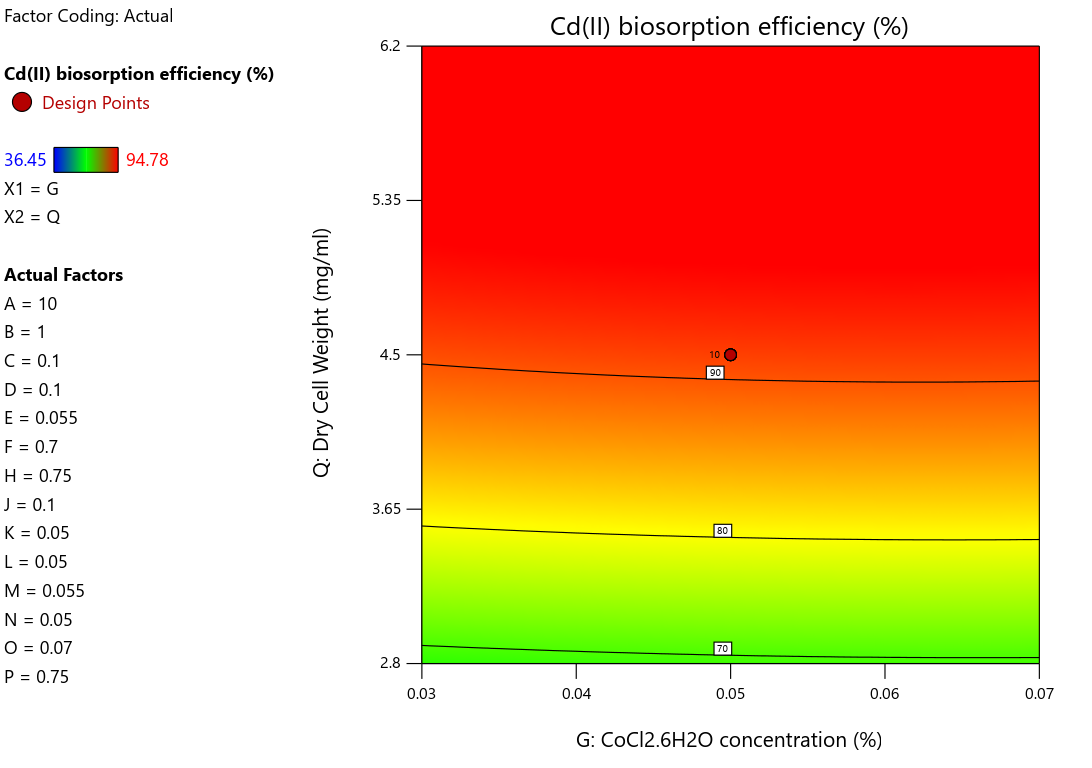

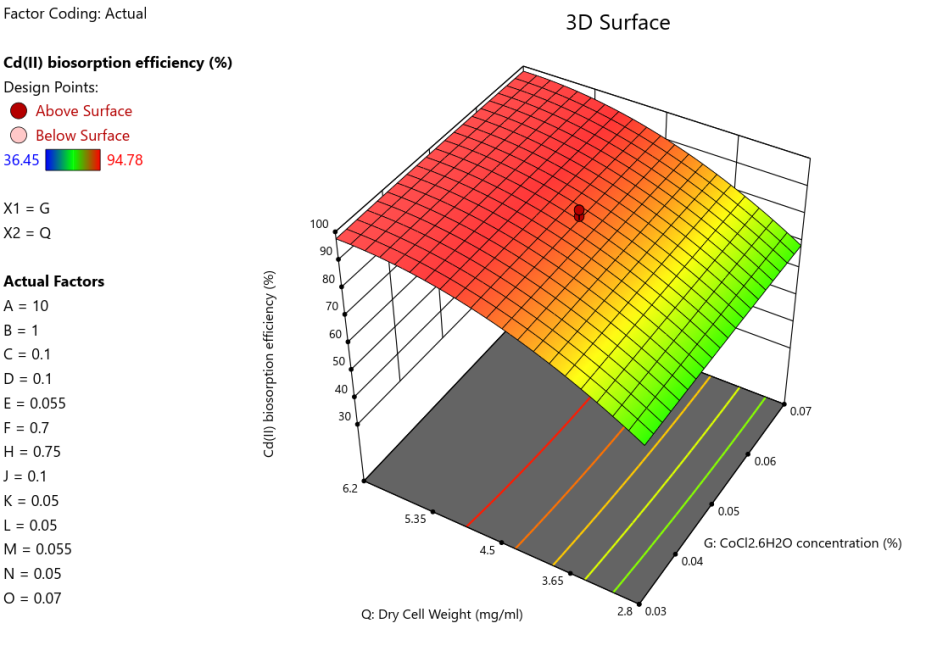


G1

G2


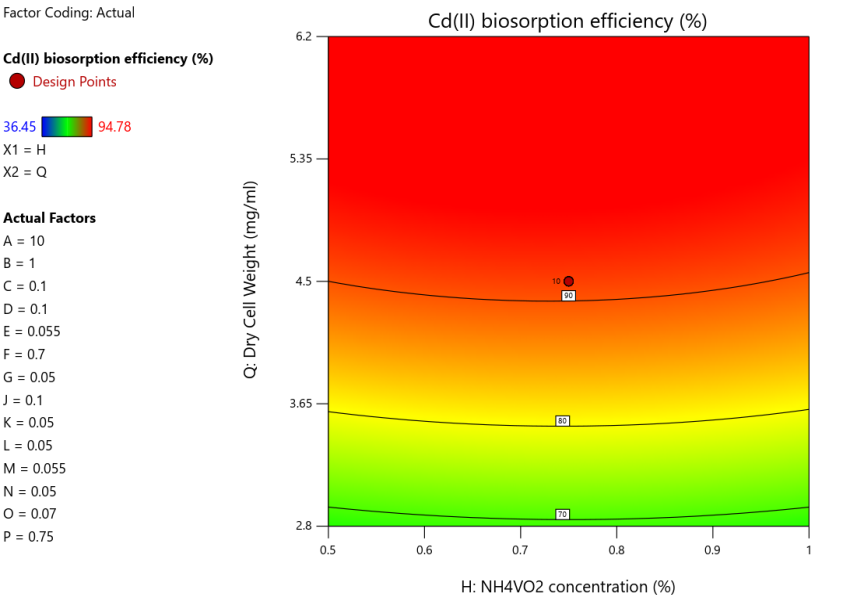

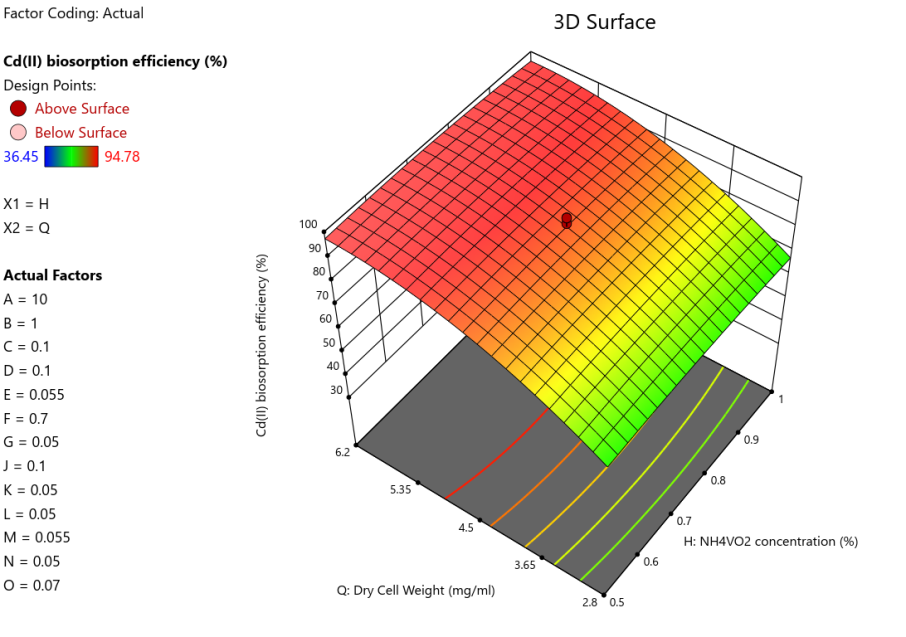


H2

H1


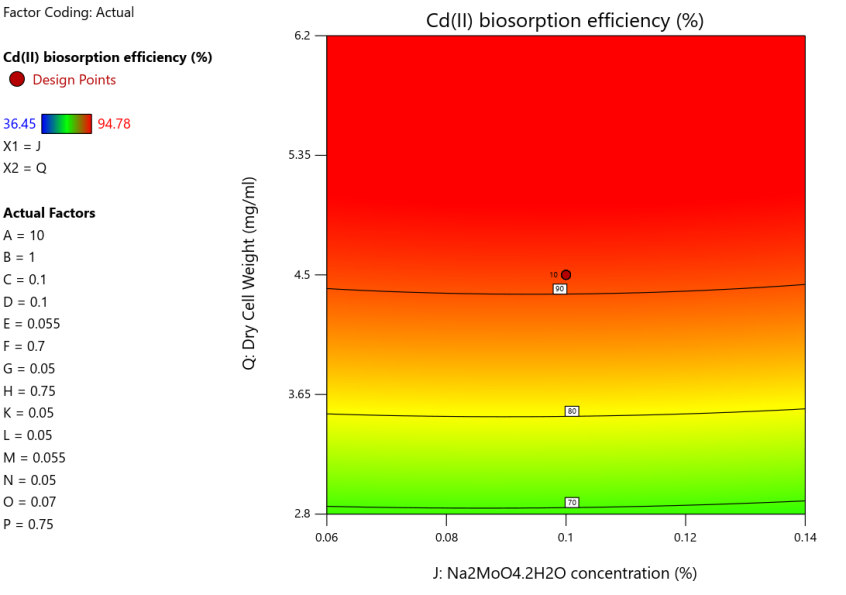

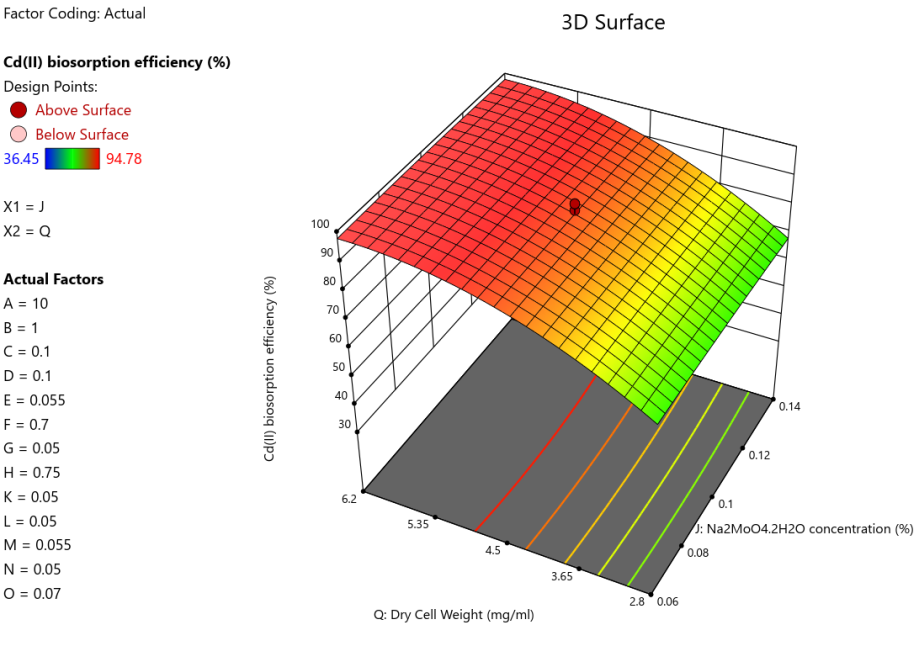


I2

I1


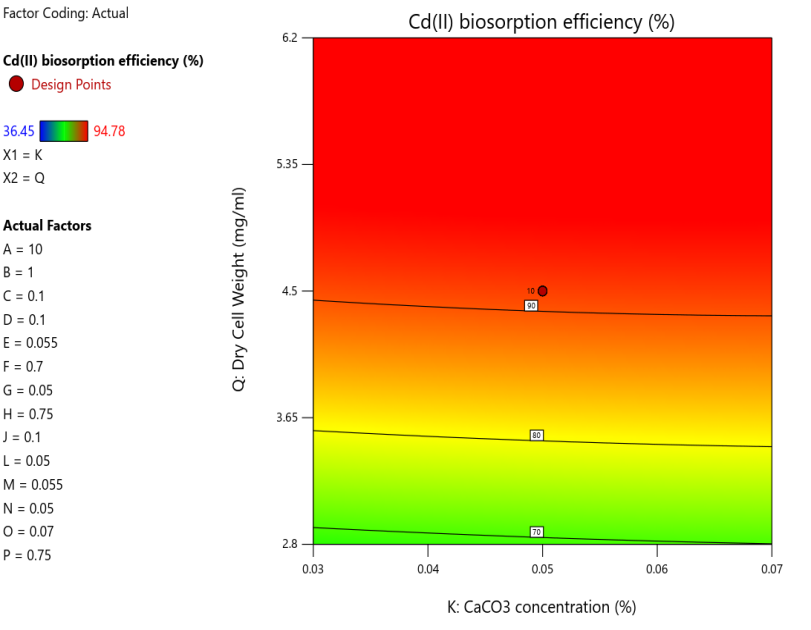

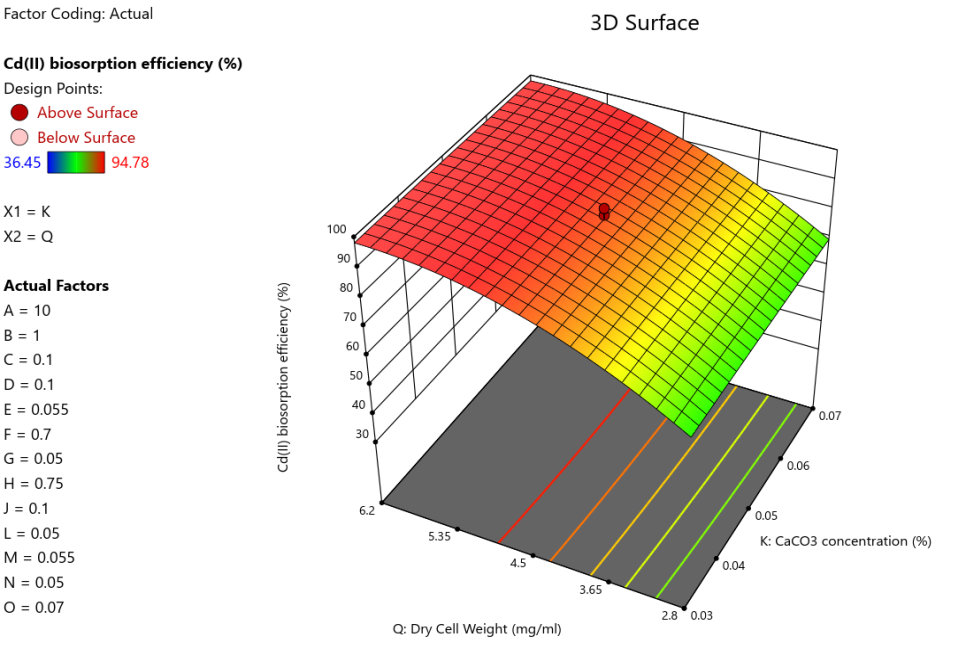


J2

J1


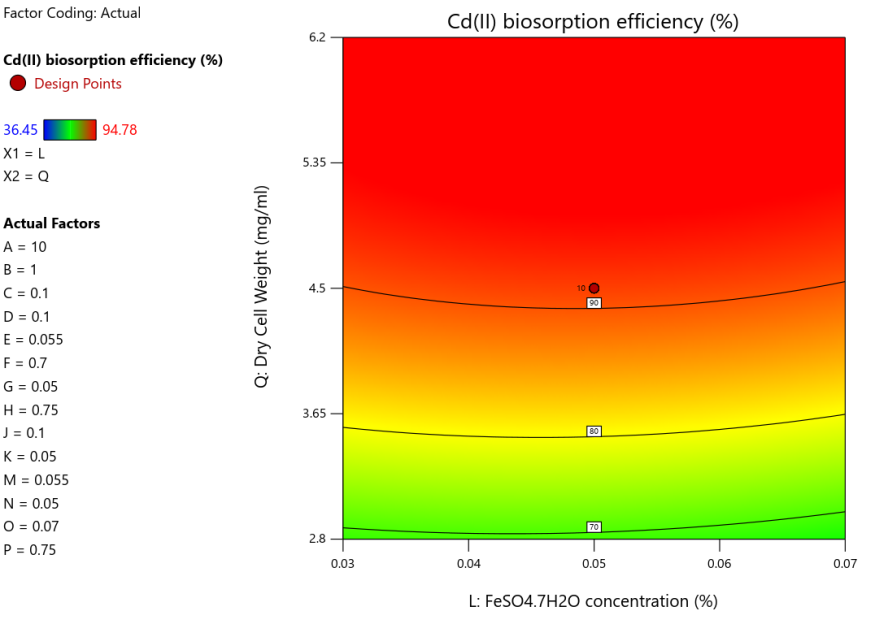

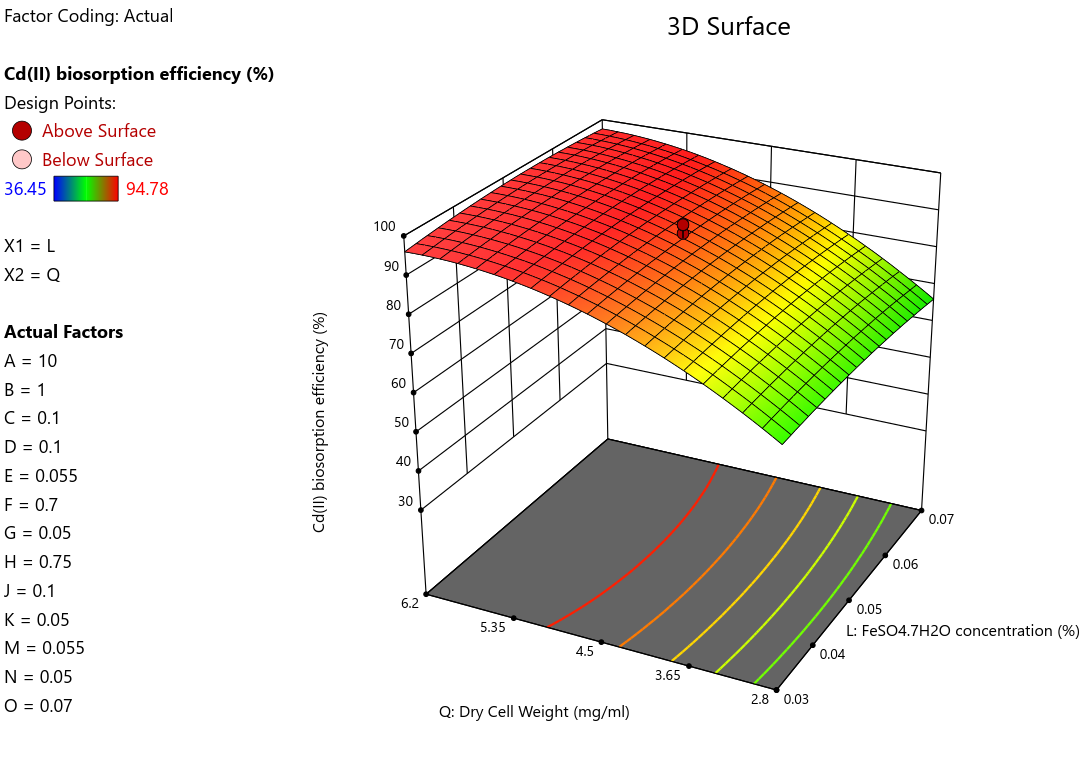


K2

K1


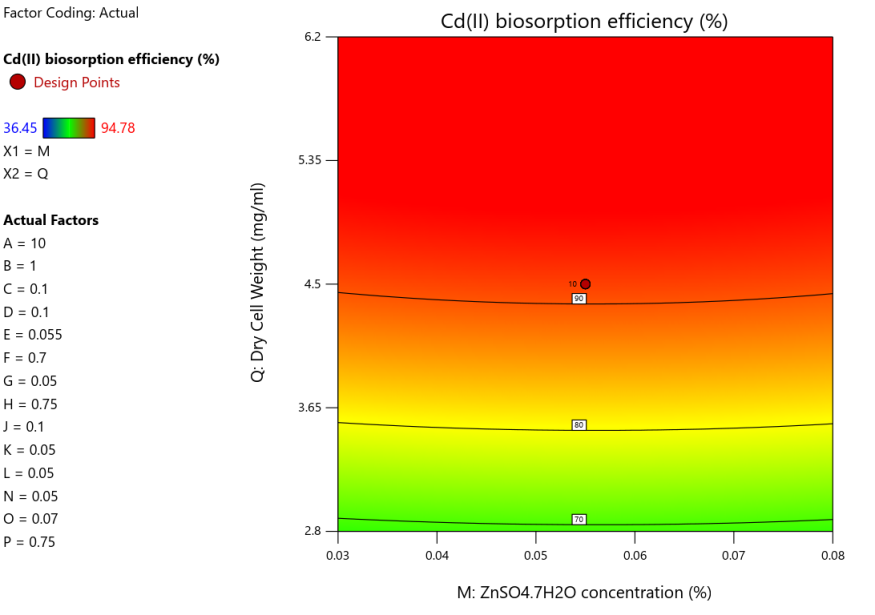

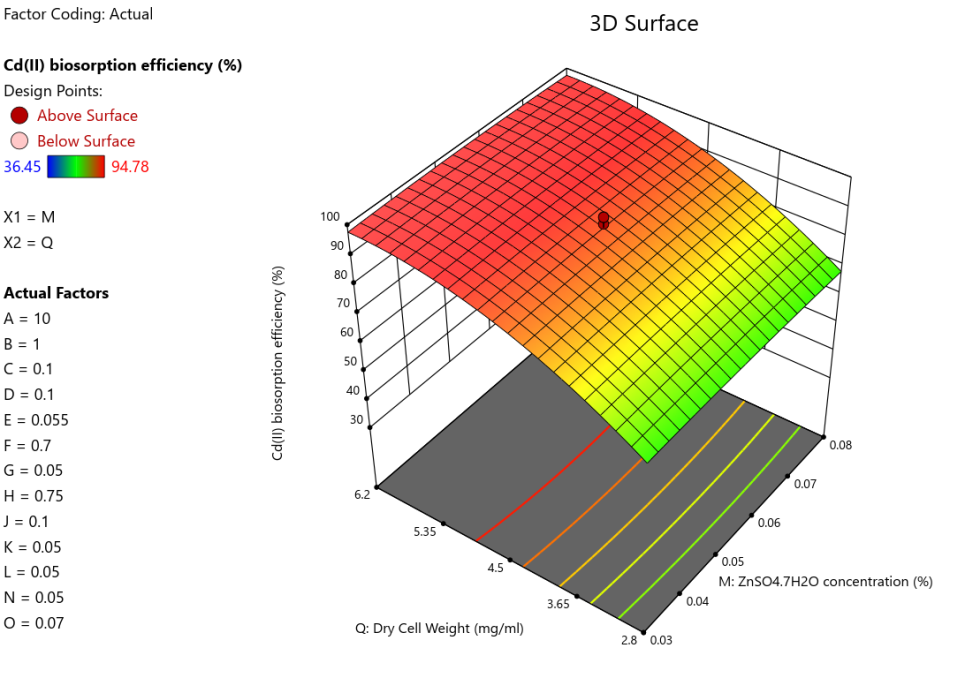


L2

L1


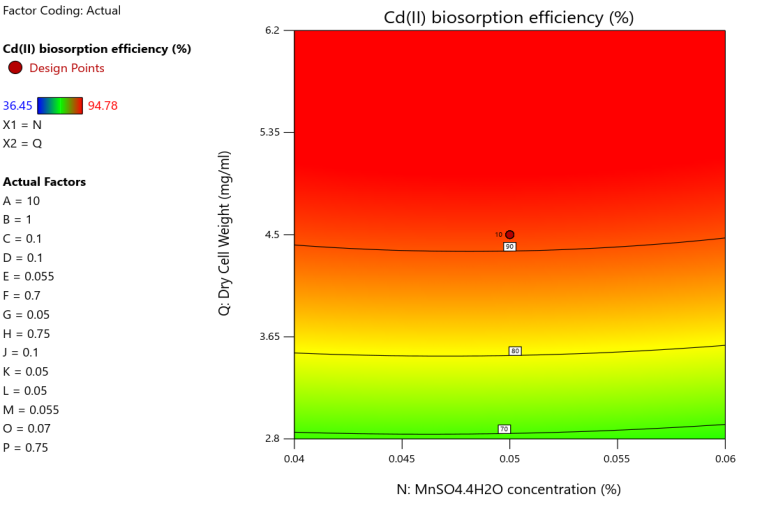

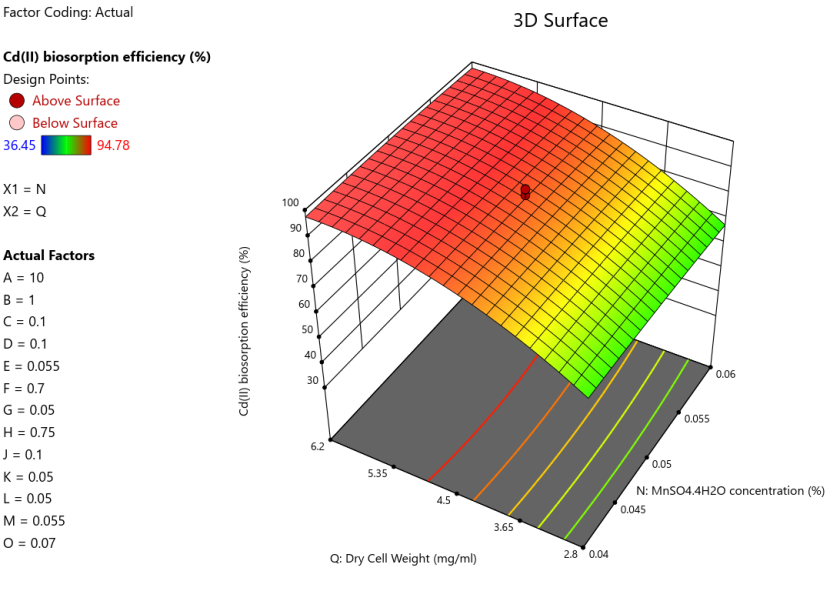


M2

M1


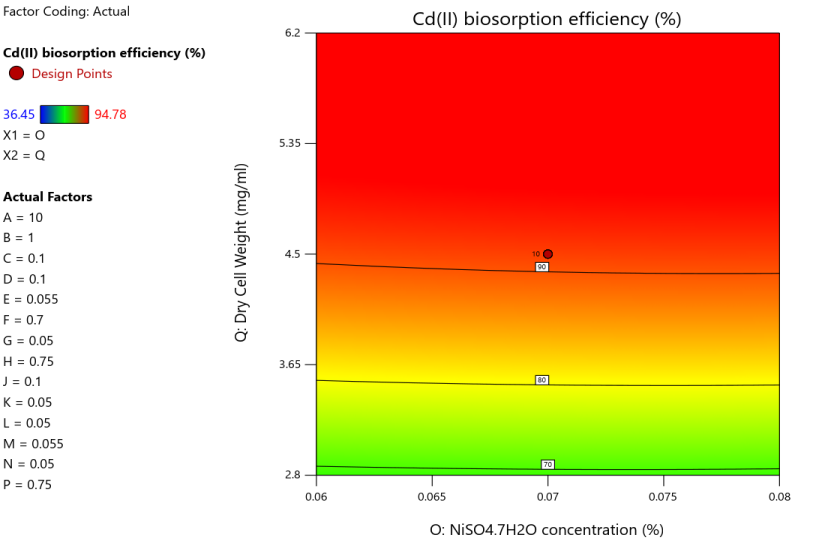

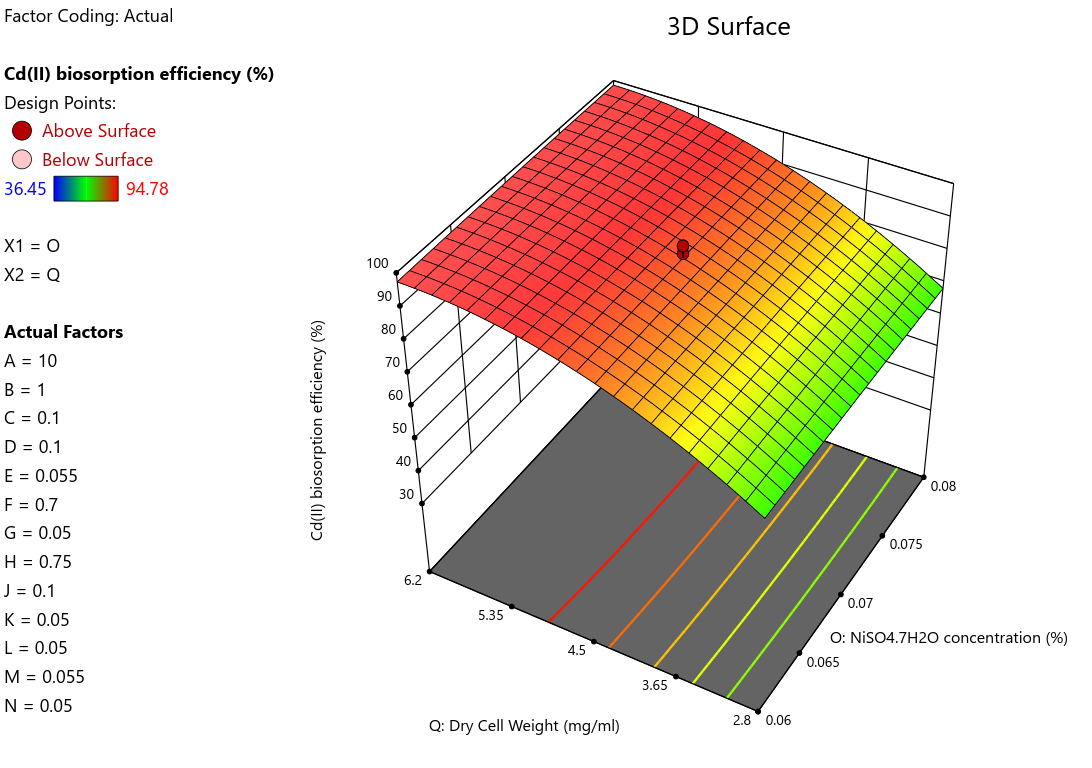


N2

N1


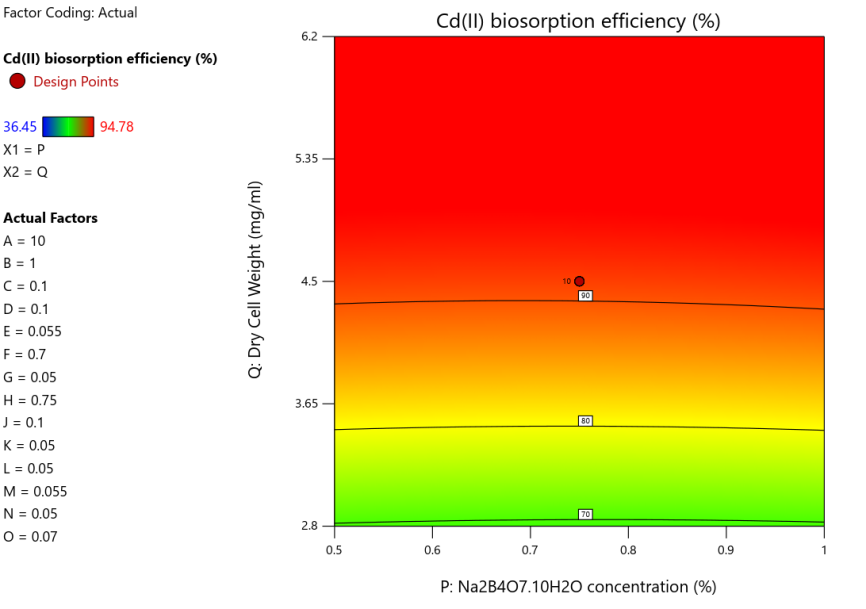

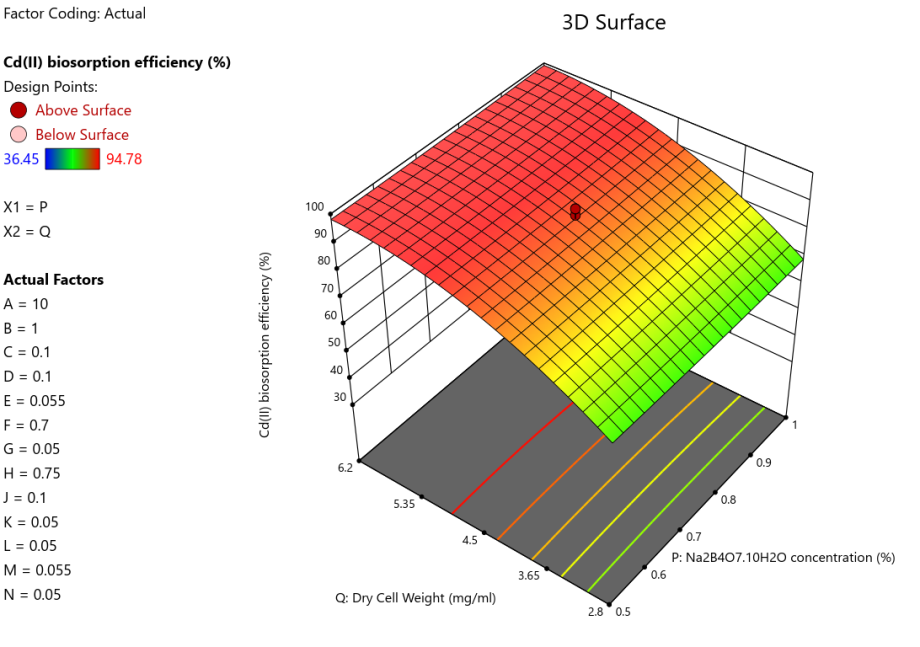


O2

O1

**S Fig. 2.** Contour and 3D Response Surface Plots For Surface Optimization of (A1 and A2) Glucose concentration (%) (B1 and B2) Urea concentration (%) (C1 and C2) K_2_HPO_4_ concentration (%) (D1 and D2) KH_2_PO_4_ concentration (%) (E1 and E2) MgSO_4_.7H_2_O concentration (%) (F1 and F2) KCl concentration (%) (G1 and G2) CoCl_2_.6H_2_O concentration (%) (H1 and H2) NH_4_VO_2_ concentration (%) (I1 and I2) Na_2_MoO_4_ concentration (%) (J1 and J2) CaCO_3_ concentration (%) (K1 and K2) FeSO_4_.7H_2_O concentration (%) (L1 and L2) ZnSO_4_.7H_2_O concentration (%) (M1 and M2) MnSO_4_.7H_2_O concentration (%) (N1 and N2) NiSO_4_.7H_2_O concentration (%) (O1 and O2) Na_2_B_4_O_7_concentration (%)

B

A

**S Fig. 3.** Linear Plots For Pseudo First (A) and Second Order (B) Kinetic Models For Cd(II) Biosorption by the strain *Candida tropicalis* XTA1874 Before Optimization

D

C

**S Fig. 4.** Linear Plots For Pseudo First (C) and Second Order (D) Kinetic Models For Cd(II) Biosorption by the strain *Candida tropicalis* XTA1874 After Optimization

F

E

**S Fig. 5.** Linear Plots For Langmuir (E) and Freundlich (F) Isotherm Model For Cd(II) Biosorption by the strain *Candida tropicalis* XTA1874 Before and After Optimization

**S Fig. 6.** FT-IR Analysis For Cd(II) Biosorption by the live cells of the Cd(II) resistant strain *Candida tropicalis* XTA1874 after (AO-LC) and before optimization (BO-LC)

**
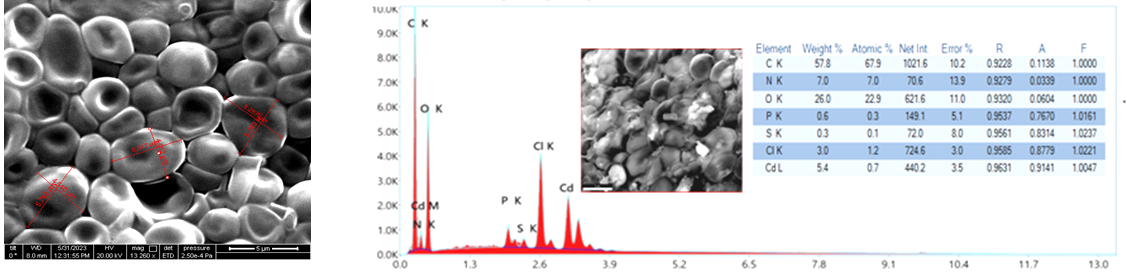
**

A

**
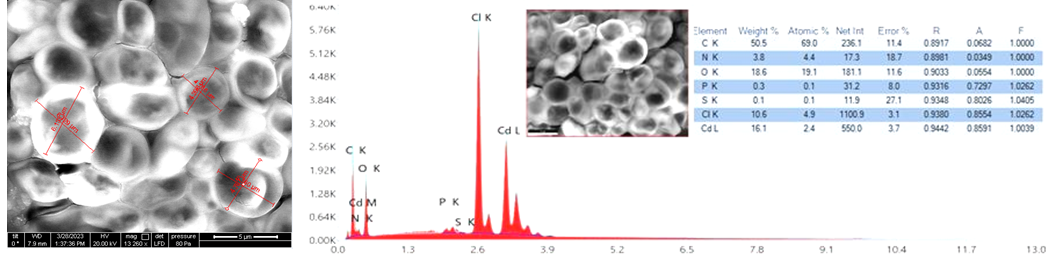
**

B

**S Fig. 7.** FE-SEM and EDAX Analyses For Cd(II) Biosorption by the live cells of the Cd(II) resistant strain *Candida tropicalis* XTA1874 before (A) and after optimization (B)

**S Fig. 8.** Graphical representation of the desorption efficiences with the number of cycles

H

G

**S Fig. 9.** Evaluation of Cd(II) desorption kinetics by Parabolic diffusion (G) and Elovich Model (H) by Cd(II) resistant strain *Candida tropicalis* XTA1874

**
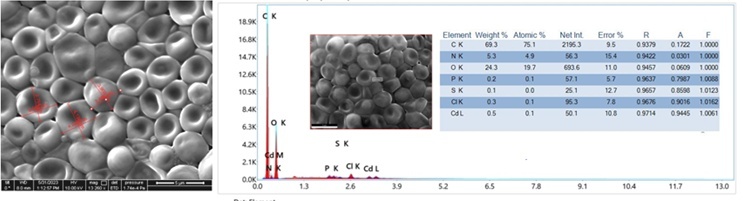
**

**S Fig. 10.** FE-SEM and EDAX Analyses of developed Cd(II) resistant strain *Candida tropicalis* XTA1874 After Desorption
